# Single-cell analysis reveals immune landscape in kidneys of patients with chronic transplant rejection

**DOI:** 10.7150/thno.48201

**Published:** 2020-07-11

**Authors:** Yongguang Liu, Jianmin Hu, Ding Liu, Song Zhou, Jun Liao, Guorong Liao, Siqiang Yang, Zefeng Guo, Yuzhu Li, Shichao Li, Hua Chen, Ying Guo, Ming Li, Lipei Fan, Liuyang Li, Anqi Lin, Ming Zhao

**Affiliations:** Department of Organ Transplantation, Zhujiang Hospital, Southern Medical University, Guangzhou, Guangdong, China.

**Keywords:** Chronic kidney transplant rejection, Single-cell RNA sequencing, Immune landscape, Kidney, Graft

## Abstract

**Rationale:** Single-cell RNA sequencing (scRNA-seq) has provided an unbiased assessment of specific profiling of cell populations at the single-cell level. Conventional renal biopsy and bulk RNA-seq only average out the underlying differences, while the extent of chronic kidney transplant rejection (CKTR) and how it is shaped by cells and states in the kidney remain poorly characterized. Here, we analyzed cells from CKTR and matched healthy adult kidneys at single-cell resolution.

**Methods:** High-quality transcriptomes were generated from three healthy human kidneys and two CKTR biopsies. Unsupervised clustering analysis of biopsy specimens was performed to identify fifteen distinct cell types, including major immune cells, renal cells and a few types of stromal cells. Single-sample gene set enrichment (ssGSEA) algorithm was utilized to explore functional differences between cell subpopulations and between CKTR and normal cells.

**Results:** Natural killer T (NKT) cells formed five subclasses, representing CD4+ T cells, CD8+ T cells, cytotoxic T lymphocytes (CTLs), regulatory T cells (Tregs) and natural killer cells (NKs). Memory B cells were classified into two subtypes, representing reverse immune activation. Monocytes formed a classic CD14+ group and a nonclassical CD16+ group. We identified a novel subpopulation [myofibroblasts (MyoF)] in fibroblasts, which express collagen and extracellular matrix components. The CKTR group was characterized by increased numbers of immune cells and MyoF, leading to increased renal rejection and fibrosis.

**Conclusions:** By assessing functional differences of subtype at single-cell resolution, we discovered different subtypes that correlated with distinct functions in CKTR. This resource provides deeper insights into CKTR biology that will be helpful in the diagnosis and treatment of CKTR.

## Introduction

Kidney transplantation is one of the most effective methods for the treatment of end-stage renal disease. The early and late immune responses to allografts are different processes. However, the pathogenesis of CKTR (mainly from a late immune response) remains poorly characterized. The long-term effect of renal transplantation has not been substantially improved in 20 years 1-3. Fibrointimal thickening of the arteries, interstitial fibrosis and tubular atrophy seriously affect not only graft function but also survival 4,5. Traditional bulk RNA-seq and renal biopsy approaches reflect the average gene expression, not the types and status at the single-cell level, thereby neglecting the heterogeneity of the transcriptome at single-cell resolution 6.

scRNA-seq has been extensively developed, allowing expression profiles of individual cell types to be obtained rapidly. It plays an important role in identifying cell subtypes and illustrating molecular differences 7-9. More recently, scRNA-seq has revealed a comprehensive portrait of cancer cells via the growth and differentiation of cells. It also provides new insights into the pathogenesis of renal diseases 10,11. For instance, a single-cell profile of systemic lupus erythematosus with nephritis revealed that the highly expressed interferon-inducible genes in renal tubular cells were associated with disease severity 12. Another study identified three distinct endothelial subclusters generated from mixed renal rejection by scRNA-seq 11. The complex interactions between the immune system and renal cells play an important role in CKTR 13. Bulk transcriptional analysis results have indicated that antibody-mediated rejection (AMR) is the most common driver of late allograft loss 14. However, it is unable to uncover transcriptional profiles of individual cells, nor can it be used for the molecular characterization of CKTR 14.

Hence, this study provides a remarkably comprehensive catalog of cell types by characterizing their molecular functions, providing insights into CKTR biology that will be helpful in kidney transplantation. By analyzing single cells using an unsupervised clustering algorithm at a much higher resolution, we identified diverse states of immune and stromal cells involved in CKTR. Additionally, we uncovered the distinct function of immune cell subclasses in CKTR and healthy adult kidney samples.

## Materials and Methods

### Chronic kidney transplantation rejection samples

Our study received approval from the Institutional Review Board (IRB) at Zhujiang Hospital of Southern Medical University. The two patients described in this study provided informed consent. The first transplantation recipient was a 30-year-old male with two-fold higher serum creatinine and high panel reactive antibodies (PRA) (class I: 28%; class II: 41%) in the biopsy specimen, for which the histologic read was chronic rejection (tubular atrophy and moderate interstitial fibrosis). The second recipient was a 53-year-old female with high PRA (class II: 11%) in the biopsy specimen, for which the histologic read was chronic rejection (tubular atrophy and mild interstitial fibrosis). Detailed information on the two patients is provided in Supplementary Table S1.

### Healthy adult kidney samples

Healthy adult kidney scRNA-seq data were collected from the Gene Expression Omnibus database 6 (Accession ID: GSE131685) for three samples (barcodes.tsv, features.tsv and gene expression matrix (*.mtx)). Basic information for the scRNA-seq data, including the number of cells, genes and depth, is provided in Supplementary Table S2.

### Tissue processing, 10x Genomics sample processing and bioinformatic analysis

Detailed information can be found in the Supplemental Material.

## Results

### scRNA-seq transcriptomic profiles of the CKTR and normal groups

**Figure 1:**
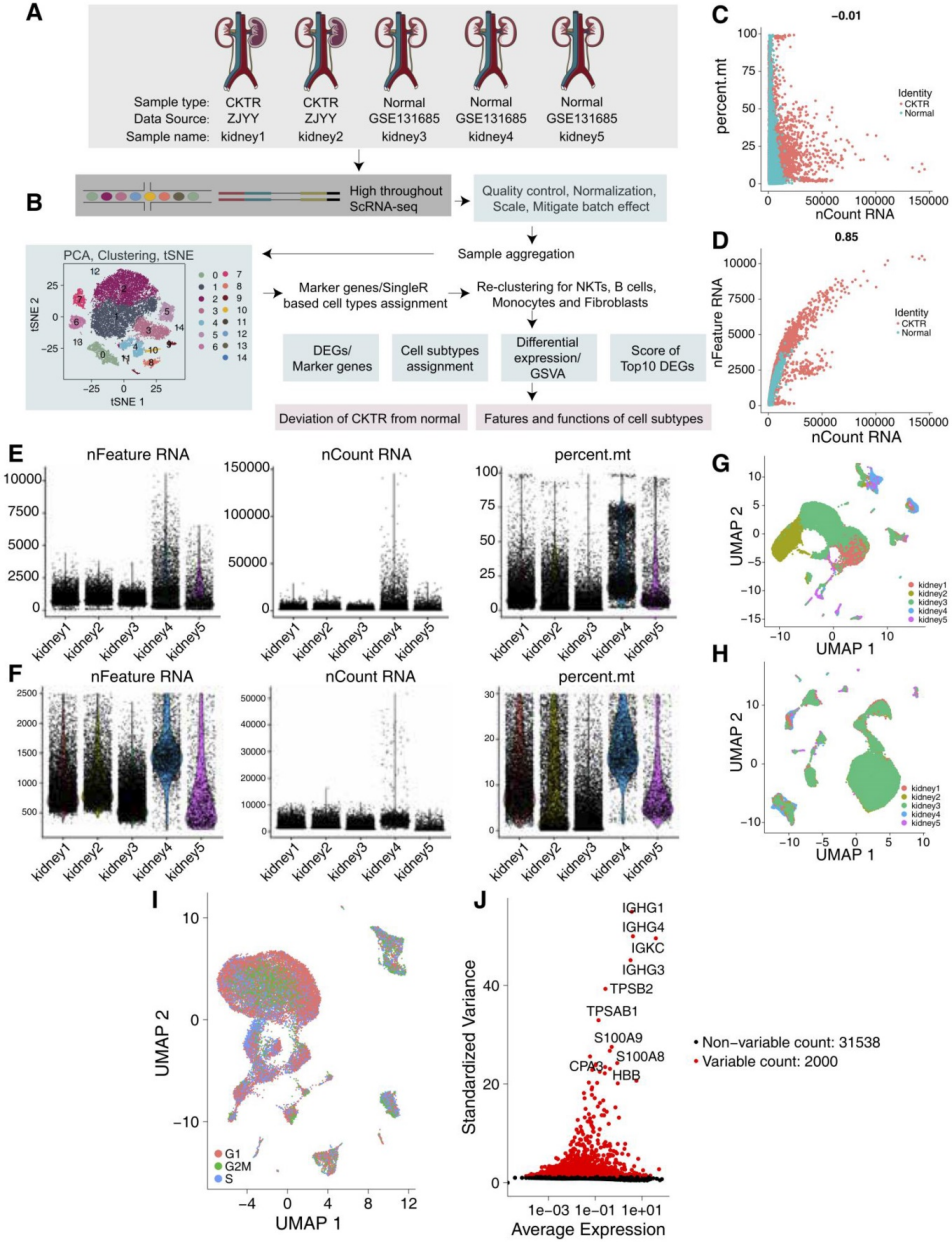
** Quality control (QC) of single cells from healthy adult human kidney and kidney allograft biopsy samples. (A)** Overview of the scRNA-seq process using healthy adult human kidney and kidney allograft biopsy samples. **(B)** Summary of the sample origins. GEO: Gene Expression Omnibus **(C)** The relationship between the percentage of mitochondrial genes and the mRNA reads. **(D)** The relationship between the amount of mRNA and the mRNA reads. **(E)** Before QC, scatterplot illustrating the number of genes, unique molecular identifiers (UMIs) and percentage of mitochondrial genes in each cell type from the five kidney samples. **(F)** After QC, scatterplot illustrating the number of genes, UMIs and percentage of mitochondrial genes in each cell type from the five kidney samples. **(G)** We detected the batch effect between five different kidney samples. **(H)** We used the Harmony R package to remove the batch effect between five different kidney samples. **(I)** UMAP plot showing the cell cycle status of each cell. **(J)** Red point illustrating the top 2000 highly variable genes.

We collected scRNA-Seq data from three healthy adult kidneys from a public database 6 and two CKTR biopsy specimens from Zhujiang Hospital of Southern Medical University (Figure 1A-B). The number of UMIs (Figure 1C) was not significantly correlated with the percentage of mitochondrial genes but was positively correlated with the amount of mRNA (Figure 1D). We carried out QC analyses (Figure 1E-F) on the basis of the amount of mRNA, the mRNA reads and the percentage of mitochondrial genes. As the five kidney data sets were not well integrated to be represented as a distinct batch, we used the R package Harmony to correct the batch (Figure 1G-H). We classified the cells according to the maximum average expression in the G1/S (43 genes) and G2/M (54 genes) phases and colored the cells. In each region, three periods of cells were randomly distributed, and there was no difference in the cell cycle status (Figure 1I). After QC, highly variable genes (the top 2000) were identified and used in the downstream analysis (Figure 1J).

**Figure 2:**
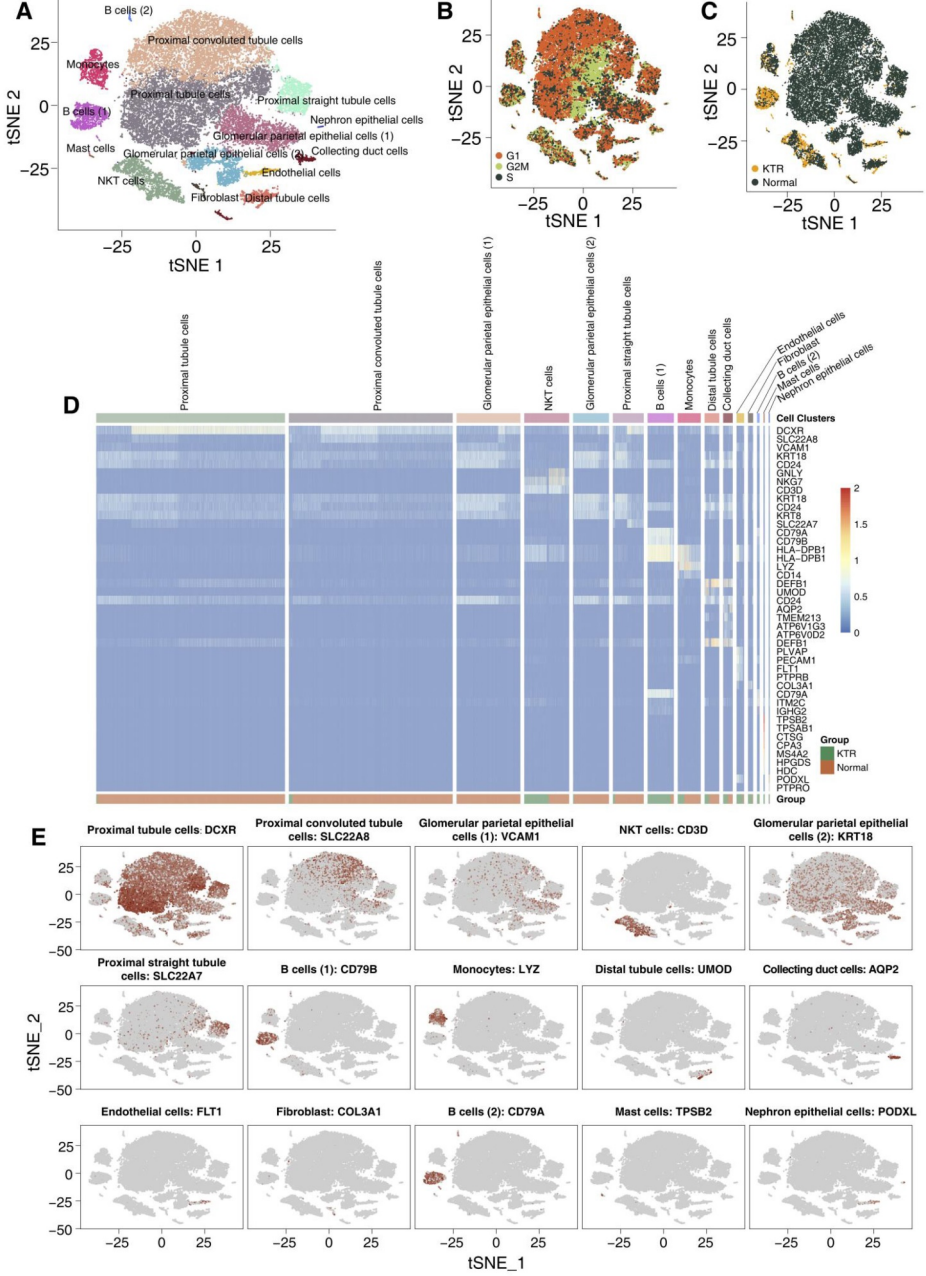
** Overview of the 27,197 single cells from healthy adult human kidney and kidney allograft biopsy samples.** tSNE of the 27,197 cell profiles, with each cell color coded for the associated cell type **(A)**, the cell cycle status **(B)** and its sample origin (**C**; normal or CKTR). Normal: healthy adult human kidney; CKTR: kidney allograft biopsy. **(D)** Heatmap showing the marker genes of each cluster, highlighting the selected marker genes for each cluster. **(E)** Expression of marker genes for the cell types are defined above each panel. Additional marker genes for each cell type are shown in Figure S1.

We used tSNE to visualize 15 clusters (Figure 2A), and cell type annotation was performed based on the specifically high gene expression of each cluster reported in the CellMarker database (Table S3). We have defined clusters 0-15 as follows: proximal tubule cells, proximal convoluted tubule cells, glomerular parietal epithelial cells (1), NKT cells, glomerular parietal epithelial cells (2), proximal straight tubule cells, B cells (1), monocytes, distal tubule cells, collecting duct cells, endothelial cells, fibroblasts, B cells (2), mast cells and nephron epithelial cells, and no bias included by the cell cycle status was identified. Most of the tubular and epithelial cells were from the healthy adult kidney samples (Figure 2B-C). We used a heatmap to visualize the marker genes related to each cluster, which is shown in Figure 2D. Additionally, the distribution of the marker genes in all cells in each cluster also confirms the representativeness of our cell assignments (Figure 2E). A complete list of DEGs between each cluster is shown in Table S4.

### mRNA expression portraits differences among the NKT cell subclasses

**Figure 3:**
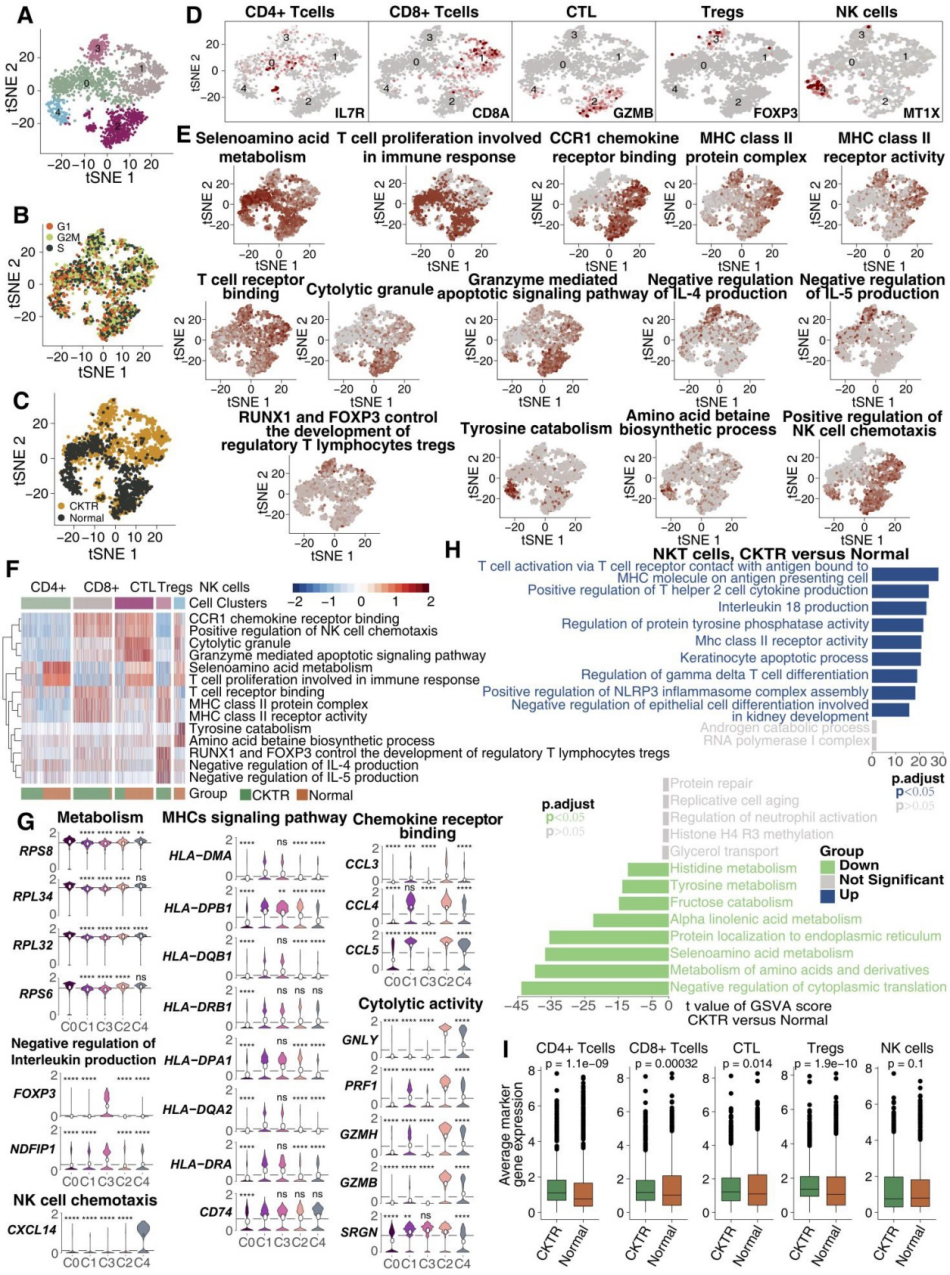
** NKT cell clusters.** tSNE plot of 1973 NKT cells, color-coded by their associated cluster **(A)**, the cell cycle status **(B)** and its sample origin (**C**; normal or CKTR). Normal: healthy adult human kidney; CKTR: kidney allograft biopsy. **(D)** tSNE plot color-coded for expression (gray to white to red) of marker genes for CD4+ T cells, CD8+ T cells, CTLs, Tregs and NK cells. CTL: Cytotoxic T lymphocyte. **(E)** tSNE plot color-coded for the ssGSEA score for CD4+ T cells, CD8+ T cells, CTLs, Tregs and NK cells. ssGSEA: single-sample gene set enrichment. **(F)** Heatmap of the ssGSEA score, as estimated using gene sets from MsigDB, for five NKT cell clusters from CD4+ T cells, CD8+ T cells, CTLs, Tregs and NK cells. **(G)** Violin plots showing the expression distribution of selected genes involved in metabolism, MHC-related, chemokine/cytolytic activity, negative regulation of interleukin production and NK cell chemotaxis, stratified by NKT cell subpopulation. **(H)** Differences in pathway activities were scored per cell by GSVA between CKTR and normal group NKT cells (n = 899 and 1074 cells from 5 samples, respectively). Shown are t values from a linear model, corrected for patient of origin. **(I)** Average expression of marker genes for each NKT subclass in normal and CKTR samples. The box plot center, box and whiskers correspond to the median, IQR and 1.5 × IQR, respectively. Data were analyzed using the Mann-Whitney U test.

We detected 1973 NKT cells. Reclustering these 1973 NKT cells revealed five clusters (Figure 3A), and these five clusters were evenly distributed in cell cycle status (Figure 3B). Three were mostly from CKTR samples (clusters 0, 1 and 3), and the two others were mostly from normal samples (clusters 2 and 4; Figure 3C). Marker genes for each subpopulation were used to assign corresponding cells to known NKT cell types (Figure 3D; Figure S1; Table S5). This analysis revealed CD4+ T cells (cluster 0; marker genes LTB and IL7R), CD8+ T cells (cluster 1; marker genes CD8A and CD8B), CTLs (cluster 2; marker genes GNLY and GZMB), Tregs (cluster 3; marker genes FOXP3 and CTLA4) and natural killer (NK) cells (cluster 4; marker genes MT1X and MT2A).

Analysis of signaling signatures highlighted that (Figure 3E-G) CD8+ T cells (cluster 1) and CTLs (cluster 2) were involved in antigen processing and presentation (APP), cytotoxicity (granzyme) or chemokine secretion, while the other three subpopulations (clusters 0, 3 and 4) showed relatively stationary or inhibitory functions. For instance, the negative regulation of the interleukin production signature in Tregs (cluster 3) was much higher than that in the other four subtypes. A more detailed analysis revealed the upregulation of genes involved in cytotoxic activities (GNLY, PRF1, GZMH and GZMB) and chemotaxis (CCL4 and CCL5) in CTLs (Figure 3G). Tregs and CTLA4 were upregulated in Tregs, while INFG was downregulated (Figure 3G).

A direct comparison of NKT cells in the CKTR and normal groups revealed T-cell activation and antigen presentation as the top enriched signature in CKTR NKT cells (Figure 3H). Additionally, the average expression of marker genes in each of the five clusters was enriched in CKTR compared with the normal group (Figure 3I). We performed an analysis of the disease samples alone (Figure S2A-F). The similarities and differences in marker gene expression across matched clusters as expected.

### B lymphocytes harbor two distinct types of memory B cells

**Figure 4:**
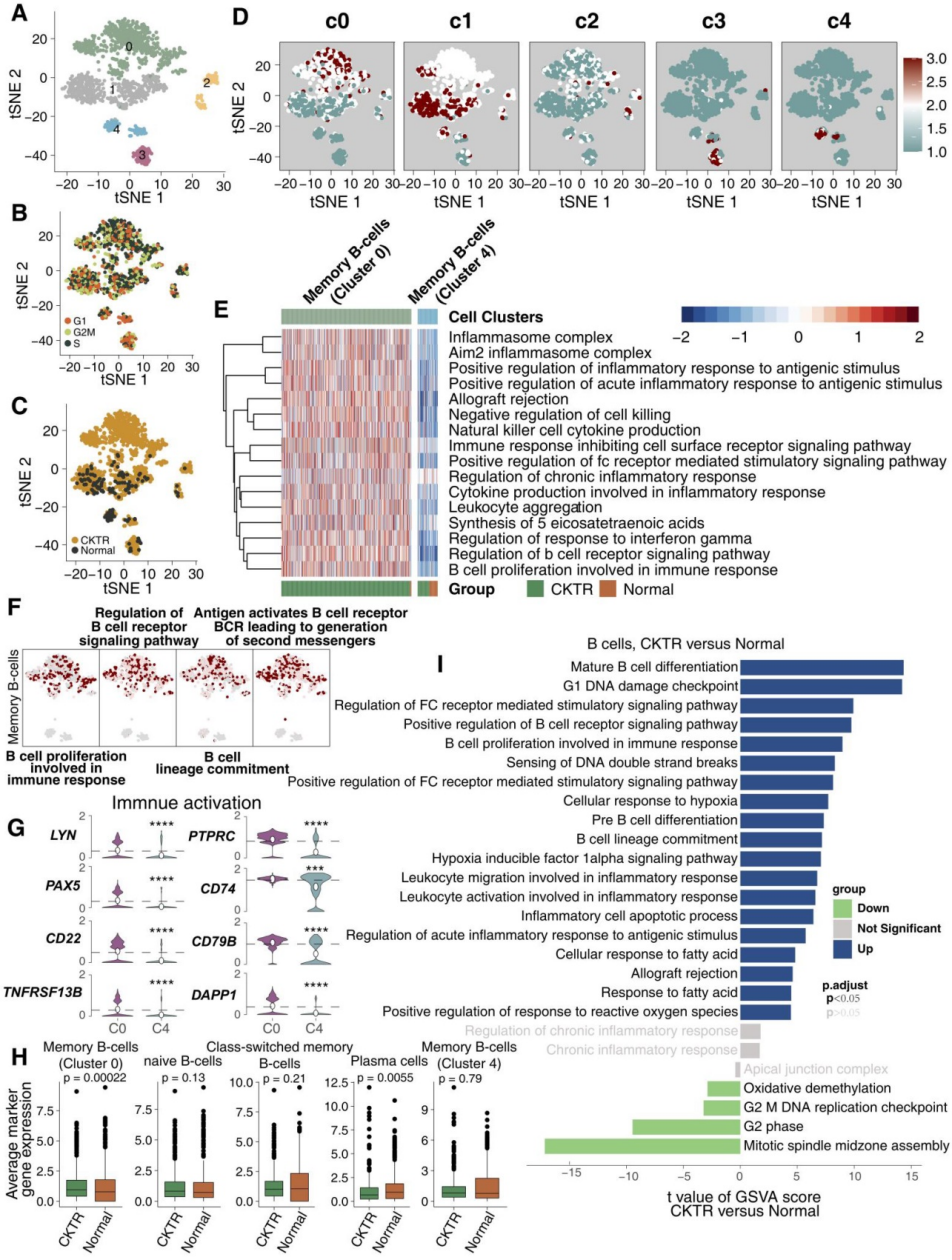
** B cell subclasses in healthy adult human kidney and kidney allograft biopsy samples.** tSNE plot of 1267 B cells, color-coded by their associated cluster **(A)**, the cell cycle status **(B)**, and its sample origin (**C**; normal or CKTR) and the score of the top 10 marker genes **(D)**. Normal: healthy adult human kidney; CKTR: kidney allograft biopsy. **(E)** Heatmap of the ssGSEA score, as estimated using gene sets from MsigDB, for two B cell clusters for memory B cells from cluster 0 and memory B cells from cluster 4. **(F)** tSNE plot color-coded for ssGSEA score of representative pathways for the clusters (clusters 0 and 4) are indicated. **(G)** Violin plots showing the expression distribution of selected genes involved in immune activation, stratified memory B cells (cluster 0) and memory B cells (cluster 4). **(H)** Average expression of marker genes for each B cell subclass in normal and CKTR samples. The box plot center, box and whiskers correspond to the median, IQR and 1.5 × IQR, respectively. Data were analyzed using the Mann-Whitney U test. **(I)** Differences in pathway activities scored per cell by GSVA between CKTR and normal group B cells (n = 1121 and 146 cells from 5 samples, respectively). Shown are t values from a linear model, corrected for the patient origin.

We detected 1267 B lymphocyte cells. B cell infiltration has been suggested to be involved in allograft rejection. Clustering revealed five subclasses (clusters 0-4; Figure 4A; Table S5). Of these, cell type assignments were performed based on the R package 'SingleR'. The tSNE plot showed a rather good distribution of B cells, suggesting that there was no bias in the cell cycle status (Figure 4B). Figure 4C shows that B cell subtypes represent the most CKTR-enriched subtypes, especially memory B cells (cluster 0). DEG analyses failed to identify marker genes of each cell type, and we adapted an algorithm (reported by Mariathasan et al) to calculate the score of the top 10 DEG sets, which validated the robustness of our clustering (Figure 4D). Surprisingly, we identified a distinct signaling signature between memory B cells in cluster 0 and memory B cells in cluster 4, revealing immune activation-associated pathways (inflammation, proinflammatory cytokine and B cell proliferation) upregulated in cluster 0 (Figure 4E). Additionally, memory B cells (cluster 0) enriched a repertoire of B cell proliferation and lineage commitment genes, while these signaling signatures showed weak enrichment in memory B cells (cluster 4; Figure 4F). In contrast to memory B cells (cluster 4), memory B cells (cluster 0) displayed high expression of genes involved in immune activation (for example, TNFRSF13B, CD79B, LYN, PAX5, CD74, PTPRC, DAPP1 and CD22; Figure 4G). Additionally, the average expression of marker genes in memory B cells (cluster 0) was enriched in the CKTR group compared with the normal group (Figure 4H). We identified differences in B cells, revealing CKTR-associated increases in mature B-cell differentiation, proliferation, and regulation of Fc receptor-mediated stimulatory signaling (Figure 4I). Additionally, Figure S3A-F indicated that B lymphocytes of the CKTR harbored two distinct types of memory B cells.

### Transcriptome profiles of classic and nonclassical monocytes

**Figure 5:**
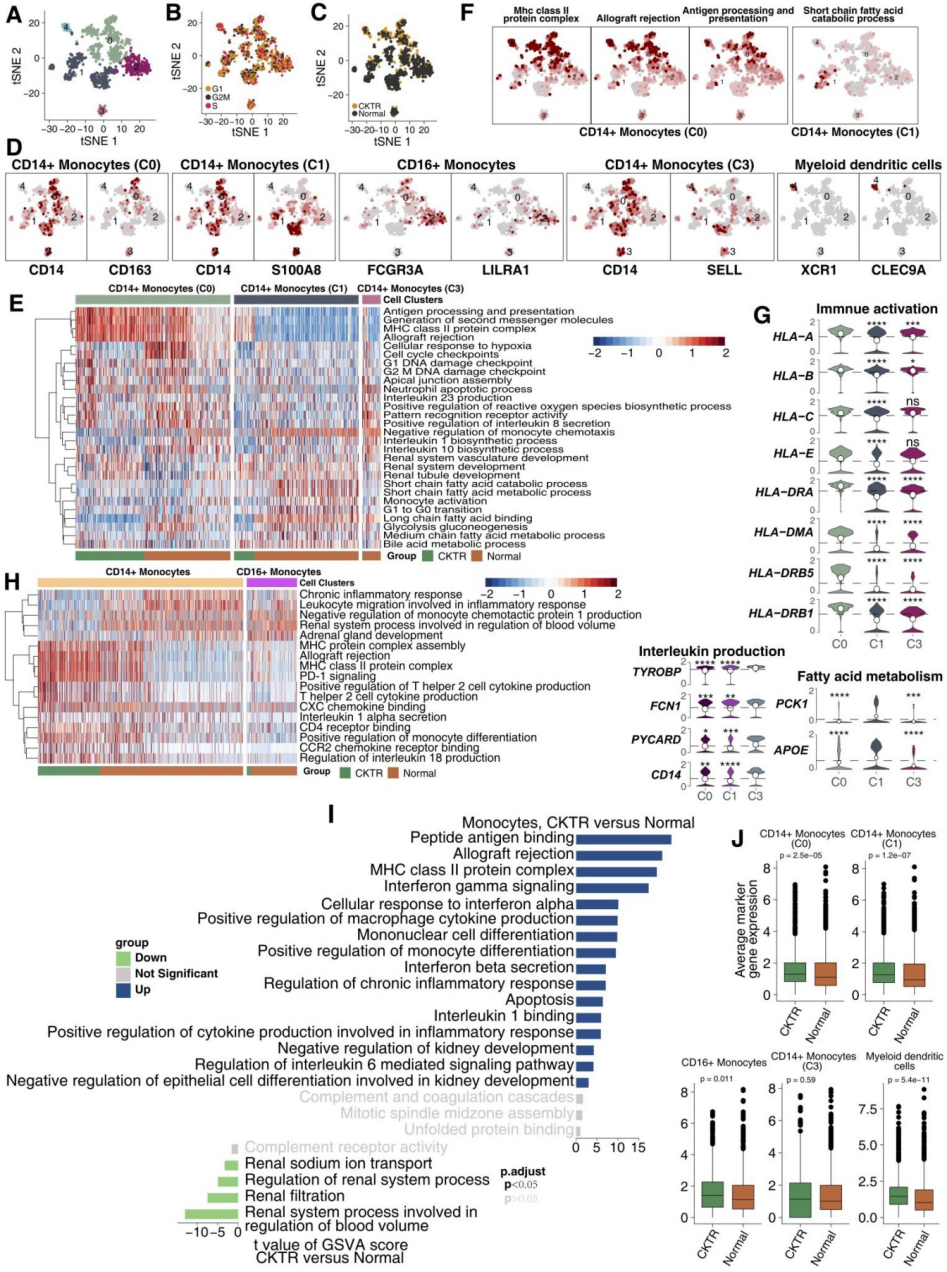
** Monocyte subpopulations in healthy kidney and kidney allograft biopsy samples.** tSNE plot of 1011 monocytes, color-coded by their associated cluster **(A)**, the cell cycle status **(B)** and its sample origin (**C**; normal or CKTR). Normal: healthy adult human kidney; CKTR: kidney allograft biopsy. **(D)** tSNE plot color-coded for expression (gray to white to red) of marker genes for CD14+ monocytes (cluster 0), CD14+ monocytes (cluster 1), CD16+ monocytes (cluster 2), CD14+ monocytes (cluster 3) and myeloid dendritic cells (cluster 4). **(E)** Heatmap of the ssGSEA score, as estimated using gene sets from MsigDB, for three CD14+ monocyte (classical type) clusters from clusters 0, 1 and 3. **(F)** tSNE plot color-coded for ssGSEA score of representative pathways for the clusters (clusters 0 and 1) as indicated. **(G)** Violin plots showing the expression distribution of selected genes involved in immune activation, fatty acid metabolism and interleukin production, stratified CD14+ monocytes (cluster 0), CD14+ monocytes (cluster 1) and CD14+ monocytes (cluster 3). **(H)** Heatmap of the ssGSEA score, as estimated using gene sets from MsigDB, for two monocyte clusters from classical (Clusters 0, 1, and 3) and nonclassical (cluster 2) monocytes. **(I)** Differences in pathway activities scored per cell by GSVA between CKTR and normal group monocytes (n = 290 and 721 cells from 5 samples, respectively). Shown are t values from a linear model, corrected for the sample origin. **(J)** Average expression of marker genes for each monocyte subclass in normal and CKTR samples. The box plot center, box and whiskers correspond to the median, IQR and 1.5 × IQR, respectively. Data were analyzed using the Mann-Whitney U test.

Monocytes are composed of several cell subtypes and play important roles in CKTR. A total of 1011 monocytes detected here were divided into five clusters, corresponding to the following:

CD14+ monocytes (cluster 0; CD14 and CD163), CD14+ monocytes (cluster 1; CD14 and S100A8), CD16+ monocytes (cluster 2; LILRA1 and CD16), CD14+ monocytes (cluster 3; SELL and CD14) and myeloid dendritic cells (cluster 4; XCR1 and CLEC9A; Figure 5A-D; Table S5). We compared pathway enrichment levels between classic and nonclassical monocytes (Figure 5E). For instance, antigen presentation and second messengers were generally high in cluster 0. Several fatty acid metabolism pathways were enriched in cluster 1. Similarly, tSNE plots show cluster 0 represents strong immune responses and monocyte activation, while cluster 1 shows high expression of fatty acids (Figure 5F). Moreover, cluster 0 expressed higher levels of major histocompatibility complex class (MHC), including HLA-A, HLA-B, HLA-C, HLA-DRA, HLA-DMA and HLA-DRB5 (Figure 5G).

Some molecules that correlated with fatty acids (PCK1 and APOE) were upregulated in cluster 1 (Figure 5G). Monocytes were derived predominantly from healthy adult kidneys. Some signaling signatures correlating to inflammation (proinflammatory cytokines, chemokines), lymphocyte recruitment and antigen presentation were enriched in classic monocytes (CD14+) compared with nonclassical monocytes (CD16+; Figure 5H). We compared signature expression levels between CKTR and normal monocytes (Figure 5I). Mostly, monocyte activation pathways, including MHC and INF-γ/β, and the inflammatory response, were significantly higher in CKTR-derived monocytes. Additionally, the average expression of marker genes in monocytes (CD14+, C0 and C1) and myeloid dendritic cells was enriched in the CKTR group compared with the normal group (Figure 5J). Also, CKTR-derived monocytes are composed of several cell subtypes and play important roles in CKTR. A total of 290 monocytes detected here were divided into five clusters (Figure S4A-G).

### Myofibroblasts (MyoFs) were enriched in the CKTR biopsies

**Figure 6:**
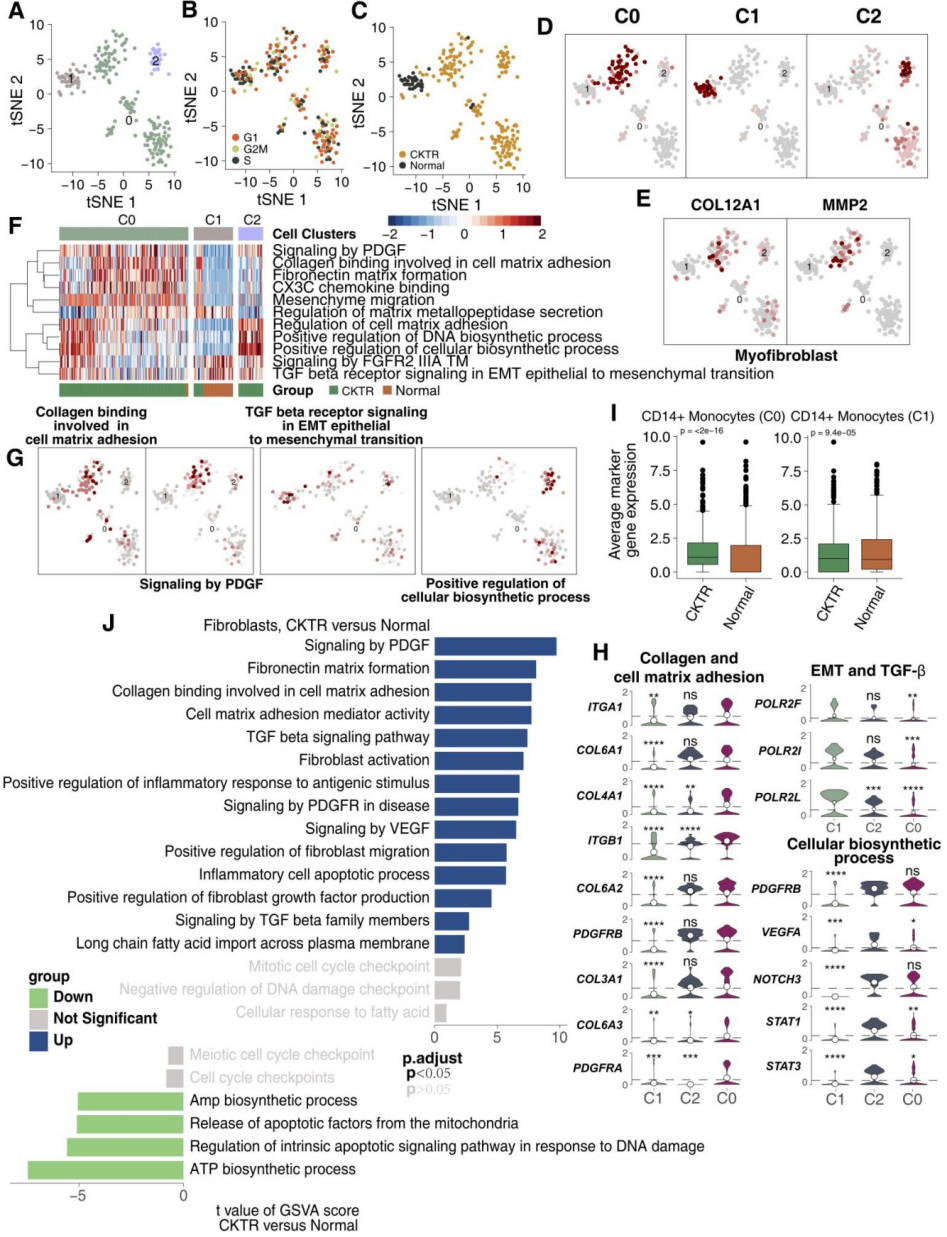
** Fibroblast clusters in healthy kidney and kidney allograft biopsy samples.** tSNE plot of 226 fibroblasts, color-coded by their associated cluster **(A)**, the cell cycle status **(B)**, its sample type of origin (**C**: Normal or CKTR) and the score of top 10 marker genes **(D)**. Normal: healthy adult human kidney; CKTR: kidney allograft biopsy. **(E)** Heatmap of the ssGSEA score, as estimated using gene sets from MsigDB, for three fibroblast clusters from clusters 0, 1 and 3. **(F)** tSNE plot color-coded for ssGSEA score of representative pathways for the clusters (clusters 0, 1 and 3) as indicated. **(G)** Violin plots showing the expression distribution of selected genes involved in collagen and cell matrix adhesion, EMT/TGF-β, and cellular biosynthesis, stratified by monocyte subpopulation. EMT: epithelial-to-mesenchymal transition. **(H)** tSNE plot color-coded for expression (gray to red) of marker genes for myofibroblasts (MyoFs). **(I)** Average expression of marker genes for each fibroblast subclass in normal and CKTR samples. The box plot center, box and whiskers correspond to the median, IQR and 1.5 × IQR, respectively. Data were analyzed using the Mann-Whitney U test. **(J)** Differences in pathway activities scored per cell by GSVA between CKTR and normal group fibroblasts (n = 188 and 38 cells from 5 samples, respectively). Shown are t values from a linear model, corrected for the sample origin.

Fibroblasts have been considered to correlate strongly with allograft fibrosis. In our datasets, 226 fibroblasts were detected. Subcluster analysis using the R package 'SingleR' revealed three subpopulations (Figure 6A; Table S5). Major fibroblasts were derived from CKTR, and most fibroblasts showed no bias in cell cycle status (Figure 6B-C). All fibroblasts can be divided into three clusters using an algorithm (reported by Mariathasan et al) to calculate the score of the top 10 DEG sets, validating the subclustering robustness (Figure 6D). The first stroma cluster (fibroblast cluster 0) uniquely expressed COL12A1 and MMP2, two genes strongly upregulated in kidney MyoFs during fibrosis, indicating a MyoF cluster (Figure 6E) 11,15. Remarkably, the MyoF subclass (mostly derived from CKTR) represents a high signature level of collagen and extracellular matrix (ECM) molecules and included platelet-derived growth factor (PDGF) signaling, collagen binding with the cell matrix and CXC3 chemokine binding. Cluster 1 displayed high expression levels of TGF-β signaling involved in activities related to epithelial-to-mesenchymal transition (EMT) and angiogenesis, while cluster 2 showed an enrichment in cellular biosynthesis processes (Figure 6F-G). Additionally, we observed MyoFs involved collagen and ECM signaling, with MyoFs expressing collagen-related genes (COL3A1, COL6A1, COL6A2, COL6A3, and COL4A1) and PDGR-related genes (PDGFRA and PDGFRB. Cluster 1 displayed high expression of EMT-related genes (POLR2F, POLR2I and POLR2L), while cluster 2 showed a high expression of biosynthesis signatures (for example, STAT1, STAT3, NOTCH3, PDGFRB and VEGFA; Figure 6H). Additionally, the average expression of marker genes in cluster 0 and cluster 1 showed differences between the CKTR and normal groups (Figure 6I). Additionally, we did uncover signaling differences in fibroblasts, indicating CKTR-associated increases in collagen, PDGF, ECM, and TGF-β and in fibroblast activation and migration (Figure 6J). The comparison of different fibroblasts subpopulations in CKTR was detailed in Figure S5A-G.

## Discussion

Here, we present comprehensive profiles of cell types and subtypes (mainly immune cells and a stromal cell type) in healthy adult kidney and CKTR biopsy samples at a single-cell resolution. By assessing key molecular function differences cell subclasses coopted by CKTR and matching healthy adult kidney samples, our study did uncover many important results made previously in bulk and highlight critical points for further research in CKTR biology. By identifying cell subpopulations and distinct signaling signatures and by analyzing the expression levels of key molecular functions in cell subtypes, our data will help advance the diagnosis and treatment of CKTR.

First, our analysis identified 15 separate cell types, including five tubular cell types (33.3%), three epithelial populations (20.0%), five types of immune cells (33.3%), one type of stromal cells (6.7%) and endothelial cells (6.7%). Second, each subclass showed distinct pathway signatures and activities, both between CKTR and normal samples or within each other, indicating that these subtypes represent different biological and molecular entities. A third observation associated major cell subclasses enriched in immune activation activities with CKTR samples.

T and NK cells play important roles in CKTR. Donor antigen-presenting cells (APCs), including macrophages, recognize allogeneic antigens of donors and then send them to the cell surface. They are indirectly identified by T lymphocyte cells and participate in allograft rejection, forming interstitial fibrosis areas 16-18. Studies suggest that CD8+ T cells and CTLs can directly activate cell killing via cytotoxic activities (including high interferon (IFN) and granzyme expression) 19-21. In addition, type I IFN can enhance Th1 polarization of CD4+ T cells and favor B cell differentiation involved in antibody production 22.

Previous bulk RNA-seq on CKTR suggested that extensive activation of the immune system can lead to necrosis of renal tubular epithelial cells, rupture of the basement membrane, and finally progression to fibrosis and loss of renal allograft function 22. Additionally, proinflammatory transcripts were previously associated with allograft rejection 22. We therefore explored these potential functional differences between each NKT cell subtype or between CKTR and normal samples. CD8+ T cells and CTLs representing the immunoactivation signature were more abundant in the CKTR biopsy samples. A similar ssGSEA was observed in CD8+ T cells and CTLs, showing higher cytotoxic (IFN and granzyme secretion), antigen presentation and proinflammatory (cytokines and chemokine production) activities. Interestingly, NKs exhibited weak immunoactivation states, except for encoding several molecules (granzyme). Together, these observations suggest that T cell subclusters represent distinct signatures that have different roles in allograft loss 20.

B lymphocyte cell infiltration plays an important role in the CKTR 17,23. We consistently identified B cells representing the most CKTR-enriched cells: two memory B cells representing totally distinct biological functions 24-26 (activated and stationary states). For instance, activated memory B cells were mostly derived from CKTR samples and were enriched in B-cell proliferation, differentiation, antigen presentation, lymphocyte recruitment and inflammatory responses, while major stationary memory B cells were mostly derived from healthy human kidney samples and displayed a strong fatty acid metabolism and cell cycle signal.

The interaction between B lymphocyte cells and surrounding stromal cells plays key roles in proinflammatory and profibrotic activities and remains poorly characterized 17,23. The ECM is an important repertoire of molecules in CKTR. For instance, MyoFs express high levels of some collagens, TGF-β, and other ECM molecules involved in tissue remodeling 23,27,28. Our subclustering showed that one stroma cluster may be MyoFs. ssGSEA highlighted that myofibroblasts secreted (collagen and fibronectin) or expressed PDGFs, which further led to renal interstitial fibrosis 29-31 (Figure 6E). Additionally, EMT and vascular regulatory activities were also enriched in MyoFs. Although the source of myofibroblasts is not yet clear, they may originate from renal tubular epithelial cells, intrinsic fibroblasts or other cells (such as pericytes) 32. These observations suggest that specific functions of myofibroblasts and memory B cells play important roles in allograft rejection and loss.

Our analyses, however, have several limitations. First, chronic renal rejection samples are single biopsies, and therefore, our observations cannot generalize the whole dynamic development of chronic renal rejection. More recently, there is a lack of dynamic immune portraits and molecular characterization of CKTR. Clearly, additional work and research are needed to describe the dynamic immune profiles of CKTR. Second, scRNA-seq has offered opportunities to uncover novel cell types and status comprehensively without some bias and RNA degradation, while the cell viability used for this technique is normally high. Due to the high number of dead renal cells in CKTR, it is still necessary to carry out further work on how to determine the best time to biopsy. Third, three matched healthy adult kidney samples obtained from the GEO database were used as controls, and we could not perform immunohistochemistry to further verify the observations. Fourth, there is no comparison between acute and chronic renal rejection.

## Conclusions

Despite these limitations, we describe comprehensive profiles of immune cells, stromal cells and novel cell subtypes in healthy adults and renal allograft rejection samples at single-cell resolution and further compare the distinctive features in signaling pathways among each cell subpopulation. We expect that our findings will provide novel and deeper insights into human chronic renal transplantation rejection that will be helpful in the therapy of human allograft rejection.

## Supplementary Material

Supplementary figures and tables.

## Abbreviations

scRNA-seq: single-cell RNA sequencing
CKTR: chronic kidney transplant rejection
ssGSEA: single-sample gene set enrichment
NKT: natural killer T cells
CTLs: cytotoxic T lymphocytes
Tregs: regulatory T cells
NKs: natural killer cells
MyoF: myofibroblasts
AMR: antibody-mediated rejection
IRB: Institutional Review Board
PRA: panel reactive antibodies
MHC: major histocompatibility complex class
ECM: extracellular matrix
PDGF: platelet-derived growth factor
EMT: epithelial-to-mesenchymal transition
IFN: interferon
