# Supplementary material for: Single-cell analysis reveals immune landscape in kidneys of patients with chronic transplant rejection
Source: Theranostics. 2020 Jul 11;10(19):8851–62. doi: 10.7150/thno.48201 (PMC7392010; doi:10.7150/thno.48201)
Supplement: Supplementary file 1 — Supplementary figures and tables. [file thnov10p8851s1.pdf]

Supplementary Files:

Supplementary Methods

Figure S1. tSNE plots color-coded for the expression of the additional marker genes from Figure 2e.

Figure S2. NKT cell clusters of the CKTR

Figure S3. B cell subclasses in kidney allograft biopsy samples

Figure S4. Monocyte subpopulations in kidney allograft biopsy samples

Figure S5. Fibroblast clusters in kidney allograft biopsy samples

Table S1. Information on the five patients

Table S2. Characteristics of the five kidney tissue samples included in this study

Table S3 The marker genes for the fifteen cell clusters

Table S4 Differentially expressed genes (DEGs) for each cluster of human kidney cells

Table S5 Subpopulation characteristics for the four cell types

Figure S1: tSNE plots color-coded for the expression of the additional marker genes from Figure 2e.

Shown are the markers for cell clusters (related to Table S3): major histocompatibility complex (MHC), class II, DP beta 1 (HLA-DPB1); integral membrane protein 2C (ITM2C); immunoglobulin heavy constant gamma 2 (IGHG2); collecting duct cells: transmembrane protein 213 (TMEM213); ATPase H+ transporting V1 subunit G3 (ATP6V1G3); ATPase H+ transporting V0 subunit d2 (ATP6V0D2); distal tubule cells: defensin beta 1 (DEFB1); endothelial cell: plasmalemma vesicle associated protein (PLVAP); platelet and endothelial cell adhesion molecule 1 (PECAM1); protein tyrosine phosphatase receptor type B (PTPRB); glomerular parietal epithelial cells: CD24 molecule (CD24); keratin 8 (KRT8); mast cell: tryptase alpha/beta 1 (TPSAB1); cathepsin G (CTSG); carboxypeptidase A3 (CPA3); membrane spanning 4-domains A2 (MS4A2); hematopoietic prostaglandin D synthase (HPGDS); histidine decarboxylase (HDC); monocytes: CD14 molecule (CD14); nephron epithelial cells: protein tyrosine phosphatase receptor type O (PTPRO); and NKT cells: granulysin (GNLY); natural killer cell granule protein 7 (NKG7).

Figure S2. NKT cell clusters of the CKTR

Figure S2-Figure supplement 1. tSNE plot of 1074 NKT cells (from the CKTR), color-coded by their associated cluster (A) and the cell cycle status (B). CKTR: kidney allograft biopsy. (C) tSNE plot color-coded for expression (gray to white to red) of marker genes for CD4+ T cells, CD8+ T cells, CTLs, Tregs and NK cells. CTL: Cytotoxic T lymphocyte. (D) tSNE plot color-coded for the ssGSEA score for CD4+ T cells, CD8+ T cells, CTLs, Tregs and NK cells. ssGSEA: single-sample gene set enrichment. (E) Heatmap of the ssGSEA score, as estimated using gene sets from MsigDB, for five NKT cell clusters from CD4+ T cells, CD8+ T cells, CTLs, Tregs and NK cells. MsigDB: Molecular Signatures Database; (F) Violin plots showing the expression distribution of selected genes involved in metabolism, MHC-related, chemokine/cytolytic activity, negative

regulation of interleukin production and NK cell chemotaxis, stratified by NKT cell subpopulation.

Figure S3. B cell subclasses in kidney allograft biopsy samples

Figure S3-Figure supplement 1. tSNE plot of 1121 B cells (from the CKTR), color-coded by their associated cluster (A) and the cell cycle status (B) and the score of the top 10 marker genes (C). CKTR: kidney allograft biopsy. (D) Heatmap of the ssGSEA score, as estimated using gene sets from MsigDB, for two B cell clusters for memory B cells from cluster 0 and memory B cells from cluster 4. MsigDB: Molecular Signatures Database; (E) tSNE plot color-coded for ssGSEA score of representative pathways for the clusters (clusters 0 and 4) are indicated. (F) Violin plots showing the expression distribution of selected genes involved in immune activation, stratified memory B cells (cluster 0) and memory B cells (cluster 4)

Figure S4. Monocyte subpopulations in kidney allograft biopsy samples

Figure S4-Figure supplement 1. tSNE plot of 290 monocytes, color-coded by their associated cluster (A) and the cell cycle status (B). CKTR: kidney allograft biopsy. (C). tSNE plot color-coded for expression (gray to white to red) of marker genes for CD14+ monocytes (cluster 0), CD14+ monocytes (cluster 1), CD16+ monocytes (cluster 2), CD14+ monocytes (cluster 3) and myeloid dendritic cells (cluster 4). (D) Heatmap of the ssGSEA score, as estimated using gene sets from MsigDB, for three CD14+ monocyte (classical type) clusters from clusters 0, 1 and 3. (E) tSNE plot color-coded for ssGSEA score of representative pathways for the clusters (clusters 0 and 1) as indicated. (F) Violin plots showing the expression distribution of selected genes involved in immune activation, fatty acid metabolism and interleukin production, stratified CD14+ monocytes (cluster 0), CD14+ monocytes (cluster 1) and CD14+ monocytes (cluster 3). (G) Heatmap of the ssGSEA score, as estimated using gene sets from MsigDB, for two monocyte clusters from

classical (Clusters 0, 1, and 3) and nonclassical (cluster 2) monocytes. MsigDB: Molecular Signatures Database;

Figure S5. Fibroblast clusters in kidney allograft biopsy samples

Figure S5-Figure supplement 1. tSNE plot of 226 fibroblasts, color-coded by their associated cluster (A), the cell cycle status (B), and the score of top 10 marker genes (C). CKTR: kidney allograft biopsy. (D) tSNE plot color-coded for expression (gray to red) of marker genes for myofibroblasts (MyoFs). (E) Heatmap of the ssGSEA score, as estimated using gene sets from MsigDB, for three fibroblast clusters from clusters 0, 1 and 3. MsigDB: Molecular Signatures Database; (F) tSNE plot color-coded for ssGSEA score of representative pathways for the clusters (clusters 0, 1 and 2) as indicated. (G) Violin plots showing the expression distribution of selected genes involved in collagen and cell matrix adhesion, EMT/TGF-β, and cellular biosynthesis, stratified by monocyte subpopulation. EMT: epithelial-to-mesenchymal transition

Supplementary Methods

Chronic kidney transplantation rejection tissue processing and dissociation

Two fresh chronic kidney transplantation rejection samples (Supplementary Table S1) were collected at the Zhujiang Hospital of Southern Medical University. First, fresh samples were taken from the operating room, placed in a solution containing Hank's balanced salt solution (HBSS; 311-512-CL) and 1% antibiotic-antimycotic (Gibco, 15240062) on ice, and transported to the laboratory within 20 min. Then, 0.5-1 g full-thickness sections of kidney tissue were cut lengthwise using surgical scissors. Subsequently, the tissue was washed twice with cold Dulbecco's phosphate-buffered saline (DPBS; WISENT, 311-425-CL). The tissue was placed on a stainless-steel cell filter, crushed with the plunger of a syringe and washed with DPBS. Flushing fluid was added to a centrifuge tube, and the kidney fragments were collected and placed in the tube, which was then spun at 350 × g for 5 min at 4 °C; this step was then repeated. After discarding the supernatant, TrypLETM Express Enzyme (1X, Gibco, 12605010) was used to further digest the sticky clumps of cells for 5–10 min at 37 °C, and then the digestion was terminated using Dulbecco's modified Eagle's medium (DMEM; WISENT, 319-006-CL) containing 10% fetal bovine serum (FBS; Gibco, 10099141). The digested cells were centrifuged at 350 × g for 5 min at 4 °C. After discarding the supernatant, the cells were resuspended in 5 ml of DPBS and filtered through a 100 μm cell strainer. Next, red blood cells (RBCs) were removed using 1X RBC lysis buffer (10X diluted to 1X, BioLegend, B250015) for 5 min, and the cells were centrifuged at 300 × g for 5 min at 4 °C. After discarding the supernatant, the cells were suspended in DPBS and centrifuged again. The supernatant was again discarded, and the cells were resuspended in cold DPBS and passed through a 40 μm cell strainer. Live cells were counted using trypan blue staining (0.4%, Gibco, 420301). If the cell viability was above 90%, we performed 10x Genomics sample processing.

10x Genomics sample processing and cDNA library preparation.

To prepare the single-cell suspension, the 10x Genomics Chromium Single Cell 3′ Reagent Kit v3 user guide (https://support.10xgenomics.com/single-cell-gene-expression/index/doc/user-guide-chromium-single-cell-3-reagent-kits-user-guide-v31-chemistry) was followed. Single-cell samples were passed through a 40 μm cell strainer and counted using a hemocytometer with trypan blue. Then, the appropriate volume of each sample was diluted to recover 10,000 kidney cells. Subsequently, the single-cell suspension, gel beads and oils were added to the 10x Genomics single-cell A chip. We checked to ensure that there were no errors before running the assay. After droplet generation, samples were transferred into PCR tubes, and we performed reverse transcription using a thermal cycler (Bio-Rad). After reverse transcription, cDNA was recovered using a recovery agent provided by 10x Genomics, followed by silane DynaBead clean-up as outlined in the user guide. Before clean-up using SPRIselect beads, we amplified the cDNA for 10 cycles. The cDNA concentration was detected by a Qubit 4.0 fluorometer (Invitrogen). The kidney cDNA libraries were prepared according to the Chromium Single Cell 3′ Reagent Kit v3.1 user guide.

Single-cell RNA-seq details and preliminary results

Samples were sequenced by Hiseq Xten (Illumina, San Diego, CA, USA) with the following run parameters: read 1 for 150 cycles, read 2 for 150 cycles, and index for 14 cycles. Preliminary sequencing results (bcl files) were converted to FASTQ files with CellRanger (version 3.0, https://support.10xgenomics.com/single-cell-gene-expression/software/pipelines/latest/what-is-cell-ranger). We followed the 10x Genomics standard seq protocol by trimming the barcode and unique molecular identifier (UMI) end to 26 bp and the mRNA end to 98 bp. Then, the FASTQ files were aligned to the human genome reference sequence GRCh38. Subsequently, we applied CellRanger for preliminary data analysis and generated a file that contained a barcode table, a gene table and a

gene expression matrix. We carried out preliminary quality control (QC) on the FASTQ files to ensure high-quality scRNA-seq data. At the same time, using the website, we obtained some basic information, including the number of cells, the median number of detected genes, sequencing saturation and sequencing depth (Supplementary Table 2). The strategy of using CellRanger V3.0 and trimming the FASTQ data to 26 bp × 98 bp was used to preprocess the scRNA-seq data and perform downstream analysis.

Using Seurat R for quality control (QC) and data second analysis after mitigating the batch effect

We used R (version 3.6.1, https://www.r-project.org/) and the Seurat[1,2] R package (version 3.6.1, https://satijalab.org/seurat/) for the analyses. We used the MergeSeurat function to merge the five kidney data sets (three GEO/healthy adult kidney samples; two chronic kidney rejection samples). The filter criteria of cells were determined after referencing previous studies[3,4]. According to the median number of genes and the percentage of mitochondrial genes in the kidney samples (Figure 1e), cells with <200 and >2,500 genes (potential cell duplets) and a mitochondrial gene percentage of >30% were filtered. After QC, 27197 high-quality kidney cells were obtained. The relationship between the percentage of mitochondrial genes and the mRNA reads was detected and visualized as the relationship between the number of mRNAs and the reads of mRNA (Figure 1c-d). After data normalization, all highly variable genes in single cells were identified after controlling for the relationship between the average expression and dispersion.

Since these data came from five different samples, to avoid the batch effect affecting the downstream analysis, we adopted a strategy called ‘Harmony’ to mitigate the batch effect. The R package Harmony focuses on the scalable integration of scRNA-seq data for batch correction and meta-analysis[5] (version 1,0, https://github. Com/immunogenomics/Harmony). Subsequently, we used principal component analysis (PCA) with variable genes as the input and identified significant principal

components (PCs) based on the jackStraw function. Twenty PCs were selected as the input for uniform manifold approximation and projection (UMAP) and t-distributed stochastic neighbor embedding (tSNE) when statistically significant. We detected the batch effect between five different kidney samples (Figure 1g-h). With a resolution of 0.25, cells were clustered by the FindClusters function and classified into 15 different cell types (clusters 0-14). Next, we used the FindAllMarkers function to find differentially expressed genes (DEGs) between each type of cell (Supplementary Table S4).

Cell cycle analysis

Cell cycle analysis was performed by using the Seurat program (CellCycleScoring function). We used a previously defined core set of 43 G1/S and 54 G2/M cell cycle genes[6]. Cells were classified by the maximal average expression (‘cycle score’) in these two gene sets. When the cycle scores of G1/S and G2/M were both less than 2, we considered these cells to be noncycling. Otherwise, we considered the cells to be proliferative. After the cell cycle analysis, no bias induced by cell cycle genes was observed (Figure 1i).

Cell type markers and subclustering analysis

Cell type assignment was performed based on the marker genes reported in previous studies[7], the CellMaker database[8] and the R package ‘SingleR’[9]. Subclustering analysis of NKT cells, monocytes, fibroblasts, and B cells was performed by using the SubsetData function in the Seurat program. We used the R package ‘Harmony’[5] to mitigate the batch effect. After data normalization and scaling, all highly variable genes in single cells were identified after controlling for the relationship. Subsequently, twenty PCs were selected as the input for tSNE when statistically significant. The FindClusters function was used to classify cells into different clusters. Next, the FindAllMarkers function was used to find DEGs between each subtype. We adapted

the method reported by Mariathasan et al to calculate the score of the top 10 DEGs, which validated the clustering robustness. Next, to assign pathway activity estimates to individual cells, the R package GSVA[10] with the standard settings was implemented in ssGSEA, and a gene set containing all human pathways was downloaded from the MsigDB database[11] (KEGG, GO and Reactome). We identified significantly enriched pathways defined by the Benjamini–Hochberg-corrected P value < 0.05. To assess differential activities of pathways (GSVA) between sets of cells (for example, derived from chronic kidney rejection, normal samples or belonging to different subclusters), we contrasted the activity scores for each cell using a generalized linear model. To avoid inflating signals because of interindividual differences (for example, in the relative frequencies of cells from different patients), we always included the patient of origin as a categorical variable. The results of these linear models were visualized using bar plots or heatmaps. For the heatmaps, pathways or regulons that did not show significant changes (Benjamini–Hochberg-corrected P value > 0.05) in any of the sets of cells contrasted in one analysis were not visualized. For the bar plot, pathways or regulons that did not show significant changes (Benjamini–Hochberg-corrected P value > 0.05) were visualized in gray font.

Statistics

For all experiments, samples from a single patient were processed in parallel, and cells for each sample of one patient were processed for scRNA-seq (10x Genomics) at the same time but in separate lanes and vials. Box plots were generated using the R base package and default parameters. Hence, the boxes span the interquartile range (IQR; from the 25th to the 75th percentiles), with the centerline corresponding to the median. Lower whiskers represent the data minimum or the 25th percentile minus 1.5 × IQR, whichever is greater. Upper whiskers represent the data maximum or the 75th percentile plus 1.5 × IQR (lower), whichever is lower. Given the number of

data points represented in the box and violin plots, we opted not to display each data point, as this would obscure the overall distribution. Comparisons between two groups were performed using an unpaired two-tailed t-test (R package limma[12]) or a Mann-Whitney U test. All statistical analyses and presentations were performed using R.

Reference

1. Satija R, Farrell JA, Gennert D, Schier AF, Regev A. Spatial reconstruction of single-cell gene expression data. Nat Biotechnol. 2015; 33: 495–502.

2. Stuart T, Butler A, Hoffman P, Hafemeister C, Papalexi E, Mauck WM, Hao Y, Stoeckius M, Smibert P, Satija R. Comprehensive Integration of Single-Cell Data. Cell. 2019; 177: 1888-1902.e21.

3. Park J, Shrestha R, Qiu C, Kondo A, Huang S, Werth M, Li M, Barasch J, Suszták K. Single-cell transcriptomics of the mouse kidney reveals potential cellular targets of kidney disease. Science (80-). 2018; 360: 758–63.

4. Young MD, Mitchell TJ, Vieira Braga FA, Tran MGB, Stewart BJ, Ferdinand JR, Collord G, Botting RA, Popescu D-M, Loudon KW, Vento-Tormo R, Stephenson E, Cagan A, et al. Single-cell transcriptomes from human kidneys reveal the cellular identity of renal tumors. Science (80-). 2018; 361: 594–9.

5. Korsunsky I, Millard N, Fan J, Slowikowski K, Zhang F, Wei K, Baglaenko Y, Brenner M, Loh P, Raychaudhuri S. Fast, sensitive and accurate integration of single-cell data with Harmony. Nat Methods. 2019; 16: 1289–96.

6. Tirosh I, Izar B, Prakadan SM, Wadsworth MH, Treacy D, Trombetta JJ, Rotem A, Rodman C, Lian C, Murphy G, Fallahi-Sichani M, Dutton-Regester K, Lin J-R, et al. Dissecting the multicellular ecosystem of metastatic melanoma by single-cell RNA-seq. Science (80-). 2016; 352: 189–96.

7. Liao J, Yu Z, Chen Y, Bao M, Zou C, Zhang H, Liu D, Li T, Zhang Q, Li J,

Cheng J, Mo Z. Single-cell RNA sequencing of human kidney. Sci Data. England; 2020; 7: 4.

8. Zhang X, Lan Y, Xu J, Quan F, Zhao E, Deng C, Luo T, Xu L, Liao G, Yan M, Ping Y, Li F, Shi A, et al. CellMarker: a manually curated resource of cell markers in human and mouse. Nucleic Acids Res. 2019; 47: D721–8.

9. Aran D, Looney AP, Liu L, Wu E, Fong V, Hsu A, Chak S, Naikawadi RP, Wolters PJ, Abate AR, Butte AJ, Bhattacharya M. Reference-based analysis of lung single-cell sequencing reveals a transitional profibrotic macrophage. Nat Immunol. 2019; 20: 163–72.

10. Hänzelmann S, Castelo R, Guinney J. GSVA: gene set variation analysis for microarray and RNA-Seq data. BMC Bioinformatics. 2013; 14: 7.

11. Liberzon A, Birger C, Thorvaldsdóttir H, Ghandi M, Mesirov JP, Tamayo P. The Molecular Signatures Database Hallmark Gene Set Collection. Cell Syst. 2015; 1: 417–25.

12. Ritchie ME, Phipson B, Wu D, Hu Y, Law CW, Shi W, Smyth GK. limma powers differential expression analyses for RNA-sequencing and microarray studies. Nucleic Acids Res. 2015; 43: e47–e47.

Supplementary Figure 1

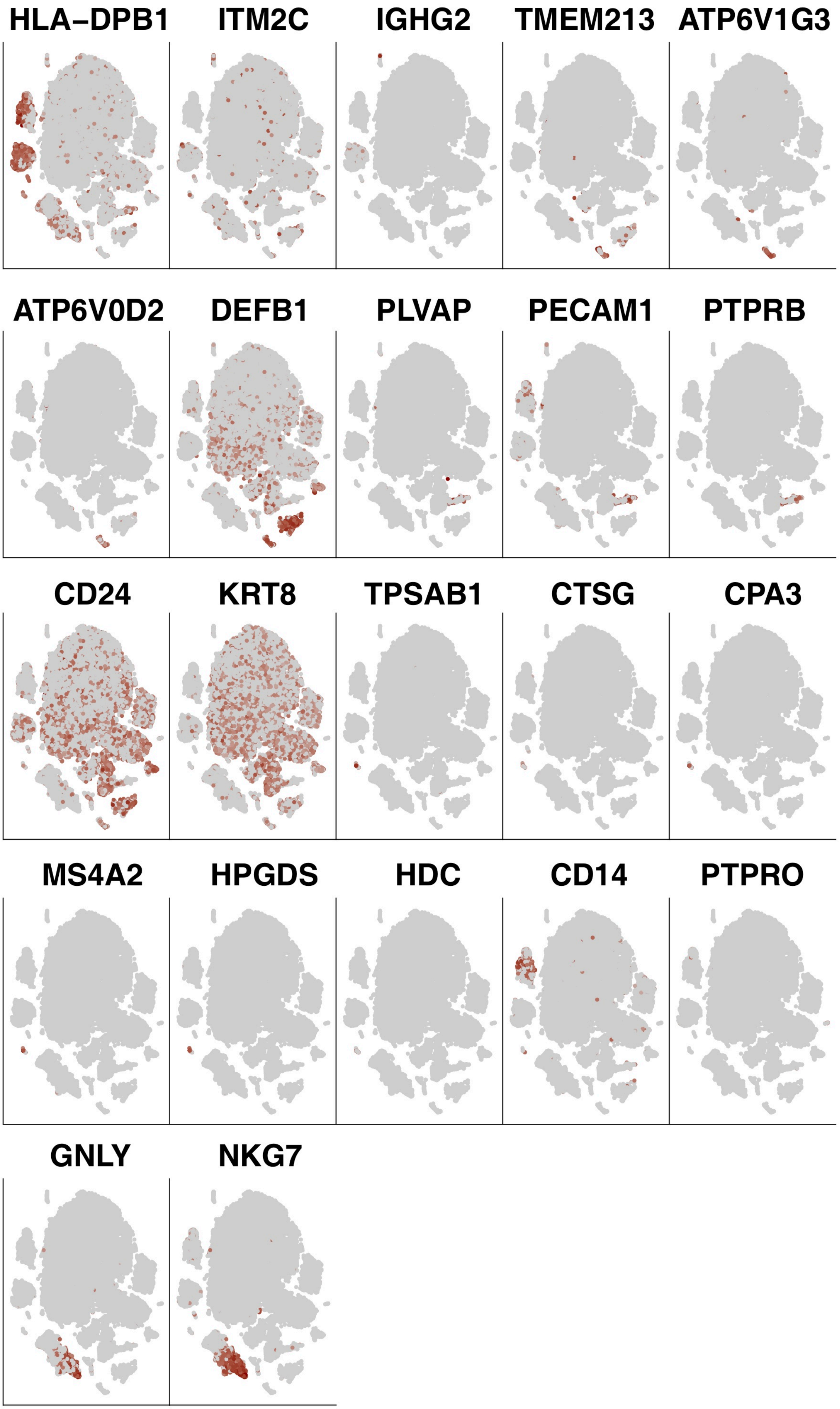

Supplementary Figure 2

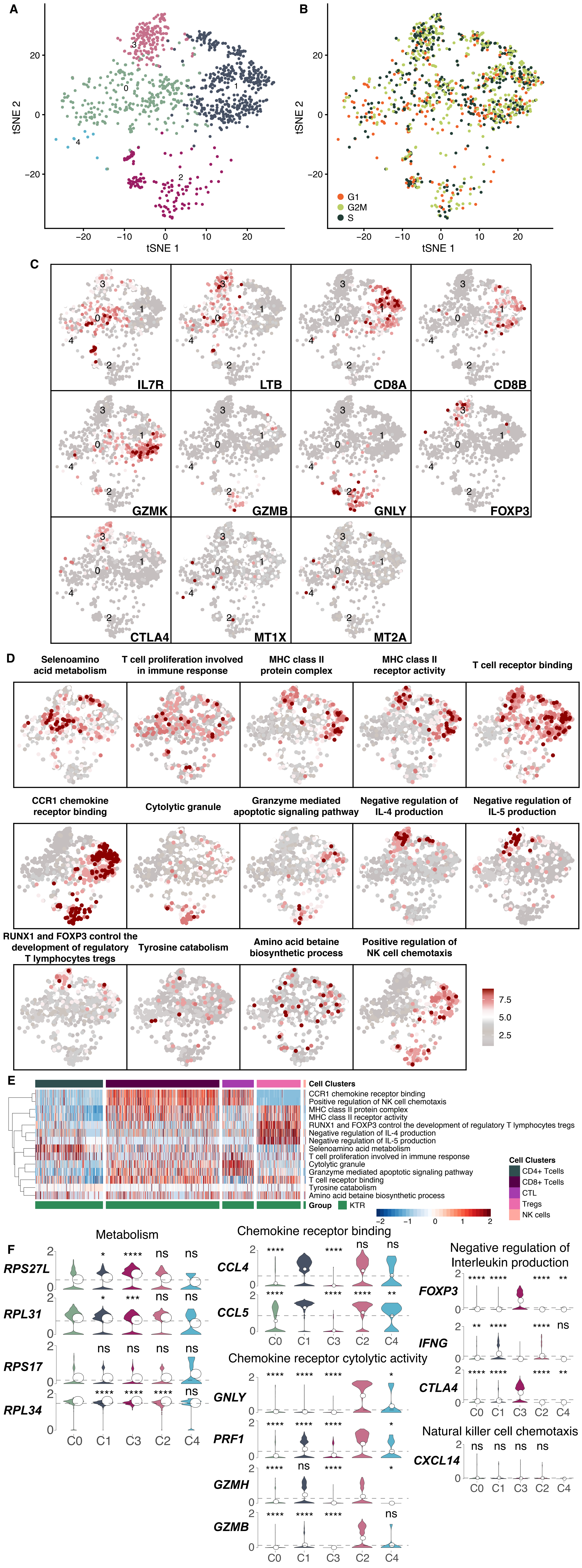

Supplementary Figure 3

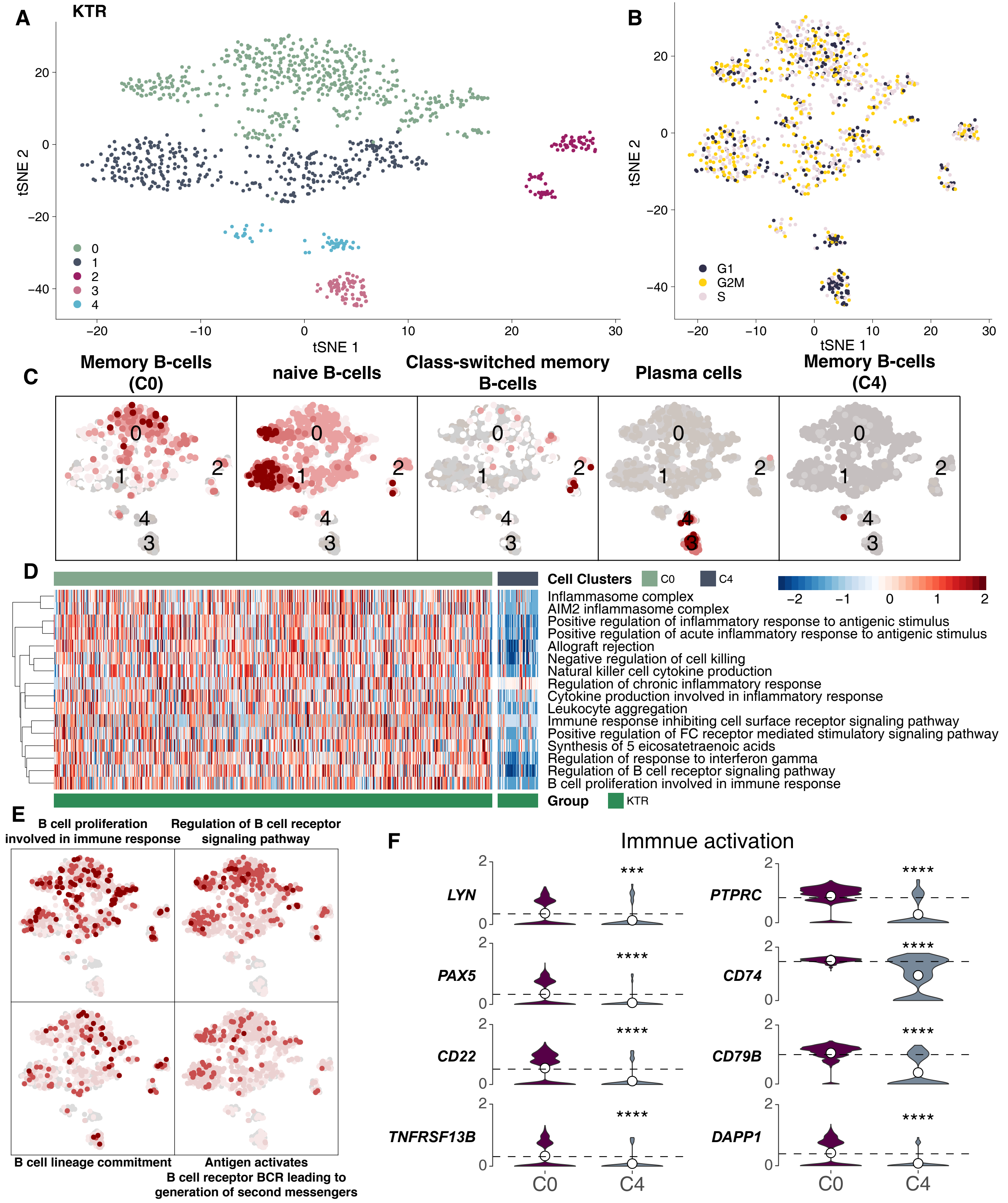

Supplementary Figure 4

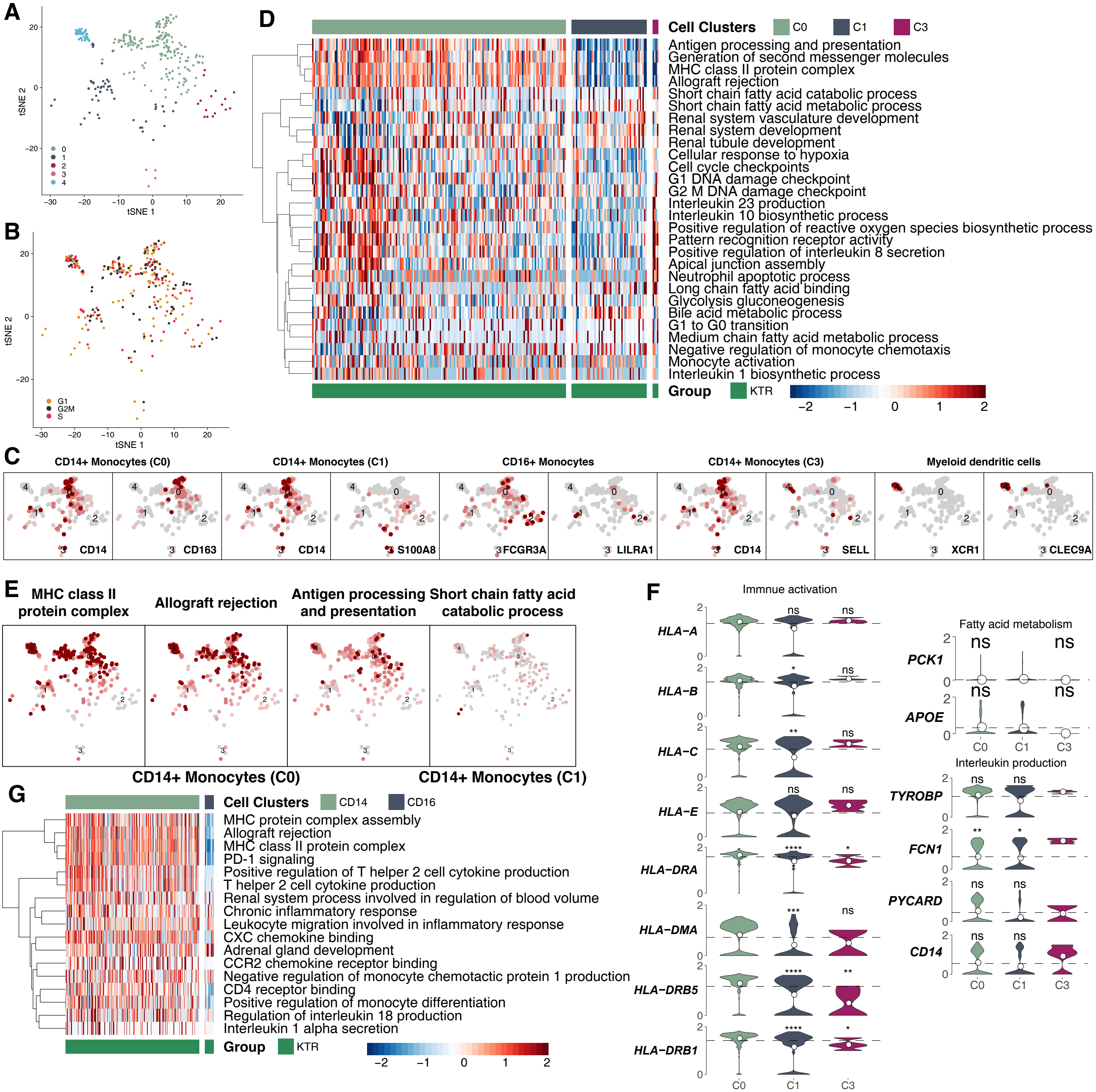

Supplementary Figure 5

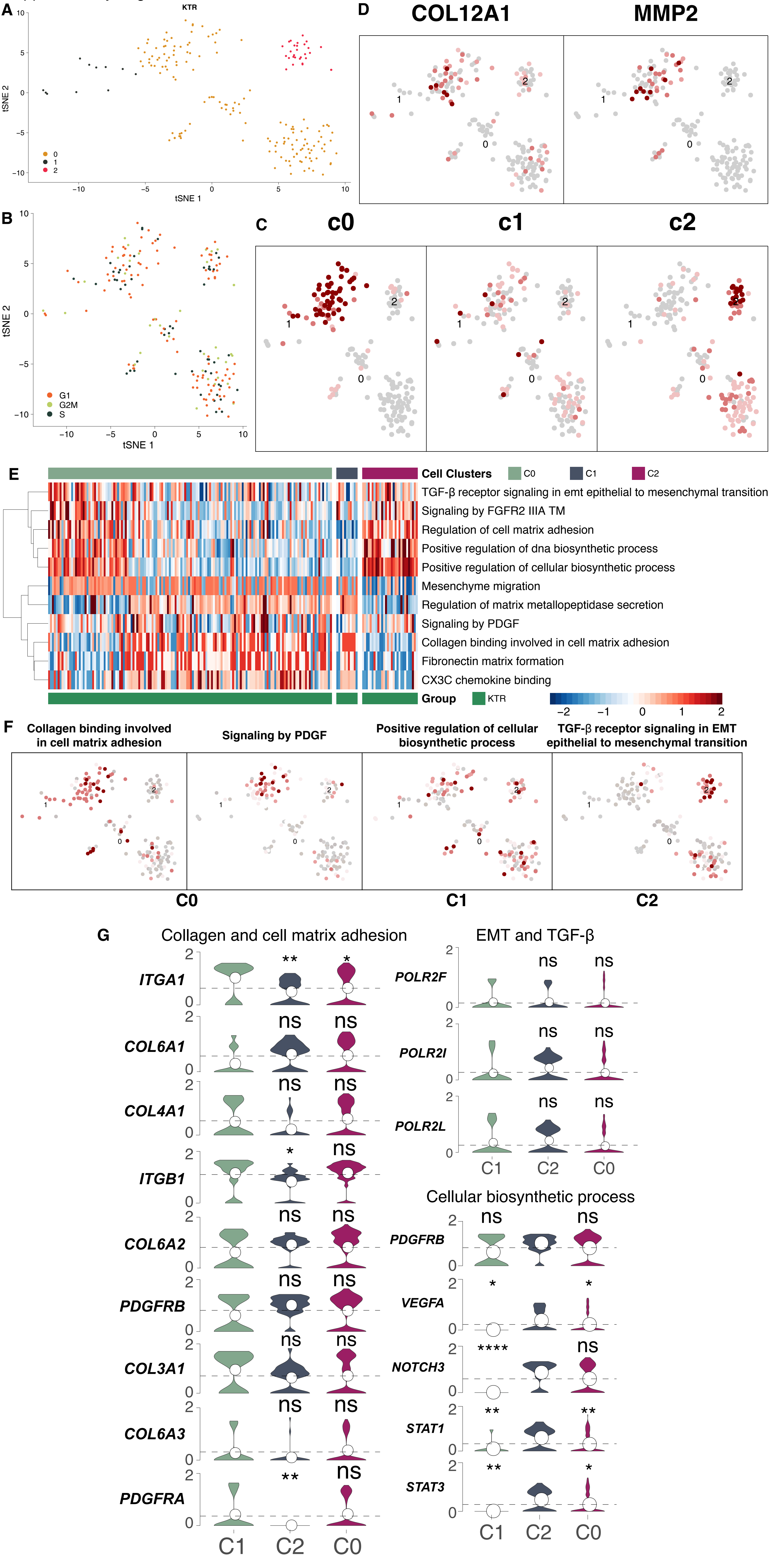

Table S1. Information of five different patients.

ID	Hospital	Age/Sex	Smoker/Dringker	Height(cm)/Weight(kg)	Sample	Surgery Date	Ref
kidney1	Zhujiang Hospital of the Southern Medical University, Guangzhou, Guangdong, China	30/male	Y/Y	174/62	chronic kidney transplant rejection	2012.11.21	Zhujiang Hospital of the Southern Medical University, Guangzhou, Guangdong, China
kidney2	Zhujiang Hospital of the Southern Medical University, Guangzhou, Guangdong, China	53/female	N/N	162/50	chronic kidney transplant rejection	2019.7.25	Zhujiang Hospital of the Southern Medical University, Guangzhou, Guangdong, China
kidney3	The first affiliated hospital of Guangxi Medical University	57/male	Y/Y	170/74	normal kidney	2018.4.16	PMID: 31896769
kidney4	Affiliated tumor hospital of Guangxi Medical University	59/female	N/N	152/47	normal kidney	2018.8.1	PMID: 31896769
kidney5	Affiliated tumor hospital of Guangxi Medical University	65/male	Y/N	162/59	normal kidney	2018.8.2	PMID: 31896769

Table S2.Characteristics of the five kidney tissue samples included in this study

ID	Q30 Bases in Barcode	Q30 Bases in RNA Read	Q30 Bases in UMI	Reads Mapped Confidently to Genome	Reads Mapped Confidently to Exonic Regions	Reads Mapped Confidently to Transcriptome	Total Genes Detected	Median UMI Counts per Cell
kidney1	96.20%	94.00%	95.80%	92.10%	56.00%	53.40%	25,036	3,489
kidney2	95.10%	92.40%	94.00%	89.40%	59.20%	56.00%	20,606	1,622
kidney3	95.70%	89.60%	95.10%	92.90%	82.90%	79.30%	8,164	837
kidney4	98.30%	96.90%	98.50%	87.40%	83.30%	79.60%	6,499	887
kidney5	98.30%	94.60%	98.40%	96.50%	88.50%	83.20%	10,741	684

Table S3. The marker genes for the fifteen cell clusters

cluster	gene	cell type	Figure
0	DCXR	Proximal tubule cells	related to figure 2e
1	SLC22A8	Proximal convoluted tubule cells	related to figure 2e
2	VCAM1	Glomerular parietal epithelial cells (1)	related to figure 2e
2	KRT18	Glomerular parietal epithelial cells (1)	related to Supplementary Figure 1
2	CD24	Glomerular parietal epithelial cells (1)	related to Supplementary Figure 1
3	GNLY	NKT cells	related to Supplementary Figure 1
3	NKG7	NKT cells	related to Supplementary Figure 1
3	CD3D	NKT cells	related to figure 2e
4	KRT18	Glomerular parietal epithelial cells (2)	related to figure 2e
4	KRT8	Glomerular parietal epithelial cells (2)	related to Supplementary Figure 1
4	CD24	Glomerular parietal epithelial cells (2)	related to Supplementary Figure 1
5	SLC22A7	Proximal straight tubule	related to figure 2e
6	CD79A	B cells (1)	related to Supplementary Figure 1
6	CD79B	B cells (1)	related to figure 2e
6	HLA-DPB1	B cells (1)	related to Supplementary Figure 1
7	LYZ	Monocytes	related to figure 2e
7	CD14	Monocytes	related to Supplementary Figure 1
8	DEFB1	Distal tubule cells	related to Supplementary Figure 1
8	UMOD	Distal tubule cells	related to figure 2e
9	AQP2	Collecting duct cells	related to figure 2e
9	TMEM213	Collecting duct cells	related to Supplementary Figure 1
9	ATP6V1G3	Collecting duct cells	related to Supplementary Figure 1
9	ATP6V0D2	Collecting duct cells	related to Supplementary Figure 1
10	PLVAP	Endothelial cell	related to Supplementary

			Figure 1
10	PECAM1	Endothelial cell	related to Supplementary Figure 1
10	FLT1	Endothelial cell	related to figure 2e
10	PTPRB	Endothelial cell	related to Supplementary Figure 1
11	COL3A1	Fibroblast	related to figure 2e
12	CD79A	B cells (2)	related to figure 2e
12	ITM2C	B cells (2)	related to Supplementary Figure 1
12	IGHG2	B cells (2)	related to Supplementary Figure 1
13	TPSB2	Mast cell	related to figure 2e
13	TPSAB1	Mast cell	related to Supplementary Figure 1
13	CTSG	Mast cell	related to Supplementary Figure 1
13	CPA3	Mast cell	related to Supplementary Figure 1
13	MS4A2	Mast cell	related to Supplementary Figure 1
13	HPGDS	Mast cell	related to Supplementary Figure 1
13	HDC	Mast cell	related to Supplementary Figure 1
14	PODXL	Nephron epithelial cell	related to figure 2e
14	PTPRO	Nephron epithelial cell	related to Supplementary Figure 1

Table S4 Differentially expressed genes (DEGs) for each cluster of human kidney cells.

p_val	avg_logFC	pct.1	pct.2	p_val_adj	cluster	gene
0	1.431160593	0.454	0.076	0	0	RBP4
0	1.358516362	0.85	0.507	0	0	DCXR
0	1.298387838	0.386	0.06	0	0	AGXT
0	1.282413535	0.887	0.502	0	0	MIOX
0	1.121463512	0.334	0.05	0	0	AC093496.1
0	1.020720425	0.6	0.243	0	0	GLYATL1
0	0.992639152	0.617	0.268	0	0	AKR7A3
0	0.968159928	0.797	0.428	0	0	PCK1
0	0.958918495	0.803	0.456	0	0	BBOX1
0	0.95834772	0.472	0.158	0	0	HAO2
0	0.923286997	0.666	0.337	0	0	ALDH6A1
0	0.917493447	0.629	0.32	0	0	ALDH2
0	0.910560089	0.465	0.174	0	0	ACY3
0	0.836893597	0.257	0.047	0	0	GCAT
0	0.831389977	0.797	0.499	0	0	ACAT1
0	0.823797924	0.494	0.21	0	0	MSRB1
0	0.823565326	0.482	0.257	0	0	SERPINA1
0	0.805184159	0.551	0.268	0	0	MPC1
0	0.795490989	0.603	0.309	0	0	ADIRF
0	0.79412924	0.768	0.457	0	0	RIDA
0	0.787639862	0.263	0.051	0	0	GPT
0	0.77802932	0.604	0.316	0	0	NIT2
0	0.762262068	0.609	0.335	0	0	BHMT
0	0.7544914	0.588	0.319	0	0	ACAA2
0	0.753229596	0.49	0.227	0	0	HGD
0	0.749199087	0.717	0.437	0	0	PEPD
0	0.7231811	0.622	0.358	0	0	GRHPR
0	0.704218055	0.728	0.431	0	0	GLYAT
0	0.702737795	0.658	0.379	0	0	CRYL1
0	0.69846642	0.538	0.296	0	0	ACADM
0	0.691686247	0.465	0.202	0	0	ASPDH
0	0.681685392	0.598	0.326	0	0	FBP1
0	0.677271763	0.964	0.794	0	0	PEBP1
0	0.67189846	0.728	0.451	0	0	C11orf54
0	0.667606042	0.499	0.239	0	0	MAOA
0	0.666102893	0.988	0.912	0	0	GAPDH
0	0.665695579	0.51	0.275	0	0	PTGR1

0	0.657817201	0.879	0.636	0	0	ECHS1
0	0.653048659	0.473	0.215	0	0	GSTA2
0	0.651737108	0.813	0.538	0	0	DAB2
0	0.649650286	0.526	0.292	0	0	ECI2
0	0.640631881	0.888	0.599	0	0	RBP5
0	0.639059651	0.997	0.823	0	0	MT1G
0	0.636852842	0.565	0.342	0	0	AKR7A2
0	0.623664793	0.426	0.221	0	0	PHYH
0	0.612071658	0.415	0.198	0	0	ACMSD
0	0.6107032	0.617	0.378	0	0	BDH2
0	0.604581097	0.948	0.759	0	0	TXN
0	0.601052729	0.727	0.495	0	0	AKR1A1
0	0.600153723	0.382	0.178	0	0	BPHL
0	0.600031985	0.698	0.483	0	0	ETFB
0	0.594647277	0.334	0.133	0	0	LINC01510
0	0.592559384	0.985	0.729	0	0	MT1F
0	0.579880135	0.929	0.643	0	0	ALDOB
0	0.57568818	0.639	0.447	0	0	ECH1
0	0.575151444	0.985	0.726	0	0	MT1H
0	0.572528767	0.717	0.432	0	0	HPD
0	0.559222892	0.669	0.443	0	0	PDZK1
0	0.553680824	0.928	0.706	0	0	CYB5A
0	0.552989978	0.655	0.455	0	0	SDHC
0	0.545385902	0.57	0.345	0	0	NQO2
0	0.543501152	0.789	0.5	0	0	GSTA1
0	0.542939431	0.574	0.358	0	0	BNIP3
0	0.527574446	0.758	0.577	0	0	DDT
0	0.505721877	0.928	0.78	0	0	SOD1
0	0.505425626	0.882	0.66	0	0	SUCLG1
0	0.496426129	0.929	0.783	0	0	GPX4
0	0.490316267	0.904	0.75	0	0	PRDX1
0	0.470827675	0.725	0.556	0	0	MGST3
0	0.459573211	0.924	0.781	0	0	LDHB
0	0.457547167	0.991	0.819	0	0	MT2A
0	0.4497475	0.909	0.653	0	0	GATM
0	0.445037547	0.716	0.514	0	0	SAT2
0	0.442988714	0.942	0.846	0	0	HINT1
0	0.432436605	0.976	0.737	0	0	MT1E
0	0.430207304	0.787	0.598	0	0	DSTN
0	0.420279165	0.998	0.979	0	0	FTL

0	0.40760832	0.988	0.766	0	0	MT1X
0	0.395092168	0.966	0.711	0	0	CRYAB
0	0.382529613	0.854	0.714	0	0	ATP5MC3
0	0.349579874	0.921	0.85	0	0	UBB
0	0.293259055	0.979	0.918	0	0	COX7C
0	0.270875049	0.991	0.959	0	0	RPS6
0	0.263024693	0.998	0.975	0	0	FTH1
9.89E-306	0.635036177	0.418	0.22	3.32E-301	0	HIBCH
1.12E-302	0.430456108	0.745	0.597	3.75E-298	0	ISCU
4.69E-300	0.618251535	0.416	0.222	1.57E-295	0	TUBB4B
1.22E-296	0.532414905	0.506	0.292	4.09E-292	0	AK4
4.60E-292	0.407429244	0.759	0.572	1.54E-287	0	ADI1
3.26E-288	0.511350248	0.604	0.413	1.09E-283	0	TMEM256
1.17E-285	0.556176211	0.474	0.268	3.92E-281	0	ACOT13
2.20E-282	0.523768193	0.608	0.429	7.39E-278	0	HSPD1
1.70E-280	0.572776688	0.431	0.232	5.71E-276	0	SHMT1
5.07E-280	0.284995271	0.963	0.905	1.70E-275	0	RPL31
1.23E-279	0.576154703	0.289	0.118	4.12E-275	0	ASRGL1
1.24E-279	0.58860249	0.289	0.119	4.15E-275	0	IDNK
1.72E-278	0.453405006	0.706	0.548	5.77E-274	0	ST13
2.33E-278	0.533251378	0.44	0.237	7.83E-274	0	CMBL
1.02E-275	0.396865921	0.762	0.594	3.41E-271	0	NDUFC1
3.98E-275	0.534883107	0.325	0.143	1.33E-270	0	SORD
6.87E-275	0.625139334	0.451	0.247	2.31E-270	0	AZGP1
6.64E-274	0.549202186	0.469	0.272	2.23E-269	0	HAGH
1.75E-271	0.440511168	0.674	0.483	5.87E-267	0	DNPH1
2.43E-268	0.387715944	0.734	0.544	8.14E-264	0	CYSTM1
2.50E-260	0.533088675	0.535	0.351	8.40E-256	0	ACAA1
1.98E-257	0.321927072	0.874	0.726	6.64E-253	0	LAMTOR5
5.74E-256	0.445148012	0.649	0.473	1.93E-251	0	MPC2
7.76E-254	0.55655915	0.332	0.158	2.60E-249	0	AHCY
9.36E-254	0.299452311	0.911	0.767	3.14E-249	0	UQCRQ
1.21E-251	0.541136707	0.264	0.107	4.05E-247	0	PTER
3.63E-248	0.421095432	0.615	0.404	1.22E-243	0	KHK
2.17E-243	0.410255109	0.695	0.541	7.28E-239	0	PRDX2
1.30E-242	0.355100692	0.793	0.668	4.37E-238	0	TPI1
8.75E-242	0.40988189	0.697	0.558	2.94E-237	0	PRR13
1.10E-241	0.573204166	0.265	0.112	3.69E-237	0	WDR18
2.30E-241	0.49871405	0.339	0.163	7.70E-237	0	AGMAT
1.13E-239	0.315640259	0.85	0.685	3.78E-235	0	HSPB1

6.13E-230	0.304379722	0.826	0.704	2.06E-225	0	ATP5PF
3.53E-229	0.466679503	0.512	0.326	1.18E-224	0	MSRA
1.12E-225	0.324824136	0.787	0.579	3.77E-221	0	LGALS2
9.83E-224	0.496445905	0.405	0.227	3.30E-219	0	GAMT
6.60E-222	0.265679673	0.901	0.803	2.21E-217	0	COX5B
4.90E-221	0.359622931	0.751	0.614	1.64E-216	0	CHCHD10
6.74E-220	0.464893922	0.403	0.224	2.26E-215	0	SMIM32
1.98E-218	0.489102862	0.36	0.192	6.64E-214	0	SCRN2
7.14E-216	0.462734626	0.498	0.321	2.40E-211	0	DHRS4L2
3.15E-215	0.313210619	0.807	0.691	1.06E-210	0	HSPE1
1.04E-212	0.315704433	0.819	0.72	3.47E-208	0	ATP5F1D
1.70E-212	0.487562701	0.474	0.306	5.69E-208	0	CNDP2
1.10E-206	0.439768669	0.606	0.464	3.69E-202	0	GHITM
8.99E-204	0.49297029	0.312	0.157	3.01E-199	0	TCEA3
1.96E-201	0.397462465	0.636	0.481	6.58E-197	0	PRDX3
2.54E-198	0.379513615	0.684	0.569	8.50E-194	0	C4orf3
2.12E-197	0.481661279	0.44	0.277	7.10E-193	0	SDHB
3.30E-195	0.416287911	0.558	0.392	1.11E-190	0	PLA2G16
3.88E-194	0.451524252	0.402	0.236	1.30E-189	0	MORN2
4.37E-192	0.322437732	0.789	0.71	1.47E-187	0	EDF1
1.29E-187	0.430373222	0.404	0.237	4.32E-183	0	BHMT2
3.07E-187	0.380437014	0.634	0.495	1.03E-182	0	SCP2
3.14E-187	0.417501124	0.489	0.316	1.05E-182	0	QPRT
3.65E-187	0.447538078	0.482	0.313	1.22E-182	0	AKR1C3
1.76E-183	0.430433891	0.335	0.18	5.89E-179	0	PXMP2
1.38E-182	0.384277137	0.583	0.432	4.64E-178	0	UQCRFS1
1.37E-180	0.424328459	0.475	0.312	4.60E-176	0	CRYZ
8.98E-180	0.460424026	0.285	0.144	3.01E-175	0	NUDT8
3.03E-179	0.43358728	0.346	0.191	1.02E-174	0	SLC6A13
2.56E-178	0.4114377	0.397	0.234	8.59E-174	0	HSD17B14
3.04E-176	0.476182758	0.499	0.354	1.02E-171	0	SIGIRR
6.71E-175	0.38503256	0.589	0.45	2.25E-170	0	NDUFS7
7.61E-175	0.440005902	0.308	0.163	2.55E-170	0	EPHX2
8.18E-175	0.293257694	0.756	0.638	2.74E-170	0	SLC25A5
5.83E-172	0.364130839	0.583	0.421	1.96E-167	0	PCBD1
8.87E-172	0.429330804	0.381	0.226	2.97E-167	0	FTCD
1.25E-170	0.368850839	0.7	0.608	4.20E-166	0	EEF2
1.15E-169	0.371098139	0.646	0.522	3.86E-165	0	ATP5F1A
2.66E-169	0.455532525	0.268	0.136	8.93E-165	0	NAPRT
1.69E-168	0.355465402	0.611	0.462	5.68E-164	0	RNF181

4.18E-168	0.446848881	0.276	0.141	1.40E-163	0	ENOSF1
1.77E-167	0.34427841	0.636	0.498	5.94E-163	0	NDUFS8
2.00E-167	0.461408933	0.259	0.127	6.69E-163	0	DHRS4
2.47E-167	0.484540357	0.259	0.131	8.27E-163	0	OAT
1.64E-164	0.255245118	0.852	0.795	5.50E-160	0	ATP5MC2
3.54E-164	0.370672733	0.628	0.517	1.19E-159	0	BRK1
1.56E-162	0.405387841	0.438	0.284	5.22E-158	0	MSRB2
4.08E-161	0.432838476	0.41	0.265	1.37E-156	0	ETFA
2.06E-158	0.355237222	0.589	0.444	6.92E-154	0	TXNDC17
5.88E-158	0.411519424	0.435	0.289	1.97E-153	0	AK2
1.00E-157	0.420382181	0.264	0.133	3.37E-153	0	PCK2
1.37E-156	0.402221595	0.379	0.233	4.60E-152	0	HIBADH
1.52E-156	0.272666888	0.782	0.712	5.11E-152	0	CHCHD2
4.66E-156	0.25058063	0.684	0.541	1.56E-151	0	MDH1
1.14E-154	0.262716839	0.809	0.721	3.81E-150	0	UQCR10
1.26E-154	0.378077529	0.451	0.298	4.22E-150	0	CISD3
2.31E-154	0.354533218	0.541	0.387	7.75E-150	0	CISD1
1.39E-152	0.400544579	0.427	0.278	4.67E-148	0	MPST
1.87E-152	0.390355203	0.437	0.287	6.27E-148	0	ISOC2
8.99E-152	0.429504971	0.276	0.147	3.01E-147	0	GALM
2.40E-150	0.381495473	0.438	0.287	8.05E-146	0	IMPA2
2.48E-150	0.370641333	0.519	0.374	8.32E-146	0	CFDP1
2.91E-150	0.393525575	0.312	0.174	9.77E-146	0	GPD1
8.23E-150	0.379711497	0.349	0.203	2.76E-145	0	AGXT2
1.80E-148	0.355283399	0.638	0.526	6.02E-144	0	ATP5F1B
2.26E-148	0.4377738	0.345	0.206	7.59E-144	0	AIFM1
3.43E-145	0.329799534	0.631	0.509	1.15E-140	0	ATP5F1C
2.84E-143	0.371097312	0.404	0.258	9.51E-139	0	PBLD
4.28E-143	0.316090485	0.64	0.503	1.44E-138	0	SLIRP
1.52E-140	0.317749335	0.66	0.542	5.09E-136	0	GLRX
4.25E-140	0.395092192	0.281	0.153	1.43E-135	0	ANGPTL3
8.83E-139	0.264132059	0.683	0.515	2.96E-134	0	ASS1
3.72E-138	0.389250024	0.299	0.169	1.25E-133	0	GIPC2
9.33E-138	0.395542395	0.369	0.234	3.13E-133	0	CLIC4
3.68E-136	0.264581554	0.775	0.677	1.23E-131	0	ATP5MF
2.51E-135	0.258005601	0.88	0.733	8.42E-131	0	SPP1
5.52E-134	0.369403702	0.314	0.182	1.85E-129	0	CLDN10
1.29E-132	0.328020427	0.612	0.495	4.32E-128	0	ZNHIT1
1.42E-130	0.260474569	0.722	0.586	4.75E-126	0	OCIAD2
2.67E-130	0.371750944	0.495	0.367	8.95E-126	0	ESD

2.87E-130	0.426071734	0.385	0.251	9.64E-126	0	GADD45A
1.82E-129	0.355044596	0.383	0.244	6.11E-125	0	DDC
1.87E-129	0.455504102	0.438	0.32	6.29E-125	0	SNHG8
2.69E-129	0.390264912	0.321	0.193	9.03E-125	0	STK19
7.06E-129	0.376759182	0.323	0.194	2.37E-124	0	FAH
9.51E-127	0.25185482	0.78	0.707	3.19E-122	0	SKP1
1.04E-126	0.384333857	0.322	0.195	3.50E-122	0	ALDH7A1
1.02E-125	0.343584334	0.363	0.226	3.43E-121	0	PPP1R16A
5.09E-125	0.326361726	0.594	0.483	1.71E-120	0	NDUFV2
1.53E-124	0.334065787	0.489	0.354	5.14E-120	0	HINT2
1.29E-123	0.294602749	0.621	0.51	4.33E-119	0	COX5A
3.38E-123	0.351326947	0.462	0.335	1.13E-118	0	NDUFV1
1.14E-122	0.380605059	0.442	0.321	3.82E-118	0	C1QBP
1.28E-122	0.346359461	0.472	0.339	4.29E-118	0	MRPS36
3.00E-121	0.267168584	0.705	0.616	1.01E-116	0	ATP5PO
4.65E-120	0.368720215	0.44	0.315	1.56E-115	0	SMS
4.72E-120	0.275384732	0.629	0.479	1.58E-115	0	S100A1
7.39E-120	0.284906191	0.65	0.491	2.48E-115	0	MGST1
7.60E-120	0.33273964	0.532	0.411	2.55E-115	0	TSTD1
1.96E-119	0.360251587	0.417	0.291	6.59E-115	0	SLC9A3R1
8.59E-117	0.358444867	0.389	0.264	2.88E-112	0	ANAPC13
1.69E-115	0.285500371	0.615	0.509	5.68E-111	0	NDUFB10
3.82E-115	0.276865506	0.656	0.551	1.28E-110	0	NDUFA2
1.72E-114	0.308054064	0.41	0.263	5.77E-110	0	APOM
4.93E-114	0.367037907	0.301	0.184	1.65E-109	0	ATP6V1B2
3.65E-113	0.336448931	0.503	0.387	1.23E-108	0	VDAC1
1.11E-111	0.410012265	0.33	0.216	3.74E-107	0	ARID5B
5.61E-111	0.359405655	0.482	0.369	1.88E-106	0	GSTO1
6.95E-111	0.383710855	0.368	0.251	2.33E-106	0	HEBP2
1.16E-110	0.293784321	0.511	0.378	3.89E-106	0	GCSH
1.98E-110	0.324554764	0.525	0.41	6.63E-106	0	NDUFAF3
4.75E-110	0.348733472	0.442	0.324	1.59E-105	0	CYC1
1.81E-109	0.319689299	0.417	0.289	6.07E-105	0	GCHFR
9.03E-109	0.341945941	0.451	0.334	3.03E-104	0	PSMB7
1.19E-108	0.269158845	0.62	0.509	3.98E-104	0	ATP5MC1
1.41E-107	0.278130205	0.463	0.324	4.71E-103	0	KRT8
1.98E-107	0.269753618	0.677	0.576	6.66E-103	0	NDUFA11
2.32E-107	0.306832859	0.646	0.572	7.77E-103	0	HSPA8
6.00E-107	0.363701568	0.271	0.162	2.01E-102	0	FYN
9.24E-107	0.35327489	0.266	0.157	3.10E-102	0	HMGCL

5.54E-106	0.333132908	0.305	0.19	1.86E-101	0	HDHD3
1.14E-105	0.352520242	0.337	0.221	3.81E-101	0	TPMT
3.95E-105	0.289984978	0.582	0.481	1.33E-100	0	AURKAIP1
4.45E-105	0.303261478	0.542	0.431	1.49E-100	0	MRPL57
1.89E-104	0.327112538	0.424	0.303	6.32E-100	0	TMEM141
2.15E-104	0.348378057	0.445	0.334	7.21E-100	0	MDH2
3.40E-104	0.347574115	0.371	0.25	1.14E-99	0	ALDH1A1
3.69E-104	0.2991225	0.344	0.219	1.24E-99	0	RAB11FIP3
1.53E-101	0.325655327	0.266	0.157	5.13E-97	0	SMIM1
7.87E-101	0.340453951	0.28	0.173	2.64E-96	0	ASL
1.91E-100	0.365832028	0.358	0.248	6.42E-96	0	HADHB
7.76E-100	0.303621989	0.414	0.288	2.60E-95	0	DPYS
1.03E-98	0.279099426	0.436	0.309	3.46E-94	0	CAMK2N1
3.81E-98	0.320288338	0.444	0.327	1.28E-93	0	MTCH2
1.07E-97	0.350483782	0.309	0.202	3.57E-93	0	ABHD14B
1.12E-97	0.335884857	0.395	0.284	3.74E-93	0	UQCRC1
3.26E-97	0.335173817	0.444	0.335	1.09E-92	0	DECR1
4.97E-97	0.315312424	0.418	0.302	1.67E-92	0	PSMB5
5.53E-97	0.324834009	0.327	0.216	1.85E-92	0	MCRIP2
1.53E-94	0.326187281	0.325	0.215	5.13E-90	0	AP1S1
1.80E-94	0.263272013	0.617	0.523	6.03E-90	0	NDUFB8
8.61E-93	0.331444283	0.368	0.259	2.89E-88	0	SUCLG2
4.91E-92	0.254239272	0.609	0.512	1.65E-87	0	FIS1
4.63E-91	0.30639675	0.278	0.174	1.55E-86	0	TST
9.33E-91	0.343516171	0.259	0.16	3.13E-86	0	SLC39A4
1.69E-90	0.311823599	0.447	0.337	5.68E-86	0	GTF2H5
5.31E-90	0.347642838	0.368	0.265	1.78E-85	0	ACADVL
1.16E-89	0.309476564	0.407	0.298	3.89E-85	0	HSD17B10
1.63E-89	0.331478571	0.31	0.2	5.47E-85	0	FOLR1
6.68E-87	0.258976451	0.547	0.44	2.24E-82	0	CAMTA1
4.35E-86	0.338067781	0.338	0.237	1.46E-81	0	NDUFA10
3.77E-85	0.29160839	0.352	0.241	1.26E-80	0	AP1M2
3.81E-85	0.303576004	0.399	0.295	1.28E-80	0	RPA3
1.21E-84	0.366018866	0.497	0.416	4.05E-80	0	LDHA
2.85E-84	0.330844299	0.251	0.157	9.57E-80	0	GRPEL1
2.89E-84	0.306420383	0.518	0.429	9.68E-80	0	GSTK1
2.73E-83	0.260910938	0.591	0.519	9.15E-79	0	ARL6IP4
5.70E-83	0.280857636	0.495	0.4	1.91E-78	0	NDUFAB1
2.14E-82	0.296985116	0.498	0.402	7.18E-78	0	SCAND1
8.64E-82	0.283822792	0.341	0.234	2.90E-77	0	FUOM

1.62E-81	0.281018931	0.506	0.408	5.45E-77	0	CHMP2A
2.18E-81	0.337029772	0.304	0.208	7.31E-77	0	IDH3G
1.58E-80	0.311254463	0.423	0.327	5.30E-76	0	SDHD
3.32E-80	0.288780734	0.269	0.172	1.11E-75	0	MCUR1
7.84E-80	0.267734981	0.503	0.4	2.63E-75	0	LAMTOR2
7.05E-79	0.254914712	0.53	0.436	2.37E-74	0	NDUFA5
1.46E-78	0.306270994	0.276	0.182	4.89E-74	0	MRPS35
4.09E-78	0.321566655	0.318	0.223	1.37E-73	0	STOML2
1.81E-77	0.258410249	0.544	0.448	6.07E-73	0	LSM3
6.30E-76	0.305343178	0.429	0.335	2.11E-71	0	TUFM
3.34E-75	0.308014444	0.333	0.237	1.12E-70	0	SCOC
2.15E-74	0.319453551	0.361	0.268	7.22E-70	0	NDUFS2
8.11E-74	0.289468314	0.398	0.301	2.72E-69	0	NDUFS4
1.13E-73	0.256902768	0.502	0.405	3.78E-69	0	TIMM13
1.61E-73	0.257473685	0.461	0.36	5.41E-69	0	PTMS
1.77E-73	0.280222284	0.48	0.388	5.93E-69	0	PSMB6
3.45E-73	0.28357674	0.483	0.394	1.16E-68	0	SMIM26
4.36E-73	0.289204969	0.416	0.319	1.46E-68	0	MTIF3
9.13E-73	0.295233341	0.411	0.318	3.06E-68	0	RAB1A
2.15E-72	0.279427474	0.283	0.189	7.22E-68	0	GABARAPL1
2.46E-72	0.271725146	0.539	0.464	8.25E-68	0	CIB1
1.16E-71	0.301145347	0.377	0.284	3.89E-67	0	SEPHS2
2.46E-71	0.289622073	0.485	0.402	8.23E-67	0	PRELID1
6.50E-71	0.277674125	0.396	0.3	2.18E-66	0	MRPL34
3.36E-68	0.318254087	0.292	0.206	1.13E-63	0	BIN1
4.15E-67	0.291451939	0.301	0.212	1.39E-62	0	CCNG1
4.53E-67	0.282680975	0.281	0.194	1.52E-62	0	PMVK
9.85E-66	0.30746967	0.289	0.205	3.30E-61	0	MRPL40
2.12E-65	0.26624487	0.374	0.271	7.12E-61	0	PCP4
9.56E-64	0.294688009	0.273	0.19	3.21E-59	0	RDX
1.09E-63	0.251451941	0.458	0.372	3.66E-59	0	GLS
1.52E-63	0.278165662	0.353	0.265	5.09E-59	0	IER3IP1
4.18E-63	0.289440078	0.323	0.237	1.40E-58	0	TRAPPC6A
3.94E-62	0.254634139	0.423	0.329	1.32E-57	0	BLVRB
3.24E-61	0.273945345	0.255	0.173	1.09E-56	0	CHP1
5.20E-61	0.251085365	0.528	0.461	1.74E-56	0	APRT
5.56E-61	0.2714371	0.303	0.218	1.87E-56	0	ACO2
1.74E-57	0.265508673	0.487	0.415	5.83E-53	0	PGK1
4.19E-56	0.261685507	0.392	0.316	1.40E-51	0	METTL9
4.57E-56	0.259752044	0.272	0.192	1.53E-51	0	RBPMS

5.96E-56	0.301375603	0.347	0.27	2.00E-51	0	ZC3H15
4.55E-55	0.274256922	0.29	0.211	1.53E-50	0	MRPL21
5.62E-55	0.272745592	0.341	0.26	1.89E-50	0	MGMT
7.60E-55	0.286159851	0.339	0.261	2.55E-50	0	UQCRC2
4.01E-54	0.266544715	0.277	0.196	1.35E-49	0	MIR4458HG
1.08E-53	0.278916768	0.364	0.286	3.61E-49	0	CHMP5
4.59E-52	0.262432832	0.281	0.202	1.54E-47	0	HIST1H1C
5.35E-52	0.271022383	0.333	0.258	1.80E-47	0	ADH5
3.84E-51	0.257117258	0.359	0.279	1.29E-46	0	MRPS33
5.55E-51	0.267982091	0.331	0.257	1.86E-46	0	MLF2
2.45E-38	0.251190678	0.254	0.193	8.23E-34	0	MMADHC
0	1.401643361	0.323	0.094	0	1	MTRNR2L1
0	1.235874789	0.81	0.286	0	1	FABP1
0	1.232029453	0.992	0.789	0	1	GPX3
0	1.172709275	0.291	0.076	0	1	MTRNR2L10
0	1.122065145	0.333	0.14	0	1	MTRNR2L8
0	1.065981399	0.399	0.091	0	1	FAM151A
0	1.052871445	0.429	0.118	0	1	SLC22A8
0	1.043706613	0.272	0.066	0	1	MTRNR2L6
0	1.025828904	0.589	0.267	0	1	DPEP1
0	1.001283916	0.533	0.203	0	1	PRODH2
0	0.993130668	0.475	0.175	0	1	ALB
0	0.931611866	0.963	0.709	0	1	APOE
0	0.915106794	0.723	0.416	0	1	UGT2B7
0	0.85063366	0.417	0.165	0	1	PRAP1
0	0.783797591	0.832	0.501	0	1	GSTA1
0	0.783529877	0.405	0.155	0	1	MCCD1
0	0.774220554	0.283	0.095	0	1	ARG2
0	0.767911996	0.808	0.515	0	1	SMIM24
0	0.7604175	0.293	0.105	0	1	LGMN
0	0.739622467	0.792	0.468	0	1	PCSK1N
0	0.715574375	0.925	0.64	0	1	CXCL14
0	0.699433872	0.771	0.429	0	1	HPD
0	0.683864375	0.492	0.241	0	1	APOM
0	0.677887961	0.391	0.183	0	1	SLC7A7
0	0.652785519	0.967	0.645	0	1	ALDOB
0	0.629658572	0.681	0.459	0	1	SELENOP
0	0.607197801	0.746	0.502	0	1	ASS1
0	0.561338494	0.965	0.712	0	1	PDZK1IP1
0	0.549633423	0.98	0.781	0	1	MT1X

0	0.505839775	0.902	0.67	0	1	GATM
0	0.499580994	0.998	0.98	0	1	FTL
0	0.483360469	0.748	0.579	0	1	ATOX1
0	0.475435518	0.874	0.675	0	1	SUCLG1
0	0.448268066	0.805	0.583	0	1	NAT8
0	0.420078608	0.872	0.62	0	1	RBP5
0	0.415757332	0.879	0.754	0	1	NDUFB2
0	0.395438066	0.877	0.757	0	1	RPS27L
0	0.295079115	0.996	0.977	0	1	FTH1
4.25E-306	0.650944744	0.387	0.186	1.42E-301	1	PAH
1.51E-305	0.505987505	0.633	0.409	5.08E-301	1	KHK
5.19E-305	0.363650195	0.898	0.729	1.74E-300	1	CYB5A
2.91E-284	0.59106915	0.698	0.544	9.74E-280	1	MDH1
2.66E-280	0.404048656	0.789	0.59	8.91E-276	1	LGALS2
5.29E-278	0.450982709	0.706	0.529	1.78E-273	1	SAT2
3.33E-276	0.49726964	0.917	0.826	1.12E-271	1	MT-ND2
4.89E-271	0.438083889	0.961	0.888	1.64E-266	1	MT-CO3
1.72E-265	0.473371286	0.653	0.464	5.76E-261	1	TMEM176A
3.84E-264	0.378002498	0.903	0.778	1.29E-259	1	UQCRQ
2.37E-259	0.322848832	0.915	0.846	7.94E-255	1	NDUFA4
1.73E-253	0.411808425	0.747	0.586	5.82E-249	1	ADI1
3.05E-252	0.580806147	0.326	0.15	1.02E-247	1	SLC22A6
1.91E-251	0.479028141	0.652	0.504	6.42E-247	1	NPC2
1.66E-248	0.280991169	0.972	0.924	5.56E-244	1	COX7C
1.47E-246	0.549805408	0.661	0.475	4.94E-242	1	S100A1
2.59E-241	0.583446229	0.312	0.144	8.68E-237	1	SLC16A9
6.22E-241	0.36181015	0.966	0.898	2.08E-236	1	MT-CO2
1.01E-233	0.465429035	0.882	0.774	3.39E-229	1	MT-ND1
1.81E-233	0.404155342	0.937	0.85	6.06E-229	1	MT-ND4
4.44E-229	0.407432099	0.756	0.646	1.49E-224	1	ATP5IF1
1.32E-218	0.320956392	0.882	0.764	4.43E-214	1	DBI
1.12E-216	0.383439668	0.962	0.886	3.75E-212	1	MT-CO1
1.18E-216	0.294535996	0.901	0.798	3.94E-212	1	SOD1
5.78E-216	0.358612559	0.79	0.704	1.94E-211	1	COX8A
7.24E-215	0.569595805	0.272	0.122	2.43E-210	1	SLC4A4
4.32E-211	0.338628182	0.973	0.835	1.45E-206	1	MT2A
9.95E-206	0.328519523	0.992	0.834	3.34E-201	1	MT1G
1.31E-202	0.306305778	0.956	0.754	4.41E-198	1	MT1F
1.06E-198	0.53590427	0.296	0.144	3.56E-194	1	SLC13A3
2.77E-198	0.351366501	0.799	0.739	9.28E-194	1	COX6C

4.74E-196	0.513091338	0.458	0.299	1.59E-191	1	QDPR
3.52E-192	0.364821706	0.785	0.705	1.18E-187	1	HSPE1
1.11E-183	0.287797462	0.861	0.74	3.71E-179	1	LAMTOR5
8.65E-182	0.520928499	0.393	0.242	2.90E-177	1	CTSB
5.13E-181	0.530813652	0.332	0.185	1.72E-176	1	PSAT1
1.79E-180	0.44185739	0.449	0.282	6.01E-176	1	DPYS
1.93E-178	0.257510451	0.518	0.349	6.46E-174	1	IGFBP7
2.38E-174	0.297784649	0.842	0.778	7.98E-170	1	ITM2B
8.44E-171	0.528194867	0.35	0.204	2.83E-166	1	PTH1R
1.94E-170	0.537158454	0.446	0.304	6.51E-166	1	CTSH
4.85E-168	0.342391978	0.944	0.754	1.63E-163	1	MT1H
1.83E-166	0.36054364	0.795	0.704	6.15E-162	1	POLR2L
2.49E-164	0.660204818	0.41	0.252	8.34E-160	1	GSTA2
4.54E-162	0.302789374	0.797	0.721	1.52E-157	1	ATP5PF
2.62E-161	0.50197397	0.279	0.144	8.77E-157	1	NUPR1
2.46E-157	0.409958975	0.577	0.45	8.26E-153	1	FKBP2
4.88E-157	0.408425714	0.586	0.445	1.64E-152	1	TMEM176B
6.24E-155	0.282453067	0.94	0.763	2.09E-150	1	MT1E
6.21E-153	0.323875155	0.785	0.709	2.08E-148	1	NDUFA1
3.33E-144	0.431759993	0.36	0.221	1.12E-139	1	RAB11FIP3
2.18E-143	0.312333127	0.913	0.818	7.31E-139	1	MT-CYB
5.65E-141	0.315887095	0.639	0.506	1.90E-136	1	DNPH1
1.10E-136	0.334845034	0.56	0.419	3.69E-132	1	ATP1B1
3.83E-131	0.460471049	0.33	0.204	1.28E-126	1	GGH
5.26E-127	0.327403798	0.653	0.567	1.76E-122	1	ATP6V1F
5.21E-126	0.255408222	0.64	0.478	1.75E-121	1	GLYAT
8.67E-125	0.336194397	0.802	0.756	2.91E-120	1	ATP5ME
1.99E-124	0.268207422	0.792	0.732	6.68E-120	1	UQCR10
1.37E-123	0.408095723	0.575	0.49	4.60E-119	1	TMBIM6
1.15E-122	0.342136519	0.486	0.345	3.86E-118	1	MSRA
1.54E-122	0.388369824	0.423	0.294	5.15E-118	1	GCHFR
2.04E-120	0.375212747	0.381	0.248	6.84E-116	1	HSD17B14
9.71E-120	0.3984578	0.403	0.273	3.26E-115	1	CA2
5.85E-118	0.268314647	0.744	0.664	1.96E-113	1	PPDPF
8.05E-117	0.434140874	0.319	0.195	2.70E-112	1	CUBN
8.84E-117	0.278939872	0.785	0.73	2.96E-112	1	ATP5MPL
1.12E-112	0.277880147	0.717	0.619	3.74E-108	1	NDUFC1
4.14E-108	0.266279139	0.778	0.712	1.39E-103	1	COX7B
4.63E-107	0.441820731	0.347	0.237	1.55E-102	1	FCGRT
2.34E-106	0.364741929	0.358	0.236	7.83E-102	1	PPP1R16A

2.58E-106	0.260160465	0.726	0.655	8.66E-102	1	SLC25A5
4.30E-106	0.339221264	0.323	0.202	1.44E-101	1	FOLR1
1.53E-102	0.43480828	0.298	0.19	5.13E-98	1	ALDH4A1
7.33E-100	0.326405267	0.549	0.443	2.46E-95	1	PCBD1
1.83E-96	0.266705032	0.772	0.718	6.13E-92	1	ATP5MD
1.34E-95	0.491427904	0.266	0.17	4.48E-91	1	SLC3A2
3.70E-92	0.353246607	0.33	0.218	1.24E-87	1	AGXT2
1.04E-91	0.522172635	0.378	0.275	3.50E-87	1	PCP4
1.54E-88	0.371856483	0.437	0.345	5.18E-84	1	NENF
1.31E-85	0.273982977	0.671	0.607	4.41E-81	1	NDUFB4
4.52E-85	0.269198084	0.499	0.383	1.51E-80	1	NQO2
2.21E-84	0.257394646	0.64	0.556	7.40E-80	1	GLRX
1.50E-81	0.33576239	0.363	0.259	5.03E-77	1	DDC
8.03E-81	0.273803525	0.495	0.378	2.69E-76	1	FBP1
1.03E-79	0.371326853	0.312	0.218	3.45E-75	1	KIAA1191
1.46E-79	0.306370664	0.698	0.65	4.89E-75	1	NDUFB1
4.04E-79	0.308355221	0.488	0.393	1.35E-74	1	GCSH
4.17E-78	0.298530857	0.565	0.493	1.40E-73	1	COX14
7.13E-78	0.383675651	0.301	0.21	2.39E-73	1	PGRMC1
7.22E-76	0.54159881	0.325	0.239	2.42E-71	1	MTRNR2L12
8.96E-75	0.309648668	0.461	0.368	3.01E-70	1	SH3YL1
4.49E-74	0.259903708	0.629	0.566	1.51E-69	1	NDUFA2
1.33E-72	0.273369256	0.547	0.467	4.45E-68	1	TXNDC17
4.73E-72	0.292937043	0.287	0.19	1.59E-67	1	GPD1
1.35E-71	0.277011234	0.442	0.343	4.54E-67	1	QPRT
1.24E-65	0.260193854	0.725	0.703	4.17E-61	1	UQCR11
1.66E-60	0.279382705	0.506	0.445	5.56E-56	1	MRPL41
3.28E-59	0.306820609	0.311	0.229	1.10E-54	1	MACROD1
9.60E-58	0.311528178	0.287	0.207	3.22E-53	1	MGST2
2.88E-56	0.287809351	0.355	0.269	9.66E-52	1	GADD45A
8.80E-56	0.315444376	0.325	0.246	2.95E-51	1	FUOM
2.27E-54	0.271478971	0.483	0.422	7.60E-50	1	RTN4
2.41E-52	0.311662383	0.318	0.246	8.07E-48	1	SLC25A39
7.36E-52	0.251582567	0.508	0.45	2.47E-47	1	MRPL57
9.01E-51	0.331097109	0.403	0.343	3.02E-46	1	PHB
5.67E-50	0.272893578	0.31	0.232	1.90E-45	1	AMN
1.78E-45	0.288386571	0.271	0.2	5.98E-41	1	LINC02294
2.99E-45	0.274238448	0.521	0.483	1.00E-40	1	COPS9
1.25E-44	0.250003306	0.511	0.466	4.21E-40	1	LSM3
1.88E-44	0.292886158	0.262	0.195	6.31E-40	1	AIG1

8.78E-43	0.328567522	0.263	0.201	2.94E-38	1	CTSA
2.13E-42	0.253846522	0.327	0.256	7.15E-38	1	AP1M2
1.09E-40	0.251846484	0.475	0.431	3.65E-36	1	FAM96B
3.14E-39	0.255326988	0.421	0.359	1.05E-34	1	SPATS2L
1.23E-36	0.254436838	0.297	0.236	4.12E-32	1	HADH
2.81E-35	0.269862706	0.284	0.227	9.43E-31	1	C1orf122
1.04E-32	0.273706226	0.316	0.264	3.49E-28	1	CAT
2.45E-32	0.255003008	0.254	0.195	8.22E-28	1	LRP2
0	1.222687567	0.913	0.565	0	2	GSTP1
0	1.069560628	0.532	0.166	0	2	PIGR
0	0.915706728	0.696	0.324	0	2	TNFSF10
0	0.866243983	0.625	0.272	0	2	SOD2
0	0.850874422	0.966	0.697	0	2	IL32
0	0.808192371	0.428	0.111	0	2	CDH6
0	0.76878462	0.391	0.122	0	2	VCAM1
0	0.643018268	0.863	0.519	0	2	PCSK1N
0	0.613363387	0.974	0.756	0	2	SPP1
8.70E-299	0.673075219	0.427	0.148	2.92E-294	2	APP
5.17E-279	0.675761255	0.782	0.495	1.73E-274	2	LAPTM4A
1.60E-273	0.655234397	0.684	0.36	5.38E-269	2	KRT18
8.52E-270	0.490077295	0.937	0.714	2.86E-265	2	TMSB10
3.92E-268	0.628334569	0.664	0.353	1.31E-263	2	CD24
4.14E-265	0.488058108	0.953	0.72	1.39E-260	2	FXYD2
6.41E-262	0.456337648	0.958	0.854	2.15E-257	2	MYL6
2.21E-258	0.670793812	0.593	0.286	7.40E-254	2	TSPAN1
1.92E-243	0.571433673	0.713	0.416	6.43E-239	2	C12orf75
8.07E-227	0.60644029	0.713	0.409	2.71E-222	2	CLTRN
2.40E-224	0.571506957	0.652	0.348	8.03E-220	2	FABP3
3.92E-221	0.464050905	0.789	0.502	1.32E-216	2	S100A11
5.25E-221	0.611513205	0.441	0.184	1.76E-216	2	NAPSA
5.89E-220	0.56828678	0.374	0.139	1.98E-215	2	THY1
1.65E-218	0.439244805	0.673	0.361	5.53E-214	2	IGFBP7
5.47E-209	0.557739632	0.322	0.113	1.84E-204	2	ADGRG1
7.28E-206	0.456916566	0.939	0.779	2.44E-201	2	ITM2B
5.03E-197	0.519697688	0.79	0.51	1.69E-192	2	MGST1
1.27E-195	0.38738942	0.98	0.756	4.27E-191	2	PDZK1IP1
3.31E-195	0.538487136	0.539	0.272	1.11E-190	2	HCFC1R1
1.61E-189	0.553189314	0.531	0.276	5.40E-185	2	ANXA4
2.01E-182	0.497376104	0.739	0.487	6.74E-178	2	CD63
1.05E-177	0.522141488	0.571	0.313	3.51E-173	2	CARHSP1

1.72E-169	0.505012433	0.583	0.325	5.78E-165	2	CNN3
9.56E-166	0.513283705	0.293	0.11	3.21E-161	2	SERPINE2
1.53E-164	0.395193823	0.888	0.613	5.13E-160	2	NAT8
2.68E-162	0.352750073	0.942	0.778	8.98E-158	2	DBI
9.59E-162	0.513261931	0.398	0.179	3.22E-157	2	RARRES2
2.24E-157	0.392906403	0.619	0.352	7.51E-153	2	IFITM3
5.49E-150	0.504600174	0.475	0.253	1.84E-145	2	CDK2AP2
5.70E-148	0.437190413	0.574	0.324	1.91E-143	2	S100A13
1.92E-147	0.467176774	0.372	0.17	6.43E-143	2	HIF1A
6.87E-144	0.420869266	0.404	0.188	2.31E-139	2	LRP2
2.25E-143	0.457936836	0.31	0.129	7.54E-139	2	SDC4
4.90E-140	0.438462449	0.708	0.475	1.64E-135	2	RARRES3
1.85E-138	0.439067351	0.387	0.186	6.21E-134	2	STMN1
1.81E-134	0.420733185	0.252	0.096	6.08E-130	2	TNFRSF12A
2.61E-132	0.256679359	0.987	0.946	8.74E-128	2	RPL7
3.02E-129	0.401514954	0.667	0.427	1.01E-124	2	DUSP23
5.72E-126	0.437057757	0.458	0.242	1.92E-121	2	CLU
5.84E-124	0.420476141	0.426	0.223	1.96E-119	2	APLP2
9.79E-124	0.416338042	0.333	0.154	3.28E-119	2	TAX1BP3
1.80E-123	0.438073678	0.286	0.124	6.02E-119	2	CD151
4.60E-121	0.371773456	0.303	0.133	1.54E-116	2	BCAM
6.78E-121	0.397737446	0.424	0.22	2.27E-116	2	AQP1
2.23E-119	0.319416053	0.625	0.395	7.49E-115	2	S100A10
1.41E-114	0.392535538	0.302	0.136	4.74E-110	2	ENPEP
1.32E-113	0.366849842	0.428	0.226	4.43E-109	2	TCEAL9
7.30E-113	0.277358457	0.975	0.767	2.45E-108	2	CRYAB
1.84E-110	0.33533506	0.809	0.607	6.16E-106	2	OCIAD2
4.12E-110	0.421929075	0.406	0.221	1.38E-105	2	TAGLN2
7.56E-107	0.368514183	0.562	0.344	2.54E-102	2	KRT8
2.72E-104	0.30672299	0.844	0.707	9.11E-100	2	MYL12B
1.78E-100	0.346731904	0.341	0.169	5.98E-96	2	AC245595.1
1.49E-99	0.268249126	0.88	0.71	4.99E-95	2	POLR2L
1.88E-99	0.358878027	0.326	0.161	6.29E-95	2	EPCAM
1.88E-99	0.343572779	0.468	0.275	6.29E-95	2	CTSD
4.03E-97	0.287115422	0.867	0.744	1.35E-92	2	PPIA
1.69E-95	0.324622345	0.429	0.238	5.65E-91	2	SLC3A1
6.86E-94	0.338954591	0.255	0.116	2.30E-89	2	SOX4
2.04E-93	0.342359484	0.739	0.562	6.85E-89	2	ENO1
7.11E-92	0.327570489	0.638	0.438	2.38E-87	2	RHOC
1.20E-91	0.275135547	0.891	0.718	4.02E-87	2	HSPB1

3.52E-89	0.354547827	0.304	0.152	1.18E-84	2	SLC22A2
2.10E-88	0.332839233	0.742	0.585	7.05E-84	2	NPM1
2.04E-85	0.262277734	0.836	0.663	6.84E-81	2	PRDX5
3.12E-85	0.280634241	0.716	0.49	1.05E-80	2	TMEM176A
3.35E-85	0.251594375	0.955	0.838	1.12E-80	2	MT-ND2
9.13E-84	0.252352684	0.897	0.792	3.06E-79	2	UQCRB
3.98E-83	0.338759405	0.447	0.269	1.33E-78	2	LYPLAL1
4.33E-82	0.35524914	0.342	0.189	1.45E-77	2	LEPROT
1.28E-81	0.31361304	0.293	0.148	4.30E-77	2	TMEM37
3.31E-81	0.250642172	0.806	0.568	1.11E-76	2	SMIM24
3.07E-77	0.253023897	0.824	0.669	1.03E-72	2	PPDPF
3.78E-76	0.356633384	0.31	0.171	1.27E-71	2	TRAM1
4.09E-75	0.2540728	0.971	0.862	1.37E-70	2	MT-ND4
1.88E-74	0.310915317	0.705	0.56	6.31E-70	2	HNRNPA1
4.38E-74	0.295944825	0.468	0.289	1.47E-69	2	CA2
6.15E-74	0.300032184	0.377	0.218	2.06E-69	2	PGRMC1
6.30E-73	0.305398098	0.272	0.141	2.11E-68	2	GRN
1.04E-72	0.318713116	0.32	0.178	3.50E-68	2	KRTCAP2
1.55E-71	0.295666268	0.345	0.194	5.21E-67	2	ACOT7
2.19E-71	0.314175797	0.312	0.17	7.35E-67	2	CFI
5.15E-71	0.333870592	0.284	0.153	1.73E-66	2	SELENOM
9.93E-71	0.253135953	0.67	0.461	3.33E-66	2	TMEM176B
3.14E-70	0.272336364	0.288	0.153	1.05E-65	2	GNG12
9.67E-70	0.255123139	0.347	0.198	3.24E-65	2	CD59
5.36E-68	0.301433174	0.424	0.269	1.80E-63	2	HSP90B1
2.67E-67	0.290248832	0.556	0.386	8.94E-63	2	PPA1
4.09E-67	0.275321459	0.427	0.261	1.37E-62	2	SLC13A1
1.82E-66	0.273086713	0.296	0.162	6.10E-62	2	SLC16A4
1.58E-64	0.250851	0.65	0.477	5.29E-60	2	NDUFA13
2.43E-64	0.308083704	0.377	0.233	8.14E-60	2	TMEM147
1.61E-63	0.291813256	0.343	0.203	5.41E-59	2	CTSA
4.36E-63	0.282637895	0.268	0.143	1.46E-58	2	SLC17A3
1.32E-62	0.31315285	0.451	0.304	4.42E-58	2	RABAC1
2.66E-62	0.283195376	0.65	0.502	8.92E-58	2	ATP6V0E1
2.73E-62	0.257905273	0.542	0.378	9.16E-58	2	CSTB
2.24E-61	0.268419119	0.713	0.577	7.51E-57	2	SUMO2
1.59E-60	0.267718495	0.601	0.436	5.33E-56	2	RPL17
2.38E-60	0.267103287	0.391	0.242	7.98E-56	2	LACTB2
2.42E-59	0.28228826	0.35	0.21	8.12E-55	2	PLIN2
4.86E-59	0.298611653	0.269	0.15	1.63E-54	2	LAMP2

7.52E-59	0.262109177	0.567	0.408	2.52E-54	2	HSBP1
4.03E-58	0.259396218	0.564	0.408	1.35E-53	2	TRAPPC1
2.40E-55	0.293957237	0.3	0.178	8.06E-51	2	PSENEN
9.79E-55	0.288244262	0.505	0.363	3.28E-50	2	DAD1
5.91E-53	0.256559374	0.614	0.466	1.98E-48	2	RPL36A
4.14E-52	0.276492924	0.522	0.379	1.39E-47	2	TMEM59
1.38E-50	0.268557309	0.325	0.203	4.62E-46	2	JPT1
2.37E-50	0.269048296	0.509	0.369	7.95E-46	2	NDUFC2
2.04E-49	0.276024842	0.419	0.285	6.86E-45	2	DNAJC15
4.72E-49	0.251631766	0.308	0.189	1.58E-44	2	SPINT2
3.68E-41	0.255972325	0.361	0.248	1.23E-36	2	TMBIM4
0	3.071454818	0.648	0.01	0	3	CCL5
0	2.918103039	0.27	0.005	0	3	GNLY
0	2.877649023	0.537	0.009	0	3	NKG7
0	2.33949314	0.574	0.003	0	3	GZMA
0	2.331287208	0.747	0.01	0	3	TRAC
0	2.315445992	0.441	0.007	0	3	CCL4
0	2.209578619	0.747	0.004	0	3	CD3D
0	2.142838975	0.72	0.004	0	3	CD2
0	2.112864655	0.826	0.072	0	3	CD52
0	2.073793038	0.993	0.418	0	3	TMSB4X
0	2.046491106	0.83	0.064	0	3	PTPRC
0	2.045490671	0.776	0.036	0	3	HCST
0	2.006241795	0.51	0.002	0	3	TRBC1
0	1.969936789	0.637	0.015	0	3	TRBC2
0	1.890958493	0.366	0.002	0	3	GZMH
0	1.879895256	0.995	0.829	0	3	B2M
0	1.876835571	0.711	0.003	0	3	CD3E
0	1.874541559	0.534	0.004	0	3	CST7
0	1.856147635	0.956	0.348	0	3	HLA-A
0	1.842217247	0.377	0.003	0	3	IL7R
0	1.840185007	0.274	0.002	0	3	GZMB
0	1.839624786	0.859	0.078	0	3	S100A4
0	1.816818676	0.955	0.309	0	3	HLA-C
0	1.79887776	0.317	0.002	0	3	KLRB1
0	1.782874624	0.785	0.065	0	3	CORO1A
0	1.753725283	0.973	0.281	0	3	HLA-B
0	1.744150325	0.63	0.111	0	3	JUN
0	1.739968276	0.281	0.001	0	3	GZMK
0	1.724822867	0.782	0.078	0	3	ARHGDIB

0	1.667072173	0.888	0.232	0	3	BTG1
0	1.649874333	0.739	0.058	0	3	SRGN
0	1.584368787	0.493	0.002	0	3	CD7
0	1.564345304	0.396	0.002	0	3	PRF1
0	1.544913798	0.883	0.182	0	3	HLA-E
0	1.543829376	0.657	0.053	0	3	EVL
0	1.5366608	0.442	0.058	0	3	LTB
0	1.526841651	0.65	0.152	0	3	JUNB
0	1.512727154	0.301	0.001	0	3	KLRD1
0	1.508504556	0.999	0.352	0	3	MALAT1
0	1.472337667	0.52	0.002	0	3	CD3G
0	1.462044801	0.49	0.015	0	3	ITM2A
0	1.460713579	0.552	0.018	0	3	GIMAP7
0	1.42521805	0.619	0.049	0	3	RAC2
0	1.39814288	0.541	0.006	0	3	LCK
0	1.390409196	0.662	0.151	0	3	ZFP36L2
0	1.387023235	0.665	0.062	0	3	GMFG
0	1.371753238	0.482	0.003	0	3	CD247
0	1.355655376	0.612	0.072	0	3	EMP3
0	1.354020483	0.438	0.023	0	3	CD69
0	1.351694681	0.526	0.021	0	3	GIMAP4
0	1.342505294	0.558	0.054	0	3	FYB1
0	1.329105561	0.521	0.053	0	3	CXCR4
0	1.323967515	0.549	0.036	0	3	ACAP1
0	1.323740393	0.446	0.034	0	3	ANXA1
0	1.318677834	0.525	0.141	0	3	JUND
0	1.30673856	0.507	0.033	0	3	CLEC2D
0	1.30089344	0.344	0.002	0	3	CTSW
0	1.297262184	0.508	0.022	0	3	IL2RG
0	1.28277595	0.585	0.062	0	3	CD48
0	1.27193009	0.294	0.001	0	3	TRGC2
0	1.262914777	0.538	0.059	0	3	FXYD5
0	1.25190686	0.455	0.014	0	3	ARL4C
0	1.24896058	0.543	0.045	0	3	ITGB2
0	1.247769498	0.35	0.013	0	3	DUSP2
0	1.238507103	0.551	0.057	0	3	LIMD2
0	1.220421061	0.515	0.104	0	3	TSC22D3
0	1.206569434	0.792	0.258	0	3	TXNIP
0	1.20517529	0.548	0.061	0	3	STK4
0	1.202043314	0.453	0.05	0	3	KIAA1551

0	1.178033076	0.288	0.008	0	3	HOPX
0	1.17712004	0.538	0.054	0	3	CD53
0	1.175101273	0.382	0.002	0	3	GZMM
0	1.156124094	0.521	0.057	0	3	LSP1
0	1.154392066	0.48	0.036	0	3	IKZF1
0	1.136629445	0.263	0.001	0	3	CD8A
0	1.135963001	0.406	0.015	0	3	PTPN7
0	1.133723588	0.556	0.108	0	3	IFITM2
0	1.130896373	0.343	0.017	0	3	CD27
0	1.13051054	0.47	0.029	0	3	TBC1D10C
0	1.125701992	0.496	0.047	0	3	LCP1
0	1.124148938	0.572	0.115	0	3	IER2
0	1.123053283	0.564	0.103	0	3	CD99
0	1.118776418	0.42	0.098	0	3	HSPA1B
0	1.117678087	0.482	0.042	0	3	TRAF3IP3
0	1.111402978	0.475	0.101	0	3	DUSP1
0	1.103325695	0.492	0.051	0	3	HLA-F
0	1.095346283	0.575	0.104	0	3	PSMB9
0	1.09352194	0.875	0.441	0	3	SH3BGRL3
0	1.092180958	0.981	0.822	0	3	ACTB
0	1.088121623	0.453	0.049	0	3	ISG20
0	1.086794323	0.841	0.352	0	3	DDX5
0	1.046928781	0.599	0.162	0	3	SRSF7
0	1.040635079	0.357	0.018	0	3	APOBEC3G
0	1.040633367	0.545	0.073	0	3	LAPTM5
0	1.034975537	0.981	0.84	0	3	RPS3
0	1.028198596	0.454	0.048	0	3	GPSM3
0	1.021024382	0.358	0.017	0	3	SLFN5
0	1.014966554	0.859	0.45	0	3	ACTG1
0	1.014377952	0.402	0.028	0	3	CYTIP
0	1.013447958	0.395	0.051	0	3	DDIT4
0	1.012011842	0.278	0.001	0	3	TIGIT
0	1.009907679	0.399	0.035	0	3	CELF2
0	1.004712992	0.442	0.043	0	3	CD44
0	0.994133134	0.459	0.059	0	3	IFI16
0	0.989440268	0.357	0.015	0	3	BIN2
0	0.988122278	0.405	0.069	0	3	FOS
0	0.983412466	0.348	0.006	0	3	LAT
0	0.97859035	0.569	0.14	0	3	ARL6IP5
0	0.978454492	0.436	0.068	0	3	IRF1

0	0.971342809	0.322	0.005	0	3	PYHIN1
0	0.971201986	0.633	0.171	0	3	N4BP2L2
0	0.968076327	0.393	0.046	0	3	STK17A
0	0.955840594	0.571	0.138	0	3	AES
0	0.955153471	0.394	0.045	0	3	PCSK7
0	0.954227614	0.579	0.141	0	3	PNISR
0	0.947664617	0.465	0.096	0	3	ANKRD12
0	0.944136651	0.363	0.059	0	3	KLF2
0	0.936303454	0.314	0.011	0	3	SPOCK2
0	0.934838615	0.448	0.093	0	3	PPP2R5C
0	0.931338274	0.459	0.082	0	3	RSRP1
0	0.930820936	0.396	0.038	0	3	BTN3A2
0	0.930476308	0.352	0.025	0	3	AKNA
0	0.924994527	0.387	0.04	0	3	STK17B
0	0.924122598	0.391	0.056	0	3	ATM
0	0.923039382	0.354	0.033	0	3	ETS1
0	0.919694647	0.4	0.04	0	3	GYPC
0	0.919603816	0.318	0.027	0	3	ALOX5AP
0	0.916918829	0.316	0.041	0	3	FOSB
0	0.911516428	0.344	0.022	0	3	CLEC2B
0	0.903946804	0.59	0.067	0	3	CD37
0	0.899285803	0.974	0.855	0	3	RPS2
0	0.898347933	0.41	0.063	0	3	PRPF38B
0	0.896140924	0.328	0.008	0	3	SKAP1
0	0.894087558	0.984	0.906	0	3	RPS19
0	0.889811476	0.319	0.016	0	3	SLA
0	0.884881342	0.312	0.027	0	3	IKZF3
0	0.874973977	0.366	0.092	0	3	DNAJB1
0	0.873059421	0.602	0.197	0	3	SEPT7
0	0.87115332	0.427	0.082	0	3	POLR2J3.1
0	0.869956211	0.282	0.009	0	3	GBP5
0	0.862532843	0.465	0.137	0	3	KLF6
0	0.860710376	0.473	0.104	0	3	PRRC2C
0	0.85851349	0.357	0.039	0	3	SEPT6
0	0.855174738	0.361	0.038	0	3	ARHGEF1
0	0.849345717	0.312	0.044	0	3	CYTOR
0	0.843603108	0.313	0.024	0	3	MYO1F
0	0.842082991	0.329	0.041	0	3	UCP2
0	0.840506677	0.307	0.013	0	3	DOK2
0	0.838242898	0.352	0.03	0	3	FMNL1

0	0.835876404	0.505	0.142	0	3	FUS
0	0.832690795	0.269	0.001	0	3	IL2RB
0	0.831902499	0.875	0.541	0	3	MYL12A
0	0.827374068	0.342	0.045	0	3	ABHD17A
0	0.826423498	0.365	0.051	0	3	CCND3
0	0.820552073	0.34	0.03	0	3	PPP1R18
0	0.814774035	0.303	0.025	0	3	CHST12
0	0.809655845	0.396	0.07	0	3	NKTR
0	0.807523831	0.274	0.003	0	3	CD96
0	0.80724471	0.315	0.021	0	3	RHOH
0	0.80642842	0.344	0.038	0	3	HCLS1
0	0.802754558	0.316	0.027	0	3	SAMD9
0	0.802214291	0.987	0.874	0	3	RPL30
0	0.798476605	0.477	0.134	0	3	RNF213
0	0.798243164	0.99	0.915	0	3	RPL28
0	0.796884541	0.385	0.068	0	3	WIPF1
0	0.793214026	0.337	0.042	0	3	SUN2
0	0.787120306	0.416	0.085	0	3	ICAM3
0	0.784281602	0.302	0.063	0	3	H1FX
0	0.784136753	0.294	0.02	0	3	ITGA4
0	0.7837235	0.269	0.002	0	3	ZAP70
0	0.777166142	0.364	0.05	0	3	EVI2B
0	0.776789035	0.626	0.213	0	3	VIM
0	0.775074139	0.388	0.074	0	3	SEPT1
0	0.770827354	0.333	0.053	0	3	LBH
0	0.766607605	0.285	0.025	0	3	PIK3IP1
0	0.764733873	0.345	0.059	0	3	SEPT9
0	0.763078882	0.322	0.027	0	3	APBB1IP
0	0.759189686	0.292	0.024	0	3	ARHGAP30
0	0.757429428	0.991	0.957	0	3	RPS27
0	0.754978852	0.411	0.092	0	3	MBNL1
0	0.754861277	0.326	0.06	0	3	CD47
0	0.752006895	0.349	0.061	0	3	AKAP13
0	0.74941674	0.255	0.013	0	3	SIT1
0	0.7464624	0.376	0.073	0	3	FAM49B
0	0.741454506	0.265	0.011	0	3	GIMAP1
0	0.7412332	0.387	0.089	0	3	KMT2E
0	0.7385366	0.292	0.06	0	3	EFHD2
0	0.734841313	0.282	0.033	0	3	CARD16
0	0.734140322	0.439	0.124	0	3	ARGLU1

0	0.733143273	0.281	0.03	0	3	PIM1
0	0.730077286	0.27	0.01	0	3	LCP2
0	0.725240314	0.333	0.062	0	3	PDCD4
0	0.721852526	0.333	0.054	0	3	FNBP1
0	0.719083938	0.312	0.051	0	3	SMCHD1
0	0.718169093	0.266	0.015	0	3	EMB
0	0.711075077	0.28	0.039	0	3	DENND2D
0	0.705081117	0.305	0.054	0	3	RORA
0	0.69887533	0.275	0.053	0	3	BTG2
0	0.696330303	0.353	0.072	0	3	CCDC85B
0	0.696012998	0.288	0.048	0	3	KMT2A
0	0.694618691	0.351	0.071	0	3	CCNL1
0	0.694430528	0.341	0.065	0	3	ELF1
0	0.692334633	0.273	0.027	0	3	SLC38A1
0	0.685054463	0.254	0.021	0	3	ARHGAP15
0	0.684990687	0.301	0.045	0	3	DOCK8
0	0.680066146	0.266	0.022	0	3	IL16
0	0.675387844	0.317	0.06	0	3	ISG15
0	0.671578493	0.266	0.034	0	3	ANKRD44
0	0.671298782	0.332	0.066	0	3	ARID4B
0	0.669928555	0.326	0.071	0	3	AC245297.3
0	0.669906776	0.298	0.046	0	3	TGFB1
0	0.66888628	0.989	0.862	0	3	RPL39
0	0.667174208	0.274	0.03	0	3	RGS10
0	0.666416412	0.32	0.065	0	3	TNFAIP8
0	0.662929572	0.276	0.038	0	3	DGKZ
0	0.659365477	0.265	0.035	0	3	CNN2
0	0.656924023	0.286	0.047	0	3	RBL2
0	0.655105616	0.287	0.04	0	3	CSK
0	0.653449014	0.291	0.042	0	3	FLNA
0	0.648082938	0.357	0.085	0	3	TAPBP
0	0.63883896	0.274	0.037	0	3	TAP1
0	0.62504277	0.27	0.051	0	3	CLK1
0	0.619071011	0.967	0.816	0	3	RPL18
0	0.615919214	0.265	0.04	0	3	CYLD
0	0.613451664	0.983	0.718	0	3	TMSB10
0	0.611117164	0.334	0.076	0	3	MYH9
0	0.601419086	0.251	0.031	0	3	ARHGAP45
0	0.601144959	0.314	0.05	0	3	BST2
0	0.600159575	0.26	0.045	0	3	RIPOR2

0	0.58900867	0.984	0.944	0	3	RPS15A
0	0.573019344	0.261	0.046	0	3	KANSL1
0	0.339347628	0.516	0.149	0	3	HLA-DPB1
0	0.339211583	0.813	0.221	0	3	S100A6
1.01E-306	0.708547686	0.41	0.115	3.38E-302	3	PAXX
1.20E-304	0.631146316	0.358	0.089	4.04E-300	3	GNAI2
1.62E-304	0.611074708	0.29	0.06	5.43E-300	3	MYCBP2
4.36E-303	0.577563588	0.274	0.054	1.46E-298	3	EPC1
2.20E-301	0.681824921	0.36	0.091	7.39E-297	3	RBM25
2.90E-300	0.651175091	0.349	0.086	9.72E-296	3	PNN
1.93E-298	0.811172785	0.735	0.366	6.47E-294	3	ARPC2
1.79E-295	0.588545393	0.304	0.066	6.01E-291	3	IQGAP1
1.11E-294	0.585796651	0.293	0.062	3.71E-290	3	SH3KBP1
8.75E-294	0.596646966	0.304	0.067	2.93E-289	3	BPTF
1.80E-293	0.642889762	0.38	0.101	6.04E-289	3	LEPROTL1
2.52E-293	0.64564268	0.299	0.065	8.45E-289	3	MBP
3.89E-292	0.639284784	0.373	0.099	1.30E-287	3	SP100
6.11E-291	0.579070181	0.295	0.064	2.05E-286	3	PPP1R12A
4.68E-289	0.574193573	0.286	0.059	1.57E-284	3	AHNAK
3.94E-285	0.661236606	0.433	0.133	1.32E-280	3	PSMB8
4.50E-284	0.716763765	0.391	0.111	1.51E-279	3	GCC2
1.36E-277	0.469229174	0.989	0.979	4.55E-273	3	RPL13
1.39E-277	0.573784758	0.257	0.052	4.67E-273	3	ODF2L
1.67E-276	0.759997095	0.337	0.084	5.59E-272	3	LGALS1
2.62E-276	0.652226327	0.413	0.121	8.79E-272	3	SYNE2
2.36E-275	0.490590419	0.99	0.96	7.90E-271	3	RPS12
4.05E-275	0.451008644	0.99	0.97	1.36E-270	3	RPS27A
2.26E-270	0.570426208	0.271	0.058	7.58E-266	3	SP110
2.04E-266	0.605779412	0.39	0.115	6.85E-262	3	TERF2IP
2.42E-265	0.664602356	0.418	0.131	8.13E-261	3	JAK1
2.40E-264	1.183495132	0.408	0.131	8.05E-260	3	HSPA1A
6.52E-264	0.635075155	0.331	0.086	2.19E-259	3	ANXA6
6.36E-263	0.598239959	0.307	0.075	2.13E-258	3	TPR
1.07E-262	0.603497167	0.922	0.701	3.58E-258	3	RPSA
1.84E-262	0.63469202	0.387	0.114	6.18E-258	3	MPHOSPH8
1.32E-258	0.607980292	0.328	0.085	4.43E-254	3	HMGB2
2.47E-255	0.571437532	0.385	0.111	8.29E-251	3	AAK1
1.57E-253	0.568696023	0.286	0.067	5.25E-249	3	MACF1
6.77E-253	0.626174644	0.329	0.089	2.27E-248	3	BRD2
2.57E-252	0.554882159	0.269	0.061	8.62E-248	3	RSBN1L

2.87E-249	0.613339553	0.408	0.128	9.63E-245	3	LUC7L3
4.54E-249	0.611114513	0.988	0.885	1.52E-244	3	RPS29
1.34E-247	0.597850251	0.283	0.068	4.48E-243	3	SLC38A2
1.97E-245	0.525827427	0.981	0.966	6.60E-241	3	RPS18
2.64E-240	0.537807511	0.271	0.064	8.85E-236	3	ROCK1
3.08E-239	0.520124264	0.255	0.057	1.03E-234	3	IDS
2.31E-238	0.459238522	0.265	0.061	7.75E-234	3	WNK1
8.39E-238	0.54785948	0.281	0.069	2.81E-233	3	ARID1B
5.76E-237	0.523452101	0.256	0.058	1.93E-232	3	BOD1L1
6.97E-235	0.636698073	0.541	0.219	2.34E-230	3	RBM39
1.05E-234	0.544228752	0.306	0.08	3.54E-230	3	CASP4
4.14E-233	0.57867596	0.346	0.101	1.39E-228	3	ATRX
1.03E-231	0.605331799	0.368	0.114	3.45E-227	3	SF1
4.64E-231	0.539591116	0.305	0.081	1.56E-226	3	DHRS7
2.02E-230	0.549399195	0.334	0.096	6.79E-226	3	NCOR1
2.16E-228	0.556285541	0.264	0.062	7.25E-224	3	PTPN6
4.18E-228	0.490807936	0.252	0.058	1.40E-223	3	VPS13C
1.01E-226	0.661912971	0.636	0.304	3.38E-222	3	SARAF
1.43E-226	0.661114704	0.475	0.179	4.78E-222	3	DDX17
9.08E-223	0.524647297	0.932	0.723	3.04E-218	3	RPS21
7.55E-218	0.546879643	0.295	0.08	2.53E-213	3	DDX6
1.02E-214	0.478433313	0.972	0.915	3.42E-210	3	RPL18A
4.79E-213	0.467985865	0.251	0.061	1.60E-208	3	TMEM259
5.24E-213	0.578899022	0.291	0.08	1.76E-208	3	ANKRD11
4.02E-211	0.583591794	0.354	0.11	1.35E-206	3	CKLF
2.10E-210	0.905136169	0.53	0.24	7.06E-206	3	ID2
9.79E-208	0.579216474	0.382	0.13	3.28E-203	3	SF3B1
2.88E-207	0.506014716	0.301	0.086	9.66E-203	3	ORMDL1
3.80E-207	0.738948607	0.973	0.848	1.27E-202	3	MT-ATP6
2.10E-204	0.507311572	0.276	0.075	7.03E-200	3	NSD3
6.45E-204	0.571413543	0.359	0.118	2.16E-199	3	MCL1
9.28E-201	0.599171677	0.346	0.111	3.11E-196	3	ZFP36
1.77E-198	0.539502968	0.34	0.109	5.94E-194	3	BUB3
1.96E-198	0.595341221	0.439	0.167	6.56E-194	3	ARPC1B
3.07E-193	0.586295392	0.483	0.198	1.03E-188	3	CAP1
7.40E-193	0.50232038	0.285	0.082	2.48E-188	3	OGA
1.33E-186	0.456960534	0.276	0.078	4.46E-182	3	RNASET2
3.29E-186	0.577574522	0.583	0.277	1.10E-181	3	SSR2
3.29E-183	0.515590612	0.335	0.11	1.10E-178	3	LRRFIP1
6.18E-182	0.547874653	0.45	0.182	2.07E-177	3	SRRM2

3.88E-179	0.62273146	0.947	0.816	1.30E-174	3	MT-ND3
1.18E-178	0.595844312	0.455	0.189	3.94E-174	3	ACTR3
2.90E-178	0.546791748	0.308	0.099	9.74E-174	3	OSTF1
7.85E-178	0.609682366	0.372	0.137	2.63E-173	3	DDX3X
4.48E-177	0.504486074	0.365	0.129	1.50E-172	3	YPEL3
3.65E-175	0.49306657	0.253	0.071	1.22E-170	3	PPP1R10
1.07E-174	0.553321416	0.411	0.161	3.59E-170	3	SRSF11
1.14E-174	0.498339999	0.966	0.834	3.83E-170	3	MT-CYB
1.50E-172	0.368877181	0.977	0.901	5.04E-168	3	FAU
1.23E-170	0.543981318	0.573	0.277	4.14E-166	3	SON
1.30E-168	0.449829478	0.282	0.086	4.36E-164	3	ADAR
2.48E-168	0.441677576	0.978	0.96	8.30E-164	3	RPS14
3.63E-168	0.4855168	0.319	0.106	1.22E-163	3	AKAP9
1.41E-167	0.530979925	0.854	0.612	4.74E-163	3	H3F3B
4.19E-167	0.553813929	0.39	0.153	1.41E-162	3	CFLAR
9.67E-166	0.466173358	0.267	0.081	3.24E-161	3	CCNDBP1
3.49E-165	0.549594359	0.367	0.136	1.17E-160	3	HNRNPH1
5.54E-162	0.39019686	0.301	0.097	1.86E-157	3	CD81
8.49E-162	0.516842017	0.401	0.157	2.85E-157	3	CDC42SE2
9.12E-162	0.453167405	0.28	0.088	3.06E-157	3	BCLAF1
1.44E-161	0.455262742	0.254	0.075	4.83E-157	3	ASH1L
6.77E-161	0.562769059	0.472	0.21	2.27E-156	3	HNRNPU
1.06E-158	0.421026571	0.988	0.98	3.55E-154	3	RPL10
8.03E-157	0.512607637	0.489	0.223	2.69E-152	3	HNRNPA3
1.63E-156	0.462047192	0.253	0.076	5.46E-152	3	PSMA3-AS1
1.75E-156	0.444539259	0.967	0.916	5.86E-152	3	RPL23A
2.37E-155	0.526832967	0.942	0.792	7.95E-151	3	MT-ND1
4.88E-154	0.472630853	0.338	0.124	1.64E-149	3	GPBP1
2.64E-153	0.523201798	0.503	0.238	8.87E-149	3	GNAS
8.68E-153	0.442351805	0.276	0.089	2.91E-148	3	PSIP1
1.24E-152	0.488035838	0.393	0.16	4.17E-148	3	ANP32B
2.08E-152	0.469500646	0.332	0.122	6.98E-148	3	TAF7
2.41E-151	0.691443761	0.461	0.216	8.08E-147	3	ZFP36L1
1.07E-150	0.406322996	0.996	0.899	3.60E-146	3	MT-CO1
7.42E-150	0.532581709	0.334	0.123	2.49E-145	3	ITGB1
1.50E-149	0.457082539	0.386	0.154	5.02E-145	3	TMEM50A
1.06E-145	0.34127555	0.974	0.894	3.56E-141	3	PTMA
1.67E-145	0.530392079	0.494	0.235	5.60E-141	3	TPM3
2.05E-144	0.47552647	0.381	0.155	6.86E-140	3	ACTR2
6.93E-144	0.46713642	0.98	0.941	2.32E-139	3	RPLP2

5.99E-143	0.515388346	0.428	0.188	2.01E-138	3	CTSC
2.06E-142	0.474019522	0.313	0.113	6.91E-138	3	VMP1
1.15E-141	0.482372033	0.426	0.188	3.85E-137	3	SRSF2
7.36E-141	0.506361889	0.256	0.083	2.47E-136	3	STAT1
3.22E-140	0.526818369	0.285	0.1	1.08E-135	3	XBP1
9.48E-138	0.419952339	0.26	0.086	3.18E-133	3	ZRANB2
1.84E-137	0.470311033	0.349	0.139	6.19E-133	3	PPP1R2
4.79E-137	0.495208907	0.481	0.231	1.61E-132	3	VAMP2
4.46E-136	0.416381136	0.864	0.648	1.49E-131	3	MT-ND5
1.77E-135	0.528121702	0.463	0.223	5.94E-131	3	RAP1B
9.03E-135	0.466310768	0.33	0.129	3.03E-130	3	ARPC5L
1.50E-134	0.47917377	0.458	0.214	5.04E-130	3	YWHAZ
7.63E-133	0.443798095	0.326	0.127	2.56E-128	3	DDX46
3.77E-131	0.445510385	0.252	0.085	1.26E-126	3	STAT3
1.43E-130	0.463152306	0.271	0.095	4.79E-126	3	TMEM123
1.74E-130	0.50628869	0.595	0.334	5.84E-126	3	PNRC1
2.72E-130	0.392241443	0.994	0.91	9.12E-126	3	MT-CO2
1.13E-128	0.486591303	0.965	0.912	3.78E-124	3	RPL27A
5.05E-128	0.466843026	0.307	0.117	1.69E-123	3	TNRC6B
7.13E-128	0.374833646	0.274	0.095	2.39E-123	3	TUBA1A
5.24E-126	0.45675403	0.838	0.634	1.76E-121	3	UBC
1.06E-125	0.469247834	0.537	0.281	3.55E-121	3	SRSF5
1.31E-124	0.463214676	0.719	0.471	4.39E-120	3	HNRNPA2B1
8.06E-122	0.561578034	0.338	0.143	2.70E-117	3	LITAF
2.83E-121	0.444687886	0.257	0.092	9.51E-117	3	DHX36
5.57E-120	0.493228702	0.637	0.377	1.87E-115	3	NOP53
7.08E-120	0.490807573	0.447	0.22	2.37E-115	3	PTP4A2
1.86E-119	0.432431917	0.348	0.147	6.25E-115	3	SCAF11
6.22E-119	0.455056872	0.457	0.223	2.09E-114	3	LY6E
3.30E-117	0.420497781	0.836	0.564	1.11E-112	3	CYBA
1.83E-116	0.408062197	0.264	0.097	6.13E-112	3	SRSF4
8.15E-116	0.426037425	0.341	0.145	2.73E-111	3	SFPQ
1.48E-115	0.297193691	0.981	0.948	4.97E-111	3	RPS3A
4.14E-115	0.444985214	0.788	0.552	1.39E-110	3	HSP90AA1
8.57E-115	0.318710701	0.502	0.243	2.87E-110	3	MTRNR2L12
1.24E-114	0.402364255	0.371	0.165	4.17E-110	3	MSN
2.83E-113	0.503225348	0.647	0.398	9.50E-109	3	PABPC1
2.36E-112	0.400043362	0.343	0.145	7.91E-108	3	TUBA4A
1.07E-111	0.427598168	0.431	0.208	3.60E-107	3	ARPC5
1.28E-110	0.417613206	0.28	0.109	4.28E-106	3	TRA2B

1.40E-110	0.386504	0.297	0.119	4.70E-106	3	DPP7
5.24E-110	0.40154033	0.285	0.113	1.76E-105	3	EIF3A
1.40E-109	0.322084403	0.264	0.098	4.70E-105	3	WSB1
3.56E-105	0.349242808	0.256	0.096	1.19E-100	3	U2SURP
3.69E-104	0.331539477	0.989	0.901	1.24E-99	3	MT-CO3
3.86E-104	0.396755111	0.333	0.147	1.29E-99	3	LSM8
1.07E-103	0.4092611	0.265	0.104	3.58E-99	3	ZNF207
2.31E-103	0.420128643	0.304	0.127	7.74E-99	3	CAPZA1
9.30E-103	0.422820257	0.488	0.259	3.12E-98	3	DDX24
1.97E-102	0.438139241	0.364	0.169	6.60E-98	3	DEK
1.07E-101	0.437711206	0.41	0.205	3.58E-97	3	ARF6
2.78E-101	0.393996624	0.798	0.589	9.33E-97	3	HMGB1
9.11E-100	0.371420372	0.293	0.123	3.06E-95	3	HNRNPR
1.94E-96	0.277900015	0.979	0.865	6.49E-92	3	MT-ND4
1.24E-93	0.325748584	0.268	0.11	4.17E-89	3	NAP1L4
1.97E-93	0.32253342	0.253	0.1	6.59E-89	3	WASF2
8.41E-92	0.346668422	0.381	0.181	2.82E-87	3	FYN
3.20E-91	0.364244458	0.252	0.103	1.07E-86	3	RNPS1
7.61E-90	0.392730641	0.467	0.255	2.55E-85	3	C12orf57
1.14E-89	0.319591385	0.263	0.109	3.82E-85	3	TLN1
1.34E-89	0.354926216	0.349	0.166	4.51E-85	3	UBE2I
1.67E-89	0.372549801	0.303	0.135	5.61E-85	3	HNRNPD
3.59E-88	0.311048959	0.253	0.103	1.20E-83	3	ANXA2
2.57E-87	0.365489489	0.275	0.119	8.63E-83	3	THRAP3
2.12E-86	0.354491194	0.371	0.184	7.12E-82	3	SNRPB
1.26E-85	0.326528019	0.284	0.125	4.21E-81	3	AIP
5.88E-84	0.355224953	0.473	0.265	1.97E-79	3	HNRNPF
1.01E-81	0.352517572	0.336	0.165	3.37E-77	3	PPIG
1.56E-80	0.364050688	0.48	0.273	5.24E-76	3	CNBP
9.25E-80	0.318126527	0.316	0.149	3.10E-75	3	MTDH
9.59E-80	0.350666557	0.35	0.174	3.22E-75	3	PDIA3
8.58E-78	0.344708595	0.276	0.125	2.88E-73	3	SRSF10
1.60E-76	0.328867946	0.283	0.13	5.38E-72	3	SLTM
2.16E-76	0.268893957	0.606	0.368	7.26E-72	3	SSR4
2.71E-75	0.322328377	0.279	0.129	9.08E-71	3	CDC37
9.00E-74	0.28815566	0.254	0.113	3.02E-69	3	SS18L2
9.05E-74	0.3196422	0.388	0.207	3.03E-69	3	ARF1
1.16E-73	0.342118167	0.398	0.215	3.89E-69	3	TCF25
3.33E-73	0.306007446	0.261	0.117	1.12E-68	3	PRPF40A
3.96E-72	0.285253839	0.99	0.977	1.33E-67	3	RPL32

1.97E-71	0.323887481	0.356	0.186	6.60E-67	3	XRCC5
3.07E-71	0.354800665	0.311	0.155	1.03E-66	3	HNRNPM
5.76E-71	0.37075654	0.682	0.475	1.93E-66	3	CLIC1
2.18E-70	0.346332986	0.423	0.235	7.32E-66	3	SRRM1
8.07E-69	0.320506079	0.432	0.244	2.71E-64	3	C9orf16
9.15E-69	0.293877533	0.9	0.755	3.07E-64	3	EEF1D
1.39E-67	0.356525026	0.831	0.606	4.65E-63	3	RPS10
1.54E-67	0.46118741	0.896	0.752	5.18E-63	3	PFN1
2.14E-67	0.292548595	0.267	0.126	7.18E-63	3	MAP2K2
1.14E-66	0.371410852	0.855	0.694	3.81E-62	3	CFL1
7.20E-66	0.299211137	0.326	0.168	2.41E-61	3	RSRC2
1.22E-63	0.292469854	0.252	0.119	4.08E-59	3	HNRNPH3
1.38E-63	0.295618922	0.307	0.156	4.62E-59	3	HP1BP3
3.10E-63	0.287008925	0.285	0.141	1.04E-58	3	TAF1D
4.95E-63	0.298874295	0.971	0.923	1.66E-58	3	RPS4X
6.90E-63	0.281825503	0.268	0.129	2.31E-58	3	TGOLN2
1.05E-62	0.263169689	0.487	0.289	3.52E-58	3	PPIB
9.91E-61	0.306498732	0.489	0.301	3.32E-56	3	SRSF3
1.74E-60	0.316548073	0.564	0.37	5.84E-56	3	YWHAB
1.91E-59	0.309898189	0.458	0.277	6.41E-55	3	CAPZB
4.59E-59	0.278889371	0.288	0.148	1.54E-54	3	SF3B2
5.85E-59	0.323762308	0.323	0.174	1.96E-54	3	ARPC4
4.53E-57	0.302872086	0.318	0.171	1.52E-52	3	RAP1A
6.40E-55	0.265552268	0.272	0.139	2.15E-50	3	PRMT2
1.21E-54	0.294175065	0.49	0.31	4.06E-50	3	EIF3F
9.24E-53	0.263596565	0.976	0.955	3.10E-48	3	RPL3
2.39E-51	0.308349814	0.356	0.209	8.01E-47	3	RBM3
3.77E-51	0.270947487	0.258	0.133	1.26E-46	3	P4HB
5.71E-51	0.254772878	0.368	0.213	1.92E-46	3	CALR
3.86E-49	0.268794618	0.475	0.302	1.29E-44	3	NCL
4.15E-43	0.267826271	0.471	0.311	1.39E-38	3	NAP1L1
7.70E-43	0.262411171	0.488	0.327	2.58E-38	3	HNRNPDL
1.00E-41	0.255289227	0.461	0.303	3.35E-37	3	PPP1CA
2.31E-30	0.300897658	0.962	0.939	7.75E-26	3	RPL21
3.92E-28	0.257219874	0.26	0.162	1.32E-23	3	NFKBIA
0	1.315446222	0.895	0.583	0	4	GSTP1
0	1.114858327	0.955	0.71	0	4	IL32
0	1.024583627	0.875	0.51	0	4	S100A11
0	0.841230335	0.946	0.724	0	4	TMSB10
0	0.760955458	0.961	0.859	0	4	MYL6

8.58E-263	1.066432151	0.642	0.288	2.88E-258	4	SOD2
1.14E-255	0.462357981	0.996	0.995	3.81E-251	4	EEF1A1
7.49E-254	0.684174424	0.254	0.047	2.51E-249	4	AL353751.1
6.24E-241	0.789008028	0.759	0.427	2.09E-236	4	C12orf75
4.04E-232	0.923212721	0.714	0.374	1.36E-227	4	KRT18
8.63E-225	0.825775409	0.66	0.32	2.90E-220	4	CARHSP1
2.18E-222	0.855054827	0.387	0.114	7.30E-218	4	SOX4
5.27E-222	0.794155719	0.734	0.399	1.77E-217	4	S100A10
2.63E-218	0.899993229	0.581	0.254	8.82E-214	4	TPM1
2.06E-215	0.806292139	0.7	0.36	6.90E-211	4	FABP3
1.05E-214	0.835893634	0.61	0.283	3.51E-210	4	ANXA4
7.55E-210	0.64432028	0.963	0.778	2.53E-205	4	CRYAB
1.64E-196	0.44209328	0.973	0.949	5.51E-192	4	RPL7
2.14E-191	0.546985796	0.893	0.754	7.17E-187	4	PFN1
2.02E-190	0.674365591	0.438	0.157	6.76E-186	4	TAX1BP3
4.06E-178	0.777904703	0.659	0.348	1.36E-173	4	KRT8
8.55E-177	0.71600214	0.528	0.225	2.87E-172	4	VIM
8.27E-174	0.744732983	0.633	0.334	2.77E-169	4	CNN3
8.76E-165	0.331072027	0.982	0.973	2.94E-160	4	RPL13A
5.84E-163	0.678187444	0.503	0.224	1.96E-158	4	TAGLN2
6.40E-161	0.704469739	0.46	0.191	2.15E-156	4	STMN1
4.81E-158	0.616678314	0.779	0.562	1.61E-153	4	HNRNPA1
5.87E-157	0.544960047	0.855	0.713	1.97E-152	4	MYL12B
4.39E-154	0.393796929	0.966	0.924	1.47E-149	4	RPL15
9.06E-153	0.35861136	0.964	0.939	3.04E-148	4	RPL8
9.07E-150	0.37446981	0.973	0.946	3.04E-145	4	RPL26
2.25E-149	0.346303497	0.977	0.955	7.53E-145	4	RPL3
1.66E-146	0.495638203	0.871	0.749	5.56E-142	4	PPIA
1.35E-144	0.612497212	0.508	0.231	4.53E-140	4	TCEAL9
6.90E-144	0.52909471	0.898	0.726	2.31E-139	4	HSPB1
2.66E-141	0.588939775	0.658	0.368	8.92E-137	4	CD24
3.42E-139	0.297738786	0.985	0.98	1.15E-134	4	RPL10
6.21E-138	0.451027153	0.908	0.808	2.08E-133	4	RPLP0
4.10E-137	0.662846364	0.767	0.569	1.38E-132	4	ENO1
6.76E-136	0.551378985	0.743	0.464	2.27E-131	4	ACTG1
3.45E-131	0.374496324	0.963	0.92	1.16E-126	4	RPL31
5.96E-128	0.377353404	0.976	0.952	2.00E-123	4	RPL12
2.39E-126	0.585961914	0.606	0.334	8.01E-122	4	S100A13
1.49E-122	0.405835232	0.932	0.732	5.00E-118	4	FXYD2
4.04E-122	0.524045761	0.651	0.363	1.35E-117	4	IFITM3

1.30E-118	0.558531433	0.663	0.409	4.36E-114	4	HSBP1
2.24E-118	0.520097137	0.765	0.553	7.50E-114	4	MYL12A
1.11E-117	0.370313149	0.954	0.897	3.72E-113	4	PTMA
4.50E-116	0.498919368	0.807	0.651	1.51E-111	4	YBX1
7.76E-115	0.286279094	0.98	0.968	2.60E-110	4	RPS6
5.04E-114	0.567502206	0.541	0.285	1.69E-109	4	HCFC1R1
1.85E-111	0.517787109	0.392	0.17	6.20E-107	4	NME1
2.27E-111	0.524505716	0.768	0.591	7.61E-107	4	NPM1
3.84E-110	0.489233346	0.788	0.631	1.29E-105	4	CALM2
4.41E-107	0.506601373	0.808	0.639	1.48E-102	4	UBC
2.20E-105	0.589808943	0.497	0.262	7.37E-101	4	CDK2AP2
3.60E-104	0.479942079	0.301	0.116	1.21E-99	4	NIPSNAP2
9.54E-104	0.488293249	0.684	0.445	3.20E-99	4	RHOC
1.65E-103	0.341541403	0.934	0.872	5.53E-99	4	RPL10A
1.99E-102	0.295970543	0.951	0.906	6.67E-98	4	RPS9
9.61E-102	0.514968037	0.716	0.485	3.22E-97	4	RARRES3
1.15E-101	0.486272007	0.259	0.093	3.87E-97	4	PLSCR1
1.25E-101	0.555085343	0.536	0.306	4.21E-97	4	FKBP1A
4.40E-101	0.291693417	0.962	0.917	1.48E-96	4	RPL18A
5.66E-101	0.3741809	0.89	0.717	1.90E-96	4	MIF
1.55E-99	0.490521952	0.342	0.146	5.20E-95	4	YWHAH
2.33E-99	0.277888541	0.977	0.954	7.83E-95	4	SERF2
4.08E-96	0.516206985	0.614	0.39	1.37E-91	4	PPA1
2.04E-95	0.532560868	0.414	0.203	6.83E-91	4	JPT1
4.80E-94	0.400770705	0.828	0.698	1.61E-89	4	CFL1
1.37E-93	0.492905845	0.611	0.381	4.58E-89	4	CSTB
3.90E-92	0.420631511	0.789	0.612	1.31E-87	4	RPS10
5.93E-92	0.289671177	0.957	0.923	1.99E-87	4	RPL14
2.61E-88	0.281389236	0.957	0.923	8.77E-84	4	RPL29
5.63E-88	0.320095697	0.926	0.834	1.89E-83	4	SRP14
5.72E-88	0.272253538	0.966	0.931	1.92E-83	4	RPS24
1.79E-84	0.41146658	0.783	0.62	6.02E-80	4	H3F3B
2.17E-84	0.402935261	0.836	0.676	7.28E-80	4	PPDPF
2.55E-84	0.275285819	0.965	0.939	8.54E-80	4	RPL21
1.04E-83	0.480697887	0.369	0.176	3.49E-79	4	AC245595.1
1.97E-83	0.424996688	0.751	0.582	6.62E-79	4	SUMO2
2.32E-82	0.472427089	0.428	0.225	7.77E-78	4	SDCBP
3.57E-82	0.262783881	0.977	0.951	1.20E-77	4	RPS8
4.48E-82	0.378458319	0.829	0.671	1.50E-77	4	PRDX5
5.88E-82	0.500690878	0.266	0.11	1.97E-77	4	PDLIM1

7.75E-81	0.468507074	0.597	0.39	2.60E-76	4	CAPZA2
8.38E-81	0.309462557	0.927	0.862	2.81E-76	4	RACK1
9.45E-80	0.487924051	0.427	0.227	3.17E-75	4	YBX3
1.54E-79	0.355732418	0.836	0.705	5.18E-75	4	UQCRH
3.67E-79	0.478543796	0.281	0.118	1.23E-74	4	RCAN1
6.21E-79	0.414131029	0.654	0.448	2.08E-74	4	CYCS
4.74E-78	0.492282164	0.257	0.105	1.59E-73	4	ANXA2
1.92E-77	0.398254512	0.26	0.106	6.43E-73	4	RRAS2
4.65E-77	0.471776348	0.471	0.272	1.56E-72	4	YWHAQ
5.61E-77	0.285840012	0.936	0.886	1.88E-72	4	RPL7A
5.75E-77	0.436617096	0.715	0.526	1.93E-72	4	HMGN3
9.98E-77	0.379971639	0.817	0.686	3.35E-72	4	BTF3
1.09E-75	0.455426869	0.398	0.21	3.66E-71	4	SRI
1.16E-74	0.41451974	0.647	0.438	3.88E-70	4	DYNLT1
7.95E-74	0.412748638	0.694	0.511	2.67E-69	4	PSME1
2.20E-73	0.343920624	0.838	0.723	7.39E-69	4	SKP1
3.58E-73	0.428853761	0.328	0.155	1.20E-68	4	TSC22D1
4.82E-72	0.287189184	0.926	0.866	1.62E-67	4	RPL5
2.41E-71	0.361627228	0.27	0.116	8.07E-67	4	MAP1LC3A
5.56E-71	0.398586497	0.573	0.359	1.86E-66	4	ARL2
9.20E-71	0.484918993	0.561	0.38	3.09E-66	4	RAN
2.86E-70	0.463452188	0.568	0.376	9.58E-66	4	EIF6
1.71E-69	0.427526733	0.651	0.44	5.72E-65	4	DUSP23
5.45E-68	0.38624729	0.647	0.463	1.83E-63	4	CLTA
6.53E-68	0.338738851	0.455	0.252	2.19E-63	4	S100A6
4.60E-66	0.342965312	0.716	0.517	1.54E-61	4	PRDX3
6.29E-66	0.373239466	0.64	0.442	2.11E-61	4	RPL17
8.32E-66	0.405501649	0.601	0.413	2.79E-61	4	TRAPPC1
1.18E-65	0.389836952	0.304	0.143	3.96E-61	4	S100A16
1.91E-65	0.404239475	0.661	0.479	6.41E-61	4	CLIC1
1.20E-63	0.314033042	0.852	0.721	4.01E-59	4	NDUFS5
1.22E-63	0.389450938	0.353	0.181	4.10E-59	4	HIF1A
1.38E-63	0.378312437	0.347	0.177	4.64E-59	4	EEF1E1
1.80E-63	0.313029201	0.897	0.789	6.04E-59	4	DBI
3.58E-61	0.293155348	0.898	0.791	1.20E-56	4	PRDX1
1.40E-60	0.403814405	0.368	0.2	4.68E-56	4	MEAF6
1.12E-59	0.414648904	0.542	0.358	3.77E-55	4	SNRPE
2.77E-59	0.368763833	0.304	0.151	9.29E-55	4	PAIP1
2.85E-59	0.365734093	0.381	0.206	9.57E-55	4	RBPMS
5.21E-59	0.411372359	0.462	0.276	1.75E-54	4	ALDH1A1

7.92E-59	0.371555953	0.466	0.277	2.65E-54	4	LYPLAL1
1.44E-58	0.346284219	0.743	0.603	4.84E-54	4	SUB1
1.68E-58	0.286083867	0.85	0.755	5.63E-54	4	RPL4
2.51E-58	0.355397298	0.617	0.42	8.43E-54	4	BEX3
2.66E-58	0.346275113	0.294	0.143	8.93E-54	4	SLC40A1
2.89E-58	0.279982664	0.855	0.72	9.70E-54	4	POLR2L
3.49E-58	0.290207657	0.813	0.646	1.17E-53	4	DSTN
1.11E-57	0.338457706	0.706	0.536	3.74E-53	4	SNRPD2
1.96E-55	0.381655373	0.312	0.159	6.56E-51	4	SPX
3.43E-55	0.384798342	0.424	0.247	1.15E-50	4	LACTB2
9.66E-55	0.342906799	0.351	0.188	3.24E-50	4	RAB13
2.22E-54	0.25875995	0.897	0.782	7.43E-50	4	RPS27L
2.72E-53	0.316390912	0.725	0.55	9.12E-49	4	RPS17
3.28E-53	0.34019032	0.277	0.137	1.10E-48	4	RFK
6.70E-53	0.341495392	0.688	0.534	2.25E-48	4	MORF4L1
1.39E-52	0.287423373	0.824	0.708	4.65E-48	4	OST4
3.13E-52	0.267104476	0.808	0.61	1.05E-47	4	DAB2
3.33E-52	0.349820079	0.419	0.248	1.12E-47	4	HIGD1A
6.08E-52	0.374069282	0.305	0.158	2.04E-47	4	SELENOM
9.55E-52	0.340758483	0.59	0.421	3.20E-47	4	ERH
1.81E-51	0.403977976	0.375	0.215	6.08E-47	4	PLIN2
4.35E-51	0.366809168	0.522	0.351	1.46E-46	4	PSME2
6.96E-51	0.26488856	0.988	0.981	2.33E-46	4	FTH1
8.12E-51	0.367148294	0.416	0.251	2.72E-46	4	MORF4L2
2.08E-50	0.370019413	0.437	0.273	6.96E-46	4	RHEB
3.17E-50	0.34273549	0.338	0.184	1.06E-45	4	AIF1L
3.42E-50	0.313908183	0.257	0.125	1.15E-45	4	TRIP6
4.93E-50	0.330520301	0.694	0.534	1.65E-45	4	RAC1
3.95E-49	0.365455305	0.625	0.479	1.32E-44	4	HMGN1
4.57E-49	0.312043155	0.405	0.234	1.53E-44	4	PPA2
8.27E-49	0.340348242	0.646	0.498	2.77E-44	4	SNX3
1.70E-48	0.361089265	0.386	0.228	5.69E-44	4	ANXA5
3.06E-47	0.338799513	0.42	0.258	1.03E-42	4	RAB11A
3.41E-47	0.341572921	0.627	0.472	1.15E-42	4	RPL36A
2.25E-46	0.326368241	0.535	0.354	7.55E-42	4	AKR1C3
3.40E-46	0.331626669	0.509	0.341	1.14E-41	4	VPS29
1.20E-45	0.307274224	0.259	0.13	4.03E-41	4	BICC1
4.06E-45	0.267016515	0.817	0.699	1.36E-40	4	TPI1
3.75E-44	0.316502108	0.685	0.551	1.26E-39	4	EIF3E
5.60E-44	0.343081342	0.637	0.494	1.88E-39	4	RPS4Y1

2.81E-43	0.339004612	0.496	0.335	9.43E-39	4	MRPL33
4.73E-43	0.308106956	0.263	0.137	1.59E-38	4	UBE2A
1.39E-42	0.268658104	0.946	0.801	4.65E-38	4	MT1E
6.30E-42	0.345009824	0.426	0.275	2.11E-37	4	NHP2
1.36E-41	0.358399532	0.349	0.212	4.57E-37	4	RBM3
1.11E-40	0.294035987	0.315	0.178	3.71E-36	4	ARPC1B
1.24E-40	0.281151444	0.42	0.26	4.15E-36	4	CALD1
3.62E-40	0.292824001	0.713	0.597	1.21E-35	4	SLC25A3
4.17E-40	0.304344239	0.318	0.184	1.40E-35	4	APEX1
4.97E-40	0.288222074	0.724	0.612	1.67E-35	4	ATP6V1G1
1.28E-39	0.275936666	0.253	0.133	4.31E-35	4	NUTF2
1.36E-39	0.324975779	0.512	0.36	4.57E-35	4	SF3B6
1.82E-39	0.271311505	0.705	0.563	6.10E-35	4	POMP
2.62E-39	0.281772318	0.535	0.366	8.79E-35	4	SPATS2L
3.72E-39	0.278252574	0.668	0.525	1.25E-34	4	MINOS1
3.92E-39	0.307866991	0.408	0.257	1.32E-34	4	SCOC
5.29E-39	0.290507432	0.252	0.133	1.77E-34	4	LMNA
1.03E-38	0.325713876	0.562	0.421	3.46E-34	4	SRP9
1.05E-38	0.326676252	0.3	0.172	3.51E-34	4	CAPG
2.64E-38	0.308807205	0.396	0.248	8.86E-34	4	RHOB
1.38E-37	0.380206479	0.389	0.256	4.64E-33	4	TUBB
2.69E-37	0.27012143	0.42	0.264	9.04E-33	4	CETN2
3.12E-37	0.308462443	0.621	0.496	1.05E-32	4	CCNI
3.21E-37	0.283420709	0.566	0.414	1.08E-32	4	SUMO1
4.08E-37	0.336137557	0.492	0.349	1.37E-32	4	C11orf58
4.30E-37	0.294543313	0.626	0.476	1.44E-32	4	YWHAE
6.85E-37	0.312878014	0.446	0.302	2.30E-32	4	SQSTM1
7.33E-37	0.276356761	0.363	0.223	2.46E-32	4	TSPO
2.44E-36	0.307912676	0.332	0.202	8.17E-32	4	PCNP
3.48E-36	0.285194767	0.409	0.265	1.17E-31	4	PSMA2
3.59E-36	0.28072011	0.28	0.158	1.21E-31	4	MAGED2
1.48E-35	0.268194532	0.325	0.194	4.97E-31	4	MRPL47
1.76E-35	0.271641153	0.276	0.156	5.92E-31	4	HNRNPH2
1.89E-35	0.289174998	0.471	0.325	6.33E-31	4	PSMA4
3.39E-35	0.318846677	0.382	0.243	1.14E-30	4	DYNC1I2
5.40E-35	0.292184273	0.305	0.18	1.81E-30	4	CHMP4B
8.16E-35	0.294651131	0.292	0.171	2.74E-30	4	DDAH2
1.58E-34	0.250017707	0.267	0.15	5.30E-30	4	CCDC124
2.42E-34	0.257272273	0.653	0.509	8.11E-30	4	PRDX6
3.05E-34	0.256714876	0.282	0.16	1.02E-29	4	GNG12

4.14E-34	0.282232744	0.312	0.186	1.39E-29	4	BEX4
6.12E-34	0.254107899	0.294	0.171	2.05E-29	4	BTF3L4
1.22E-33	0.274899837	0.616	0.476	4.08E-29	4	SNRPG
1.46E-33	0.30878852	0.39	0.26	4.88E-29	4	MRFAP1
4.33E-33	0.278034442	0.344	0.212	1.45E-28	4	COMMD8
7.88E-33	0.298954114	0.47	0.335	2.64E-28	4	EIF4G2
1.08E-32	0.285469449	0.438	0.298	3.62E-28	4	RAB7A
2.89E-32	0.28237346	0.327	0.203	9.70E-28	4	TBCB
4.66E-32	0.254438763	0.734	0.621	1.56E-27	4	DYNLL1
6.27E-32	0.258585126	0.491	0.344	2.10E-27	4	SMS
6.90E-32	0.289849533	0.347	0.224	2.32E-27	4	SNRPD1
1.81E-31	0.280214058	0.34	0.217	6.08E-27	4	SSB
2.46E-31	0.27147812	0.322	0.198	8.24E-27	4	PDHA1
2.84E-31	0.250610503	0.261	0.149	9.54E-27	4	CTTN
2.89E-31	0.272799385	0.361	0.231	9.71E-27	4	CCNG1
6.20E-31	0.291034278	0.325	0.204	2.08E-26	4	RAB18
1.40E-30	0.273677733	0.479	0.346	4.70E-26	4	PTGES3
1.68E-30	0.263313066	0.521	0.389	5.62E-26	4	EID1
2.52E-30	0.268240329	0.49	0.35	8.44E-26	4	C1QBP
2.23E-29	0.276733892	0.599	0.48	7.49E-25	4	NOP10
2.38E-29	0.251707412	0.437	0.303	7.99E-25	4	EIF2S2
3.49E-29	0.269454614	0.491	0.357	1.17E-24	4	ABRACL
3.86E-29	0.257494471	0.323	0.203	1.29E-24	4	FKBP3
8.80E-29	0.258048728	0.252	0.147	2.95E-24	4	APOPT1
1.22E-28	0.261521568	0.505	0.374	4.10E-24	4	SSBP1
1.62E-28	0.261172247	0.306	0.192	5.43E-24	4	ANXA11
2.21E-27	0.27638495	0.302	0.191	7.43E-23	4	SNRPB
2.56E-27	0.264696675	0.476	0.352	8.59E-23	4	VAPA
2.79E-27	0.259851938	0.354	0.233	9.35E-23	4	EIF3J
2.87E-27	0.250968654	0.346	0.228	9.63E-23	4	RANBP1
3.63E-27	0.25513521	0.373	0.252	1.22E-22	4	MZT2A
6.37E-27	0.251278533	0.35	0.231	2.14E-22	4	MRPL13
9.62E-27	0.281068349	0.36	0.246	3.23E-22	4	EIF3M
2.87E-26	0.258930736	0.265	0.163	9.63E-22	4	MCTS1
6.40E-26	0.268122345	0.304	0.197	2.15E-21	4	EIF4A1
1.16E-25	0.261894913	0.444	0.322	3.88E-21	4	RSL24D1
4.29E-25	0.257786785	0.362	0.248	1.44E-20	4	TCEAL8
5.48E-25	0.267721467	0.447	0.333	1.84E-20	4	SERBP1
7.97E-25	0.254624895	0.444	0.326	2.67E-20	4	TMEM230
5.43E-24	0.253434138	0.316	0.211	1.82E-19	4	SNRPC

3.58E-23	0.270547683	0.356	0.247	1.20E-18	4	TPM3
6.66E-23	0.269238549	0.451	0.344	2.23E-18	4	SET
9.70E-23	0.250677233	0.434	0.319	3.25E-18	4	EIF3L
1.08E-22	0.25602389	0.358	0.25	3.64E-18	4	C6orf48
2.68E-22	0.261315529	0.3	0.203	8.97E-18	4	ACTR3
1.49E-20	0.254823789	0.309	0.213	4.99E-16	4	CCT8
0	2.735377397	0.911	0.177	0	5	NEAT1
0	2.016693731	0.938	0.371	0	5	MALAT1
0	1.271141228	0.356	0.056	0	5	SLC22A7
0	1.233235229	0.616	0.102	0	5	VMP1
0	1.225561267	0.331	0.005	0	5	ABHD14A-ACY1
0	1.185542327	0.434	0.031	0	5	KCNQ1OT1
0	1.179657567	0.531	0.112	0	5	ELF3
0	1.148687087	0.537	0.085	0	5	POLR2J3.1
0	1.086794793	0.737	0.236	0	5	ACSM2A
0	1.05288654	0.541	0.108	0	5	RASSF4
0	1.045369413	0.451	0.068	0	5	KIF12
0	1.041861338	0.61	0.117	0	5	SYNE2
0	1.03093512	0.77	0.287	0	5	ACSM2B
0	1.012751048	0.652	0.206	0	5	CUBN
0	0.938269924	0.642	0.188	0	5	LRP2
0	0.937003037	0.375	0.031	0	5	UNC5CL
0	0.935172167	0.485	0.109	0	5	LINC01320
0	0.930879616	0.525	0.116	0	5	PAX8
0	0.923398659	0.624	0.183	0	5	N4BP2L2
0	0.919657995	0.639	0.208	0	5	ZBTB20
0	0.91874444	0.43	0.07	0	5	MUC20-OT1
0	0.890702447	0.416	0.073	0	5	SLC47A2
0	0.876145053	0.364	0.046	0	5	HOOK2
0	0.810900885	0.318	0.021	0	5	PKHD1
0	0.786317227	0.309	0.027	0	5	SORBS2
0	0.740435483	0.357	0.062	0	5	IL17RB
0	0.686595745	0.282	0.028	0	5	TRPM3
0	0.67755496	0.297	0.043	0	5	SLC47A1
0	0.642347881	0.294	0.041	0	5	SLC17A1
0	0.632368565	0.285	0.037	0	5	VPS13A
0	0.624648999	0.29	0.043	0	5	PATJ
0	0.592756265	0.251	0.028	0	5	ARHGEF28
0	0.583169748	0.251	0.029	0	5	PAX2
3.65E-306	0.711986833	0.317	0.055	1.22E-301	5	PPFIBP1

8.26E-303	0.725853493	0.418	0.094	2.77E-298	5	WSB1
1.78E-298	0.749588476	0.489	0.125	5.98E-294	5	FARP1
4.25E-296	0.5919906	0.284	0.046	1.43E-291	5	FTX
3.46E-293	0.62805278	0.332	0.061	1.16E-288	5	NRP1
1.54E-286	0.78650014	0.537	0.153	5.18E-282	5	PNISR
3.55E-268	0.610728415	0.281	0.049	1.19E-263	5	GPR155
8.63E-268	0.841124341	0.7	0.266	2.89E-263	5	BHMT2
1.45E-249	0.738492525	0.536	0.166	4.85E-245	5	SLC13A3
1.97E-247	0.906392913	0.468	0.135	6.60E-243	5	LINC00671
5.23E-237	0.632767218	0.354	0.082	1.75E-232	5	ARHGAP29
1.06E-225	0.603750184	0.39	0.1	3.57E-221	5	MYO6
9.07E-225	0.813643665	0.369	0.093	3.04E-220	5	INSR
7.08E-219	0.911320612	0.454	0.144	2.37E-214	5	RNF213
6.90E-218	0.565679885	0.26	0.05	2.31E-213	5	SLC25A37
1.31E-202	0.612027089	0.306	0.07	4.38E-198	5	ITGB8
2.09E-202	0.604564076	0.268	0.056	7.02E-198	5	MST1
4.31E-202	0.72676115	0.592	0.223	1.44E-197	5	AQP1
6.91E-192	0.755161177	0.43	0.136	2.32E-187	5	SLC5A12
1.79E-190	0.752862391	0.714	0.344	6.00E-186	5	TNFSF10
2.38E-190	0.694118873	0.97	0.768	7.98E-186	5	SPP1
3.41E-190	0.501586366	0.262	0.057	1.14E-185	5	ZKSCAN1
1.70E-186	0.551124042	0.324	0.084	5.69E-182	5	PABPN1
2.93E-186	0.624719033	0.463	0.152	9.84E-182	5	SLC22A2
5.36E-175	0.5312704448	0.3	0.076	1.80E-170	5	MIA2
1.09E-173	0.523957587	0.252	0.057	3.66E-169	5	RBM6
4.38E-173	0.527236404	0.317	0.083	1.47E-168	5	GDA
6.47E-171	0.483321288	0.253	0.057	2.17E-166	5	ANK3
1.34E-170	0.567428826	0.504	0.184	4.51E-166	5	DDX17
1.42E-165	0.688057966	0.292	0.077	4.76E-161	5	CYP4A11
2.81E-165	0.586520642	0.405	0.131	9.42E-161	5	RETREG1
7.57E-161	0.556081946	0.347	0.104	2.54E-156	5	CHD9
1.39E-156	0.64939121	0.503	0.188	4.66E-152	5	PIGR
1.05E-150	0.551043128	0.393	0.13	3.53E-146	5	CDH6
8.49E-150	0.472470697	0.286	0.077	2.85E-145	5	CHDH
1.58E-146	0.520969001	0.404	0.137	5.29E-142	5	BCAM
8.72E-146	0.47578275	0.275	0.074	2.93E-141	5	FNIP2
2.98E-145	0.535188798	0.57	0.236	9.98E-141	5	AMN
1.53E-142	0.407188375	0.252	0.064	5.12E-138	5	CTNND1
1.12E-140	0.670502131	0.392	0.141	3.75E-136	5	HNRNPH1
1.95E-140	0.474092112	0.294	0.084	6.54E-136	5	RAB3IP

2.34E-139	0.469970025	0.306	0.09	7.84E-135	5	KCNJ16
1.46E-137	0.581586571	0.358	0.12	4.89E-133	5	CLDN2
1.66E-136	0.514109525	0.472	0.187	5.56E-132	5	SRRM2
3.35E-136	0.420477563	0.259	0.069	1.13E-131	5	CPM
1.52E-133	0.509360623	0.256	0.071	5.10E-129	5	CBX6
6.92E-132	0.524152608	0.465	0.182	2.32E-127	5	KCNJ15
3.31E-128	0.490520713	0.35	0.12	1.11E-123	5	AAK1
7.51E-127	0.551902592	0.553	0.242	2.52E-122	5	SLC3A1
3.20E-126	0.446576191	0.26	0.075	1.07E-121	5	THUMPD3-AS1
2.26E-125	0.544839045	0.92	0.627	7.57E-121	5	NAT8
2.60E-125	0.460240216	0.277	0.081	8.71E-121	5	NTN4
5.66E-122	0.492036254	0.715	0.371	1.90E-117	5	DDX5
2.80E-119	0.590264302	0.569	0.263	9.38E-115	5	SLC13A1
2.55E-117	0.446054416	0.267	0.082	8.54E-113	5	CCNL1
2.68E-117	0.508658284	0.355	0.128	8.97E-113	5	SLC39A5
3.43E-113	0.43491252	0.432	0.171	1.15E-108	5	CFI
6.88E-113	0.470999006	0.281	0.09	2.31E-108	5	ADAMTS9-AS1
8.85E-111	0.385983781	0.371	0.14	2.97E-106	5	HSPA1A
6.73E-110	0.544423658	0.561	0.272	2.26E-105	5	CEBPD
4.45E-108	0.479373966	0.372	0.145	1.49E-103	5	MAT2A
1.16E-107	0.468968168	0.315	0.111	3.89E-103	5	AKAP9
1.25E-107	0.439365634	0.4	0.158	4.20E-103	5	HPN
1.60E-106	0.417825818	0.27	0.087	5.38E-102	5	OGA
3.61E-106	0.616165512	0.359	0.139	1.21E-101	5	VCAM1
7.22E-106	0.424382869	0.364	0.136	2.42E-101	5	SDC4
8.96E-105	0.406582952	0.368	0.143	3.01E-100	5	ACTN4
1.79E-103	0.523186597	0.306	0.108	5.99E-99	5	SLC34A1
4.18E-103	0.365318121	0.255	0.08	1.40E-98	5	RRBP1
4.70E-103	0.441742328	0.773	0.439	1.57E-98	5	ATP1B1
1.75E-102	0.384498042	0.314	0.109	5.86E-98	5	SLC5A2
3.85E-102	0.404601036	0.308	0.109	1.29E-97	5	PLEKHA5
4.59E-102	0.487131973	0.587	0.293	1.54E-97	5	ANPEP
9.46E-102	0.362687869	0.251	0.08	3.17E-97	5	ASH1L
1.22E-101	0.432538552	0.548	0.268	4.10E-97	5	ATP1A1
1.70E-101	0.509031982	0.3	0.104	5.72E-97	5	ANK2
4.17E-101	0.41373575	0.404	0.164	1.40E-96	5	NFIB
1.89E-98	0.500068838	0.414	0.176	6.33E-94	5	SLC16A9
1.46E-96	0.415030352	0.266	0.09	4.90E-92	5	UGCG
3.44E-96	0.364587676	0.366	0.142	1.15E-91	5	ENPEP
5.05E-96	0.422822799	0.4	0.168	1.69E-91	5	KTN1

1.29E-95	0.415522064	0.374	0.154	4.32E-91	5	GTF2I
7.16E-95	0.384014348	0.26	0.087	2.40E-90	5	QTRT1
9.08E-94	0.41856359	0.254	0.084	3.04E-89	5	CDHR5
2.08E-93	0.429740672	0.385	0.16	6.96E-89	5	EMX2
2.12E-93	0.498057214	0.711	0.403	7.09E-89	5	BHMT
3.89E-93	0.343731202	0.252	0.082	1.31E-88	5	TINAGL1
5.09E-93	0.355692712	0.254	0.085	1.71E-88	5	NKTR
8.99E-93	0.406753674	0.595	0.303	3.02E-88	5	TSPAN1
3.06E-92	0.387003321	0.396	0.166	1.03E-87	5	CCDC198
3.54E-92	0.422975277	0.3	0.112	1.19E-87	5	GOLGA4
1.90E-90	0.38081741	0.344	0.138	6.36E-86	5	LUC7L3
8.31E-90	0.442688336	0.334	0.132	2.79E-85	5	SLC22A12
9.60E-90	0.376916415	0.324	0.126	3.22E-85	5	PLEKHA1
6.33E-89	0.42066289	0.295	0.111	2.12E-84	5	TRIM38
8.87E-87	0.417559261	0.325	0.132	2.97E-82	5	SNRNP70
1.64E-86	0.339372242	0.261	0.091	5.52E-82	5	ACLY
6.63E-86	0.356936095	0.285	0.105	2.22E-81	5	LPP
2.69E-85	0.450325752	0.399	0.18	9.01E-81	5	HIF1A
2.92E-85	0.416951782	0.423	0.191	9.79E-81	5	ARHGAP24
1.43E-84	0.380474562	0.262	0.093	4.79E-80	5	PARD6B
4.35E-84	0.34886785	0.284	0.106	1.46E-79	5	EPS8L2
6.46E-83	0.373512211	0.351	0.145	2.17E-78	5	CTTN
1.10E-82	0.39859275	0.294	0.113	3.68E-78	5	FHIT
1.41E-82	0.376750384	0.339	0.142	4.73E-78	5	TAF1D
1.43E-82	0.341019832	0.52	0.256	4.78E-78	5	CALD1
3.78E-82	0.354322117	0.486	0.23	1.27E-77	5	PTH1R
2.08E-81	0.467196553	0.421	0.196	6.98E-77	5	EPHX2
3.39E-81	0.356027703	0.382	0.166	1.14E-76	5	APP
6.45E-81	0.365738846	0.474	0.231	2.16E-76	5	RBM39
9.88E-77	0.34778889	0.378	0.167	3.31E-72	5	EPCAM
9.97E-77	0.459250399	0.3	0.121	3.35E-72	5	SOX4
9.27E-76	0.389866657	0.371	0.169	3.11E-71	5	SRSF11
8.46E-75	0.395180952	0.251	0.096	2.84E-70	5	SRSF6
1.68E-72	0.311443369	0.283	0.111	5.64E-68	5	UGT2A3
8.96E-72	0.301369898	0.417	0.196	3.01E-67	5	TMEM205
1.61E-70	0.356016193	0.372	0.171	5.41E-66	5	GALNT11
2.30E-70	0.336006339	0.426	0.205	7.72E-66	5	RBPMS
1.61E-68	0.301304166	0.321	0.138	5.41E-64	5	RBM47
1.64E-68	0.370779092	0.475	0.245	5.51E-64	5	RHOB
2.11E-68	0.326727068	0.272	0.11	7.06E-64	5	PUM3

1.58E-67	0.339969527	0.401	0.194	5.30E-63	5	LEPROT
5.49E-67	0.318341402	0.399	0.191	1.84E-62	5	SPINT2
3.87E-66	0.292205338	0.465	0.232	1.30E-61	5	APLP2
1.17E-65	0.297415282	0.946	0.787	3.94E-61	5	ITM2B
7.48E-65	0.322696823	0.262	0.108	2.51E-60	5	ZNF207
1.21E-64	0.304356444	0.253	0.102	4.05E-60	5	RSRP1
1.84E-64	0.342081542	0.338	0.153	6.16E-60	5	SLC4A4
5.25E-64	0.980651923	0.348	0.183	1.76E-59	5	RBP4
5.06E-63	0.312139491	0.411	0.202	1.70E-58	5	AIG1
2.25E-62	0.331922978	0.286	0.125	7.54E-58	5	SF1
2.94E-62	0.319172568	0.255	0.104	9.87E-58	5	SLC22A18
3.88E-62	0.317566852	0.252	0.104	1.30E-57	5	RBM25
6.55E-62	0.330401796	0.756	0.513	2.20E-57	5	LAPTM4A
1.17E-61	0.299548074	0.328	0.15	3.94E-57	5	AHCYL1
1.38E-61	0.346595777	0.689	0.427	4.64E-57	5	CLTRN
2.08E-61	0.30345459	0.326	0.147	6.97E-57	5	SLC17A3
2.34E-61	0.315317019	0.297	0.129	7.83E-57	5	BICC1
4.22E-60	0.283346809	0.283	0.121	1.41E-55	5	SERPINE2
6.18E-60	0.299302405	0.3	0.134	2.07E-55	5	MLEC
3.32E-59	0.31053746	0.368	0.18	1.11E-54	5	RAB29
4.48E-59	0.337834048	0.435	0.228	1.50E-54	5	LRPAP1
7.93E-59	0.28957855	0.353	0.166	2.66E-54	5	SLC16A4
1.07E-58	0.262139756	0.291	0.126	3.57E-54	5	ADGRG1
1.63E-58	0.339309237	0.426	0.223	5.45E-54	5	ZFP36L1
6.13E-58	0.344060254	0.339	0.163	2.06E-53	5	TINAG
3.64E-57	0.28472267	0.339	0.161	1.22E-52	5	CNPY2
5.92E-57	0.339320712	0.275	0.123	1.98E-52	5	TNRC6B
1.73E-56	0.269206036	0.254	0.107	5.81E-52	5	TNFRSF1A
1.25E-55	0.420154982	0.516	0.298	4.18E-51	5	AZGP1
1.19E-53	0.426523413	0.31	0.153	4.00E-49	5	KLF6
1.29E-53	0.328976251	0.53	0.315	4.32E-49	5	HIST1H4C
1.69E-53	0.310406981	0.272	0.121	5.66E-49	5	FN3K
1.71E-53	0.291576329	0.277	0.126	5.73E-49	5	MPHOSPH8
3.05E-53	0.285263246	0.765	0.5	1.02E-48	5	TMEM176A
4.31E-53	0.310777216	0.719	0.47	1.45E-48	5	TMEM176B
5.01E-52	0.275304309	0.382	0.196	1.68E-47	5	CTSC
9.25E-52	0.311736757	0.299	0.14	3.10E-47	5	SLC27A2
8.87E-50	0.277672033	0.48	0.271	2.97E-45	5	BSG
1.06E-48	0.310709569	0.355	0.181	3.56E-44	5	G0S2
1.79E-48	0.311324374	0.301	0.147	6.01E-44	5	CES2

1.07E-46	0.261234819	0.294	0.142	3.59E-42	5	SLC37A4
1.53E-46	0.251753054	0.319	0.16	5.14E-42	5	FUS
2.60E-45	0.415390246	0.296	0.151	8.73E-41	5	PLG
4.33E-45	0.305598829	0.356	0.188	1.45E-40	5	SLC22A6
5.97E-45	0.310898122	0.714	0.502	2.00E-40	5	TMBIM6
1.24E-44	0.30068861	0.466	0.275	4.14E-40	5	HSP90B1
1.69E-43	0.267482491	0.314	0.163	5.68E-39	5	CFLAR
3.29E-43	0.277941031	0.759	0.525	1.10E-38	5	HMGN3
6.11E-42	0.25543205	0.254	0.121	2.05E-37	5	RCAN1
1.93E-41	0.277216778	0.358	0.197	6.47E-37	5	SRSF2
2.26E-41	0.290188064	0.477	0.29	7.57E-37	5	SRSF5
4.15E-36	0.255495245	0.321	0.178	1.39E-31	5	NME3
5.32E-35	0.254407728	0.279	0.148	1.79E-30	5	DNAJB9
3.77E-29	0.259022709	0.263	0.143	1.27E-24	5	MAF
9.58E-29	0.262800315	0.556	0.385	3.21E-24	5	KRT18
7.99E-27	0.285224088	0.48	0.317	2.68E-22	5	SERPINA1
0	2.745795329	0.695	0.009	0	6	IGHM
0	2.520703685	0.931	0.004	0	6	MS4A1
0	2.434611014	0.957	0.067	0	6	CD37
0	2.396044098	0.99	0.311	0	6	HLA-DRA
0	2.263318813	0.924	0.006	0	6	CD79A
0	2.22250457	0.924	0.049	0	6	LTB
0	2.115838098	0.908	0.016	0	6	CD79B
0	2.051210238	0.974	0.141	0	6	HLA-DPB1
0	1.961694404	0.878	0.052	0	6	CXCR4
0	1.908113013	0.965	0.115	0	6	HLA-DPA1
0	1.880623939	0.921	0.062	0	6	HLA-DQB1
0	1.873679674	0.853	0.006	0	6	BANK1
0	1.855478766	0.963	0.09	0	6	CD52
0	1.690109722	0.807	0.049	0	6	KLF2
0	1.665361738	0.998	0.51	0	6	CD74
0	1.645417972	0.885	0.072	0	6	LAPTM5
0	1.628010521	0.806	0.037	0	6	HLA-DQA2
0	1.510375175	0.936	0.268	0	6	TXNIP
0	1.496596277	0.833	0.14	0	6	JUND
0	1.492815959	0.816	0.068	0	6	CD48
0	1.450243092	0.957	0.25	0	6	BTG1
0	1.423139513	0.847	0.077	0	6	HLA-DRB5
0	1.414127895	0.428	0	0	6	IGHD
0	1.394523266	0.592	0.003	0	6	VPREB3

0	1.392575306	0.8	0.04	0	6	HLA-DQA1
0	1.391915289	0.689	0.023	0	6	POU2F2
0	1.376628168	0.619	0.003	0	6	LINC00926
0	1.363259367	0.674	0.016	0	6	RALGPS2
0	1.329155452	0.675	0.011	0	6	MARCH1
0	1.309180011	0.754	0.06	0	6	CD53
0	1.29618967	0.694	0.033	0	6	RIPOR2
0	1.295363139	0.779	0.062	0	6	LIMD2
0	1.287195953	0.995	0.878	0	6	RPL30
0	1.275405076	0.713	0.045	0	6	EVI2B
0	1.261294724	0.683	0.028	0	6	NCF1
0	1.24486478	0.977	0.303	0	6	HLA-B
0	1.242049391	0.607	0.02	0	6	TCF4
0	1.239646172	0.828	0.085	0	6	CORO1A
0	1.238529044	0.572	0.004	0	6	BCL11A
0	1.216744897	0.777	0.089	0	6	HLA-DMA
0	1.212501924	0.924	0.202	0	6	HLA-E
0	1.204527881	0.695	0.052	0	6	ISG20
0	1.190136252	0.769	0.121	0	6	IER2
0	1.188681757	0.578	0.04	0	6	BIRC3
0	1.183889704	0.825	0.144	0	6	PNISR
0	1.182016392	0.64	0.03	0	6	MEF2C
0	1.165940272	0.594	0.012	0	6	BASP1
0	1.165061062	0.686	0.057	0	6	TNFAIP8
0	1.15290033	0.717	0.062	0	6	RAC2
0	1.152578428	0.596	0.101	0	6	HSPA1B
0	1.148143122	0.997	0.866	0	6	RPL39
0	1.145561598	1	0.372	0	6	MALAT1
0	1.129151397	0.663	0.051	0	6	PTPN6
0	1.123232798	0.257	0.005	0	6	IGLC3
0	1.116563236	0.626	0.03	0	6	HLA-DMB
0	1.112741518	0.944	0.25	0	6	HLA-DRB1
0	1.112671509	0.516	0.002	0	6	CD22
0	1.101636893	0.994	0.858	0	6	RPS2
0	1.100925916	0.994	0.82	0	6	RPL18
0	1.100821322	0.668	0.062	0	6	IFI16
0	1.097212421	0.633	0.127	0	6	JUN
0	1.092723507	0.81	0.099	0	6	ARHGDIB
0	1.084015588	0.523	0.019	0	6	SELL
0	1.080912851	0.997	0.917	0	6	RPL28

0	1.074076722	0.564	0.032	0	6	PLAC8
0	1.054012041	0.619	0.049	0	6	SP110
0	1.052177169	0.975	0.706	0	6	RPSA
0	1.047640255	0.508	0.001	0	6	BLK
0	1.044346514	0.508	0.007	0	6	HVCN1
0	1.041546762	0.991	0.727	0	6	RPS21
0	1.039382203	0.536	0.002	0	6	TNFRSF13C
0	1.039294559	0.919	0.062	0	6	IGKC
0	1.034479167	0.49	0.001	0	6	SPIB
0	1.026296608	0.529	0.017	0	6	FCMR
0	1.024796254	0.506	0.01	0	6	ADAM28
0	1.020012469	0.58	0.047	0	6	SMCHD1
0	1.017200687	0.585	0.042	0	6	SMAP2
0	1.016848421	0.53	0.012	0	6	IRF8
0	0.995335364	0.991	0.844	0	6	RPS3
0	0.994649332	0.959	0.367	0	6	HLA-A
0	0.994303127	0.676	0.094	0	6	SP100
0	0.989140232	0.592	0.049	0	6	BOD1L1
0	0.987357217	0.997	0.436	0	6	TMSB4X
0	0.986951846	0.549	0.046	0	6	CLEC2D
0	0.984568668	0.785	0.09	0	6	PTPRC
0	0.98232913	0.627	0.106	0	6	DUSP1
0	0.976604397	0.489	0.02	0	6	IGLC2
0	0.972159484	0.995	0.851	0	6	MT-ATP6
0	0.970455343	0.659	0.071	0	6	STK4
0	0.964575357	0.655	0.069	0	6	FXYD5
0	0.959650926	0.61	0.05	0	6	ACAP1
0	0.956815706	0.53	0.035	0	6	REL
0	0.954136115	0.496	0.024	0	6	PRKCB
0	0.950286181	0.537	0.026	0	6	IKZF3
0	0.946813534	0.48	0.003	0	6	GAPT
0	0.942807267	0.422	0.001	0	6	LINC02397
0	0.940975792	0.606	0.054	0	6	GPSM3
0	0.939922934	0.99	0.818	0	6	MT-ND3
0	0.936759427	0.661	0.111	0	6	TSC22D3
0	0.936545277	0.651	0.099	0	6	ANKRD12
0	0.935857676	0.521	0.048	0	6	PARP14
0	0.93095785	0.659	0.085	0	6	RSRP1
0	0.926658056	0.443	0.001	0	6	FCRLA
0	0.926505727	0.546	0.05	0	6	STK17A

0	0.926205235	0.569	0.059	0	6	ATM
0	0.924816154	0.532	0.033	0	6	ID3
0	0.923510019	0.477	0.043	0	6	FOSB
0	0.923151491	0.734	0.164	0	6	ZFP36L2
0	0.914851789	0.952	0.329	0	6	HLA-C
0	0.913410247	0.723	0.165	0	6	JUNB
0	0.912991082	0.959	0.664	0	6	EEF1B2
0	0.911852462	0.609	0.069	0	6	RNASET2
0	0.901514044	0.562	0.046	0	6	IKZF1
0	0.898856529	0.99	0.817	0	6	RPS11
0	0.898754361	0.479	0.015	0	6	LYL1
0	0.897717946	0.678	0.125	0	6	LUC7L3
0	0.895592024	0.5	0.026	0	6	SWAP70
0	0.886914992	0.418	0.037	0	6	CD69
0	0.886464077	0.485	0.018	0	6	RASGRP2
0	0.8837182	0.545	0.056	0	6	MYCBP2
0	0.882922246	0.59	0.083	0	6	PNN
0	0.88121829	0.546	0.044	0	6	STK17B
0	0.879053457	0.994	0.916	0	6	RPL18A
0	0.872654461	0.482	0.032	0	6	ANKRD44
0	0.871154327	0.914	0.365	0	6	DDX5
0	0.867556384	0.995	0.958	0	6	RPS27
0	0.862823642	0.381	0.003	0	6	P2RX5
0	0.860954406	0.555	0.058	0	6	KIAA1551
0	0.857087825	0.473	0.023	0	6	ORAI2
0	0.854991031	0.452	0.028	0	6	IER5
0	0.853824847	0.458	0.012	0	6	CD40
0	0.853239732	0.443	0.003	0	6	HLA-DOB
0	0.852567649	0.571	0.137	0	6	SMIM14
0	0.84597867	0.457	0.026	0	6	RCSD1
0	0.84491279	0.377	0.001	0	6	FCRL2
0	0.843354216	0.997	0.966	0	6	RPS18
0	0.84229962	0.452	0.01	0	6	LY86
0	0.841758352	0.505	0.042	0	6	SEPT6
0	0.827294355	0.738	0.211	0	6	ZFP36L1
0	0.826922738	0.734	0.17	0	6	SRSF7
0	0.825062189	0.477	0.051	0	6	BTG2
0	0.823938328	0.356	0.011	0	6	PHACTR1
0	0.823924881	0.525	0.041	0	6	TBC1D10C
0	0.82164508	0.415	0.001	0	6	TLR10

0	0.819188328	0.422	0.015	0	6	TRAF5
0	0.819159359	0.49	0.043	0	6	UCP2
0	0.817346306	0.423	0.007	0	6	HHEX
0	0.817315534	0.419	0.004	0	6	POU2AF1
0	0.81415298	0.391	0.003	0	6	AIM2
0	0.813369085	0.991	0.908	0	6	RPS19
0	0.810112	0.535	0.061	0	6	AKAP13
0	0.807491261	0.433	0.019	0	6	CIITA
0	0.804444634	0.545	0.072	0	6	PPM1K
0	0.804368504	0.749	0.176	0	6	DDX17
0	0.804178206	0.436	0.025	0	6	SEL1L3
0	0.802042283	0.993	0.961	0	6	RPS12
0	0.800217267	0.521	0.065	0	6	ARID1B
0	0.799990164	0.416	0.012	0	6	DAPP1
0	0.793015702	0.51	0.054	0	6	TRAF3IP3
0	0.791528266	0.612	0.11	0	6	PRRC2C
0	0.78935541	0.998	0.834	0	6	B2M
0	0.787352167	0.269	0.006	0	6	JCHAIN
0	0.777322786	0.789	0.233	0	6	GNAS
0	0.774224312	0.615	0.111	0	6	SF1
0	0.763649935	0.284	0.003	0	6	FCRL5
0	0.763494316	0.454	0.038	0	6	MARCKS
0	0.762767186	0.64	0.125	0	6	ARGLU1
0	0.761071813	0.409	0.012	0	6	TMEM154
0	0.754721939	0.495	0.055	0	6	LBH
0	0.751891916	0.739	0.181	0	6	N4BP2L2
0	0.750704227	0.648	0.147	0	6	FUS
0	0.750462527	0.454	0.041	0	6	CSK
0	0.742952251	0.427	0.056	0	6	FOXP1
0	0.740473735	0.567	0.099	0	6	ATRX
0	0.740437593	0.997	0.979	0	6	RPL13
0	0.739523241	0.398	0.011	0	6	LAT2
0	0.739037617	0.373	0.013	0	6	SP140
0	0.736076929	0.992	0.926	0	6	RPL9
0	0.735200774	0.995	0.949	0	6	RPS3A
0	0.734498231	0.567	0.096	0	6	TMEM243
0	0.734484549	0.456	0.044	0	6	CELF2
0	0.733877546	0.994	0.903	0	6	FAU
0	0.733283827	0.349	0.001	0	6	CD19
0	0.731232411	0.289	0.002	0	6	FAM30A

0	0.728057029	0.329	0.001	0	6	FAM129C
0	0.726260417	0.639	0.065	0	6	CTSS
0	0.724261245	0.368	0.018	0	6	GPR183
0	0.723749579	0.393	0.027	0	6	RHOH
0	0.72268384	0.338	0	0	6	FCRL1
0	0.720211318	0.561	0.091	0	6	KMT2E
0	0.712145134	0.386	0.021	0	6	INPP5D
0	0.71069489	0.5	0.061	0	6	CD47
0	0.709887833	0.502	0.08	0	6	MBD4
0	0.7089261	0.611	0.117	0	6	PSMB9
0	0.707369793	0.349	0	0	6	PAX5
0	0.705877813	0.345	0.003	0	6	CD180
0	0.704053757	0.263	0.001	0	6	FCER2
0	0.700829088	0.412	0.026	0	6	PLEKHO1
0	0.699191583	0.26	0.012	0	6	STAG3
0	0.698312695	0.587	0.136	0	6	SNX2
0	0.696351251	0.497	0.061	0	6	LCP1
0	0.693996366	0.438	0.054	0	6	PCSK7
0	0.691635687	0.523	0.072	0	6	LSP1
0	0.691615585	0.43	0.033	0	6	TRIM22
0	0.689245604	0.543	0.089	0	6	GNAI2
0	0.689145783	0.287	0.005	0	6	CD72
0	0.688828572	0.478	0.067	0	6	BPTF
0	0.683163356	0.365	0.038	0	6	GABPB1-AS1
0	0.681616693	0.997	0.971	0	6	RPS27A
0	0.68066978	0.985	0.885	0	6	RPL7A
0	0.679626496	0.985	0.86	0	6	RPS5
0	0.678806918	0.997	0.95	0	6	RPS8
0	0.678504496	0.447	0.059	0	6	MDM4
0	0.676595859	0.997	0.945	0	6	RPS15A
0	0.676520228	0.51	0.086	0	6	ANXA6
0	0.675287834	0.346	0.006	0	6	FCGR2B
0	0.675138991	0.267	0.001	0	6	LINC01857
0	0.674873754	0.505	0.083	0	6	PARP1
0	0.674378179	0.345	0.007	0	6	IGHA1
0	0.672124315	0.435	0.056	0	6	VPS13C
0	0.671368465	0.387	0.03	0	6	STRBP
0	0.671019021	0.315	0.005	0	6	AFF3
0	0.670885131	0.995	0.905	0	6	RPL37
0	0.670671685	0.321	0.01	0	6	SCIMP

0	0.665803313	0.438	0.062	0	6	ITSN2
0	0.664252399	0.479	0.065	0	6	HLA-F
0	0.663816236	0.296	0.008	0	6	CCR7
0	0.661822285	0.512	0.103	0	6	TRIM38
0	0.65885086	0.645	0.15	0	6	ARL6IP5
0	0.656265709	0.387	0.017	0	6	CYBB
0	0.655443727	0.595	0.142	0	6	SCAF11
0	0.65523439	0.329	0.004	0	6	CCR6
0	0.655064357	0.995	0.966	0	6	RPS23
0	0.65432365	0.419	0.036	0	6	PLCG2
0	0.652762816	0.507	0.093	0	6	RBM25
0	0.650253413	0.36	0.021	0	6	RIC3
0	0.64972529	0.476	0.077	0	6	NKTR
0	0.648883027	0.355	0.022	0	6	CNTRL
0	0.648663143	0.284	0.002	0	6	TNFRSF13B
0	0.648432162	0.756	0.22	0	6	VIM
0	0.647497993	0.353	0.012	0	6	BCAS4
0	0.646187652	0.465	0.069	0	6	JMJD1C
0	0.645485174	0.521	0.111	0	6	ZFP36
0	0.645302254	0.385	0.03	0	6	MIS18BP1
0	0.642881496	0.444	0.079	0	6	IRF1
0	0.63909392	0.306	0.012	0	6	ZNF107
0	0.638995016	0.334	0.008	0	6	BTK
0	0.638887965	0.43	0.054	0	6	SETX
0	0.63849553	0.514	0.104	0	6	SNHG7
0	0.637846495	0.517	0.078	0	6	EVL
0	0.635805575	0.418	0.044	0	6	HCLS1
0	0.634714866	0.334	0.009	0	6	BLNK
0	0.634317673	0.387	0.028	0	6	DCK
0	0.631343816	0.459	0.073	0	6	NSD3
0	0.628876876	0.396	0.049	0	6	DOCK8
0	0.628749943	0.303	0.003	0	6	CPNE5
0	0.628539928	0.389	0.04	0	6	CYTIP
0	0.62852199	0.377	0.03	0	6	SLC38A1
0	0.627142273	0.351	0.026	0	6	NCOA3
0	0.62610931	0.356	0.02	0	6	LYN
0	0.62243454	0.355	0.029	0	6	QRSL1
0	0.621911162	0.548	0.086	0	6	GMFG
0	0.62171122	0.464	0.068	0	6	ARID4B
0	0.619765715	0.329	0.012	0	6	SYK

0	0.617518718	0.33	0.024	0	6	CYB561A3
0	0.61676345	0.377	0.033	0	6	ARHGAP45
0	0.6161788	0.288	0.005	0	6	TSPAN13
0	0.616069448	0.584	0.091	0	6	EMP3
0	0.615906642	0.425	0.059	0	6	FNBP1
0	0.612956644	0.436	0.066	0	6	PDCD4
0	0.612956133	0.378	0.033	0	6	ALOX5AP
0	0.607751306	0.521	0.113	0	6	TNRC6B
0	0.607240297	0.441	0.072	0	6	PRPF38B
0	0.605732763	0.396	0.05	0	6	BTN3A2
0	0.604281574	0.286	0.02	0	6	IFI44L
0	0.603828956	0.3	0.01	0	6	HSH2D
0	0.603616085	0.48	0.099	0	6	MBNL1
0	0.603391831	0.473	0.088	0	6	TAPBP
0	0.602681252	0.348	0.03	0	6	GNG7
0	0.602248836	0.398	0.05	0	6	ATP2B1
0	0.601936628	0.333	0.02	0	6	SP140L
0	0.601626051	0.365	0.044	0	6	FNBP4
0	0.601623489	0.391	0.05	0	6	ZNF292
0	0.601411475	0.353	0.026	0	6	IL16
0	0.598972176	0.414	0.061	0	6	DDIT4
0	0.595380104	0.431	0.056	0	6	CD44
0	0.595175992	0.314	0.018	0	6	CD82
0	0.593169712	0.384	0.055	0	6	RNASEH2B
0	0.590240109	0.812	0.237	0	6	MTRNR2L12
0	0.590163657	0.326	0.02	0	6	ST6GAL1
0	0.587331324	0.333	0.025	0	6	ARHGAP15
0	0.586922101	0.454	0.081	0	6	OGA
0	0.586596235	0.413	0.059	0	6	CCND3
0	0.586219563	0.998	0.977	0	6	RPL32
0	0.584932332	0.377	0.037	0	6	GGA2
0	0.582883565	0.378	0.043	0	6	OFD1
0	0.582791653	0.381	0.05	0	6	CNPY3
0	0.582519658	0.294	0.011	0	6	TFEB
0	0.581641493	0.365	0.048	0	6	TTC14
0	0.580080425	0.3	0.008	0	6	TMEM156
0	0.57743221	0.372	0.04	0	6	FMNL1
0	0.575047051	0.495	0.09	0	6	POLR2J3.1
0	0.57416743	0.395	0.047	0	6	ARHGEF1
0	0.57258368	0.994	0.967	0	6	RPL11

0	0.571012097	0.369	0.043	0	6	PLP2
0	0.570575764	0.409	0.064	0	6	EBLN3P
0	0.567733978	0.345	0.03	0	6	PSMB8-AS1
0	0.567706419	0.394	0.07	0	6	SLC38A2
0	0.566944484	0.296	0.027	0	6	MARCKSL1
0	0.56690955	0.997	0.981	0	6	RPLP1
0	0.565531999	0.35	0.041	0	6	CHD2
0	0.564451186	0.329	0.029	0	6	PRDM2
0	0.563283871	0.346	0.044	0	6	ATF7IP
0	0.562311419	0.398	0.067	0	6	WASHC4
0	0.560697943	0.387	0.052	0	6	KMT2A
0	0.558750316	0.285	0.014	0	6	FCHSD2
0	0.558653715	0.36	0.036	0	6	GRK2
0	0.557494989	0.533	0.093	0	6	CD81
0	0.556102058	0.431	0.076	0	6	CCNL1
0	0.553491315	0.456	0.091	0	6	BRD2
0	0.553345815	0.404	0.061	0	6	SH3BP5
0	0.552187329	0.411	0.056	0	6	VASP
0	0.551966886	0.364	0.038	0	6	CNN2
0	0.55156422	0.309	0.011	0	6	NCF4
0	0.551023511	0.321	0.036	0	6	SAMD9
0	0.547390076	0.326	0.04	0	6	PPP1R15A
0	0.546638285	0.376	0.042	0	6	ETS1
0	0.546317209	0.289	0.009	0	6	CYSLTR1
0	0.545166536	0.426	0.081	0	6	ANKRD11
0	0.54497994	0.391	0.053	0	6	LYSMD2
0	0.543792035	0.385	0.065	0	6	DBNL
0	0.543747868	0.407	0.065	0	6	SYNGR2
0	0.537921873	0.304	0.02	0	6	CDKN2D
0	0.536221236	0.262	0.005	0	6	PARP15
0	0.535556208	0.28	0.012	0	6	ATP2A3
0	0.534877352	0.326	0.031	0	6	ARHGAP30
0	0.534250556	0.28	0.016	0	6	TAGAP
0	0.533118833	0.357	0.052	0	6	TUT4
0	0.531152694	0.306	0.033	0	6	PLEKHA2
0	0.531059667	0.346	0.036	0	6	AKNA
0	0.530925303	0.322	0.028	0	6	TMC6
0	0.529721675	0.316	0.024	0	6	RGS19
0	0.529488681	0.309	0.029	0	6	CD27
0	0.52454185	0.296	0.021	0	6	TMC8

0	0.523463608	0.312	0.027	0	6	NCKAP1L
0	0.52247604	0.348	0.044	0	6	IL2RG
0	0.520640938	0.376	0.051	0	6	UTRN
0	0.519977703	0.476	0.094	0	6	WSB1
0	0.514937072	0.294	0.023	0	6	CD55
0	0.514460185	0.299	0.025	0	6	SYTL1
0	0.514345129	0.378	0.065	0	6	ROCK1
0	0.512224665	0.397	0.066	0	6	SEPT9
0	0.511754619	0.309	0.022	0	6	S1PR4
0	0.507779326	0.338	0.04	0	6	PPP1R18
0	0.506168511	0.263	0.008	0	6	FGD2
0	0.506013496	0.316	0.037	0	6	APBB1IP
0	0.50578154	0.316	0.033	0	6	PTK2B
0	0.503709486	0.314	0.038	0	6	BCL2
0	0.503252334	0.373	0.062	0	6	TMEM259
0	0.503112878	0.345	0.054	0	6	RBM6
0	0.498294924	0.284	0.032	0	6	SLC2A3
0	0.49672484	0.312	0.03	0	6	LRMP
0	0.495522658	0.264	0.017	0	6	AC025164.1
0	0.495034482	0.312	0.042	0	6	PDE7A
0	0.494465709	0.275	0.02	0	6	UNC93B1
0	0.492237229	0.294	0.035	0	6	ZNF791
0	0.491518789	0.291	0.024	0	6	UVRAG
0	0.487258045	0.29	0.031	0	6	CYTH1
0	0.487182393	0.3	0.024	0	6	MAP4K4
0	0.484645852	0.355	0.058	0	6	EPC1
0	0.48381531	0.275	0.028	0	6	SMC6
0	0.482131933	0.297	0.032	0	6	GLIPR1
0	0.481871862	0.332	0.049	0	6	KANSL1
0	0.481056344	0.322	0.045	0	6	CDK13
0	0.479234396	0.31	0.036	0	6	ZCCHC7
0	0.479187775	0.266	0.028	0	6	PLEK
0	0.476398136	0.317	0.045	0	6	SIPA1
0	0.475088788	0.28	0.028	0	6	XAF1
0	0.46453965	0.256	0.02	0	6	TCOF1
0	0.458037839	0.264	0.02	0	6	SIT1
0	0.453669267	0.299	0.041	0	6	LNPEP
0	0.452947407	0.276	0.029	0	6	ERAP2
0	0.451568808	0.275	0.027	0	6	GRK6
0	0.45064491	0.341	0.052	0	6	TGFB1

0	0.448300491	0.306	0.041	0	6	MAP3K1
0	0.447825973	0.273	0.026	0	6	ARHGAP4
0	0.445251343	0.267	0.03	0	6	ITGA4
0	0.443787861	0.312	0.045	0	6	CYBC1
0	0.442876302	0.253	0.023	0	6	PXK
0	0.442527812	0.314	0.042	0	6	MKNK2
0	0.441241383	0.269	0.025	0	6	MYO1G
0	0.432462107	0.254	0.029	0	6	NIN
0	0.431700257	0.253	0.023	0	6	FAM214A
0	0.430532802	0.261	0.025	0	6	MORC3
0	0.419342456	0.262	0.03	0	6	WAS
5.64E-307	0.468962887	0.302	0.043	1.89E-302	6	LENG8
7.88E-306	0.472630833	0.329	0.051	2.64E-301	6	SCPEP1
3.67E-305	0.403804414	0.262	0.032	1.23E-300	6	CSNK1G2
3.73E-305	0.473106621	0.324	0.049	1.25E-300	6	RBM26
6.63E-303	0.500704943	0.415	0.078	2.22E-298	6	CCDC85B
1.48E-301	0.482518011	0.389	0.07	4.96E-297	6	NASP
2.16E-301	0.444913725	0.299	0.043	7.25E-297	6	CHD1
5.06E-301	0.63955585	0.591	0.15	1.70E-296	6	AES
1.13E-299	0.67575312	0.7	0.205	3.79E-295	6	SEPT7
3.84E-297	0.438994706	0.307	0.046	1.29E-292	6	KDM5A
1.13E-295	0.463329941	0.319	0.049	3.78E-291	6	BAZ1A
1.08E-294	0.39099639	0.253	0.031	3.64E-290	6	VOPP1
5.84E-294	0.462607365	0.337	0.054	1.96E-289	6	GYPC
3.09E-293	0.487746441	0.365	0.063	1.04E-288	6	RSBN1L
2.02E-292	0.52523045	0.406	0.078	6.79E-288	6	TPR
4.89E-292	0.57518716	0.51	0.117	1.64E-287	6	MPHOSPH8
3.08E-287	0.520595832	0.997	0.98	1.03E-282	6	RPL10
6.52E-287	0.567276946	0.993	0.917	2.19E-282	6	RPL23A
7.58E-287	0.499239399	0.414	0.081	2.54E-282	6	FAM49B
3.30E-286	0.417163166	0.32	0.051	1.11E-281	6	MBD2
2.83E-283	0.603880948	0.994	0.888	9.49E-279	6	RPS29
3.40E-280	0.542547707	0.445	0.095	1.14E-275	6	ICAM3
3.45E-279	0.583841019	0.644	0.182	1.16E-274	6	SRRM2
1.12E-278	0.525704119	0.333	0.057	3.76E-274	6	PHIP
4.03E-278	0.40183108	0.296	0.045	1.35E-273	6	COBLL1
1.70E-277	0.402937381	0.258	0.034	5.72E-273	6	PIK3IP1
2.85E-277	0.457549147	0.334	0.057	9.54E-273	6	NSD1
2.75E-276	0.418533629	0.285	0.042	9.24E-272	6	BDP1
3.98E-276	0.756780306	0.877	0.374	1.34E-271	6	NOP53

3.26E-275	0.436651435	0.297	0.046	1.09E-270	6	RBM5
4.74E-275	0.47373042	0.346	0.061	1.59E-270	6	USP15
8.11E-274	0.421120886	0.276	0.04	2.72E-269	6	DNAJC10
2.63E-273	0.45141314	0.286	0.043	8.81E-269	6	INTS6
3.01E-272	0.441769128	0.312	0.05	1.01E-267	6	PHF20
9.50E-272	0.450999072	0.367	0.068	3.19E-267	6	UPF2
1.19E-271	0.633880877	0.597	0.165	3.98E-267	6	STX7
4.17E-268	0.427628178	0.338	0.059	1.40E-263	6	CDC42SE1
1.61E-267	0.54384673	0.536	0.135	5.41E-263	6	JAK1
7.46E-266	0.548974303	0.994	0.941	2.50E-261	6	RPLP2
7.64E-264	0.493218298	0.994	0.96	2.56E-259	6	RPS14
1.01E-263	0.732455339	0.992	0.795	3.39E-259	6	MT-ND1
4.40E-263	0.733248962	0.885	0.395	1.48E-258	6	PABPC1
4.67E-263	0.347438715	0.333	0.057	1.57E-258	6	BST2
1.39E-260	0.551999299	0.581	0.159	4.67E-256	6	ANP32B
2.74E-257	0.447317944	0.302	0.049	9.18E-253	6	ZEB2
1.92E-254	0.413058795	0.307	0.052	6.44E-250	6	CREBRF
5.88E-254	0.507620413	0.5	0.124	1.97E-249	6	GPBP1
1.94E-253	0.46004206	0.437	0.097	6.50E-249	6	WASF2
3.18E-253	0.500533884	0.497	0.121	1.07E-248	6	CTSZ
4.20E-252	0.506960789	0.99	0.914	1.41E-247	6	RPS16
3.23E-251	0.526291047	0.524	0.135	1.08E-246	6	TAF1D
2.63E-250	0.492513343	0.457	0.107	8.82E-246	6	GRB2
3.52E-249	0.502453438	0.532	0.138	1.18E-244	6	PSMB8
1.20E-248	0.515450077	0.407	0.089	4.03E-244	6	BCLAF1
3.65E-248	0.465872912	0.393	0.083	1.23E-243	6	DDX6
6.91E-248	0.498886194	0.363	0.073	2.32E-243	6	ELF1
7.50E-246	0.489695131	0.529	0.136	2.52E-241	6	HNRNPH1
2.33E-245	0.54017398	0.473	0.116	7.80E-241	6	GCC2
2.99E-244	0.384998393	0.293	0.049	1.00E-239	6	NIPBL
1.53E-242	0.333462993	0.28	0.045	5.13E-238	6	PYCARD
2.68E-242	0.433467573	0.993	0.965	8.99E-238	6	RPL19
3.59E-241	0.457747018	0.344	0.067	1.20E-236	6	NEMF
2.03E-240	0.547427001	0.556	0.154	6.80E-236	6	ACTR2
2.76E-240	0.415276889	0.313	0.056	9.26E-236	6	CLK1
1.35E-238	0.632000052	0.999	0.912	4.54E-234	6	MT-CO2
2.46E-238	0.353176972	0.293	0.05	8.24E-234	6	FLNA
9.20E-238	0.482110328	0.51	0.132	3.09E-233	6	SF3B1
9.36E-236	0.401521463	0.277	0.046	3.14E-231	6	PCF11
4.40E-235	0.379323104	0.266	0.043	1.48E-230	6	CENPC

2.45E-234	0.457692702	0.355	0.073	8.23E-230	6	PHF3
8.30E-234	0.388534237	0.273	0.045	2.78E-229	6	TAP1
7.84E-233	0.475575317	0.42	0.097	2.63E-228	6	DRAM2
1.50E-232	0.474207114	0.99	0.922	5.05E-228	6	RPL29
1.03E-231	0.503445852	0.262	0.043	3.44E-227	6	PLPP5
2.94E-231	0.419269603	0.346	0.07	9.85E-227	6	PHF14
1.08E-230	0.460013437	0.424	0.099	3.63E-226	6	NCOR1
1.14E-230	0.479488504	0.456	0.112	3.81E-226	6	LRRFIP1
2.51E-230	0.462626725	0.394	0.088	8.40E-226	6	ORMDL1
6.36E-229	0.379092653	0.278	0.047	2.13E-224	6	CYLD
4.50E-226	0.368048029	0.293	0.053	1.51E-221	6	PPP4R3A
6.15E-226	0.48931094	0.476	0.123	2.06E-221	6	MTPN
6.71E-226	0.490976433	0.475	0.123	2.25E-221	6	RPL22L1
5.30E-225	0.412742721	0.261	0.043	1.78E-220	6	ARID4A
5.53E-225	0.6548617	0.99	0.837	1.86E-220	6	MT-CYB
8.94E-225	0.52975178	0.61	0.187	3.00E-220	6	SRSF2
3.87E-221	0.372423564	0.296	0.054	1.30E-216	6	ZBTB7A
1.33E-219	0.619787693	0.824	0.332	4.46E-215	6	PNRC1
2.80E-219	0.456887161	0.996	0.927	9.39E-215	6	RPL36
4.52E-219	0.380172514	0.27	0.047	1.51E-214	6	CCDC32
8.59E-219	0.455611356	0.424	0.103	2.88E-214	6	CHD9
1.06E-218	0.546033505	0.674	0.224	3.56E-214	6	RBM39
5.21E-218	0.418068442	0.34	0.071	1.75E-213	6	PCM1
1.23E-217	0.399881981	0.29	0.054	4.12E-213	6	TMF1
1.25E-217	0.50523149	0.407	0.098	4.20E-213	6	CCDC50
3.26E-217	0.466356769	0.986	0.931	1.09E-212	6	RPS24
6.24E-216	0.417921112	0.363	0.079	2.09E-211	6	WIPF1
1.83E-215	0.425623757	0.379	0.086	6.13E-211	6	ZRANB2
4.60E-215	0.51792235	0.977	0.864	1.54E-210	6	RPL5
2.33E-214	0.45414483	0.465	0.122	7.83E-210	6	SRSF10
3.80E-214	0.375091514	0.281	0.051	1.27E-209	6	TRIM56
4.62E-214	0.370839798	0.26	0.044	1.55E-209	6	RICTOR
5.40E-214	0.367396411	0.293	0.055	1.81E-209	6	USP34
6.23E-214	0.479071171	0.432	0.108	2.09E-209	6	AKAP9
1.23E-213	0.499919361	0.992	0.913	4.12E-209	6	RPL27A
1.84E-213	0.502988921	0.484	0.131	6.16E-209	6	YPEL3
9.60E-213	0.574208721	0.644	0.211	3.22E-208	6	HNRNPU
3.68E-209	0.452743284	0.996	0.935	1.23E-204	6	RPS13
8.49E-208	0.435183146	0.348	0.077	2.85E-203	6	ASH1L
6.14E-207	0.476614177	0.458	0.123	2.06E-202	6	TAF7

2.70E-206	0.338681519	0.251	0.043	9.06E-202	6	MIA3
2.52E-205	0.401451998	0.329	0.07	8.44E-201	6	TNFRSF14
4.22E-205	0.414037836	0.37	0.085	1.42E-200	6	CASP4
6.72E-205	0.40493964	0.323	0.068	2.25E-200	6	BRD4
2.04E-204	0.40373362	0.367	0.084	6.85E-200	6	PABPN1
4.48E-204	0.442539265	0.32	0.067	1.50E-199	6	SKIL
6.78E-203	0.34190716	0.366	0.083	2.27E-198	6	MYH9
2.76E-202	0.3660062	0.278	0.052	9.25E-198	6	SMG1
6.95E-201	0.347096421	0.295	0.058	2.33E-196	6	HDAC1
9.86E-201	0.348141441	0.253	0.044	3.31E-196	6	CDKN1B
2.31E-200	0.632629552	0.78	0.331	7.76E-196	6	ZFAS1
1.15E-199	0.425899264	0.989	0.955	3.85E-195	6	RPL3
1.49E-198	0.41381739	0.345	0.078	5.01E-194	6	PSMA3-AS1
4.91E-198	0.358449844	0.293	0.059	1.65E-193	6	KLF13
1.27E-197	0.525589882	0.534	0.163	4.26E-193	6	SRSF11
7.60E-197	0.424100468	0.376	0.09	2.55E-192	6	PSIP1
2.95E-196	0.336553462	0.26	0.047	9.89E-192	6	KMT2C
3.14E-196	0.372036084	0.301	0.061	1.05E-191	6	IDS
7.99E-196	0.568593535	0.974	0.65	2.68E-191	6	MT-ND5
1.73E-195	0.453348741	0.992	0.905	5.81E-191	6	RPS9
2.78E-193	0.489833096	0.983	0.871	9.34E-189	6	RPL10A
3.13E-193	0.410396983	0.392	0.097	1.05E-188	6	CDC40
1.18E-189	0.411585597	0.327	0.073	3.96E-185	6	TAOK3
1.38E-189	0.376448473	0.321	0.07	4.63E-185	6	PPP1R12A
2.11E-189	0.384150202	0.309	0.066	7.08E-185	6	ZC3HAV1
2.25E-189	0.354777541	0.26	0.049	7.55E-185	6	RBBP6
8.05E-189	0.332014085	0.265	0.05	2.70E-184	6	MLLT6
4.56E-188	0.362711012	0.27	0.053	1.53E-183	6	BIRC6
5.48E-188	0.352124794	0.256	0.048	1.84E-183	6	OGT
5.96E-186	0.361118453	0.326	0.073	2.00E-181	6	IQGAP1
6.46E-185	0.320482431	0.26	0.049	2.17E-180	6	RPA2
1.23E-182	0.525730563	0.73	0.286	4.12E-178	6	ERP29
7.58E-182	0.318602968	0.254	0.048	2.54E-177	6	HERC4
4.71E-181	0.346337899	0.319	0.072	1.58E-176	6	UBE2J1
2.13E-180	0.347970472	0.261	0.051	7.15E-176	6	HNRNPL
4.31E-177	0.409700506	0.991	0.922	1.45E-172	6	RPL14
4.61E-177	0.355714809	0.329	0.077	1.54E-172	6	TANK
2.44E-176	0.314288404	0.286	0.061	8.18E-172	6	ZNF638
2.82E-175	0.321437807	0.268	0.055	9.45E-171	6	PRKDC
5.77E-175	0.318988418	0.263	0.053	1.93E-170	6	SMARCA2

7.14E-175	0.438257481	0.517	0.164	2.39E-170	6	UBE2I
2.89E-174	0.357862906	0.251	0.049	9.68E-170	6	CCAR1
1.85E-173	0.519862704	0.746	0.304	6.22E-169	6	NAP1L1
1.01E-171	0.370934993	0.381	0.1	3.40E-167	6	PLEKHF2
1.96E-171	0.346986344	0.314	0.072	6.58E-167	6	MBP
3.43E-171	0.380537859	0.332	0.08	1.15E-166	6	USP8
2.29E-170	0.529212017	0.996	0.903	7.67E-166	6	MT-CO3
3.41E-170	0.35780897	0.309	0.071	1.14E-165	6	EML4
4.95E-170	0.34555367	0.293	0.064	1.66E-165	6	ELOVL5
5.55E-170	0.418827919	0.574	0.192	1.86E-165	6	ACTR3
1.27E-167	0.327210566	0.288	0.063	4.25E-163	6	SINHCAF
7.05E-167	0.628223276	0.881	0.462	2.36E-162	6	ACTG1
1.79E-165	0.397323805	0.45	0.134	6.00E-161	6	HNRNPD
2.26E-165	0.457928871	0.605	0.215	7.58E-161	6	YWHAZ
2.04E-164	0.319146494	0.337	0.083	6.84E-160	6	MED10
5.31E-164	0.499301265	0.998	0.902	1.78E-159	6	MT-CO1
1.93E-163	0.466615429	0.615	0.223	6.48E-159	6	HERPUD1
1.98E-163	0.319302662	0.374	0.099	6.65E-159	6	CMTM6
3.07E-163	0.325946595	0.27	0.058	1.03E-158	6	ACAP2
1.42E-161	0.385579701	0.995	0.923	4.75E-157	6	RPS4X
4.20E-161	0.37865067	0.372	0.101	1.41E-156	6	GGNBP2
1.17E-160	0.365408873	0.313	0.076	3.93E-156	6	RSF1
2.36E-159	0.299617407	0.273	0.06	7.92E-155	6	TMPO
1.26E-158	0.334220895	0.298	0.07	4.22E-154	6	IFNGR2
1.51E-158	0.3038528	0.268	0.059	5.05E-154	6	IRF3
1.96E-158	0.338399867	0.996	0.975	6.57E-154	6	RPS28
5.05E-158	0.311139359	0.288	0.066	1.69E-153	6	SMC3
1.04E-157	0.333630134	0.27	0.06	3.50E-153	6	TRABD
1.97E-156	0.398913067	0.464	0.145	6.62E-152	6	SFPQ
4.05E-156	0.416394237	0.496	0.163	1.36E-151	6	PPIG
1.69E-155	0.309866403	0.286	0.066	5.68E-151	6	PTEN
3.41E-154	0.412484978	0.976	0.861	1.14E-149	6	RACK1
8.47E-154	0.405409612	0.385	0.11	2.84E-149	6	TRA2B
2.07E-152	0.358199894	0.454	0.141	6.95E-148	6	DDX3X
2.31E-152	0.3816625	0.347	0.093	7.74E-148	6	DHX36
6.33E-151	0.33018759	0.271	0.062	2.12E-146	6	THOC2
1.18E-150	0.364386403	0.325	0.084	3.94E-146	6	CCNDBP1
1.36E-150	0.500718075	0.933	0.686	4.57E-146	6	RPS26
2.68E-150	0.336550306	0.278	0.065	8.98E-146	6	PPP3CA
5.07E-150	0.299337828	0.263	0.059	1.70E-145	6	ZNF24

8.43E-149	0.307922605	0.26	0.058	2.83E-144	6	ABHD17A
8.38E-148	0.34906922	0.48	0.156	2.81E-143	6	NFKBIA
4.63E-147	0.370022115	0.335	0.09	1.55E-142	6	ADAR
4.90E-147	0.327297159	0.313	0.08	1.64E-142	6	AC245297.3
1.45E-146	0.376996727	0.986	0.924	4.87E-142	6	RPL15
4.17E-146	0.311012234	0.308	0.078	1.40E-141	6	MUC20-OT1
9.93E-146	0.448709326	0.997	0.868	3.33E-141	6	MT-ND4
1.24E-145	0.364264417	0.55	0.193	4.14E-141	6	EIF3D
1.29E-145	0.323380774	0.365	0.102	4.33E-141	6	RNPS1
1.40E-145	0.420451502	0.694	0.282	4.68E-141	6	SRSF5
2.64E-144	0.43388643	0.953	0.567	8.86E-140	6	CYBA
8.23E-144	0.29063251	0.259	0.059	2.76E-139	6	PHKB
7.62E-143	0.3841447	0.416	0.129	2.55E-138	6	SNRNP70
1.21E-142	0.357998762	0.347	0.097	4.05E-138	6	U2SURP
1.25E-142	0.432610013	0.991	0.939	4.20E-138	6	RPL21
1.57E-142	0.526569579	0.945	0.608	5.27E-138	6	RPS10
5.01E-142	0.369752332	0.986	0.883	1.68E-137	6	UBA52
1.05E-141	0.304907261	0.303	0.077	3.53E-137	6	TIAL1
1.75E-141	0.39079347	0.484	0.165	5.88E-137	6	NT5C
5.61E-141	0.338768291	0.296	0.075	1.88E-136	6	PPP1R10
7.17E-140	0.34383511	0.309	0.081	2.41E-135	6	ADD1
2.17E-139	0.332876203	0.417	0.129	7.26E-135	6	DDX46
6.94E-139	0.310532904	0.994	0.966	2.33E-134	6	RPS15
9.44E-139	0.29310295	0.271	0.065	3.16E-134	6	CPNE1
1.46E-138	0.381743747	0.451	0.148	4.88E-134	6	MTDH
1.61E-138	0.323774501	0.323	0.087	5.40E-134	6	SEPT1
3.21E-138	0.317800152	0.39	0.117	1.08E-133	6	HNRNPH3
5.27E-136	0.368635904	0.607	0.232	1.77E-131	6	LSM7
1.51E-135	0.305513165	0.298	0.078	5.05E-131	6	USF2
2.33E-134	0.329529732	0.261	0.064	7.81E-130	6	EIF2AK2
4.59E-134	0.735787449	0.407	0.14	1.54E-129	6	HSPA1A
2.76E-133	0.274529309	0.287	0.074	9.24E-129	6	MACF1
6.05E-133	0.308536193	0.398	0.123	2.03E-128	6	TERF2IP
6.01E-132	0.29880453	0.3	0.08	2.02E-127	6	CELF1
6.37E-131	0.322414414	0.324	0.091	2.14E-126	6	KPNB1
7.17E-131	0.349471408	0.605	0.233	2.40E-126	6	SRRM1
1.88E-128	0.303431942	0.277	0.072	6.31E-124	6	KRR1
4.08E-128	0.256134223	0.307	0.084	1.37E-123	6	FOS
4.77E-128	0.337902511	0.411	0.133	1.60E-123	6	IFT57
1.66E-127	0.466421813	0.865	0.472	5.55E-123	6	HNRNPA2B1

9.88E-127	0.292651261	0.388	0.122	3.31E-122	6	PRPF4B
1.09E-124	0.349357535	0.543	0.205	3.66E-120	6	CAP1
1.68E-124	0.328459305	0.347	0.105	5.65E-120	6	SMARCB1
2.08E-124	0.298281801	0.368	0.114	6.98E-120	6	EIF3A
4.65E-123	0.384230529	0.701	0.307	1.56E-118	6	EIF3F
2.32E-122	0.313016012	0.251	0.063	7.77E-118	6	ESF1
2.74E-122	0.303095503	0.325	0.095	9.19E-118	6	WAC
3.01E-122	0.262645564	0.255	0.064	1.01E-117	6	ABI1
3.15E-122	0.309155548	0.435	0.148	1.06E-117	6	LSM8
4.59E-122	0.330160113	0.571	0.22	1.54E-117	6	SYPL1
2.93E-121	0.301159667	0.345	0.105	9.81E-117	6	ZNF207
4.36E-121	0.343144206	0.671	0.282	1.46E-116	6	SON
1.03E-120	0.325027317	0.407	0.137	3.47E-116	6	TTC3
2.06E-120	0.320726971	0.986	0.946	6.90E-116	6	RPL26
8.16E-120	0.260806741	0.321	0.094	2.74E-115	6	RNMT
1.05E-119	0.317078448	0.357	0.111	3.51E-115	6	CDV3
1.10E-119	0.305491349	0.3	0.086	3.69E-115	6	LMO4
1.25E-119	0.305126519	0.373	0.119	4.19E-115	6	THRAP3
1.03E-118	0.256776581	0.251	0.063	3.45E-114	6	RHOG
8.22E-118	0.294597771	0.383	0.125	2.76E-113	6	HNRNPR
8.46E-118	0.272769178	0.27	0.072	2.84E-113	6	ATG12
1.54E-116	0.307225485	0.826	0.399	5.15E-112	6	MT-ND4L
3.90E-116	0.346021071	0.586	0.239	1.31E-111	6	TPM3
2.52E-114	0.292734154	0.463	0.167	8.44E-110	6	RSRC2
2.86E-114	0.320152141	0.388	0.13	9.58E-110	6	SLTM
1.07E-113	0.329700478	0.566	0.228	3.58E-109	6	HNRNPA3
1.94E-113	0.376922546	0.779	0.375	6.52E-109	6	ARPC2
3.20E-113	0.26036161	0.287	0.081	1.07E-108	6	RNF19A
1.22E-112	0.27260538	0.285	0.081	4.11E-108	6	APPL1
1.29E-111	0.367949709	0.693	0.314	4.32E-107	6	SERP1
4.42E-111	0.294217507	0.434	0.154	1.48E-106	6	HNRNPM
4.82E-111	0.302485911	0.496	0.187	1.62E-106	6	SELENOF
1.89E-109	0.271123494	0.459	0.167	6.33E-105	6	PHB2
3.71E-109	0.309226366	0.47	0.174	1.24E-104	6	ARPC1B
2.01E-108	0.257174476	0.253	0.069	6.75E-104	6	MIDN
2.33E-108	0.319704906	0.995	0.896	7.82E-104	6	PTMA
4.52E-107	0.273529999	0.297	0.088	1.52E-102	6	ERICH1
4.74E-107	0.272562565	0.26	0.072	1.59E-102	6	IRF2
2.44E-106	0.285348311	0.296	0.089	8.17E-102	6	IDI1
6.76E-106	0.36767407	0.321	0.103	2.27E-101	6	DNAJB1

6.78E-106	0.282719926	0.459	0.171	2.27E-101	6	DEK
1.02E-105	0.253189968	0.251	0.068	3.43E-101	6	NUB1
8.63E-105	0.255340385	0.293	0.087	2.89E-100	6	MED28
6.70E-104	0.266184248	0.998	0.991	2.25E-99	6	RPL41
4.98E-103	0.289869732	0.289	0.087	1.67E-98	6	PPHLN1
4.80E-102	0.311222608	0.575	0.243	1.61E-97	6	TOMM20
6.52E-102	0.255142284	0.267	0.078	2.19E-97	6	DNAJB14
1.49E-101	0.328159775	0.984	0.851	4.99E-97	6	RPL38
2.64E-101	0.279653253	0.441	0.164	8.84E-97	6	RBMX
6.82E-101	0.297136966	0.348	0.118	2.29E-96	6	PRPF40A
1.68E-100	0.271956656	0.992	0.947	5.64E-96	6	RPS7
8.86E-100	0.329847487	0.984	0.856	2.97E-95	6	RPL27
2.30E-99	0.295775933	0.99	0.939	7.71E-95	6	RPL35A
2.81E-99	0.283549801	0.372	0.13	9.41E-95	6	CAPZA1
7.81E-98	0.306714135	0.35	0.121	2.62E-93	6	EAF2
1.31E-97	0.273101061	0.282	0.086	4.40E-93	6	PTPN1
1.88E-97	0.280894129	0.994	0.945	6.31E-93	6	RPS25
7.02E-96	0.271545711	0.395	0.145	2.35E-91	6	BBX
2.96E-95	0.263896802	0.332	0.112	9.94E-91	6	GOLGA4
8.26E-94	0.300695952	0.989	0.827	2.77E-89	6	ACTB
1.28E-92	0.292154756	0.986	0.878	4.29E-88	6	RPL22
7.34E-90	0.284285352	0.625	0.285	2.46E-85	6	SSR2
1.71E-87	0.307607334	0.392	0.15	5.75E-83	6	KLF6
5.66E-86	0.250687255	0.504	0.211	1.90E-81	6	ARPC5
9.09E-86	0.257573635	0.459	0.186	3.05E-81	6	SNRPB
2.80E-83	0.256166452	0.606	0.276	9.40E-79	6	UBXN1
4.50E-79	0.329447601	0.852	0.513	1.51E-74	6	HSP90AB1
2.24E-78	0.252778413	0.681	0.335	7.51E-74	6	SET
6.57E-78	0.274936313	0.598	0.282	2.20E-73	6	EIF4B
1.07E-72	0.302274845	0.906	0.563	3.58E-68	6	ARPC3
1.70E-72	0.30860605	0.906	0.587	5.69E-68	6	NPM1
2.55E-70	0.294312764	0.84	0.488	8.55E-66	6	CCNI
3.09E-64	0.283395213	0.906	0.591	1.04E-59	6	HMGB1
5.85E-63	0.272023952	0.965	0.757	1.96E-58	6	EEF1D
0	4.205863181	0.706	0.012	0	7	S100A9
0	4.130297916	0.604	0.009	0	7	S100A8
0	3.665138447	0.846	0.015	0	7	LYZ
0	3.403814557	0.903	0.024	0	7	TYROBP
0	3.150359782	0.901	0.013	0	7	AIF1
0	3.028957181	0.836	0.014	0	7	FCER1G

0	2.910372205	0.813	0.01	0	7	LST1
0	2.8201418	0.846	0.108	0	7	S100A4
0	2.574400565	0.831	0.06	0	7	CTSS
0	2.518761885	0.696	0.003	0	7	FCN1
0	2.513449031	0.725	0.069	0	7	FOS
0	2.447245811	0.768	0.077	0	7	LGALS1
0	2.39374507	0.571	0.043	0	7	TIMP1
0	2.229914163	0.486	0.003	0	7	AC020656.1
0	2.22674929	0.802	0.259	0	7	HLA-DRB1
0	2.201472254	0.781	0.082	0	7	SRGN
0	2.196955408	0.69	0.13	0	7	HLA-DPA1
0	2.175232157	0.994	0.44	0	7	TMSB4X
0	2.148291294	0.866	0.319	0	7	HLA-DRA
0	2.13041329	0.675	0.157	0	7	HLA-DPB1
0	2.121807822	0.97	0.554	0	7	CST3
0	2.030729029	0.902	0.517	0	7	CD74
0	2.01298951	0.27	0.013	0	7	C1QA
0	1.992782599	0.291	0	0	7	S100A12
0	1.97058926	0.888	0.296	0	7	PSAP
0	1.968993018	0.719	0.106	0	7	DUSP1
0	1.931860022	0.909	0.239	0	7	S100A6
0	1.83321868	0.51	0.014	0	7	IFI30
0	1.811022529	0.541	0.024	0	7	FGL2
0	1.808653608	0.451	0.01	0	7	CD14
0	1.807585241	0.731	0.129	0	7	TYMP
0	1.799603781	0.53	0.01	0	7	MNDA
0	1.798718089	0.496	0.006	0	7	CFD
0	1.780554412	0.591	0.123	0	7	IFITM2
0	1.770793827	0.453	0.097	0	7	HLA-DRB5
0	1.711329815	0.633	0.06	0	7	ITGB2
0	1.698162812	0.57	0.015	0	7	SPI1
0	1.671884343	0.504	0.083	0	7	HLA-DQB1
0	1.663946246	0.861	0.458	0	7	SH3BGRL3
0	1.662412591	0.339	0.005	0	7	VCAN
0	1.661309903	0.46	0.022	0	7	RGS2
0	1.63350657	0.455	0.009	0	7	MS4A6A
0	1.622754155	0.381	0.001	0	7	SMIM25
0	1.620952909	0.6	0.093	0	7	EMP3
0	1.564760647	0.551	0.036	0	7	PYCARD
0	1.541317674	0.42	0.004	0	7	MS4A7

0	1.529615532	0.504	0.02	0	7	C1orf162
0	1.526398801	0.614	0.086	0	7	GMFG
0	1.523903355	0.554	0.106	0	7	CEBPB
0	1.504824347	0.992	0.727	0	7	TMSB10
0	1.499905972	0.452	0.001	0	7	CSTA
0	1.485994362	0.911	0.186	0	7	NEAT1
0	1.485350637	0.988	0.828	0	7	ACTB
0	1.474700979	0.488	0.034	0	7	AP1S2
0	1.452216942	0.769	0.308	0	7	COTL1
0	1.452190895	0.459	0.068	0	7	NAMPT
0	1.44714815	0.436	0.026	0	7	SAMHD1
0	1.431681146	0.537	0.075	0	7	RNASET2
0	1.426272965	0.488	0.051	0	7	CD68
0	1.415763345	0.629	0.087	0	7	LAPTM5
0	1.411282109	0.32	0.013	0	7	FCGR3A
0	1.392289023	0.405	0.018	0	7	CYBB
0	1.376998095	0.468	0.035	0	7	CARD16
0	1.349085938	0.399	0.04	0	7	CPVL
0	1.34444514	0.637	0.171	0	7	JUNB
0	1.341124109	0.383	0.002	0	7	IGSF6
0	1.334109428	0.924	0.308	0	7	HLA-B
0	1.323744903	0.375	0.009	0	7	MPEG1
0	1.315429169	0.493	0.063	0	7	LCP1
0	1.297205055	0.456	0.094	0	7	STXBP2
0	1.282025018	0.488	0.103	0	7	HLA-DMA
0	1.26051242	0.419	0.046	0	7	ZEB2
0	1.25399462	0.503	0.114	0	7	ZFP36
0	1.25040638	0.339	0.006	0	7	MAFB
0	1.234628891	0.416	0.036	0	7	VSIR
0	1.220666807	0.376	0.018	0	7	JAML
0	1.214064112	0.35	0.001	0	7	CFP
0	1.213729152	0.534	0.072	0	7	HCST
0	1.190065577	0.32	0.005	0	7	PLAUR
0	1.170721878	0.384	0.043	0	7	MEF2C
0	1.165971471	0.327	0.016	0	7	PTPRE
0	1.161541625	0.479	0.079	0	7	FXYD5
0	1.137936239	0.303	0.003	0	7	CLEC12A
0	1.134089614	0.351	0.044	0	7	MARCKS
0	1.133989222	0.554	0.102	0	7	PTPRC
0	1.126235824	0.398	0.059	0	7	CD44

0	1.120476876	0.994	0.835	0	7	B2M
0	1.11420714	0.321	0.027	0	7	PLEK
0	1.110017502	0.342	0.006	0	7	TNFSF13B
0	1.107926788	0.335	0.003	0	7	CLEC7A
0	1.100287512	0.843	0.337	0	7	HLA-C
0	1.098808399	0.326	0.002	0	7	HCK
0	1.096996592	0.346	0.014	0	7	LY96
0	1.090587502	0.534	0.114	0	7	ARHGDIB
0	1.084696001	0.433	0.063	0	7	EFHD2
0	1.074261046	0.372	0.04	0	7	C4orf48
0	1.072815651	0.528	0.101	0	7	CORO1A
0	1.059609149	0.323	0.045	0	7	HLA-DMB
0	1.044636478	0.319	0.046	0	7	DUSP6
0	1.044009332	0.389	0.051	0	7	ANXA1
0	1.04256876	0.446	0.077	0	7	FYB1
0	1.036494747	0.324	0.026	0	7	TNFRSF1B
0	1.029895407	0.288	0.001	0	7	CSF1R
0	1.011543251	0.269	0	0	7	FPR1
0	1.004582092	0.358	0.032	0	7	GLIPR1
0	0.994735827	0.275	0.018	0	7	BCL2A1
0	0.983788412	0.29	0.009	0	7	FGR
0	0.982575334	0.263	0.001	0	7	LILRB2
0	0.978259983	0.301	0.026	0	7	NUP214
0	0.976274478	0.292	0.033	0	7	PLEKHO1
0	0.962862475	0.289	0.024	0	7	LYN
0	0.962091676	0.332	0.037	0	7	RGS10
0	0.960457996	0.382	0.066	0	7	GPSM3
0	0.959738227	0.333	0.041	0	7	PPT1
0	0.959208483	0.328	0.034	0	7	MYO1F
0	0.948201578	0.275	0.018	0	7	KCTD12
0	0.92505397	0.345	0.049	0	7	HCLS1
0	0.900542613	0.273	0.003	0	7	NCF2
0	0.863088131	0.278	0.029	0	7	CASP1
0	0.812955117	0.266	0.016	0	7	LGALS9
0	0.769502348	0.99	0.376	0	7	MALAT1
8.75E-305	1.62066131	0.359	0.061	2.93E-300	7	HLA-DQA1
1.72E-303	1.034003712	0.452	0.095	5.78E-299	7	GNAI2
1.66E-302	1.210724203	0.537	0.14	5.55E-298	7	GRN
1.01E-301	1.15136644	0.595	0.171	3.37E-297	7	ARPC1B
8.12E-296	1.184682617	0.486	0.113	2.72E-291	7	VMP1

1.28E-292	1.103192449	0.456	0.101	4.30E-288	7	ANXA2
9.82E-291	1.232674832	0.675	0.226	3.30E-286	7	VIM
1.47E-288	0.80324981	0.28	0.038	4.93E-284	7	ZYX
8.59E-287	1.043019453	0.401	0.079	2.88E-282	7	LSP1
2.31E-285	0.890375902	0.351	0.061	7.75E-281	7	RHOG
8.61E-283	1.088516862	0.302	0.045	2.89E-278	7	PLAC8
1.32E-278	0.752780503	0.452	0.092	4.43E-274	7	CD37
1.52E-278	1.210946826	0.868	0.519	5.09E-274	7	S100A11
6.62E-278	0.786120288	0.272	0.037	2.22E-273	7	ARRB2
7.56E-278	1.134379082	0.893	0.571	2.53E-273	7	CYBA
2.33E-273	0.913394676	0.289	0.042	7.81E-269	7	POU2F2
2.51E-262	1.13667859	0.486	0.113	8.43E-258	7	CD52
4.77E-261	0.870477812	0.318	0.054	1.60E-256	7	CNPY3
4.79E-261	1.056610422	0.353	0.066	1.61E-256	7	GLUL
1.57E-257	1.207477198	0.619	0.216	5.28E-253	7	TSPO
2.05E-256	0.865713184	0.27	0.039	6.89E-252	7	WARS
1.43E-250	0.807368227	0.31	0.052	4.80E-246	7	UCP2
8.86E-249	0.846940051	0.339	0.063	2.97E-244	7	EVI2B
6.67E-240	0.906893837	0.658	0.216	2.24E-235	7	HLA-E
3.89E-237	0.810739918	0.406	0.088	1.30E-232	7	CD48
2.03E-232	0.833105164	0.372	0.078	6.82E-228	7	CD53
4.91E-228	0.834595813	0.305	0.055	1.65E-223	7	TGFB1
1.24E-226	0.835518942	0.317	0.06	4.17E-222	7	BST2
1.02E-221	1.147703825	0.48	0.136	3.44E-217	7	IER2
1.23E-221	1.066845547	0.451	0.125	4.12E-217	7	CTSZ
6.42E-220	0.98347326	0.274	0.047	2.15E-215	7	NCF1
8.37E-220	0.643047055	0.98	0.919	2.81E-215	7	RPL28
8.83E-219	0.797797688	0.987	0.867	2.96E-214	7	RPL39
9.43E-208	0.86585104	0.795	0.377	3.16E-203	7	HLA-A
2.99E-207	0.932481879	0.396	0.099	1.00E-202	7	WSB1
3.89E-207	0.764001782	0.263	0.045	1.31E-202	7	IFNGR1
3.06E-204	1.263180629	0.466	0.139	1.03E-199	7	HSPA1A
4.14E-197	0.780683014	0.258	0.046	1.39E-192	7	FMNL1
3.73E-196	0.814592174	0.356	0.082	1.25E-191	7	LIMD2
6.34E-195	0.773995145	0.273	0.051	2.12E-190	7	RNF149
1.62E-193	0.733726727	0.309	0.064	5.42E-189	7	RNF130
2.19E-190	0.957773529	0.435	0.126	7.34E-186	7	PSMB9
5.69E-190	1.105552705	0.898	0.737	1.91E-185	7	SAT1
8.38E-190	0.704166044	0.27	0.05	2.81E-185	7	GIMAP4
5.91E-188	0.902042324	0.413	0.116	1.98E-183	7	LRRFIP1

5.43E-184	0.792431048	0.348	0.085	1.82E-179	7	FAM49B
5.81E-182	0.815558134	0.309	0.068	1.95E-177	7	PTPN6
2.76E-181	0.670720589	0.948	0.86	9.27E-177	7	RPS2
6.53E-180	1.000302632	0.418	0.124	2.19E-175	7	MCL1
5.39E-177	0.901798619	0.303	0.067	1.81E-172	7	AHNAK
2.20E-175	1.002500697	0.412	0.123	7.38E-171	7	VAMP5
4.44E-173	0.787188842	0.317	0.074	1.49E-168	7	IQGAP1
5.77E-173	0.733706389	0.898	0.761	1.93E-168	7	OAZ1
1.59E-172	0.819315127	0.266	0.053	5.35E-168	7	FOSB
2.06E-172	0.698096327	0.262	0.052	6.90E-168	7	FLNA
2.15E-167	0.952584018	0.536	0.212	7.19E-163	7	ARPC5
2.77E-163	0.800203891	0.364	0.1	9.28E-159	7	CMTM6
4.27E-163	0.95935094	0.723	0.404	1.43E-158	7	PABPC1
6.33E-162	0.865278353	0.423	0.135	2.12E-157	7	H2AFY
1.59E-161	0.75290482	0.281	0.063	5.34E-157	7	VASP
2.08E-157	0.722511514	0.263	0.057	6.99E-153	7	SMAP2
4.78E-151	0.861194947	0.634	0.284	1.60E-146	7	TXNIP
3.31E-148	0.765166742	0.955	0.91	1.11E-143	7	RPS19
3.21E-139	0.943772154	0.365	0.112	1.08E-134	7	HSPA1B
2.10E-138	0.594695616	0.93	0.818	7.05E-134	7	H3F3A
1.31E-137	0.521378249	0.959	0.904	4.40E-133	7	FAU
1.55E-131	0.718575836	0.298	0.08	5.21E-127	7	IFI16
5.58E-131	0.843227742	0.658	0.382	1.87E-126	7	ARPC2
3.26E-128	0.804610744	0.432	0.161	1.09E-123	7	ACTR2
4.16E-126	0.653625862	0.28	0.074	1.40E-121	7	TPP1
7.23E-126	0.590172357	0.931	0.907	2.43E-121	7	RPS9
7.46E-124	0.829687345	0.302	0.087	2.50E-119	7	IRF1
7.50E-124	0.93620004	0.511	0.223	2.51E-119	7	ZFP36L1
2.80E-123	0.754353379	0.827	0.622	9.38E-119	7	H3F3B
2.10E-122	0.659459502	0.272	0.072	7.05E-118	7	IFNGR2
2.08E-121	0.834224105	0.758	0.535	6.97E-117	7	NPC2
1.40E-120	0.75981677	0.291	0.083	4.68E-116	7	CCNL1
6.78E-116	0.72737373	0.3	0.089	2.28E-111	7	GCA
5.31E-114	0.889162181	0.69	0.408	1.78E-109	7	S100A10
1.04E-112	0.681781083	0.334	0.108	3.49E-108	7	LGALS3
3.42E-111	0.418163039	0.973	0.932	1.15E-106	7	RPS24
2.79E-110	0.914986243	0.26	0.072	9.35E-106	7	ISG15
3.30E-108	0.738603763	0.345	0.12	1.11E-103	7	CKLF
4.09E-107	0.792276814	0.483	0.217	1.37E-102	7	TKT
3.86E-100	0.686265231	0.306	0.1	1.29E-95	7	TUBA1A

1.22E-98	0.689246344	0.276	0.086	4.10E-94	7	NAGK
7.26E-96	0.796594374	0.382	0.152	2.43E-91	7	KLF6
6.87E-95	1.050111713	0.612	0.371	2.31E-90	7	IFITM3
2.31E-94	0.822727045	0.564	0.313	7.75E-90	7	NAP1L1
5.46E-93	0.688040088	0.458	0.21	1.83E-88	7	CAP1
5.54E-93	0.702554091	0.581	0.32	1.86E-88	7	SERP1
6.27E-89	0.673777754	0.517	0.257	2.10E-84	7	FCGRT
8.21E-86	0.599008797	0.745	0.572	2.75E-81	7	ARPC3
4.01E-85	0.694326899	0.468	0.228	1.35E-80	7	SDCBP
1.07E-84	0.474701755	0.598	0.315	3.59E-80	7	SERPINA1
1.55E-84	0.589741434	0.839	0.759	5.20E-80	7	PFN1
1.42E-81	0.397139631	0.953	0.943	4.78E-77	7	RPLP2
2.71E-81	0.625875409	0.707	0.471	9.09E-77	7	ACTG1
7.54E-80	0.57852462	0.266	0.09	2.53E-75	7	CASP4
8.64E-80	0.334159414	0.986	0.982	2.90E-75	7	RPLP1
9.32E-79	0.569035285	0.644	0.378	3.13E-74	7	DDX5
8.53E-78	0.647614693	0.47	0.239	2.86E-73	7	ATP6V0B
1.23E-77	0.510729097	0.255	0.084	4.11E-73	7	RAC2
2.94E-77	0.558424229	0.527	0.27	9.85E-73	7	BTG1
7.41E-75	0.669017418	0.454	0.229	2.49E-70	7	ANXA5
7.91E-75	0.745659982	0.395	0.18	2.65E-70	7	ZFP36L2
1.80E-74	0.556747607	0.329	0.133	6.03E-70	7	CAPZA1
5.68E-73	0.408130931	0.929	0.886	1.91E-68	7	UBA52
8.00E-73	0.61187776	0.355	0.153	2.68E-68	7	MTDH
1.57E-71	0.591350107	0.37	0.164	5.28E-67	7	ARL6IP5
5.22E-71	0.629948137	0.47	0.245	1.75E-66	7	TPM3
2.53E-70	0.569944531	0.318	0.131	8.48E-66	7	MTPN
3.21E-70	0.482151196	0.377	0.161	1.08E-65	7	JUND
1.57E-69	0.762835827	0.493	0.278	5.26E-65	7	CEBPD
4.46E-68	0.622830448	0.28	0.109	1.49E-63	7	MBNL1
3.73E-67	0.372586767	0.963	0.937	1.25E-62	7	RPS13
1.00E-65	0.577152448	0.29	0.116	3.36E-61	7	SERPINB1
2.46E-65	0.565989575	0.288	0.115	8.24E-61	7	TRA2B
1.91E-64	0.490513592	0.79	0.702	6.41E-60	7	CFL1
3.96E-64	0.631038576	0.564	0.362	1.33E-59	7	RHOA
1.88E-63	0.560988002	0.658	0.45	6.31E-59	7	LAMTOR4
2.49E-63	0.558927369	0.874	0.733	8.35E-59	7	RPS21
2.21E-62	0.389040602	0.917	0.821	7.41E-58	7	RPS11
6.76E-62	0.306323982	0.976	0.975	2.27E-57	7	RPS28
4.26E-61	0.350824576	0.931	0.919	1.43E-56	7	RPL18A

7.03E-61	0.53984898	0.401	0.201	2.36E-56	7	ACTR3
4.42E-59	0.664675192	0.419	0.222	1.48E-54	7	HNRNPU
5.07E-59	0.557460598	0.26	0.103	1.70E-54	7	RSRP1
1.74E-58	0.537218338	0.368	0.178	5.82E-54	7	ARPC4
6.33E-58	0.323185891	0.945	0.899	2.12E-53	7	PTMA
1.93E-57	0.296204444	0.978	0.981	6.47E-53	7	RPL10
5.28E-57	0.465437781	0.322	0.142	1.77E-52	7	JUN
1.56E-56	0.762709127	0.473	0.275	5.24E-52	7	CTSB
4.16E-53	0.527237293	0.275	0.119	1.39E-48	7	EIF3A
3.66E-52	0.300276148	0.967	0.931	1.23E-47	7	ATP5F1E
5.23E-52	0.521215047	0.266	0.114	1.76E-47	7	TLN1
1.80E-51	0.340350642	0.913	0.916	6.02E-47	7	RPL27A
4.09E-51	0.283355272	0.937	0.881	1.37E-46	7	RPL30
4.67E-51	0.555727373	0.347	0.173	1.57E-46	7	MSN
5.85E-51	0.297575746	0.968	0.951	1.96E-46	7	RPS8
6.02E-51	0.312741558	0.898	0.824	2.02E-46	7	RPL18
3.67E-50	0.653462803	0.456	0.272	1.23E-45	7	TUBA1B
6.71E-49	0.382334704	0.869	0.834	2.25E-44	7	PFDN5
1.48E-48	0.415690934	0.255	0.108	4.95E-44	7	OSTF1
6.42E-48	0.447566025	0.253	0.108	2.15E-43	7	ATG3
9.08E-48	0.664393488	0.37	0.199	3.04E-43	7	CTSC
1.81E-47	0.595723953	0.305	0.148	6.05E-43	7	DDX3X
4.91E-47	0.330351161	0.964	0.959	1.65E-42	7	RPS27
4.20E-45	0.490348766	0.404	0.225	1.41E-40	7	YWHAZ
4.82E-45	0.489311112	0.496	0.312	1.62E-40	7	FKBP1A
7.16E-45	0.537892572	0.3	0.147	2.40E-40	7	HNRNPH1
1.30E-43	0.484244297	0.428	0.254	4.37E-39	7	SH3BGRL
1.05E-42	0.705523816	0.298	0.146	3.52E-38	7	HMOX1
1.83E-42	0.501664716	0.303	0.151	6.15E-38	7	ZFAND5
2.04E-42	0.438618849	0.3	0.149	6.84E-38	7	PSMB8
2.19E-42	0.292890666	0.952	0.962	7.35E-38	7	RPS14
6.52E-42	0.280107644	0.945	0.947	2.19E-37	7	RPS15A
1.15E-41	0.304670827	0.94	0.89	3.85E-37	7	RPS29
3.76E-40	0.464296271	0.529	0.358	1.26E-35	7	AP2S1
1.88E-39	0.541262718	0.388	0.23	6.32E-35	7	PTP4A2
2.07E-39	0.493221915	0.436	0.273	6.94E-35	7	ASAH1
1.28E-38	0.412155369	0.29	0.146	4.30E-34	7	GNB2
5.77E-38	0.521251824	0.3	0.156	1.93E-33	7	SCAF11
1.50E-36	0.45457681	0.257	0.126	5.03E-32	7	PAK2
5.50E-35	0.400447234	0.734	0.637	1.85E-30	7	CALM2

4.15E-34	0.498013189	0.29	0.155	1.39E-29	7	DDX21
1.14E-33	0.515694961	0.341	0.196	3.83E-29	7	SRRM2
4.54E-33	0.378035584	0.748	0.618	1.52E-28	7	RPS10
6.86E-33	0.411348999	0.463	0.306	2.30E-28	7	DAZAP2
1.61E-32	0.432092798	0.255	0.129	5.40E-28	7	TSC22D3
6.53E-32	0.724079521	0.435	0.289	2.19E-27	7	CTSD
7.29E-32	0.419008224	0.368	0.219	2.44E-27	7	GDI2
9.59E-31	0.520318544	0.534	0.389	3.22E-26	7	CSTB
1.92E-30	0.363818759	0.401	0.248	6.43E-26	7	PKM
2.85E-30	0.349187535	0.262	0.136	9.56E-26	7	IFI27L2
3.18E-30	0.36960237	0.629	0.475	1.07E-25	7	RPL36A
6.39E-30	0.447023296	0.294	0.163	2.14E-25	7	FUS
2.09E-29	0.525536719	0.278	0.155	7.03E-25	7	RNF213
3.25E-29	0.393747116	0.354	0.214	1.09E-24	7	RBM3
3.61E-29	0.498225097	0.289	0.165	1.21E-24	7	NFKBIA
1.72E-27	0.312425417	0.254	0.134	5.75E-23	7	ITGB1
1.12E-26	0.377428343	0.49	0.347	3.77E-22	7	PNRC1
1.52E-26	0.378432566	0.39	0.254	5.09E-22	7	TMBIM4
5.43E-26	0.330648809	0.334	0.2	1.82E-21	7	N4BP2L2
5.48E-26	0.456597569	0.487	0.356	1.84E-21	7	PSME2
9.99E-26	0.348373831	0.429	0.285	3.35E-21	7	CAPZB
1.59E-25	0.385706713	0.579	0.444	5.34E-21	7	CDC42
4.80E-25	0.325547258	0.377	0.238	1.61E-20	7	APLP2
4.81E-24	0.363019425	0.283	0.166	1.61E-19	7	CFLAR
4.93E-24	0.320611751	0.286	0.167	1.65E-19	7	TMEM50A
3.01E-23	0.357739493	0.297	0.177	1.01E-18	7	RAP1A
3.67E-23	0.37744431	0.253	0.146	1.23E-18	7	LSM6
1.59E-22	0.346013133	0.366	0.238	5.34E-18	7	RBM39
4.85E-22	0.280021162	0.661	0.539	1.63E-17	7	RAC1
1.57E-21	0.342254149	0.335	0.216	5.28E-17	7	PGLS
3.75E-21	0.345029895	0.259	0.154	1.26E-16	7	LITAF
3.81E-21	0.316327154	0.285	0.168	1.28E-16	7	PNISR
8.85E-21	0.289073058	0.257	0.151	2.97E-16	7	ATP6AP2
4.10E-20	0.322605904	0.29	0.179	1.37E-15	7	DEK
4.34E-20	0.298034532	0.272	0.165	1.46E-15	7	CAPNS1
1.76E-19	0.356624252	0.35	0.238	5.90E-15	7	HNRNPA3
7.95E-19	0.322067235	0.495	0.38	2.67E-14	7	YWHAB
1.06E-18	0.328728636	0.315	0.207	3.55E-14	7	MOB1A
1.33E-18	0.365761123	0.306	0.196	4.46E-14	7	DDX17
1.34E-18	0.341793363	0.256	0.157	4.50E-14	7	LSM8

5.09E-18	0.326817858	0.413	0.294	1.71E-13	7	SON
5.96E-18	0.36102857	0.368	0.253	2.00E-13	7	GNAS
1.24E-17	0.317884357	0.466	0.346	4.16E-13	7	ZFAS1
4.47E-17	0.311444975	0.352	0.244	1.50E-12	7	LSM7
2.71E-16	0.309014322	0.301	0.202	9.09E-12	7	CAST
3.21E-16	0.307240642	0.381	0.275	1.08E-11	7	DRAP1
3.50E-16	0.314791794	0.268	0.173	1.17E-11	7	ANP32B
2.47E-15	0.310786844	0.587	0.485	8.29E-11	7	HNRNPA2B1
3.62E-15	0.264679997	0.44	0.332	1.21E-10	7	HMGN2
7.21E-15	0.305862206	0.983	0.982	2.42E-10	7	FTH1
9.66E-15	0.295532969	0.544	0.454	3.24E-10	7	BRI3
2.35E-14	0.264645565	0.573	0.486	7.88E-10	7	CLIC1
1.14E-13	0.300952911	0.315	0.221	3.81E-09	7	TMEM167A
1.48E-13	0.254839507	0.348	0.248	4.97E-09	7	RNH1
2.41E-13	0.323695708	0.327	0.237	8.09E-09	7	TAGLN2
1.35E-12	0.299419231	0.303	0.217	4.51E-08	7	ARF6
1.40E-12	0.291701433	0.503	0.424	4.69E-08	7	PRELID1
3.77E-12	0.344571424	0.319	0.237	1.26E-07	7	LY6E
3.82E-12	0.25184015	0.326	0.237	1.28E-07	7	RAP1B
1.14E-11	0.250190411	0.429	0.338	3.82E-07	7	SEC11A
1.34E-09	0.333922384	0.326	0.254	4.50E-05	7	RHOB
3.66E-09	0.27648195	0.571	0.511	0.000122788	7	CD63
1.24E-06	0.25713321	0.287	0.226	0.041605493	7	DNAJA1
0	3.579269191	0.856	0.131	0	8	DEFB1
0	3.508956601	0.424	0.012	0	8	UMOD
0	3.056198883	0.45	0.025	0	8	S100A2
0	2.733392912	0.337	0.006	0	8	SLC12A1
0	2.522304128	0.491	0.005	0	8	KNG1
0	2.360342237	0.907	0.248	0	8	S100A6
0	2.291210966	0.348	0.005	0	8	TMEM52B
0	2.044006471	0.646	0.111	0	8	CKB
0	1.784749385	0.45	0.003	0	8	DUSP9
0	1.731663616	0.287	0.008	0	8	SFRP1
0	1.727850423	0.502	0.051	0	8	GSTM3
0	1.571625434	0.275	0.003	0	8	PVALB
0	1.42969981	0.253	0.002	0	8	CLDN16
0	1.348943401	0.287	0.004	0	8	KCNJ1
0	1.312222938	0.311	0.021	0	8	MAL
0	1.305459812	0.323	0.018	0	8	UCHL1
0	1.278984266	0.262	0.002	0	8	TUBB2B

0	1.228550855	0.29	0.015	0	8	FOLR3
9.01E-224	1.684396613	0.508	0.117	3.02E-219	8	PPP1R1A
3.41E-222	1.199832702	0.343	0.052	1.15E-217	8	NDRG2
3.80E-175	1.165079526	0.366	0.074	1.28E-170	8	NUDT4
1.67E-130	1.301698913	0.537	0.206	5.58E-126	8	IDH2
8.85E-126	1.147676381	0.71	0.377	2.97E-121	8	CD24
1.32E-121	1.253333585	0.422	0.131	4.41E-117	8	NFE2L2
1.43E-107	1.162590061	0.481	0.184	4.81E-103	8	MRPS6
1.38E-98	1.24425489	0.439	0.167	4.63E-94	8	SLC25A4
2.40E-98	1.021729393	0.694	0.412	8.06E-94	8	S100A10
8.46E-93	0.983240192	0.694	0.425	2.84E-88	8	BEX3
3.18E-90	0.969794993	0.359	0.116	1.07E-85	8	EFHD1
3.50E-86	1.020455744	0.407	0.148	1.17E-81	8	COX7A1
4.61E-83	1.190765924	0.27	0.074	1.54E-78	8	CA12
2.77E-80	1.035516138	0.509	0.247	9.29E-76	8	PKM
3.22E-71	0.774317597	0.804	0.652	1.08E-66	8	CHCHD10
2.38E-70	1.031762937	0.595	0.348	8.00E-66	8	MPC1
8.43E-68	0.681520479	0.842	0.724	2.83E-63	8	COX8A
8.84E-65	0.883478733	0.295	0.101	2.97E-60	8	COMT
7.35E-62	0.647540149	0.882	0.832	2.47E-57	8	COX5B
5.18E-60	0.635803192	0.831	0.71	1.74E-55	8	UQCRH
4.91E-58	0.586140732	0.825	0.651	1.65E-53	8	DSTN
5.33E-58	1.603437848	0.481	0.277	1.79E-53	8	ATP1A1
7.26E-58	0.764856777	0.759	0.637	2.43E-53	8	CALM2
3.33E-56	0.795536972	0.753	0.598	1.12E-51	8	CYSTM1
3.50E-56	0.97786647	0.467	0.252	1.17E-51	8	GNAS
2.22E-55	1.754354581	0.287	0.107	7.45E-51	8	WFDC2
1.04E-51	0.739428872	0.557	0.339	3.48E-47	8	MRPL33
1.29E-49	0.877529221	0.623	0.456	4.34E-45	8	CYCS
1.86E-49	0.713185171	0.697	0.539	6.24E-45	8	ATP5MC1
9.82E-46	0.546491095	0.823	0.727	3.29E-41	8	COX7B
5.42E-44	1.027593969	0.612	0.452	1.82E-39	8	ATP1B1
8.44E-44	0.844220139	0.275	0.113	2.83E-39	8	IVNS1ABP
1.57E-43	0.803335503	0.264	0.107	5.27E-39	8	ITGB1BP1
3.65E-39	0.437434072	0.865	0.838	1.22E-34	8	SRP14
1.03E-38	0.580251919	0.825	0.801	3.45E-34	8	COX6B1
4.24E-38	0.632830169	0.621	0.461	1.42E-33	8	NDUFA5
1.60E-37	0.586026407	0.762	0.683	5.35E-33	8	PPDPF
1.77E-37	0.851140182	0.439	0.269	5.95E-33	8	CBR1
4.61E-37	0.806076525	0.349	0.178	1.55E-32	8	BEX2

2.05E-35	0.713535786	0.379	0.211	6.87E-31	8	DNAJC19
3.31E-35	0.960272906	0.54	0.39	1.11E-30	8	IGFBP7
1.47E-30	0.350996216	0.898	0.9	4.93E-26	8	PTMA
2.34E-30	0.839854861	0.467	0.322	7.85E-26	8	HIST1H4C
3.98E-30	0.499566937	0.795	0.74	1.33E-25	8	ATP5PF
8.41E-30	0.663605928	0.346	0.19	2.82E-25	8	AIF1L
1.75E-29	0.530130842	0.668	0.558	5.86E-25	8	ATP5F1B
9.58E-29	0.419291569	0.823	0.802	3.21E-24	8	UQCRB
1.02E-28	0.525122842	0.668	0.557	3.42E-24	8	ATP5F1A
1.34E-28	0.463913131	0.795	0.754	4.51E-24	8	COX6C
1.67E-28	0.44484876	0.87	0.864	5.61E-24	8	NDUFA4
2.69E-28	0.537712087	0.626	0.513	9.02E-24	8	NDUFB3
1.15E-27	0.748126351	0.388	0.242	3.87E-23	8	MOCS2
1.24E-27	0.486608914	0.789	0.727	4.15E-23	8	NDUFA1
1.45E-27	0.493600919	0.795	0.771	4.85E-23	8	COX7A2
1.17E-26	0.609173068	0.326	0.179	3.93E-22	8	PFN2
1.69E-26	0.670961595	0.37	0.228	5.68E-22	8	NDUFA8
2.30E-26	0.474108039	0.806	0.746	7.73E-22	8	UQCR10
3.44E-26	0.555726462	0.641	0.541	1.15E-21	8	COX5A
1.42E-25	0.526368827	0.7	0.642	4.75E-21	8	ATP5PO
1.36E-24	0.612851638	0.398	0.254	4.55E-20	8	HIGD1A
1.45E-24	0.466987906	0.797	0.756	4.86E-20	8	ATP5MC3
3.41E-24	0.457682797	0.624	0.477	1.15E-19	8	ACTG1
9.80E-24	0.381669607	0.87	0.912	3.29E-19	8	EIF1
2.65E-23	0.55297206	0.481	0.35	8.88E-19	8	SMS
1.38E-22	0.628316456	0.429	0.303	4.63E-18	8	VDAC2
1.74E-22	0.618085875	0.36	0.228	5.83E-18	8	DMAC1
4.96E-22	0.473901546	0.668	0.586	1.66E-17	8	PRDX2
8.24E-22	0.602414557	0.474	0.357	2.76E-17	8	CYC1
1.06E-21	0.461020146	0.615	0.525	3.55E-17	8	HSP90AB1
1.69E-21	0.541173352	0.388	0.25	5.67E-17	8	NARS
5.21E-21	0.632419122	0.325	0.2	1.75E-16	8	ARHGAP24
5.44E-21	0.426256855	0.776	0.746	1.82E-16	8	TMA7
1.12E-20	0.614500173	0.284	0.163	3.75E-16	8	SELENOM
1.47E-20	0.590797792	0.388	0.255	4.94E-16	8	TCEAL4
1.92E-20	0.527527006	0.329	0.203	6.44E-16	8	PDHA1
1.11E-19	0.345406139	0.856	0.824	3.74E-15	8	LDHB
1.40E-19	0.430290606	0.699	0.659	4.71E-15	8	YBX1
1.74E-19	0.368784858	0.832	0.85	5.83E-15	8	ATP5MG
1.89E-19	0.57038212	0.334	0.209	6.34E-15	8	SVIP

2.21E-19	0.548697483	0.525	0.422	7.41E-15	8	NDUFA6
2.74E-19	0.579858537	0.312	0.196	9.17E-15	8	ANXA11
2.95E-19	0.513174135	0.402	0.282	9.89E-15	8	SNRPN
3.09E-19	0.544791266	0.41	0.283	1.04E-14	8	PNKD
3.60E-19	0.54131382	0.551	0.459	1.21E-14	8	MRPL41
4.16E-19	0.575043156	0.337	0.218	1.39E-14	8	SRI
6.05E-19	0.385074174	0.731	0.679	2.03E-14	8	PRDX5
6.47E-19	0.474007788	0.526	0.422	2.17E-14	8	HSBP1
2.80E-17	0.49597931	0.52	0.425	9.38E-13	8	C19orf70
5.73E-17	0.365825833	0.641	0.579	1.92E-12	8	ENO1
1.83E-16	0.5350381	0.419	0.315	6.13E-12	8	NDUFA12
2.09E-16	0.432580053	0.57	0.485	7.00E-12	8	HMGN1
2.32E-16	0.414660547	0.747	0.708	7.80E-12	8	UQCR11
2.87E-16	0.58367018	0.357	0.257	9.63E-12	8	GLRX5
6.10E-16	0.378077421	0.734	0.734	2.04E-11	8	CHCHD2
1.22E-15	0.3163773	0.429	0.293	4.10E-11	8	TXNIP
1.72E-15	0.673004277	0.266	0.162	5.76E-11	8	TSC22D1
2.30E-15	0.509902748	0.264	0.162	7.70E-11	8	MAGED2
4.18E-15	0.510759683	0.266	0.166	1.40E-10	8	C7orf50
4.98E-15	0.433435502	0.649	0.599	1.67E-10	8	PRR13
3.31E-14	0.251952875	0.896	0.845	1.11E-09	8	PEBP1
4.02E-14	0.406474543	0.579	0.509	1.35E-09	8	NDUFB9
5.47E-14	0.380369308	0.745	0.729	1.83E-09	8	SKP1
5.88E-14	0.464179164	0.38	0.279	1.97E-09	8	HADHB
9.51E-14	0.462822895	0.598	0.541	3.19E-09	8	NDUFS6
1.78E-13	0.398138549	0.557	0.502	5.99E-09	8	CCNI
1.87E-13	0.441788906	0.5	0.427	6.27E-09	8	NDUFAB1
3.86E-13	0.533026424	0.301	0.208	1.29E-08	8	FAM200B
5.34E-13	0.313129606	0.675	0.629	1.79E-08	8	H3F3B
6.49E-13	0.481012746	0.561	0.519	2.18E-08	8	CIRBP
8.16E-13	0.386332847	0.635	0.596	2.74E-08	8	NDUFB7
1.57E-12	0.386272513	0.595	0.526	5.27E-08	8	PRDX3
1.59E-12	0.389876298	0.634	0.61	5.33E-08	8	SUB1
1.83E-12	0.288148537	0.778	0.744	6.15E-08	8	UBL5
2.39E-12	0.453703426	0.345	0.244	8.02E-08	8	TCEAL9
4.12E-12	0.298009464	0.663	0.606	1.38E-07	8	MGST3
5.01E-12	0.433539219	0.491	0.429	1.68E-07	8	ERH
1.23E-11	0.36348541	0.498	0.42	4.13E-07	8	VDAC1
1.72E-11	0.262959758	0.762	0.75	5.76E-07	8	ATP5F1D
1.83E-11	0.390343907	0.64	0.589	6.15E-07	8	ATP5PD

1.99E-11	0.446491465	0.29	0.203	6.68E-07	8	CXXC5
2.52E-11	0.451141475	0.526	0.477	8.46E-07	8	UQCRFS1
2.79E-11	0.35923479	0.578	0.526	9.36E-07	8	MPC2
3.23E-11	0.292861766	0.713	0.661	1.08E-06	8	NDUFB1
3.33E-11	0.339517902	0.693	0.7	1.12E-06	8	SLC25A6
4.25E-11	0.340081913	0.644	0.614	1.42E-06	8	COMMD6
7.80E-11	0.4648626	0.467	0.396	2.62E-06	8	SNU13
7.97E-11	0.363398742	0.584	0.532	2.67E-06	8	MINOS1
8.03E-11	0.510854839	0.343	0.259	2.69E-06	8	ID2
1.17E-10	0.443355102	0.396	0.315	3.93E-06	8	MMP24OS
1.25E-10	0.332650926	0.652	0.623	4.18E-06	8	NDUFB4
1.37E-10	0.488239554	0.315	0.231	4.59E-06	8	UQCC2
1.47E-10	0.501906351	0.297	0.211	4.93E-06	8	CD59
2.17E-10	0.473686301	0.323	0.248	7.29E-06	8	VAMP2
2.51E-10	0.488823903	0.314	0.236	8.41E-06	8	ANXA5
3.49E-10	0.476428525	0.346	0.269	1.17E-05	8	C12orf57
4.74E-10	0.547187518	0.276	0.196	1.59E-05	8	CHP1
5.45E-10	0.394880553	0.77	0.743	1.83E-05	8	FXYD2
7.67E-10	0.449675472	0.671	0.674	2.57E-05	8	SLC25A5
1.01E-09	0.255525948	0.75	0.757	3.38E-05	8	PPIA
1.60E-09	0.363871414	0.446	0.368	5.38E-05	8	NAA38
2.12E-09	0.426016692	0.352	0.276	7.12E-05	8	HIBADH
2.52E-09	0.274213628	0.744	0.727	8.44E-05	8	MIF
2.99E-09	0.434383186	0.45	0.395	0.000100387	8	EID1
2.99E-09	0.514496023	0.259	0.187	0.000100398	8	MRPL36
3.54E-09	0.489205639	0.474	0.397	0.000118693	8	ADIRF
6.58E-09	0.444438422	0.393	0.329	0.00022061	8	NDUFS4
7.71E-09	0.374567559	0.266	0.191	0.000258631	8	SNX10
8.29E-09	0.436996109	0.335	0.265	0.000277977	8	BAG1
9.24E-09	0.439075893	0.269	0.198	0.000309871	8	LYPLA1
1.30E-08	0.398202994	0.483	0.434	0.000436927	8	GABARAPL2
1.59E-08	0.370693565	0.267	0.192	0.000532506	8	BEX4
1.59E-08	0.416080493	0.331	0.259	0.000532706	8	SH3BGRL
1.73E-08	0.444297764	0.266	0.195	0.000579315	8	VDAC3
2.20E-08	0.329148898	0.547	0.501	0.000736775	8	GNG5
2.39E-08	0.379074471	0.561	0.541	0.000800859	8	NDUFB10
2.93E-08	0.405734624	0.405	0.338	0.00098313	8	TMEM141
3.68E-08	0.362634569	0.494	0.447	0.001234527	8	TSTD1
3.71E-08	0.407024793	0.267	0.197	0.001243945	8	EIF4E
3.81E-08	0.392685606	0.346	0.284	0.001276304	8	UQCRC2

4.58E-08	0.803255036	0.318	0.26	0.001536474	8	MTRNR2L12
5.88E-08	0.288942522	0.86	0.928	0.001970787	8	RPS4X
7.03E-08	0.351534182	0.416	0.354	0.002357899	8	NUCKS1
1.09E-07	0.296469946	0.531	0.483	0.003665083	8	YWHAE
2.15E-07	0.28127669	0.637	0.613	0.007221364	8	EIF3K
3.74E-07	0.288089784	0.564	0.516	0.012553062	8	PRDX6
3.87E-07	0.39557025	0.373	0.313	0.012965235	8	NCL
5.71E-07	0.299336493	0.543	0.516	0.01916104	8	NDUFV2
5.86E-07	0.364665709	0.301	0.234	0.019638363	8	HSPA9
6.44E-07	0.320201481	0.47	0.429	0.021587753	8	SRP9
6.70E-07	0.493679434	0.278	0.216	0.022473718	8	GABARAPL1
7.24E-07	0.367859	0.413	0.358	0.024272585	8	FAM162A
8.45E-07	0.457928971	0.376	0.33	0.028325068	8	SELENOW
9.63E-07	0.285365328	0.651	0.642	0.032294605	8	ISCU
1.49E-06	0.367630227	0.323	0.266	0.0498227	8	RAB11A
2.60E-06	0.379412462	0.401	0.356	0.08733945	8	C11orf58
2.98E-06	0.279097247	0.57	0.546	0.099931197	8	ATP5F1C
3.37E-06	0.37360946	0.272	0.214	0.112979182	8	MRPS12
4.49E-06	0.286381656	0.576	0.592	0.150696104	8	SUMO2
6.40E-06	0.518861043	0.457	0.397	0.214785595	8	MALAT1
1.14E-05	0.254404339	0.629	0.628	0.381416718	8	DYNLL1
1.49E-05	0.323314107	0.359	0.314	0.500018971	8	TAX1BP1
1.58E-05	0.354662024	0.475	0.458	0.530041892	8	ANAPC16
1.69E-05	0.326778321	0.497	0.489	0.56783862	8	HNRNPA2B1
1.69E-05	0.330424375	0.407	0.369	0.568016991	8	SRSF9
1.73E-05	0.379049097	0.328	0.286	0.581779614	8	PPCS
1.78E-05	0.352633534	0.388	0.347	0.596713864	8	COX20
2.10E-05	0.358127196	0.325	0.282	0.7054004	8	RHEB
2.74E-05	0.336951876	0.281	0.232	0.918309902	8	PDCD6
3.04E-05	0.341539291	0.366	0.332	1	8	TMEM230
3.13E-05	0.368057869	0.331	0.286	1	8	AP2M1
3.15E-05	0.323832908	0.413	0.377	1	8	NDUFB5
3.30E-05	0.335291332	0.346	0.301	1	8	ANAPC13
3.65E-05	0.292533027	0.262	0.211	1	8	MRPS7
3.75E-05	0.348705598	0.43	0.407	1	8	COX7A2L
4.92E-05	0.332459739	0.283	0.236	1	8	HDDC2
5.45E-05	0.315092804	0.275	0.22	1	8	MIR4458HG
5.79E-05	0.521570263	0.326	0.294	1	8	CTSD
6.38E-05	0.271685803	0.467	0.433	1	8	CISD1
7.96E-05	0.282796218	0.356	0.313	1	8	SRSF3

0.000112377	0.278185472	0.508	0.492	1	8	NDUFS7
0.000141056	0.315247299	0.306	0.258	1	8	TXNL4A
0.000155338	0.289102518	0.425	0.394	1	8	HINT2
0.000161256	0.269829025	0.416	0.38	1	8	NDUFB6
0.000188365	0.326419194	0.317	0.282	1	8	YWHAQ
0.000204746	0.327086974	0.325	0.285	1	8	TMEM14B
0.000210091	0.339663985	0.359	0.325	1	8	RAB2A
0.000217925	0.278659452	0.269	0.223	1	8	MIEN1
0.000261881	0.267246699	0.399	0.371	1	8	ATP5PB
0.000303848	0.312417772	0.363	0.334	1	8	POLR2K
0.000304218	0.294620271	0.306	0.267	1	8	MRFAP1
0.000331417	0.356274691	0.311	0.274	1	8	DDX24
0.000357565	0.319279931	0.298	0.263	1	8	BANF1
0.000370557	0.293541616	0.307	0.267	1	8	NDUFA10
0.000379422	0.347295273	0.264	0.225	1	8	BOLA3
0.000383314	0.285269958	0.284	0.242	1	8	MAP3K13
0.000388443	0.314219537	0.286	0.247	1	8	PYURF
0.000417314	0.256610959	0.328	0.286	1	8	ALDH1A1
0.000426742	0.271520472	0.259	0.216	1	8	MRPS18C
0.000579272	0.281795246	0.306	0.269	1	8	NDUFS3
0.000581755	0.296498005	0.283	0.245	1	8	MAP1LC3B
0.000713218	0.257697444	0.424	0.394	1	8	CSTB
0.00101492	0.299728557	0.43	0.417	1	8	HNRNPK
0.001037946	0.324124714	0.261	0.227	1	8	SPAG7
0.001169043	0.261347141	0.5	0.495	1	8	SEC61G
0.001489611	0.281104131	0.27	0.234	1	8	MRPL21
0.001515708	0.351241807	0.321	0.299	1	8	SRSF5
0.001596452	0.272223041	0.488	0.49	1	8	CLIC1
0.001743439	0.307234673	0.284	0.254	1	8	TCEAL8
0.001826074	0.299203398	0.261	0.23	1	8	TSPO
0.002594633	0.311054865	0.276	0.248	1	8	AP1S1
0.003335829	0.269706081	0.373	0.356	1	8	SDHD
0.003482337	0.308429959	0.363	0.35	1	8	SET
0.003537188	0.282824549	0.331	0.31	1	8	EIF2S2
0.003558978	0.261090261	0.425	0.423	1	8	SUMO1
0.004561693	0.26820947	0.256	0.224	1	8	MRPS16
0.004928486	0.255850617	0.272	0.242	1	8	ECI1
0.005305449	0.333101357	0.256	0.228	1	8	PPP1CB
0.005413638	0.30318051	0.303	0.288	1	8	CNBP
0.005447978	0.252325636	0.312	0.283	1	8	NHP2

0.006274836	0.260769223	0.404	0.402	1	8	CAPZA2
0.007061447	0.26130288	0.252	0.225	1	8	STRAP
0.007990203	0.313137035	0.28	0.26	1	8	KRT10
0.008606933	0.274540163	0.256	0.234	1	8	RANBP1
0.009593695	0.27104447	0.368	0.359	1	8	VAPA
0.009756584	0.291712421	0.345	0.334	1	8	UFC1
0	3.213296659	0.471	0.013	0	9	FXYD4
0	3.146953467	0.443	0.007	0	9	AQP2
0	2.528301103	0.264	0.005	0	9	SLPI
0	2.350528286	0.257	0.001	0	9	SLC26A7
0	2.145716999	0.421	0.041	0	9	AQP3
0	2.076048658	0.41	0.007	0	9	TMEM213
0	2.043736416	0.476	0.022	0	9	HSD11B2
0	1.82491485	0.329	0.002	0	9	ATP6V1G3
0	1.771748722	0.574	0.019	0	9	MAL
0	1.724928548	0.479	0.01	0	9	MUC1
0	1.723437125	0.371	0.008	0	9	TACSTD2
0	1.72220704	0.512	0.045	0	9	CD9
0	1.71029067	0.407	0.02	0	9	KRT19
0	1.651972601	0.49	0.009	0	9	SCIN
0	1.529118881	0.31	0.003	0	9	ATP6V0D2
0	1.430252927	0.283	0.014	0	9	KRT7
0	1.242457988	0.288	0.006	0	9	NUPR2
0	1.1985239	0.343	0.002	0	9	CLDN8
0	1.19621051	0.257	0.006	0	9	CLCNKB
0	1.191153997	0.283	0.012	0	9	PTGER3
0	1.12506332	0.367	0.027	0	9	MAL2
0	1.053142943	0.298	0.002	0	9	ADGRF1
0	1.045027518	0.288	0.006	0	9	CDH1
0	0.951692222	0.321	0.014	0	9	DMKN
0	0.923532893	0.257	0.007	0	9	MECOM
0	0.828097151	0.255	0.01	0	9	SCNN1A
6.74E-300	2.319514252	0.736	0.139	2.26E-295	9	DEFB1
2.30E-260	1.371592427	0.371	0.037	7.72E-256	9	TFCP2L1
4.32E-256	2.000836607	0.881	0.254	1.45E-251	9	S100A6
5.07E-251	1.077990737	0.279	0.021	1.70E-246	9	DSP
7.43E-243	1.380080261	0.414	0.05	2.49E-238	9	COBLL1
6.70E-236	1.978637084	0.576	0.103	2.25E-231	9	WFDC2
6.79E-214	1.648692251	0.6	0.121	2.28E-209	9	CDH16
4.55E-184	1.615051333	0.536	0.11	1.52E-179	9	LGALS3

7.84E-156	0.94020953	0.283	0.035	2.63E-151	9	PLS3
4.32E-154	0.803838161	0.29	0.036	1.45E-149	9	PAX2
3.64E-139	1.612875734	0.833	0.392	1.22E-134	9	MALAT1
4.43E-139	0.844829917	0.257	0.032	1.48E-134	9	SLC5A3
2.97E-134	0.925060086	0.286	0.041	9.97E-130	9	SLC38A1
9.60E-133	0.719091283	0.252	0.032	3.22E-128	9	S100A2
1.47E-131	1.149927601	0.41	0.083	4.92E-127	9	MUC20-OT1
6.69E-122	1.152201536	0.269	0.04	2.24E-117	9	SLC43A2
1.11E-113	1.238187254	0.557	0.174	3.71E-109	9	KTN1
7.35E-108	1.117455205	0.283	0.049	2.46E-103	9	PLCG2
1.09E-105	1.386606244	0.35	0.075	3.64E-101	9	CA12
1.80E-105	1.258926019	0.781	0.387	6.05E-101	9	IGFBP7
2.17E-102	0.835976679	0.629	0.207	7.27E-98	9	NEAT1
6.94E-102	0.992832441	0.426	0.107	2.33E-97	9	CD81
4.71E-99	1.253342832	0.445	0.119	1.58E-94	9	CKB
9.08E-98	1.36689048	0.464	0.133	3.04E-93	9	NFE2L2
1.89E-95	0.684875243	0.269	0.047	6.33E-91	9	JUP
2.53E-92	0.781813516	0.336	0.072	8.48E-88	9	AHNAK
2.12E-91	1.009710087	0.402	0.105	7.10E-87	9	WSB1
2.30E-89	0.877002017	0.29	0.058	7.72E-85	9	GSTM3
1.54E-87	1.252344173	0.457	0.142	5.17E-83	9	RALBP1
4.03E-87	0.866708567	0.267	0.051	1.35E-82	9	ARHGAP18
7.02E-86	0.979998734	0.362	0.089	2.35E-81	9	COL18A1
1.09E-84	1.259864586	0.314	0.072	3.65E-80	9	WNK1
1.20E-79	0.809425514	0.321	0.075	4.02E-75	9	CLDN7
2.12E-79	1.042922321	0.5	0.173	7.12E-75	9	EPCAM
1.41E-78	0.944401842	0.298	0.067	4.74E-74	9	MT-ATP8
4.72E-78	1.758394855	0.586	0.259	1.58E-73	9	CLU
6.36E-78	1.03779878	0.59	0.248	2.13E-73	9	PKM
6.59E-77	1.226961217	0.367	0.102	2.21E-72	9	NDRG1
8.15E-77	0.993902403	0.729	0.379	2.73E-72	9	CD24
4.21E-76	1.082994788	0.348	0.094	1.41E-71	9	DHRS7
9.26E-76	0.898607558	0.457	0.146	3.10E-71	9	BCAM
2.33E-75	0.870372714	0.5	0.172	7.81E-71	9	APP
3.97E-75	0.942113191	0.4	0.117	1.33E-70	9	AKAP9
1.14E-70	0.532262236	0.862	0.454	3.81E-66	9	TMSB4X
1.29E-69	1.283126795	0.595	0.277	4.33E-65	9	ATP1A1
2.73E-66	0.878891371	0.307	0.081	9.16E-62	9	MSI2
6.90E-66	1.143793104	0.755	0.451	2.31E-61	9	ATP1B1
1.40E-64	0.765225299	0.264	0.062	4.69E-60	9	CAPN2

8.64E-63	0.896266644	0.329	0.095	2.90E-58	9	ATP6V1A
2.31E-61	0.616252035	0.312	0.084	7.74E-57	9	SPTBN1
7.68E-61	0.68559164	0.262	0.064	2.57E-56	9	GLTP
1.72E-58	0.875900635	0.31	0.088	5.78E-54	9	NEDD4L
1.88E-58	0.953039914	0.479	0.189	6.31E-54	9	AIF1L
2.34E-58	0.828829558	0.56	0.252	7.85E-54	9	GNAS
3.59E-58	0.767776838	0.771	0.475	1.20E-53	9	ACTG1
2.95E-57	0.798799564	0.402	0.138	9.88E-53	9	SYNE2
3.71E-57	0.826502704	0.39	0.132	1.24E-52	9	PAX8
2.46E-56	1.047309789	0.288	0.082	8.24E-52	9	AKR1B1
3.65E-56	0.606644691	0.257	0.065	1.22E-51	9	PTBP3
4.09E-54	0.760450377	0.712	0.414	1.37E-49	9	S100A10
6.39E-54	0.837907087	0.795	0.527	2.14E-49	9	S100A11
8.46E-54	0.859371945	0.476	0.196	2.84E-49	9	DDX17
2.70E-53	0.751547027	0.274	0.075	9.05E-49	9	GOLGB1
6.15E-53	0.737230652	0.324	0.101	2.06E-48	9	CD46
2.66E-52	0.67647793	0.267	0.073	8.91E-48	9	HACD3
2.17E-51	0.85617843	0.288	0.086	7.29E-47	9	OGDHL
1.56E-50	0.923777222	0.4	0.154	5.24E-46	9	LITAF
2.13E-50	0.813067744	0.707	0.434	7.14E-46	9	RTN4
3.31E-50	0.901740981	0.395	0.151	1.11E-45	9	ATP6AP2
1.38E-49	0.768948186	0.505	0.225	4.62E-45	9	ZBTB20
7.75E-49	0.868025329	0.569	0.291	2.60E-44	9	CTSD
2.13E-48	0.698928729	0.271	0.08	7.14E-44	9	MACF1
4.78E-48	0.640957329	0.933	0.823	1.60E-43	9	MT-ND3
7.39E-48	0.711745148	0.267	0.078	2.48E-43	9	TPM4
6.64E-45	0.719253061	0.638	0.343	2.23E-40	9	CAMK2N1
1.50E-44	0.793171486	0.648	0.383	5.04E-40	9	SEC62
1.83E-44	0.697864081	0.286	0.091	6.15E-40	9	CYFIP2
9.27E-44	0.735234424	0.305	0.103	3.11E-39	9	NORAD
2.49E-43	1.025811574	0.564	0.322	8.34E-39	9	HIST1H4C
1.18E-41	0.573678179	0.36	0.135	3.96E-37	9	ITGB1
1.70E-40	0.658368893	0.352	0.136	5.71E-36	9	TGOLN2
2.63E-40	0.877221128	0.66	0.413	8.81E-36	9	MT-ND4L
5.37E-40	0.670147075	0.255	0.08	1.80E-35	9	RTN3
1.40E-39	0.686894322	0.4	0.167	4.70E-35	9	EMX2
4.86E-39	0.62947699	0.574	0.292	1.63E-34	9	TXNIP
1.52E-38	0.781632945	0.49	0.24	5.11E-34	9	APLP2
3.22E-38	0.750363148	0.488	0.244	1.08E-33	9	ATP6V0B
4.99E-38	0.510454349	0.293	0.101	1.67E-33	9	PTTG1IP

5.54E-38	0.943055924	0.502	0.258	1.86E-33	9	MTRNR2L12
6.19E-37	0.64232585	0.286	0.101	2.08E-32	9	HIPK2
9.05E-37	0.629487087	0.431	0.198	3.04E-32	9	SRRM2
1.50E-36	0.580910189	0.407	0.176	5.05E-32	9	CAPG
3.26E-34	0.7883928	0.29	0.111	1.09E-29	9	ANXA2
4.14E-34	0.589780578	0.276	0.098	1.39E-29	9	KCNJ16
1.07E-33	0.614069527	0.388	0.169	3.60E-29	9	PNISR
1.19E-33	0.587249355	0.269	0.095	3.98E-29	9	PPP1R14B
1.54E-33	0.720374344	0.414	0.198	5.16E-29	9	SPINT2
2.44E-33	0.566395033	0.271	0.098	8.17E-29	9	TBC1D1
3.22E-33	0.574749934	0.298	0.112	1.08E-28	9	MYO6
4.67E-33	0.54370251	0.262	0.093	1.57E-28	9	PABPN1
2.98E-32	0.483901876	0.555	0.314	1.00E-27	9	PSAP
2.58E-30	0.535707175	0.298	0.118	8.66E-26	9	NAP1L4
2.97E-30	0.521586157	0.34	0.144	9.97E-26	9	SDC4
3.00E-30	0.68861497	0.326	0.138	1.01E-25	9	SNRNP70
3.73E-30	0.564783479	0.305	0.124	1.25E-25	9	LRRFIP1
7.35E-30	0.65023554	0.571	0.341	2.46E-25	9	MRPL33
6.05E-29	0.56364796	0.407	0.195	2.03E-24	9	ANXA11
7.11E-29	0.531666623	0.262	0.099	2.38E-24	9	SYNJ2BP
2.01E-26	0.487061243	0.267	0.105	6.74E-22	9	TMEM123
7.69E-26	0.615789989	0.343	0.162	2.58E-21	9	GTF2I
1.11E-25	0.546068988	0.329	0.15	3.73E-21	9	HNRNPH1
1.52E-25	0.815966071	0.319	0.151	5.09E-21	9	COX7A1
7.05E-25	0.531414772	0.707	0.524	2.36E-20	9	HSP90AB1
1.08E-24	0.369360368	0.91	0.801	3.63E-20	9	MT-ND1
1.43E-24	0.695004484	0.333	0.159	4.81E-20	9	MT-ND6
1.62E-24	0.563376541	0.26	0.109	5.44E-20	9	RBM25
1.76E-24	0.519338512	0.34	0.162	5.91E-20	9	TSC22D1
2.10E-24	0.555271493	0.371	0.189	7.04E-20	9	MRPS6
3.76E-24	0.665789793	0.393	0.212	1.26E-19	9	IDH2
6.71E-24	0.604663419	0.49	0.295	2.25E-19	9	FAM107B
9.41E-24	0.572766466	0.331	0.156	3.16E-19	9	SFPQ
3.26E-23	0.557494636	0.26	0.111	1.09E-18	9	NCOR1
3.31E-23	0.530662044	0.426	0.23	1.11E-18	9	EIF5B
5.79E-23	0.522607595	0.31	0.146	1.94E-18	9	LUC7L3
4.77E-22	0.481856642	0.495	0.295	1.60E-17	9	SON
7.90E-22	0.517903176	0.262	0.117	2.65E-17	9	ATRX
8.18E-22	0.536432455	0.262	0.114	2.74E-17	9	IVNS1ABP
1.68E-21	0.556757764	0.269	0.121	5.63E-17	9	EPS8

3.74E-20	0.490473422	0.326	0.164	1.26E-15	9	SELENOM
4.28E-20	0.553385903	0.262	0.119	1.44E-15	9	GOLGA4
4.56E-20	0.580555057	0.462	0.279	1.53E-15	9	BSG
5.00E-20	0.423063843	0.543	0.328	1.68E-15	9	HLA-B
7.84E-20	0.459010469	0.3	0.144	2.63E-15	9	RBM47
1.07E-19	0.548892267	0.429	0.255	3.60E-15	9	C9orf16
1.49E-19	0.516311135	0.343	0.18	5.01E-15	9	PFN2
3.69E-19	0.479218669	0.326	0.167	1.24E-14	9	AES
9.15E-19	0.523833589	0.34	0.181	3.07E-14	9	METAP2
1.04E-18	0.288943638	0.338	0.167	3.50E-14	9	JUND
1.27E-18	0.451428592	0.279	0.132	4.26E-14	9	ADGRG1
1.50E-18	0.522212306	0.29	0.146	5.04E-14	9	TTC3
1.72E-18	0.499919299	0.462	0.282	5.78E-14	9	HSP90B1
1.93E-18	0.442339947	0.369	0.202	6.46E-14	9	N4BP2L2
3.12E-18	0.367148251	0.267	0.124	1.05E-13	9	NIPSNAP2
3.25E-18	0.371150902	0.257	0.117	1.09E-13	9	AP2B1
8.98E-17	0.336335851	0.581	0.389	3.01E-12	9	HLA-A
2.62E-16	0.414770498	0.307	0.165	8.79E-12	9	HP1BP3
5.28E-16	0.280525683	0.279	0.138	1.77E-11	9	IFITM2
6.32E-16	0.371302645	0.274	0.137	2.12E-11	9	HDLBP
6.69E-16	0.405832342	0.529	0.353	2.24E-11	9	NUCKS1
7.16E-16	0.433417268	0.329	0.181	2.40E-11	9	TMCO1
7.30E-16	0.414603925	0.26	0.129	2.45E-11	9	PRRC2C
8.47E-16	0.519605777	0.374	0.227	2.84E-11	9	HNRNPU
8.59E-16	0.261194086	0.943	0.906	2.88E-11	9	MT-CO1
1.68E-15	0.441065232	0.386	0.234	5.62E-11	9	PTP4A2
3.36E-15	0.305545995	0.274	0.138	1.13E-10	9	LMNA
4.32E-15	0.396207206	0.431	0.271	1.45E-10	9	TPM1
6.64E-15	0.432874527	0.367	0.219	2.23E-10	9	CANX
1.22E-14	0.368949722	0.381	0.228	4.10E-10	9	PRKAR1A
1.40E-14	0.434676436	0.348	0.202	4.68E-10	9	ARL6IP1
1.60E-14	0.324273508	0.26	0.131	5.38E-10	9	PRPF4B
1.70E-14	0.464000345	0.312	0.177	5.69E-10	9	SRSF11
2.59E-14	0.283673744	0.838	0.661	8.68E-10	9	MT-ND5
3.24E-14	0.380816738	0.257	0.131	1.09E-09	9	ELF3
5.11E-14	0.409050926	0.283	0.153	1.71E-09	9	CSNK1A1
6.67E-14	0.435743292	0.371	0.231	2.24E-09	9	ZFP36L1
5.75E-13	0.395588072	0.383	0.24	1.93E-08	9	HNRNPA3
6.66E-13	0.334855315	0.269	0.145	2.23E-08	9	HNRNPD
1.27E-12	0.315247651	0.555	0.385	4.25E-08	9	DDX5

1.29E-12	0.31239292	0.276	0.152	4.32E-08	9	ACTN4
1.36E-12	0.379906191	0.371	0.226	4.57E-08	9	PPP1CB
2.97E-12	0.272307154	0.957	0.915	9.94E-08	9	MT-CO2
5.31E-12	0.432463768	0.431	0.297	1.78E-07	9	SRSF5
1.14E-11	0.337994809	0.283	0.162	3.84E-07	9	DPM3
1.27E-11	0.31846689	0.388	0.247	4.25E-07	9	VAMP2
1.92E-11	0.293819438	0.255	0.139	6.43E-07	9	CD151
2.10E-11	0.399170918	0.326	0.199	7.04E-07	9	LAMP1
2.51E-11	0.335719597	0.693	0.567	8.42E-07	9	HSP90AA1
2.85E-11	0.307216166	0.536	0.393	9.55E-07	9	CSTB
6.39E-11	0.343576594	0.305	0.185	2.14E-06	9	PDIA3
8.68E-11	0.347043647	0.357	0.229	2.91E-06	9	TSPO
2.50E-10	0.262981835	0.267	0.153	8.37E-06	9	GRN
2.98E-10	0.325015252	0.255	0.147	9.98E-06	9	MRPS5
3.70E-10	0.285619114	0.621	0.482	1.24E-05	9	YWHAE
4.71E-10	0.314270229	0.345	0.219	1.58E-05	9	SRI
4.72E-10	0.260864135	0.329	0.203	1.58E-05	9	LEPROT
4.74E-10	0.351398339	0.262	0.154	1.59E-05	9	PNRC2
6.47E-10	0.34780483	0.286	0.174	2.17E-05	9	RBMX
7.08E-10	0.372216407	0.276	0.169	2.37E-05	9	TMED10
7.58E-10	0.295975947	0.402	0.27	2.54E-05	9	PET100
8.23E-10	0.371399002	0.319	0.204	2.76E-05	9	CAST
8.91E-10	0.266265937	0.262	0.154	2.99E-05	9	WDR1
1.16E-09	0.356735878	0.267	0.16	3.89E-05	9	HSPA5
1.26E-09	0.30275002	0.274	0.165	4.23E-05	9	HNRNPM
1.50E-09	0.251467276	0.329	0.204	5.03E-05	9	PDHA1
1.85E-09	0.348528361	0.302	0.189	6.19E-05	9	HIF1A
5.57E-09	0.311820443	0.29	0.184	0.000186946	9	TRAM1
6.35E-09	0.254272085	0.381	0.257	0.000213133	9	TCEAL4
6.95E-09	0.304731886	0.624	0.487	0.000233238	9	HNRNPA2B1
7.99E-09	0.294035317	0.371	0.249	0.000267837	9	DYNC1I2
1.09E-08	0.305758713	0.414	0.285	0.000365595	9	ALDH1A1
1.13E-08	0.326877575	0.5	0.364	0.000378538	9	KRT8
1.21E-08	0.32694634	0.269	0.167	0.000407486	9	CAPNS1
1.45E-08	0.298877493	0.536	0.391	0.000485612	9	KRT18
1.60E-08	0.279631321	0.621	0.511	0.000535806	9	CD63
1.99E-08	0.395915128	0.364	0.256	0.000667723	9	HIGD1A
2.49E-08	0.319116203	0.312	0.204	0.000833636	9	CXXC5
3.11E-08	0.304774077	0.321	0.214	0.001042289	9	OCIAD1
3.60E-08	0.290925613	0.317	0.21	0.001208718	9	SVIP

5.80E-08	0.276802709	0.643	0.531	0.001945684	9	MINOS1
1.10E-07	0.398796168	0.252	0.162	0.003684479	9	CLTC
2.12E-07	0.720194826	0.533	0.445	0.007106831	9	C12orf75
4.01E-07	0.301084112	0.36	0.252	0.01343269	9	EZR
4.97E-07	0.261817469	0.267	0.173	0.016661661	9	DLD
8.03E-07	0.308122752	0.324	0.23	0.026918409	9	YWHAZ
9.11E-07	0.340148211	0.371	0.277	0.030558101	9	ASAH1
1.28E-06	0.2590176	0.367	0.265	0.042948191	9	BAG1
1.37E-06	0.292398336	0.305	0.214	0.045982055	9	JPT1
3.29E-06	0.30540965	0.376	0.28	0.110441724	9	HADHB
4.23E-06	0.270072213	0.276	0.193	0.141790996	9	REEP5
7.88E-06	0.254739474	0.579	0.498	0.264268437	9	COX17
1.21E-05	0.253167044	0.407	0.313	0.406143931	9	NCL
7.14E-05	0.297385592	0.274	0.202	1	9	ARHGAP24
0.000110378	0.320632308	0.379	0.306	1	9	CA2
0.000191259	0.297217157	0.679	0.64	1	9	CALM2
0.001122467	0.315653868	0.331	0.264	1	9	SLC25A39
0	4.0747594	0.777	0.017	0	10	IGFBP5
0	3.044177468	0.813	0.032	0	10	IFI27
0	2.807201171	0.702	0.032	0	10	TIMP3
0	2.779772629	0.693	0.004	0	10	EMCN
0	2.682744005	0.735	0.022	0	10	SLC9A3R2
0	2.634000325	0.687	0.006	0	10	RNASE1
0	2.55871841	0.765	0.053	0	10	SPARC
0	2.529171198	0.605	0.002	0	10	PLVAP
0	2.494351974	0.69	0.011	0	10	ENG
0	2.427063918	0.738	0.027	0	10	EGFL7
0	2.395107151	0.642	0.017	0	10	ID1
0	2.378922668	0.705	0.018	0	10	PECAM1
0	2.35834952	0.602	0.002	0	10	FLT1
0	2.325588648	0.705	0.043	0	10	EPAS1
0	2.319210047	0.578	0.008	0	10	HSPG2
0	2.278758679	0.614	0.034	0	10	TGFBR2
0	2.278498089	0.771	0.079	0	10	SPTBN1
0	2.27291759	0.521	0.005	0	10	PLAT
0	2.251309998	0.642	0.012	0	10	A2M
0	2.224616877	0.639	0.001	0	10	PTPRB
0	2.210299429	0.681	0.025	0	10	GNG11
0	2.156718181	0.608	0.002	0	10	SOX18
0	2.123650544	0.373	0.017	0	10	MGP

0	2.122437242	0.614	0.007	0	10	ADGRF5
0	2.121914909	0.623	0.005	0	10	MEIS2
0	2.113889836	0.569	0.003	0	10	RAMP3
0	2.047601812	0.642	0.062	0	10	GSN
0	2.017288973	0.569	0.027	0	10	GBP4
0	2.010359514	0.584	0.028	0	10	PLPP3
0	2.001779636	0.587	0.041	0	10	WARS
0	1.954691625	0.554	0.019	0	10	HYAL2
0	1.953512366	0.569	0.017	0	10	UACA
0	1.940563155	0.536	0.016	0	10	CEACAM1
0	1.883788921	0.506	0.001	0	10	KDR
0	1.872360611	0.587	0.004	0	10	PODXL
0	1.865314358	0.467	0.007	0	10	FN1
0	1.856343418	0.518	0.003	0	10	SLCO2A1
0	1.826943983	0.542	0.003	0	10	RAMP2
0	1.821960854	0.512	0.037	0	10	PLPP1
0	1.796702264	0.509	0.001	0	10	ADGRL4
0	1.77884928	0.521	0.001	0	10	CD34
0	1.774171893	0.419	0.007	0	10	DNASE1L3
0	1.77370683	0.533	0.008	0	10	HEG1
0	1.770090888	0.337	0.005	0	10	CRHBP
0	1.76698141	0.611	0.069	0	10	AHNAK
0	1.741173185	0.521	0.014	0	10	GAS6
0	1.658727427	0.467	0.008	0	10	CD93
0	1.649870868	0.509	0.006	0	10	NPDC1
0	1.642358245	0.536	0.048	0	10	ID3
0	1.627902549	0.449	0.006	0	10	TM4SF1
0	1.623190753	0.521	0.011	0	10	CAVIN1
0	1.620048587	0.509	0.021	0	10	LMO2
0	1.618342608	0.482	0.015	0	10	EHD4
0	1.60225929	0.497	0.035	0	10	TM4SF18
0	1.597664404	0.419	0.011	0	10	TCIM
0	1.590845733	0.458	0.001	0	10	TEK
0	1.588687644	0.506	0.039	0	10	TCF4
0	1.569983746	0.467	0.004	0	10	LIMS2
0	1.525804949	0.512	0.034	0	10	CRIP2
0	1.481629849	0.443	0.004	0	10	CAVIN2
0	1.470990827	0.383	0.011	0	10	EFNB1
0	1.469429378	0.536	0.051	0	10	GIMAP7
0	1.461183361	0.437	0.033	0	10	XAF1

0	1.456566418	0.434	0.001	0	10	MMRN2
0	1.441614325	0.346	0.004	0	10	NES
0	1.438701651	0.431	0.01	0	10	VWA1
0	1.432775846	0.428	0.001	0	10	CDH5
0	1.414504225	0.452	0.03	0	10	TGM2
0	1.413374482	0.431	0.019	0	10	LIMCH1
0	1.403189465	0.358	0.001	0	10	EXOC3L2
0	1.393037194	0.367	0.002	0	10	FAM167B
0	1.378778814	0.38	0.008	0	10	ADAM15
0	1.375278867	0.41	0.001	0	10	ROBO4
0	1.36269462	0.389	0.001	0	10	CLEC14A
0	1.356800434	0.398	0.002	0	10	SHANK3
0	1.353113923	0.383	0.003	0	10	NOTCH4
0	1.322655593	0.386	0.004	0	10	FXYD6
0	1.319460166	0.398	0.017	0	10	CALCRL
0	1.307558181	0.355	0.004	0	10	LRRC32
0	1.295329996	0.416	0.024	0	10	BMPR2
0	1.291978145	0.367	0.001	0	10	PDE2A
0	1.290641835	0.413	0.016	0	10	ELK3
0	1.290463812	0.319	0.002	0	10	EFNB2
0	1.284546805	0.434	0.026	0	10	KIAA0355
0	1.280632778	0.38	0.016	0	10	PRSS23
0	1.273740035	0.377	0.002	0	10	LDB2
0	1.266416806	0.373	0.007	0	10	DLC1
0	1.2564731	0.34	0.015	0	10	ZEB1
0	1.249878772	0.355	0.003	0	10	TMTC1
0	1.245965038	0.331	0.007	0	10	PLXND1
0	1.240937224	0.416	0.019	0	10	TIMP2
0	1.237097716	0.361	0.02	0	10	LIFR
0	1.220349634	0.346	0.002	0	10	ACVRL1
0	1.208431828	0.392	0.004	0	10	ESAM
0	1.200611388	0.355	0.013	0	10	PPM1F
0	1.190430631	0.395	0.019	0	10	ECE1
0	1.161585365	0.349	0.005	0	10	CASKIN2
0	1.143870637	0.298	0.001	0	10	JCAD
0	1.139254913	0.343	0.002	0	10	KANK3
0	1.131246092	0.34	0.011	0	10	APOL1
0	1.125826995	0.34	0.001	0	10	ERG
0	1.102291916	0.346	0.002	0	10	TIE1
0	1.09446742	0.313	0.006	0	10	GIMAP8

0	1.07960199	0.337	0.016	0	10	GIMAP6
0	1.078713604	0.328	0.006	0	10	PMP22
0	1.076213686	0.319	0.001	0	10	RASIP1
0	1.071553335	0.313	0.009	0	10	TMEM204
0	1.064283719	0.271	0.007	0	10	NPR1
0	1.062710239	0.286	0	0	10	ARHGEF15
0	1.052301062	0.289	0.002	0	10	TMEM88
0	1.02730923	0.325	0.019	0	10	PTPRM
0	1.027061604	0.334	0.019	0	10	EVA1B
0	1.009899788	0.298	0.004	0	10	EMP2
0	1.009335494	0.295	0.007	0	10	STAB1
0	1.006308625	0.304	0.001	0	10	SHE
0	0.996137484	0.298	0.001	0	10	ADCY4
0	0.992147266	0.271	0.006	0	10	ITGA5
0	0.990341346	0.253	0.004	0	10	EML1
0	0.985103551	0.313	0.001	0	10	ECSCR
0	0.954734368	0.256	0.005	0	10	DOCK6
0	0.952765612	0.277	0.01	0	10	TNS2
0	0.941068692	0.259	0.002	0	10	PALMD
0	0.931517686	0.292	0.006	0	10	EPHB4
0	0.90715913	0.277	0.012	0	10	RASAL2
0	0.901178002	0.256	0.004	0	10	GRASP
0	0.899628855	0.262	0.006	0	10	TLR4
0	0.893504331	0.262	0.003	0	10	SEMA3F
0	0.890337804	0.256	0.009	0	10	FGD5
0	0.847820387	0.277	0.008	0	10	CYYR1
1.12E-302	1.71383884	0.494	0.047	3.74E-298	10	CD9
3.14E-295	1.511216851	0.449	0.039	1.05E-290	10	COL4A1
1.19E-293	1.76442939	0.614	0.077	3.98E-289	10	IL6ST
2.02E-291	1.050957858	0.355	0.024	6.79E-287	10	HDAC7
4.69E-284	1.567198956	0.533	0.058	1.57E-279	10	UTRN
4.98E-277	1.247820158	0.364	0.027	1.67E-272	10	IFI44L
1.13E-273	1.607456604	0.539	0.063	3.78E-269	10	BST2
4.92E-271	1.538359324	0.527	0.06	1.65E-266	10	TACC1
4.23E-270	0.885397108	0.277	0.015	1.42E-265	10	F2R
2.54E-269	1.602223695	0.587	0.076	8.50E-265	10	MACF1
1.10E-266	0.800872402	0.256	0.013	3.70E-262	10	FZD4
6.08E-266	0.967181268	0.31	0.02	2.04E-261	10	ASAP1
6.66E-262	0.963503422	0.298	0.019	2.23E-257	10	FLNB
5.18E-261	1.288699138	0.494	0.052	1.74E-256	10	GIMAP4

8.25E-254	1.426220866	0.464	0.049	2.77E-249	10	SEC14L1
1.70E-252	1.327832855	0.44	0.043	5.70E-248	10	DPYSL2
8.38E-247	1.579189094	0.533	0.067	2.81E-242	10	PBX1
8.52E-244	1.153066896	0.416	0.04	2.86E-239	10	LAMB2
1.36E-240	1.237108927	0.422	0.042	4.56E-236	10	PIK3C2A
5.49E-239	1.771428953	0.708	0.131	1.84E-234	10	ITGB1
1.08E-237	1.189353884	0.37	0.033	3.61E-233	10	SLK
4.50E-234	2.146642201	0.849	0.225	1.51E-229	10	HLA-E
3.89E-233	1.653540413	0.639	0.106	1.31E-228	10	CD81
1.88E-232	1.455729195	0.518	0.066	6.30E-228	10	HLA-DQA1
6.67E-231	1.910924442	0.476	0.057	2.24E-226	10	SYNE1
1.85E-230	1.31879507	0.322	0.025	6.20E-226	10	RGCC
3.04E-227	1.159324988	0.355	0.031	1.02E-222	10	MYO1C
3.29E-227	0.992696752	0.331	0.027	1.10E-222	10	ICAM2
1.16E-225	1.331276835	0.449	0.051	3.89E-221	10	ETS1
1.26E-225	1.131216186	0.304	0.023	4.22E-221	10	SLC44A2
3.14E-224	0.813319011	0.262	0.017	1.05E-219	10	APOL3
5.29E-224	1.128649553	0.358	0.032	1.77E-219	10	SERPINH1
6.06E-222	1.310417846	0.482	0.059	2.03E-217	10	NOSTRIN
4.57E-219	0.943178901	0.298	0.023	1.53E-214	10	IFI44
3.99E-218	1.414212983	0.464	0.056	1.34E-213	10	ETS2
1.23E-213	1.902299757	0.687	0.135	4.14E-209	10	SYNE2
9.48E-208	1.074381984	0.389	0.04	3.18E-203	10	LIMA1
5.07E-207	1.639708135	0.428	0.051	1.70E-202	10	MARCKS
6.31E-207	0.969556631	0.319	0.027	2.12E-202	10	PTPRG
2.91E-198	2.02744851	0.907	0.324	9.76E-194	10	HLA-B
6.24E-196	1.324096425	0.44	0.056	2.09E-191	10	TRIM56
2.21E-195	0.999008359	0.307	0.027	7.40E-191	10	ITGA1
5.09E-191	1.365780557	0.461	0.063	1.71E-186	10	PARP14
7.83E-191	0.88115714	0.301	0.026	2.63E-186	10	GIMAP1
4.79E-190	1.095748095	0.358	0.037	1.61E-185	10	SLFN5
5.35E-188	1.130054799	0.401	0.047	1.80E-183	10	MEF2A
3.30E-186	1.414982197	0.545	0.09	1.11E-181	10	ARHGAP29
1.38E-185	1.14063861	0.383	0.044	4.64E-181	10	COL4A2
4.10E-183	1.175980703	0.413	0.051	1.37E-178	10	MEF2C
7.37E-180	0.86278086	0.256	0.02	2.47E-175	10	PIEZO1
7.45E-178	1.177285904	0.557	0.093	2.50E-173	10	HLA-DQB1
1.35E-177	1.733822126	0.69	0.17	4.53E-173	10	APP
3.35E-176	1.476116212	0.575	0.108	1.12E-171	10	ANXA2
2.98E-175	0.877224064	0.295	0.028	9.99E-171	10	PLEC

4.85E-175	1.724704702	0.825	0.272	1.63E-170	10	HLA-DRB1
1.04E-171	0.83861772	0.271	0.024	3.50E-167	10	RRAS
2.97E-171	1.426653902	0.461	0.071	9.95E-167	10	SH3BP5
2.94E-170	1.427586051	0.488	0.076	9.85E-166	10	KLF2
2.07E-169	0.942473336	0.316	0.033	6.94E-165	10	TSPAN14
8.70E-169	0.967595635	0.28	0.026	2.92E-164	10	SULF2
1.06E-163	0.947980964	0.31	0.033	3.57E-159	10	MAP4K4
7.65E-163	1.720773818	0.967	0.526	2.57E-158	10	CD74
8.95E-163	0.854146874	0.265	0.024	3.00E-158	10	GOLM1
3.32E-162	1.384764158	0.777	0.206	1.11E-157	10	NEAT1
2.65E-161	1.431379627	0.834	0.257	8.89E-157	10	S100A6
4.70E-158	0.837537109	0.31	0.033	1.58E-153	10	TRIOBP
7.77E-157	0.78709862	0.274	0.026	2.61E-152	10	CD109
7.65E-153	0.821008737	0.256	0.024	2.57E-148	10	TAP2
1.79E-152	0.893329212	0.319	0.036	6.00E-148	10	TJP1
3.38E-151	1.30450117	0.53	0.103	1.13E-146	10	GNAI2
3.95E-151	1.13635927	0.422	0.063	1.33E-146	10	PPFIBP1
8.27E-150	1.365264761	0.515	0.099	2.77E-145	10	PRCP
9.88E-150	0.760848958	0.268	0.026	3.31E-145	10	HTRA1
1.52E-149	0.962527415	0.361	0.047	5.10E-145	10	AP1S2
1.48E-148	1.647629645	0.889	0.386	4.95E-144	10	HLA-A
4.37E-143	0.897554326	0.31	0.037	1.47E-138	10	SUN1
3.29E-142	1.0495317	0.401	0.059	1.10E-137	10	ANXA1
4.82E-136	1.040528816	0.392	0.061	1.62E-131	10	QKI
6.40E-135	1.023734785	0.377	0.057	2.15E-130	10	SERINC3
2.48E-134	0.902597171	0.304	0.037	8.31E-130	10	IL13RA1
7.12E-134	1.05918308	0.334	0.045	2.39E-129	10	NFAT5
1.17E-133	1.180611539	0.476	0.09	3.93E-129	10	MYH9
2.81E-133	0.873090134	0.304	0.037	9.43E-129	10	SORBS2
1.98E-132	1.490366034	0.834	0.35	6.65E-128	10	HLA-C
3.87E-130	1.145604703	0.491	0.099	1.30E-125	10	PTTG1IP
8.59E-130	1.067295975	0.44	0.078	2.88E-125	10	HLA-F
5.94E-129	0.904931477	0.316	0.042	1.99E-124	10	BAZ2B
3.18E-128	0.82349979	0.34	0.048	1.07E-123	10	PHACTR2
8.11E-128	1.21329947	0.596	0.145	2.72E-123	10	HLA-DPA1
1.78E-126	1.436010387	0.63	0.179	5.96E-122	10	IGFBP4
4.64E-126	1.693121378	0.666	0.207	1.56E-121	10	CD59
9.40E-125	0.882318913	0.28	0.034	3.15E-120	10	MPRIP
3.19E-124	0.957513836	0.301	0.039	1.07E-119	10	GBP1
5.92E-123	0.909255104	0.361	0.055	1.99E-118	10	CRIM1

7.67E-123	1.221625529	0.44	0.083	2.57E-118	10	NR2F2
2.70E-122	0.86634786	0.325	0.046	9.05E-118	10	ZNF503
3.21E-122	0.903318115	0.265	0.031	1.08E-117	10	RXRA
7.39E-120	0.976344637	0.367	0.059	2.48E-115	10	SPTAN1
8.35E-119	1.084611699	0.38	0.065	2.80E-114	10	IFI6
5.17E-118	0.972226829	0.367	0.06	1.73E-113	10	GNAQ
5.68E-117	0.967683578	0.392	0.067	1.91E-112	10	WWTR1
3.64E-114	0.877046786	0.274	0.035	1.22E-109	10	ROCK2
6.47E-114	1.43292801	0.377	0.067	2.17E-109	10	CCND1
9.09E-114	1.380638155	0.813	0.374	3.05E-109	10	IFITM3
9.49E-113	1.116554203	0.994	0.839	3.18E-108	10	B2M
1.09E-112	1.306387589	0.636	0.195	3.66E-108	10	DDX17
2.79E-112	0.946868929	0.361	0.061	9.35E-108	10	BTN3A2
3.83E-112	0.904630246	0.289	0.04	1.28E-107	10	LTBP3
5.74E-112	0.883526509	0.292	0.041	1.93E-107	10	LUZP1
1.62E-111	0.839496159	0.262	0.033	5.43E-107	10	CIITA
1.73E-111	0.834571901	0.256	0.032	5.79E-107	10	SNRK
2.79E-111	0.840736099	0.271	0.035	9.37E-107	10	PEA15
3.11E-111	1.010533168	0.623	0.17	1.04E-106	10	HLA-DPB1
8.07E-111	1.187465357	0.464	0.099	2.70E-106	10	HIPK2
5.04E-110	0.885606845	0.386	0.069	1.69E-105	10	RBMS1
1.34E-107	1.105554386	0.919	0.392	4.50E-103	10	MALAT1
6.14E-106	1.313709149	0.599	0.175	2.06E-101	10	KTN1
1.68E-105	1.024080647	0.425	0.087	5.62E-101	10	TINAGL1
4.63E-105	1.146829258	0.428	0.089	1.55E-100	10	NKTR
6.11E-105	1.084001391	0.446	0.095	2.05E-100	10	DYNC1H1
8.54E-105	1.445877642	0.491	0.121	2.86E-100	10	SGK1
1.66E-103	0.735223856	0.289	0.042	5.56E-99	10	PTK2
2.05E-103	0.883650136	0.358	0.063	6.89E-99	10	ODF2L
1.99E-102	0.897499215	0.322	0.052	6.66E-98	10	HLA-DMB
4.95E-102	1.213280658	0.774	0.334	1.66E-97	10	HLA-DRA
8.30E-101	0.940388481	0.316	0.052	2.78E-96	10	CCNL2
1.93E-100	0.796741915	0.316	0.051	6.46E-96	10	TAP1
8.03E-100	1.083174764	0.41	0.085	2.69E-95	10	RRBP1
1.67E-99	0.735104954	0.343	0.059	5.58E-95	10	TIMP1
6.87E-98	0.94315963	0.304	0.049	2.30E-93	10	PLCG2
6.13E-97	0.779515309	0.307	0.05	2.05E-92	10	SCARB2
1.14E-96	1.125376173	0.5	0.13	3.82E-92	10	VAMP5
6.46E-96	0.877815312	0.38	0.075	2.17E-91	10	ISG15
1.13E-95	1.046193347	0.386	0.078	3.80E-91	10	PCM1

4.68E-95	0.95658208	0.383	0.078	1.57E-90	10	TPM4
1.68E-94	1.037552064	0.364	0.072	5.64E-90	10	MAP4
5.69E-94	1.222528487	0.515	0.142	1.91E-89	10	ARGLU1
2.97E-93	0.839137782	0.313	0.054	9.96E-89	10	FTX
4.82E-93	0.720964507	0.253	0.036	1.62E-88	10	MARCKSL1
7.70E-92	0.689400886	0.253	0.036	2.58E-87	10	RNF145
1.05E-91	0.92411937	0.398	0.084	3.52E-87	10	IFI16
1.77E-90	0.730277871	0.274	0.043	5.94E-86	10	MAP3K11
7.14E-90	1.204815871	0.738	0.291	2.40E-85	10	TXNIP
1.25E-89	0.813337499	0.334	0.062	4.18E-85	10	CAPN2
4.81E-89	1.10828228	0.675	0.252	1.61E-84	10	GNAS
8.99E-89	0.725018125	0.286	0.047	3.02E-84	10	ITM2A
9.80E-89	0.860240659	0.259	0.04	3.29E-84	10	SLC6A6
1.40E-88	0.803666102	0.271	0.043	4.68E-84	10	SWAP70
3.59E-88	1.151981634	0.545	0.166	1.20E-83	10	CFLAR
7.74E-88	1.02647619	0.319	0.059	2.60E-83	10	DHRS3
4.37E-87	1.0399588	0.455	0.115	1.47E-82	10	ATRX
1.95E-85	0.998239925	0.358	0.075	6.53E-81	10	SKIL
3.52E-84	0.617839689	0.919	0.454	1.18E-79	10	TMSB4X
1.60E-83	0.916616472	0.349	0.071	5.35E-79	10	NRP1
6.03E-83	0.886335662	0.334	0.067	2.02E-78	10	CCPG1
8.35E-82	1.133965703	0.524	0.155	2.80E-77	10	RNF213
3.99E-81	1.03537699	0.434	0.11	1.34E-76	10	STOM
1.02E-79	1.290771648	0.75	0.389	3.42E-75	10	IGFBP7
1.70E-79	0.969733372	0.485	0.134	5.70E-75	10	PSMB9
9.64E-79	0.754096566	0.28	0.049	3.23E-74	10	AFDN
3.97E-78	0.803567879	0.256	0.043	1.33E-73	10	SLC44A1
9.19E-78	0.741893598	0.289	0.054	3.08E-73	10	RBM5
2.06E-77	0.863598207	0.337	0.07	6.92E-73	10	CTNND1
2.13E-77	0.888025524	0.485	0.136	7.15E-73	10	IFITM2
2.16E-77	0.772415447	0.28	0.051	7.24E-73	10	SNTB2
1.81E-76	0.862004512	0.355	0.079	6.07E-72	10	SERINC1
1.93E-76	0.897171081	0.41	0.099	6.47E-72	10	LGALS1
6.21E-76	1.277015241	0.633	0.238	2.08E-71	10	VIM
8.01E-76	0.847369197	0.328	0.069	2.68E-71	10	VPS13C
1.63E-75	1.145944448	0.575	0.196	5.48E-71	10	SRRM2
3.13E-75	0.826580391	0.295	0.058	1.05E-70	10	MXD4
5.87E-75	0.63949688	0.319	0.064	1.97E-70	10	SERPING1
6.66E-75	0.792283375	0.292	0.056	2.24E-70	10	NIPBL
1.41E-74	0.837042762	0.322	0.067	4.73E-70	10	SETX

3.44E-74	0.70796254	0.256	0.044	1.15E-69	10	LPAR6
3.51E-74	0.697980775	0.271	0.048	1.18E-69	10	CTBP2
5.72E-74	1.009378405	0.407	0.106	1.92E-69	10	WSB1
9.47E-74	0.86124624	0.37	0.089	3.18E-69	10	CCDC85B
1.82E-72	0.920133035	0.413	0.108	6.12E-68	10	WASF2
3.02E-72	0.779137452	0.304	0.062	1.01E-67	10	ITGA6
4.17E-72	0.81030383	0.346	0.078	1.40E-67	10	DYNC1LI2
4.68E-72	0.627861234	0.265	0.048	1.57E-67	10	HOOK3
5.48E-72	0.933137794	0.422	0.114	1.84E-67	10	HLA-DMA
5.98E-72	0.706041158	0.289	0.056	2.00E-67	10	MGAT1
1.00E-70	0.793460348	0.292	0.059	3.36E-66	10	SMARCA2
1.60E-69	0.723973804	0.268	0.05	5.36E-65	10	CDC42BPA
1.87E-69	0.886619087	0.416	0.11	6.28E-65	10	NFIA
3.86E-69	1.009127539	0.572	0.213	1.29E-64	10	SERPINB6
8.43E-69	0.646246744	0.265	0.05	2.83E-64	10	BNIP2
1.06E-68	0.996298073	0.524	0.175	3.57E-64	10	MSN
8.90E-68	0.65993433	0.259	0.048	2.99E-63	10	MAN1A1
2.93E-67	0.818942384	0.355	0.086	9.81E-63	10	TMOD3
1.43E-66	0.890169533	0.295	0.063	4.79E-62	10	KMT2A
5.16E-66	0.897445853	0.325	0.075	1.73E-61	10	GOLGB1
9.70E-66	0.735991286	0.28	0.057	3.25E-61	10	FLNA
3.27E-65	0.920936929	0.355	0.088	1.10E-60	10	TPR
5.58E-65	0.702890966	0.286	0.06	1.87E-60	10	TRIP11
2.05E-64	0.695282709	0.259	0.051	6.88E-60	10	PKN1
3.57E-61	0.848168534	0.373	0.101	1.20E-56	10	TAPBP
3.97E-61	0.704517246	0.304	0.069	1.33E-56	10	BOD1L1
2.07E-60	0.885032712	0.361	0.097	6.95E-56	10	ADAR
2.24E-60	0.892996615	0.455	0.144	7.50E-56	10	LUC7L3
9.32E-60	0.869713106	0.325	0.081	3.13E-55	10	BPTF
1.75E-59	0.963308766	0.756	0.414	5.86E-55	10	S100A10
1.38E-58	0.802425318	0.389	0.111	4.61E-54	10	LPP
1.50E-58	0.744806653	0.386	0.107	5.03E-54	10	SPAG9
1.88E-57	0.834574288	0.428	0.133	6.29E-53	10	CD99
2.02E-57	0.764190221	0.271	0.059	6.79E-53	10	NFE2L1
3.45E-57	0.82060823	0.395	0.115	1.16E-52	10	SP100
5.33E-57	0.727236284	0.322	0.08	1.79E-52	10	IQGAP1
5.42E-57	0.711517806	0.283	0.064	1.82E-52	10	CLK1
9.63E-57	0.872126017	0.473	0.161	3.23E-52	10	GTF2I
5.38E-56	0.808113708	0.292	0.069	1.80E-51	10	EIF2AK2
1.85E-55	0.884812365	0.413	0.128	6.19E-51	10	PRRC2C

2.13E-55	0.726519306	0.307	0.075	7.15E-51	10	CALCOCO2
1.15E-54	0.795567417	0.38	0.11	3.84E-50	10	GNB1
5.41E-54	0.563802873	0.253	0.055	1.81E-49	10	KLF9
6.74E-54	0.922169717	0.467	0.166	2.26E-49	10	NFKBIA
1.53E-53	0.758397148	0.367	0.106	5.14E-49	10	RSRP1
2.23E-53	0.992095319	0.681	0.384	7.48E-49	10	DDX5
2.42E-53	0.884145404	0.377	0.112	8.12E-49	10	ZNF207
6.09E-53	0.688145349	0.286	0.069	2.04E-48	10	VAT1
1.33E-52	0.85256041	0.443	0.15	4.46E-48	10	ACTN4
1.99E-52	0.890723016	0.536	0.211	6.68E-48	10	RDX
5.58E-52	0.868801734	0.491	0.183	1.87E-47	10	PDIA3
1.63E-51	0.818521167	0.355	0.103	5.47E-47	10	NORAD
5.29E-51	0.690698199	0.358	0.103	1.77E-46	10	NDRG1
5.31E-51	0.650312576	0.31	0.081	1.78E-46	10	ARID1B
1.28E-50	0.780701702	0.262	0.062	4.30E-46	10	ZBTB7A
1.04E-49	0.782363305	0.328	0.092	3.47E-45	10	TM9SF3
6.63E-49	0.713809376	0.292	0.076	2.22E-44	10	ROCK1
1.09E-48	0.662489069	0.355	0.102	3.66E-44	10	GMFG
2.89E-48	0.804149	0.295	0.078	9.68E-44	10	H1FX
2.96E-48	0.912001898	0.377	0.122	9.93E-44	10	NCOA7
4.44E-48	0.631875671	0.259	0.063	1.49E-43	10	APOL6
5.04E-48	0.75632764	0.316	0.088	1.69E-43	10	ADD1
6.28E-48	0.794695097	0.322	0.092	2.11E-43	10	IRF1
1.63E-47	0.869778578	0.383	0.123	5.47E-43	10	LRRFIP1
4.03E-47	0.700313037	0.259	0.063	1.35E-42	10	SFT2D2
8.36E-47	0.622153057	0.34	0.1	2.80E-42	10	PLSCR1
8.65E-47	0.825850098	0.343	0.104	2.90E-42	10	POLR2J3.1
8.69E-47	0.615726327	0.259	0.063	2.91E-42	10	CRK
9.32E-47	0.761483552	0.355	0.109	3.13E-42	10	KIF5B
2.72E-46	0.546684042	0.262	0.064	9.12E-42	10	ABHD17A
4.33E-46	0.818702849	0.262	0.066	1.45E-41	10	EFCAB14
6.10E-46	0.804370933	0.425	0.15	2.05E-41	10	HNRNPH1
8.53E-46	0.635377127	0.259	0.064	2.86E-41	10	GYPC
1.08E-45	0.771437357	0.298	0.083	3.61E-41	10	JMJD1C
1.47E-45	0.756662785	0.428	0.151	4.93E-41	10	GRN
4.72E-45	0.676260016	0.253	0.062	1.58E-40	10	USP34
5.46E-45	0.632555109	0.292	0.081	1.83E-40	10	SLC38A2
1.76E-44	0.593873853	0.265	0.067	5.90E-40	10	PHACTR4
3.09E-44	0.877096018	0.53	0.23	1.04E-39	10	ZFP36L1
5.20E-44	0.755182979	0.262	0.068	1.75E-39	10	ZNF638

1.48E-43	0.771650201	0.497	0.201	4.95E-39	10	N4BP2L2
5.27E-43	0.528500934	0.265	0.069	1.77E-38	10	FOXP1
8.13E-43	0.71059306	0.328	0.102	2.73E-38	10	CD46
3.10E-42	0.771407325	0.53	0.225	1.04E-37	10	HNRNPU
3.63E-42	0.514006658	0.934	0.735	1.22E-37	10	TMSB10
1.28E-41	0.750778505	0.337	0.107	4.30E-37	10	CTNNB1
1.37E-41	0.546317445	0.271	0.073	4.60E-37	10	WNK1
1.93E-41	0.808748862	0.515	0.226	6.49E-37	10	ZBTB20
2.38E-41	0.621307678	0.256	0.067	7.99E-37	10	FRMD4B
2.63E-41	0.71902933	0.298	0.088	8.82E-37	10	PURA
2.74E-41	0.727419556	0.431	0.161	9.19E-37	10	SH3GLB1
3.60E-41	0.720837443	0.413	0.149	1.21E-36	10	JAK1
4.15E-41	0.593878262	0.31	0.093	1.39E-36	10	STAT1
4.16E-41	0.816386469	0.599	0.316	1.39E-36	10	FKBP1A
7.45E-41	0.559364656	0.268	0.074	2.50E-36	10	NUB1
7.68E-41	0.650007158	0.283	0.08	2.58E-36	10	NFIC
9.77E-41	0.743538922	0.425	0.158	3.28E-36	10	SCAF11
8.86E-40	0.707319942	0.322	0.102	2.97E-35	10	PNN
2.09E-39	0.675556694	0.331	0.108	7.01E-35	10	CCDC50
2.54E-39	0.819405728	0.298	0.093	8.51E-35	10	ANKRD11
5.31E-39	0.680648236	0.262	0.074	1.78E-34	10	DDIT4
5.46E-39	0.519504475	0.268	0.075	1.83E-34	10	ATP1B3
5.55E-39	0.599587091	0.343	0.112	1.86E-34	10	TNFRSF1A
6.04E-39	0.751102136	0.437	0.169	2.02E-34	10	PNISR
1.31E-38	0.632339653	0.313	0.1	4.40E-34	10	BCLAF1
1.97E-38	0.684978158	0.268	0.078	6.62E-34	10	SEPT9
3.95E-38	0.59831633	0.253	0.069	1.32E-33	10	SREK1
7.33E-38	0.614022711	0.34	0.114	2.46E-33	10	CHD9
1.46E-37	0.669658552	0.28	0.084	4.89E-33	10	RSF1
1.59E-37	0.75507199	0.434	0.172	5.34E-33	10	NFIB
5.95E-37	0.827040813	0.512	0.239	2.00E-32	10	HNRNPA3
6.09E-37	0.623323635	0.262	0.075	2.04E-32	10	NCKAP1
1.49E-36	0.525515668	0.301	0.094	4.99E-32	10	STAT3
1.59E-36	0.547070753	0.259	0.074	5.32E-32	10	MYCBP2
2.03E-36	0.708807296	0.422	0.165	6.80E-32	10	FUS
1.54E-35	0.637939276	0.259	0.075	5.15E-31	10	GLUL
2.00E-35	0.56626531	0.259	0.076	6.72E-31	10	IRF2BP2
4.35E-35	0.697967308	0.419	0.166	1.46E-30	10	AES
5.10E-35	0.857810175	0.566	0.295	1.71E-30	10	SON
1.90E-34	0.637194784	0.352	0.128	6.36E-30	10	TNRC6B

2.45E-34	0.711923277	0.337	0.119	8.22E-30	10	AKAP9
4.13E-34	0.695275179	0.289	0.094	1.38E-29	10	OGA
4.28E-34	0.601753162	0.259	0.077	1.43E-29	10	KIAA1551
5.63E-34	0.722910897	0.361	0.135	1.89E-29	10	NFE2L2
1.24E-33	0.426341471	0.319	0.107	4.15E-29	10	HLA-DRB5
2.15E-33	0.634997172	0.331	0.117	7.22E-29	10	TLN1
3.78E-33	0.709188098	0.41	0.164	1.27E-28	10	HP1BP3
8.60E-33	0.655893696	0.497	0.233	2.88E-28	10	SDCBP
8.99E-33	0.678907626	0.373	0.145	3.01E-28	10	SF3B1
1.66E-32	0.615886298	0.268	0.086	5.56E-28	10	PTBP1
1.95E-32	0.652207789	0.458	0.202	6.56E-28	10	LEPROT
2.65E-32	0.757620258	0.355	0.137	8.89E-28	10	TGOLN2
3.52E-32	0.699308689	0.389	0.156	1.18E-27	10	SFPQ
9.08E-32	0.6884002	0.386	0.155	3.05E-27	10	YWHAH
2.04E-31	0.661381835	0.38	0.152	6.85E-27	10	PSMB8
2.11E-31	0.617207711	0.307	0.108	7.07E-27	10	KMT2E
2.38E-31	0.66234773	0.283	0.096	7.97E-27	10	ZRANB2
2.46E-31	0.537516778	0.364	0.138	8.26E-27	10	CD151
3.02E-31	0.743430446	0.355	0.137	1.01E-26	10	LMNA
5.68E-31	0.690105257	0.355	0.139	1.91E-26	10	SNRNP70
6.83E-31	0.505777446	0.253	0.079	2.29E-26	10	PPP1R12A
1.27E-30	0.331634286	0.313	0.105	4.26E-26	10	SRGN
1.30E-30	0.743941574	0.449	0.202	4.36E-26	10	CAST
1.76E-30	0.651829334	0.726	0.477	5.90E-26	10	ACTG1
1.99E-30	0.532363654	0.316	0.112	6.67E-26	10	MYO6
2.14E-30	0.563641546	0.301	0.106	7.17E-26	10	PIM3
5.16E-30	0.756160333	0.259	0.086	1.73E-25	10	ASH1L
8.92E-30	0.517947724	0.277	0.091	2.99E-25	10	COL18A1
3.43E-29	0.591374216	0.301	0.106	1.15E-24	10	EIF4H
3.68E-29	0.672207257	0.714	0.486	1.23E-24	10	HNRNPA2B1
7.44E-29	0.559505205	0.373	0.153	2.49E-24	10	CSNK1A1
1.03E-28	0.526666522	0.536	0.258	3.47E-24	10	MTRNR2L12
1.31E-28	0.64119947	0.319	0.12	4.39E-24	10	ANKRD12
4.38E-28	0.666156724	0.295	0.109	1.47E-23	10	RBM25
4.44E-28	0.567426685	0.346	0.137	1.49E-23	10	CDC37
4.57E-28	0.68625329	0.431	0.198	1.53E-23	10	LAMP1
1.22E-27	0.522047933	0.316	0.119	4.09E-23	10	GOLGA4
1.80E-27	0.627229398	0.286	0.104	6.05E-23	10	NR3C1
3.33E-27	0.696467751	0.295	0.108	1.12E-22	10	HES1
3.74E-27	0.564734209	0.301	0.112	1.25E-22	10	PICALM

5.53E-27	0.536855774	0.271	0.094	1.85E-22	10	ARHGEF12
1.32E-26	0.667026682	0.476	0.24	4.43E-22	10	RBM39
1.42E-26	0.577041826	0.38	0.168	4.75E-22	10	TMEM50A
2.57E-26	0.492678704	0.265	0.094	8.61E-22	10	PABPN1
2.64E-26	0.613429866	0.253	0.088	8.84E-22	10	RNF19A
2.90E-26	0.593176399	0.392	0.176	9.71E-22	10	SRSF11
4.41E-26	0.524720124	0.289	0.106	1.48E-21	10	RAD21
6.11E-26	0.5230291	0.467	0.223	2.05E-21	10	SEPT7
6.64E-26	0.601110617	0.774	0.602	2.23E-21	10	HMGB1
1.32E-25	0.467764114	0.313	0.121	4.44E-21	10	EWSR1
2.47E-25	0.475315499	0.271	0.099	8.28E-21	10	KPNB1
8.92E-25	0.51933217	0.295	0.113	2.99E-20	10	MBNL1
1.32E-24	0.636970085	0.401	0.185	4.43E-20	10	ZFP36L2
2.43E-24	0.629999628	0.479	0.251	8.16E-20	10	TPM3
3.40E-24	0.50567024	0.395	0.178	1.14E-19	10	SEPT2
7.93E-24	0.617576013	0.599	0.359	2.66E-19	10	TNFSF10
1.09E-23	0.613266226	0.355	0.158	3.66E-19	10	MTDH
2.01E-23	0.554252462	0.256	0.095	6.74E-19	10	CASP4
2.08E-23	0.534335458	0.265	0.098	6.99E-19	10	PJA2
3.13E-23	0.663341709	0.512	0.282	1.05E-18	10	HSP90B1
3.75E-23	0.541363667	0.268	0.101	1.26E-18	10	FGD5-AS1
4.30E-23	0.55181657	0.298	0.123	1.44E-18	10	EIF3A
4.60E-23	0.58682579	0.271	0.104	1.54E-18	10	BRD2
5.30E-23	0.519417567	0.669	0.414	1.78E-18	10	MT-ND4L
7.21E-23	0.351690276	0.964	0.823	2.42E-18	10	MT-ND3
8.20E-23	0.579365914	0.316	0.133	2.75E-18	10	HNRNPR
9.45E-23	0.566409357	0.292	0.118	3.17E-18	10	RPN2
1.94E-22	0.522168176	0.343	0.148	6.51E-18	10	GSDMD
2.71E-22	0.558845466	0.265	0.102	9.09E-18	10	WAC
3.15E-22	0.530345788	0.355	0.155	1.06E-17	10	CTNNA1
4.34E-22	0.504377392	0.28	0.111	1.45E-17	10	NCOR1
1.02E-21	0.458212953	0.31	0.127	3.40E-17	10	ARHGDIB
1.29E-21	0.420698953	0.277	0.11	4.33E-17	10	SPCS3
1.52E-21	0.519569819	0.28	0.113	5.11E-17	10	PINK1
2.66E-21	0.573152452	0.271	0.109	8.92E-17	10	RBM17
3.69E-21	0.655971552	0.593	0.384	1.24E-16	10	SEC62
5.11E-21	0.505834725	0.274	0.11	1.71E-16	10	EMP3
6.29E-21	0.561465424	0.446	0.238	2.11E-16	10	LY6E
1.49E-20	0.62335317	0.431	0.226	4.99E-16	10	DNAJA1
1.50E-20	0.517458383	0.265	0.106	5.02E-16	10	U2SURP

1.50E-20	0.491768114	0.256	0.101	5.04E-16	10	PSIP1
2.46E-20	0.610779649	0.307	0.132	8.26E-16	10	MPHOSPH8
6.77E-20	0.406746229	0.343	0.151	2.27E-15	10	COX7A1
6.91E-20	0.604157955	0.425	0.222	2.32E-15	10	CALR
7.51E-20	0.566623687	0.307	0.134	2.52E-15	10	SRSF10
9.05E-20	0.363106056	0.265	0.106	3.03E-15	10	TMEM123
1.10E-19	0.532181625	0.443	0.238	3.67E-15	10	TAGLN2
3.00E-19	0.470783229	0.262	0.106	1.01E-14	10	TUBA1A
3.55E-19	0.506028969	0.319	0.145	1.19E-14	10	HNRNPD
1.09E-18	0.447663241	0.349	0.167	3.65E-14	10	CAPNS1
3.22E-18	0.562754979	0.256	0.107	1.08E-13	10	FAM120A
4.58E-18	0.550187547	0.587	0.382	1.54E-13	10	YWHAB
5.17E-18	0.427770921	0.259	0.107	1.73E-13	10	SRSF4
5.73E-18	0.536780703	0.44	0.238	1.92E-13	10	RAP1B
1.04E-17	0.446307784	0.289	0.131	3.49E-13	10	PRPF4B
1.27E-17	0.518646066	0.334	0.161	4.24E-13	10	CLTC
1.34E-17	0.35838796	0.867	0.794	4.48E-13	10	ITM2B
1.83E-17	0.501141742	0.292	0.133	6.14E-13	10	MCL1
2.41E-17	0.560367742	0.286	0.13	8.10E-13	10	GCC2
3.38E-17	0.454322827	0.346	0.17	1.13E-12	10	OPTN
6.02E-17	0.293433391	0.322	0.146	2.02E-12	10	IER2
6.73E-17	0.556594932	0.566	0.367	2.26E-12	10	RHOA
7.00E-17	0.352697565	0.262	0.111	2.35E-12	10	TCN2
1.73E-16	0.496990334	0.262	0.115	5.79E-12	10	C6orf62
2.11E-16	0.476119798	0.271	0.123	7.09E-12	10	PRMT1
3.39E-16	0.466860676	0.277	0.127	1.14E-11	10	HNRNPH3
3.69E-16	0.39543103	0.268	0.119	1.24E-11	10	CDV3
8.11E-16	0.680172422	0.464	0.277	2.72E-11	10	TUBA1B
9.58E-16	0.267898817	0.946	0.801	3.21E-11	10	MT-ND1
1.11E-15	0.433445238	0.271	0.125	3.74E-11	10	ATF4
1.21E-15	0.500247033	0.419	0.235	4.05E-11	10	ANXA5
1.28E-15	0.43381182	0.334	0.169	4.30E-11	10	ARL6IP5
1.40E-15	0.306343713	0.343	0.167	4.71E-11	10	JUND
2.48E-15	0.434677365	0.307	0.15	8.31E-11	10	VCP
4.40E-15	0.444639147	0.331	0.169	1.47E-10	10	ACTR2
4.78E-15	0.313479562	0.334	0.164	1.60E-10	10	SELENOM
6.00E-15	0.354310924	0.259	0.117	2.01E-10	10	HPCAL1
6.12E-15	0.317626109	0.253	0.114	2.05E-10	10	G3BP1
6.14E-15	0.397313985	0.262	0.12	2.06E-10	10	TRA2B
7.25E-15	0.379314887	0.265	0.122	2.43E-10	10	SYNCRIP

1.22E-14	0.388497706	0.31	0.152	4.08E-10	10	NONO
1.80E-14	0.329675735	0.292	0.139	6.03E-10	10	SLTM
2.16E-14	0.494818304	0.458	0.274	7.25E-10	10	CSDE1
2.52E-14	0.439471966	0.256	0.119	8.44E-10	10	TRIM38
2.68E-14	0.34172169	0.304	0.149	8.98E-10	10	TYMP
1.45E-13	0.32851855	0.28	0.134	4.86E-09	10	CTSL
1.69E-13	0.498745758	0.268	0.131	5.67E-09	10	SF1
2.90E-13	0.405818096	0.419	0.241	9.74E-09	10	APLP2
3.38E-13	0.405981827	0.295	0.15	1.13E-08	10	GNB2
3.62E-13	0.446575358	0.313	0.164	1.22E-08	10	HNRNPM
4.17E-13	0.4024372	0.328	0.174	1.40E-08	10	RBMX
4.27E-13	0.397826873	0.367	0.204	1.43E-08	10	UBXN4
1.38E-12	0.447934442	0.389	0.226	4.64E-08	10	TCF25
1.57E-12	0.307240746	0.982	0.855	5.26E-08	10	MT-ATP6
1.67E-12	0.406599595	0.313	0.166	5.61E-08	10	FDPS
2.08E-12	0.415487751	0.274	0.138	6.97E-08	10	RAB14
3.88E-12	0.40742974	0.304	0.159	1.30E-07	10	HSPA5
5.34E-12	0.320482397	0.446	0.267	1.79E-07	10	CALD1
6.57E-12	0.321723533	0.289	0.149	2.20E-07	10	NUCB1
1.11E-11	0.282410884	0.265	0.133	3.72E-07	10	TSC22D3
1.20E-11	0.448925611	0.253	0.129	4.02E-07	10	THRAP3
3.12E-11	0.349428015	0.533	0.35	1.04E-06	10	CNN3
5.02E-11	0.471832839	0.28	0.148	1.68E-06	10	YY1
5.72E-11	0.401372096	0.265	0.138	1.92E-06	10	GPBP1
6.40E-11	0.363121978	0.286	0.153	2.15E-06	10	ATP6AP2
1.45E-10	0.34187577	0.482	0.316	4.88E-06	10	PSAP
1.50E-10	0.370162686	0.37	0.219	5.05E-06	10	CANX
1.60E-10	0.443441884	0.319	0.184	5.37E-06	10	TRAM1
1.70E-10	0.429266876	0.476	0.313	5.71E-06	10	NCL
1.79E-10	0.356999309	0.322	0.181	6.01E-06	10	PDIA6
2.27E-10	0.412127307	0.666	0.526	7.61E-06	10	HSP90AB1
3.53E-10	0.430740519	0.274	0.148	1.18E-05	10	VCAM1
3.61E-10	0.260646558	0.271	0.142	1.21E-05	10	GINM1
3.80E-10	0.436206327	0.497	0.341	1.27E-05	10	EIF4G2
6.70E-10	0.371849083	0.259	0.138	2.25E-05	10	HDLBP
1.34E-09	0.443931104	0.392	0.249	4.50E-05	10	DYNC1I2
1.81E-09	0.403239696	0.654	0.564	6.08E-05	10	MYL12A
3.37E-09	0.500674683	0.395	0.251	0.00011292	10	LAP3
4.32E-09	0.380955438	0.298	0.175	0.000144778	10	ANP32B
7.86E-09	0.298062156	0.304	0.176	0.000263774	10	PPIG

1.46E-08	0.281834248	0.265	0.148	0.000488951	10	MAF
1.94E-08	0.302325739	0.265	0.153	0.000652212	10	DDX3X
2.08E-08	0.28806012	0.256	0.143	0.00069637	10	C5orf24
2.95E-08	0.298521441	0.292	0.168	0.000989726	10	SNX6
3.65E-08	0.265567643	0.328	0.197	0.001224298	10	ARHGDIA
3.78E-08	0.348278314	0.256	0.147	0.001266794	10	PRMT2
4.16E-08	0.353163423	0.416	0.274	0.001394139	10	CLIC4
7.49E-08	0.318944301	0.611	0.512	0.002512734	10	CD63
1.05E-07	0.34166444	0.298	0.182	0.003514752	10	DEK
1.11E-07	0.351182819	0.425	0.298	0.003710411	10	SRSF5
1.38E-07	0.296133663	0.37	0.237	0.004635799	10	YBX3
1.43E-07	0.272153093	0.322	0.195	0.004801053	10	MVP
1.93E-07	0.373617078	0.298	0.188	0.006465498	10	PAPOLA
3.63E-07	0.360049039	0.31	0.197	0.012182598	10	XRCC5
5.64E-07	0.343376807	0.256	0.154	0.018921743	10	PNRC2
6.45E-07	0.293151324	0.268	0.163	0.021636832	10	UFM1
1.06E-06	0.251021322	0.289	0.181	0.035557296	10	RAP1A
1.37E-06	0.314070385	0.349	0.23	0.04580006	10	YWHAZ
1.51E-06	0.37412052	0.398	0.274	0.050709215	10	DDX24
1.68E-06	0.317334604	0.485	0.354	0.056199691	10	NUCKS1
2.07E-06	0.264949083	0.34	0.222	0.069532163	10	ARPC5
2.11E-06	0.310792454	0.533	0.415	0.070785988	10	HNRNPK
2.75E-06	0.354254847	0.262	0.168	0.09228754	10	KHDRBS1
3.35E-06	0.291890767	0.307	0.201	0.112237025	10	RTF1
3.74E-06	0.293505142	0.645	0.542	0.12545664	10	RAC1
3.79E-06	0.258716828	0.711	0.648	0.12697027	10	UBC
4.09E-06	0.326068681	0.38	0.266	0.137100934	10	RAB11A
4.77E-06	0.275214762	0.304	0.2	0.160112854	10	BZW1
8.36E-06	0.343526792	0.455	0.337	0.280286414	10	HNRNPDL
1.30E-05	0.28032597	0.325	0.218	0.437068723	10	CBX3
1.48E-05	0.292124159	0.37	0.262	0.494908449	10	TUBB
1.66E-05	0.322218354	0.5	0.389	0.555951518	10	PTMS
2.69E-05	0.303229652	0.446	0.338	0.901070915	10	SERBP1
0.000103995	0.294415013	0.389	0.286	1	10	AP2M1
0.000111069	0.280635967	0.31	0.219	1	10	ARF1
0.000563789	0.269896589	0.253	0.182	1	10	H2AFV
0.000844691	0.25445445	0.298	0.215	1	10	OCIAD1
0.001090152	0.253892918	0.355	0.27	1	10	C12orf57
0.003466311	0.296137445	0.301	0.232	1	10	EIF5B
0	3.583028557	0.301	0.002	0	11	C7

0	3.44247999	0.509	0.004	0	11	RGS5
0	3.33726802	0.673	0.009	0	11	TAGLN
0	3.283965861	0.748	0.016	0	11	MGP
0	2.916177061	0.606	0.015	0	11	ACTA2
0	2.822601347	0.319	0.004	0	11	DCN
0	2.726003182	0.416	0.003	0	11	MYH11
0	2.673636494	0.752	0.034	0	11	TIMP3
0	2.658606846	0.686	0.014	0	11	A2M
0	2.642195845	0.46	0.003	0	11	COL3A1
0	2.55131971	0.562	0.025	0	11	SPARCL1
0	2.494036006	0.513	0.002	0	11	COL1A2
0	2.478219989	0.606	0.008	0	11	TPM2
0	2.406415675	0.646	0.048	0	11	MYL9
0	2.321729597	0.681	0.056	0	11	SPARC
0	2.227636449	0.646	0.003	0	11	BGN
0	2.182085713	0.571	0.003	0	11	C11orf96
0	2.109298869	0.527	0.022	0	11	IGFBP5
0	2.102909184	0.588	0.006	0	11	COL6A2
0	2.071634632	0.575	0.002	0	11	PDGFRB
0	2.070657096	0.575	0.001	0	11	CARMN
0	2.041119501	0.447	0.004	0	11	MCAM
0	1.984969807	0.575	0.003	0	11	MFGE8
0	1.974611506	0.447	0.002	0	11	C2orf40
0	1.969719728	0.487	0.005	0	11	AEBP1
0	1.944344644	0.345	0.001	0	11	RERGL
0	1.891151998	0.416	0.002	0	11	COL1A1
0	1.8859003	0.527	0.001	0	11	FRZB
0	1.860611936	0.597	0.012	0	11	CAVIN1
0	1.841050084	0.615	0.047	0	11	EPAS1
0	1.841026093	0.261	0.001	0	11	TNC
0	1.832938247	0.54	0.033	0	11	PALLD
0	1.830802959	0.553	0.003	0	11	SLIT3
0	1.828993059	0.544	0.028	0	11	MYLK
0	1.80098251	0.531	0.009	0	11	PTN
0	1.77613429	0.372	0.012	0	11	C1R
0	1.763163903	0.5	0.001	0	11	PLAC9
0	1.752908347	0.473	0.027	0	11	ITGA1
0	1.718505674	0.425	0.011	0	11	FSTL1
0	1.684203041	0.319	0.005	0	11	CTGF
0	1.648205241	0.429	0.001	0	11	NOTCH3

0	1.616623037	0.367	0.016	0	11	CPE
0	1.60108623	0.473	0.008	0	11	JAG1
0	1.591613003	0.381	0.009	0	11	FN1
0	1.577252635	0.372	0.01	0	11	C1S
0	1.55362105	0.31	0.011	0	11	RRAD
0	1.544044368	0.31	0	0	11	PLN
0	1.527674631	0.305	0.002	0	11	EMILIN1
0	1.463670723	0.434	0.02	0	11	TIMP2
0	1.460001734	0.482	0.027	0	11	DST
0	1.457964254	0.487	0.009	0	11	KANK2
0	1.451116943	0.341	0.006	0	11	CAV1
0	1.41329247	0.385	0.01	0	11	PTP4A3
0	1.404044787	0.46	0.019	0	11	COL6A1
0	1.364333246	0.429	0.004	0	11	MAP1B
0	1.359724543	0.385	0.001	0	11	SYNPO2
0	1.350769717	0.42	0.006	0	11	PPP1R14A
0	1.309413618	0.363	0.016	0	11	ENG
0	1.280402159	0.305	0.009	0	11	TM4SF1
0	1.271825847	0.394	0.01	0	11	TNS2
0	1.241743607	0.372	0.007	0	11	ARHGEF17
0	1.228847348	0.345	0.001	0	11	ITGA7
0	1.198478784	0.323	0.004	0	11	SLC7A2
0	1.187271638	0.261	0	0	11	NTRK3
0	1.168376169	0.305	0.002	0	11	SEPT4
0	1.145741813	0.274	0.001	0	11	NDUFA4L2
0	1.134867458	0.257	0.004	0	11	FBN1
0	1.127642237	0.27	0.002	0	11	TCF21
0	1.11568185	0.323	0.004	0	11	NFASC
0	1.096654865	0.354	0.007	0	11	LHFPL6
0	1.08771182	0.323	0.01	0	11	NTRK2
0	1.082830066	0.354	0.007	0	11	FXYD1
0	1.066804471	0.341	0.009	0	11	MAP3K20
0	1.066668194	0.274	0.003	0	11	TBX3
0	1.052349114	0.305	0.006	0	11	WTIP
0	1.046390073	0.257	0.006	0	11	NES
0	1.039715723	0.301	0.002	0	11	MRVI1
0	1.037997592	0.292	0.004	0	11	NEXN
0	1.024490607	0.265	0	0	11	PRELP
0	1.013361428	0.314	0.007	0	11	RCAN2
0	1.008391977	0.314	0	0	11	COL14A1

0	0.996963843	0.305	0.006	0	11	ITIH5
0	0.987632644	0.288	0.003	0	11	LTBP1
0	0.985805334	0.274	0.006	0	11	HES4
0	0.984527029	0.27	0.007	0	11	RBMS3
0	0.976158846	0.27	0.001	0	11	EDNRA
0	0.969358823	0.31	0.004	0	11	ADCY3
0	0.962090668	0.31	0.007	0	11	FILIP1L
0	0.959170808	0.27	0.003	0	11	MSRB3
0	0.955822914	0.305	0.001	0	11	ADGRA2
0	0.952865345	0.27	0.002	0	11	EDIL3
0	0.945230678	0.323	0.012	0	11	ADGRF5
0	0.898615974	0.252	0.001	0	11	PGF
0	0.895227227	0.283	0.009	0	11	DLC1
0	0.893071756	0.288	0.004	0	11	NPY1R
0	0.891359815	0.252	0.002	0	11	PDE5A
0	0.881242191	0.265	0.002	0	11	EHD2
0	0.879409926	0.283	0	0	11	DKK3
0	0.878828154	0.274	0.006	0	11	NR2F2-AS1
0	0.86556569	0.252	0.007	0	11	PLEKHH3
5.97E-308	2.055294914	0.628	0.055	2.00E-303	11	FLNA
4.15E-302	1.293271974	0.279	0.01	1.39E-297	11	IGFBP2
3.00E-301	0.976963662	0.296	0.011	1.01E-296	11	CAV2
3.45E-298	1.001489936	0.323	0.014	1.16E-293	11	ISYNA1
1.03E-279	2.228404875	0.712	0.082	3.46E-275	11	NR2F2
6.11E-272	0.841761846	0.283	0.011	2.05E-267	11	EBF1
1.03E-270	1.071286448	0.257	0.009	3.44E-266	11	LRP1
1.75E-250	1.439823681	0.491	0.039	5.86E-246	11	TNS1
7.33E-242	1.064835343	0.319	0.017	2.46E-237	11	GUCY1A1
1.78E-237	0.797929584	0.279	0.013	5.97E-233	11	NT5DC2
3.20E-231	0.785513243	0.252	0.01	1.07E-226	11	MEIS2
1.58E-222	0.998183325	0.336	0.02	5.31E-218	11	ZFHX3
7.48E-221	0.99591462	0.363	0.024	2.51E-216	11	RRAS
2.52E-219	1.909133921	0.726	0.107	8.45E-215	11	CD81
2.15E-211	0.972456584	0.31	0.018	7.21E-207	11	PPP1R12B
5.90E-203	1.105292114	0.319	0.02	1.98E-198	11	EPB41L2
9.36E-199	1.345547802	0.451	0.041	3.14E-194	11	LAMB2
1.46E-195	1.55242258	0.558	0.065	4.89E-191	11	GSN
1.70E-195	2.060157485	0.765	0.133	5.71E-191	11	ITGB1
3.27E-194	0.838756446	0.265	0.014	1.10E-189	11	PMEPA1
1.38E-189	1.56674861	0.58	0.072	4.61E-185	11	AHNAK

2.30E-187	1.105222783	0.363	0.028	7.72E-183	11	ACTN1
1.11E-185	1.172071103	0.319	0.022	3.72E-181	11	UACA
3.58E-179	1.519881275	0.659	0.098	1.20E-174	11	LGALS1
4.85E-175	0.883619745	0.274	0.017	1.63E-170	11	FAM129A
8.40E-169	1.309034931	0.425	0.043	2.82E-164	11	CSRP1
7.83E-167	1.37539543	0.407	0.04	2.63E-162	11	NR2F1
5.16E-163	1.022309742	0.381	0.035	1.73E-158	11	ROCK2
2.63E-161	1.344403192	0.478	0.057	8.82E-157	11	ZEB2
1.33E-159	1.280874047	0.35	0.03	4.47E-155	11	TBX2
8.68E-157	1.486826211	0.27	0.019	2.91E-152	11	PRSS23
4.74E-154	1.080447676	0.35	0.031	1.59E-149	11	PARM1
8.50E-154	1.427344695	0.482	0.061	2.85E-149	11	UTRN
8.71E-152	1.459458145	0.394	0.041	2.92E-147	11	COL4A1
1.50E-151	0.805475139	0.257	0.017	5.02E-147	11	FHL1
5.59E-149	1.573964036	0.478	0.059	1.88E-144	11	TIMP1
1.41E-148	1.226853539	0.412	0.045	4.72E-144	11	COL4A2
9.76E-146	1.460169331	0.429	0.051	3.27E-141	11	ID3
2.15E-144	1.440717629	0.27	0.02	7.20E-140	11	IGFBP6
9.43E-144	1.297560357	0.482	0.063	3.16E-139	11	SERPING1
1.93E-128	2.06870883	0.819	0.265	6.46E-124	11	CALD1
2.61E-128	0.92263026	0.314	0.03	8.75E-124	11	VCL
4.80E-128	2.498690729	0.916	0.389	1.61E-123	11	IGFBP7
5.33E-124	0.886168578	0.288	0.026	1.79E-119	11	EHBP1L1
1.62E-122	1.338389798	0.531	0.091	5.44E-118	11	MYH9
3.60E-122	1.332019373	0.394	0.049	1.21E-117	11	CD9
1.70E-120	0.749659419	0.283	0.026	5.71E-116	11	ENAH
6.17E-119	1.973564952	0.77	0.238	2.07E-114	11	VIM
2.70E-117	1.559112934	0.398	0.053	9.04E-113	11	MEF2C
8.78E-115	1.525862752	0.451	0.071	2.94E-110	11	LBH
4.58E-114	0.943025441	0.332	0.037	1.54E-109	11	CRIP2
5.62E-114	0.909550426	0.279	0.027	1.88E-109	11	EFEMP2
2.96E-111	1.494087469	0.491	0.087	9.93E-107	11	TINAGL1
1.23E-105	1.142528377	0.429	0.066	4.14E-101	11	BST2
1.50E-102	1.310696086	0.478	0.084	5.03E-98	11	SPTBN1
6.32E-102	0.926612334	0.336	0.042	2.12E-97	11	TCF4
1.16E-100	0.83099237	0.31	0.036	3.88E-96	11	PLS3
6.80E-96	1.073525726	0.398	0.062	2.28E-91	11	LGALS3BP
2.72E-95	1.222925397	0.425	0.071	9.13E-91	11	NRP1
2.48E-93	1.267614528	0.301	0.038	8.32E-89	11	NET1
4.23E-93	1.303326284	0.478	0.09	1.42E-88	11	COL18A1

1.23E-92	1.139701996	0.597	0.138	4.11E-88	11	SYNE2
4.24E-92	0.914195611	0.376	0.056	1.42E-87	11	NDRG2
2.33E-91	0.72149772	0.252	0.027	7.81E-87	11	NBL1
1.83E-90	0.87599059	0.288	0.035	6.14E-86	11	MPRIP
1.18E-89	1.863882299	0.27	0.032	3.95E-85	11	CXCL12
2.09E-85	0.829470919	0.27	0.032	7.01E-81	11	SORBS3
5.13E-85	0.903598343	0.358	0.056	1.72E-80	11	CRIM1
7.80E-84	0.731991973	0.292	0.038	2.62E-79	11	TJP1
1.22E-83	1.239317286	0.925	0.394	4.10E-79	11	MALAT1
1.06E-82	1.80991596	0.274	0.036	3.55E-78	11	FBLN5
2.68E-80	0.685911815	0.252	0.03	8.97E-76	11	IER5L
4.78E-79	1.035052176	0.416	0.079	1.60E-74	11	TPM4
1.37E-78	0.844336391	0.265	0.034	4.59E-74	11	SERPINH1
1.83E-78	1.504566572	0.739	0.269	6.14E-74	11	TPM1
1.62E-77	0.921965349	0.394	0.073	5.44E-73	11	PTEN
3.76E-74	1.263462179	0.407	0.081	1.26E-69	11	IL6ST
5.79E-74	1.007333129	0.398	0.078	1.94E-69	11	PPP1R12A
1.30E-71	1.189208402	0.473	0.111	4.36E-67	11	LPP
1.41E-70	1.29476713	0.677	0.253	4.71E-66	11	GNAS
8.03E-69	1.000491681	0.425	0.092	2.69E-64	11	LMO4
4.80E-68	0.956506411	0.265	0.039	1.61E-63	11	SORBS2
2.47E-66	1.148703701	0.531	0.151	8.28E-62	11	ACTN4
4.61E-66	0.727608371	0.301	0.049	1.54E-61	11	CAVIN3
7.50E-66	1.239760539	0.633	0.223	2.51E-61	11	SEPT7
2.43E-65	1.096456816	0.624	0.197	8.15E-61	11	DDX17
5.28E-65	0.812664386	0.265	0.04	1.77E-60	11	IFI27
1.30E-64	0.83628978	0.327	0.06	4.36E-60	11	UBA2
7.09E-64	1.030870569	0.31	0.054	2.38E-59	11	MARCKS
8.51E-64	1.03514345	0.434	0.102	2.85E-59	11	CD46
3.81E-62	0.96000777	0.367	0.076	1.28E-57	11	ROCK1
6.85E-62	0.584509519	0.274	0.043	2.30E-57	11	PTK2
1.78E-61	1.329502883	0.451	0.118	5.97E-57	11	SOD3
1.50E-60	0.836718855	0.332	0.063	5.03E-56	11	TACC1
1.58E-60	0.715537088	0.265	0.042	5.30E-56	11	GBP2
2.03E-60	0.805874392	0.305	0.054	6.81E-56	11	ITM2C
4.45E-60	1.044808056	0.54	0.163	1.49E-55	11	SELENOM
7.52E-60	0.791814692	0.35	0.069	2.52E-55	11	MT-ATP8
9.28E-60	0.679265543	0.252	0.038	3.11E-55	11	NPTN
1.49E-58	1.030106932	0.779	0.328	4.99E-54	11	HLA-B
1.50E-58	0.635948891	0.261	0.041	5.03E-54	11	SERPINI1

1.74E-58	0.960495878	0.389	0.086	5.85E-54	11	AKAP12
1.84E-56	0.989959014	0.447	0.117	6.15E-52	11	TLN1
3.14E-56	0.814903315	0.456	0.116	1.05E-51	11	ATRX
5.49E-56	1.010706522	0.487	0.137	1.84E-51	11	LMNA
5.98E-56	0.983635687	0.5	0.145	2.01E-51	11	LUC7L3
9.16E-56	0.912466814	0.257	0.043	3.07E-51	11	LIMA1
5.46E-54	0.965311413	0.305	0.06	1.83E-49	11	SYNE1
1.50E-53	0.939108531	0.845	0.477	5.04E-49	11	ACTG1
1.11E-52	0.813689292	0.363	0.081	3.74E-48	11	SLC38A2
1.62E-52	0.956923218	0.354	0.08	5.45E-48	11	NFIC
2.99E-52	0.914945034	0.513	0.151	1.00E-47	11	COX7A1
2.29E-50	0.254332861	0.655	0.21	7.67E-46	11	NEAT1
1.73E-49	0.822300971	0.27	0.051	5.82E-45	11	CDC42BPA
1.87E-48	0.768251241	0.31	0.067	6.29E-44	11	IFI6
2.33E-48	0.670162604	0.265	0.05	7.81E-44	11	PHACTR2
4.93E-48	0.936229027	0.465	0.138	1.65E-43	11	CD151
1.15E-47	0.640421277	0.292	0.059	3.84E-43	11	GSTM3
1.43E-46	0.88553449	0.327	0.076	4.78E-42	11	GOLGB1
3.81E-46	1.194892953	0.473	0.162	1.28E-41	11	TSC22D1
5.05E-46	0.978429683	0.438	0.13	1.69E-41	11	CSRP2
3.48E-44	0.6725818	0.279	0.059	1.17E-39	11	SERINC3
4.70E-44	0.69261904	0.265	0.053	1.58E-39	11	FERMT2
1.26E-43	0.727825713	0.46	0.138	4.23E-39	11	IFITM2
2.00E-43	1.043335326	0.456	0.148	6.70E-39	11	BCAM
3.33E-43	0.874562483	0.761	0.389	1.12E-38	11	HLA-A
7.65E-43	0.849303183	0.345	0.089	2.57E-38	11	ADD1
6.41E-42	0.656080639	0.305	0.07	2.15E-37	11	BOD1L1
1.50E-41	0.639779527	0.991	0.824	5.03E-37	11	MT-ND3
1.88E-41	0.704661925	0.327	0.081	6.31E-37	11	SERINC1
3.95E-41	0.970939978	0.5	0.177	1.32E-36	11	KTN1
5.82E-41	0.805308565	0.438	0.134	1.95E-36	11	CD99
8.01E-41	0.682260384	0.274	0.06	2.69E-36	11	NFE2L1
2.28E-40	0.706125781	0.659	0.258	7.63E-36	11	MTRNR2L12
2.91E-40	0.816979902	0.792	0.414	9.74E-36	11	MT-ND4L
7.76E-40	0.76481553	0.376	0.106	2.60E-35	11	TUBA1A
1.42E-39	0.726257866	0.319	0.08	4.75E-35	11	PCM1
3.96E-39	0.693174635	0.314	0.078	1.33E-34	11	CBX6
7.42E-39	0.752959579	0.314	0.08	2.49E-34	11	AKAP13
3.44E-38	0.97094125	0.69	0.377	1.15E-33	11	IFITM3
6.19E-38	0.651474665	0.288	0.069	2.08E-33	11	ZNF638

7.04E-38	1.05252125	0.549	0.231	2.36E-33	11	ZFP36L1
7.97E-38	0.551249225	0.261	0.057	2.67E-33	11	C9orf3
1.05E-37	0.91627597	0.491	0.182	3.51E-33	11	IGFBP4
3.93E-37	0.707483621	0.336	0.092	1.32E-32	11	PDLIM5
4.92E-37	0.618540533	0.274	0.064	1.65E-32	11	KMT2A
6.73E-37	0.842435728	0.965	0.84	2.26E-32	11	B2M
6.96E-37	0.670117313	0.496	0.174	2.33E-32	11	APP
2.68E-36	0.815614342	0.314	0.084	8.99E-32	11	SEPT11
2.81E-36	0.737620571	0.606	0.261	9.42E-32	11	S100A6
4.33E-36	0.62122074	0.31	0.081	1.45E-31	11	MACF1
4.36E-36	0.665363836	0.279	0.067	1.46E-31	11	PAWR
1.13E-34	0.66227479	0.332	0.093	3.80E-30	11	ANKRD11
1.25E-34	0.779026234	0.509	0.199	4.18E-30	11	SRRM2
2.09E-34	0.654303477	0.301	0.078	7.02E-30	11	H1FX
2.26E-34	0.689900954	0.252	0.059	7.57E-30	11	TRIM56
3.44E-34	0.337728181	0.845	0.457	1.15E-29	11	TMSB4X
2.16E-33	0.646141935	0.301	0.082	7.23E-29	11	IQGAP1
3.97E-33	0.678694997	0.336	0.099	1.33E-28	11	PJA2
6.64E-33	0.790795837	0.296	0.081	2.23E-28	11	CHD4
1.24E-32	0.531718245	0.252	0.06	4.17E-28	11	SUPT16H
2.80E-32	0.752659651	0.274	0.071	9.38E-28	11	CRTAP
2.93E-32	0.828820281	0.403	0.139	9.84E-28	11	SNRNP70
6.81E-32	0.564252856	0.296	0.081	2.28E-27	11	HLA-F
1.29E-31	0.860656589	0.336	0.104	4.33E-27	11	NORAD
2.25E-31	0.785210345	0.265	0.069	7.54E-27	11	ARMCX3
4.17E-31	0.812383068	0.664	0.353	1.40E-26	11	HLA-C
4.55E-30	0.628433248	0.292	0.083	1.52E-25	11	BPTF
4.64E-30	0.618757216	0.265	0.07	1.56E-25	11	VPS13C
5.62E-30	0.673864124	0.252	0.065	1.88E-25	11	GYPC
1.12E-29	0.619109845	0.345	0.11	3.74E-25	11	WASF2
1.54E-29	0.836803964	0.296	0.087	5.15E-25	11	RRBP1
1.93E-29	0.835881445	0.597	0.282	6.48E-25	11	HSP90B1
2.94E-29	0.623891528	0.341	0.11	9.85E-25	11	KIF5B
4.12E-29	0.5654154	0.261	0.069	1.38E-24	11	CCPG1
5.07E-29	0.715140257	0.456	0.178	1.70E-24	11	SEPT2
1.73E-28	0.812181017	0.602	0.294	5.79E-24	11	TXNIP
3.38E-28	0.602434664	0.336	0.108	1.13E-23	11	WSB1
2.56E-27	0.815102646	0.35	0.121	8.58E-23	11	EPS8
3.06E-27	0.570158588	0.274	0.08	1.03E-22	11	PHF14
3.15E-27	0.800840086	0.434	0.17	1.06E-22	11	PNISR

3.24E-27	0.486627703	0.301	0.09	1.09E-22	11	RCN2
3.77E-27	0.488791497	0.929	0.833	1.27E-22	11	ACTB
3.84E-27	0.60870208	0.274	0.079	1.29E-22	11	GLG1
5.01E-27	0.67230142	0.252	0.069	1.68E-22	11	CCND1
5.46E-27	0.556701773	0.358	0.12	1.83E-22	11	CYB5R3
1.15E-26	0.628834223	0.31	0.098	3.85E-22	11	DYNC1H1
2.80E-26	0.714547363	0.425	0.166	9.38E-22	11	FUS
3.09E-26	0.616947768	0.27	0.08	1.04E-21	11	NUDT4
3.76E-26	0.707714548	0.319	0.104	1.26E-21	11	NR3C1
4.68E-26	0.561723905	0.265	0.077	1.57E-21	11	NEMF
1.30E-25	0.659587045	0.535	0.241	4.37E-21	11	APLP2
2.46E-25	0.494173866	0.257	0.074	8.25E-21	11	MAP4
3.02E-25	0.535405489	0.252	0.071	1.01E-20	11	CPQ
4.08E-25	0.511625292	0.252	0.071	1.37E-20	11	PBX1
4.14E-25	0.706150956	0.513	0.24	1.39E-20	11	HNRNPA3
8.72E-25	0.59944538	0.265	0.079	2.92E-20	11	DYNC1LI2
1.02E-24	0.464721335	0.987	0.801	3.42E-20	11	MT-ND1
1.17E-24	0.526675485	0.527	0.23	3.93E-20	11	HLA-E
1.30E-24	0.800088768	0.389	0.15	4.37E-20	11	HSPA1A
1.37E-24	0.564289454	0.296	0.094	4.60E-20	11	STAT1
6.42E-24	0.57063797	0.296	0.097	2.15E-19	11	ZRANB2
8.43E-24	0.631442481	0.279	0.089	2.83E-19	11	PURA
1.43E-23	0.640507481	0.403	0.163	4.78E-19	11	MAGED2
1.98E-23	0.756927395	0.319	0.112	6.66E-19	11	NFIA
8.77E-23	0.643169783	0.31	0.109	2.94E-18	11	KMT2E
1.66E-22	0.570827576	0.301	0.103	5.57E-18	11	PNN
2.18E-22	0.715341192	0.491	0.227	7.30E-18	11	ZBTB20
2.46E-22	0.601815974	0.274	0.089	8.26E-18	11	ZBTB38
3.47E-22	0.580445685	0.305	0.107	1.16E-17	11	GNAI2
1.09E-21	0.515128108	0.257	0.081	3.67E-17	11	PCYOX1
1.14E-21	0.565893339	0.319	0.114	3.83E-17	11	OAZ2
1.19E-21	0.577668387	0.31	0.109	3.98E-17	11	SPAG9
2.87E-21	0.635882108	0.323	0.12	9.61E-17	11	AKAP9
4.68E-21	1.543091532	0.575	0.397	1.57E-16	11	ADIRF
6.19E-21	0.581724366	0.305	0.109	2.08E-16	11	RBM25
8.20E-21	0.520810022	0.257	0.083	2.75E-16	11	ARID1B
2.91E-20	0.597306839	0.358	0.146	9.74E-16	11	SF3B1
6.96E-20	0.576875479	0.252	0.084	2.33E-15	11	SDF4
7.02E-20	0.690289804	0.704	0.511	2.35E-15	11	CD63
8.15E-20	0.450655798	0.314	0.114	2.73E-15	11	LGALS3

9.52E-20	0.585956806	0.518	0.269	3.19E-15	11	C12orf57
3.41E-19	0.594572372	0.336	0.134	1.14E-14	11	HNRNPR
3.54E-19	0.604642321	0.27	0.095	1.19E-14	11	TBL1XR1
3.54E-19	0.568967555	0.265	0.092	1.19E-14	11	NKTR
3.78E-19	0.504731422	0.31	0.117	1.27E-14	11	PDLIM1
4.00E-19	0.633994835	0.376	0.159	1.34E-14	11	HSPA5
6.14E-19	0.509006303	0.593	0.315	2.06E-14	11	PSAP
7.36E-19	0.663145932	0.549	0.296	2.47E-14	11	SON
1.33E-18	0.538863804	0.372	0.159	4.46E-14	11	SCAF11
2.24E-18	0.524805382	0.257	0.09	7.52E-14	11	TPR
3.02E-18	0.553851211	0.27	0.099	1.01E-13	11	KPNB1
3.70E-18	0.418519398	0.27	0.095	1.24E-13	11	STAT3
4.90E-18	0.541111556	0.434	0.203	1.64E-13	11	CAST
9.30E-18	0.738924227	0.562	0.329	3.12E-13	11	SELENOW
9.83E-18	0.493861205	0.35	0.146	3.30E-13	11	HNRNPD
2.17E-17	0.542716524	0.305	0.121	7.27E-13	11	ANKRD12
2.36E-17	0.47365468	0.265	0.097	7.91E-13	11	SEC31A
3.80E-17	0.496544151	0.283	0.108	1.27E-12	11	RSRP1
3.86E-17	0.541665949	0.257	0.094	1.29E-12	11	TM9SF3
4.28E-17	0.474630759	0.987	0.856	1.44E-12	11	MT-ATP6
1.19E-16	0.453151981	0.252	0.091	4.00E-12	11	CCDC85B
1.25E-16	0.452215793	0.265	0.099	4.20E-12	11	ADAR
1.42E-16	0.54057528	0.314	0.13	4.75E-12	11	PRRC2C
1.52E-16	0.62453654	0.447	0.227	5.11E-12	11	HNRNPU
2.21E-16	0.458005132	0.305	0.122	7.41E-12	11	ILK
2.63E-16	0.543167528	0.35	0.15	8.82E-12	11	JAK1
6.27E-16	0.614774604	0.358	0.163	2.10E-11	11	GTF2I
6.99E-16	0.53363313	0.465	0.236	2.34E-11	11	ANXA5
9.85E-16	0.461923239	0.27	0.105	3.30E-11	11	BRD2
1.17E-15	0.545709955	0.743	0.629	3.94E-11	11	H3F3B
1.21E-15	0.838208231	0.252	0.1	4.07E-11	11	TBC1D1
1.74E-15	0.497641965	0.305	0.127	5.82E-11	11	HNRNPH3
1.85E-15	0.497192454	0.279	0.112	6.20E-11	11	NCOR1
2.01E-15	0.482807855	0.518	0.278	6.72E-11	11	BTG1
2.47E-15	0.404232828	0.252	0.095	8.28E-11	11	PABPN1
2.73E-15	0.596544006	0.407	0.2	9.17E-11	11	RARRES2
3.85E-15	0.484854296	0.416	0.203	1.29E-10	11	LEPROT
6.04E-15	0.357411045	0.867	0.662	2.03E-10	11	MT-ND5
9.94E-15	0.577672491	0.588	0.389	3.33E-10	11	PTMS
1.60E-14	0.373979996	0.27	0.106	5.38E-10	11	POLR2J3.1

1.68E-14	0.449251351	0.257	0.102	5.63E-10	11	FGD5-AS1
1.91E-14	0.855178304	0.805	0.654	6.41E-10	11	DSTN
2.34E-14	0.537493101	0.478	0.257	7.86E-10	11	TCEAL4
2.66E-14	0.449435282	0.265	0.107	8.90E-10	11	U2SURP
7.27E-14	0.388498855	0.274	0.113	2.44E-09	11	PICALM
1.14E-13	0.550084385	0.336	0.162	3.81E-09	11	CLTC
1.16E-13	0.32326133	0.363	0.168	3.88E-09	11	JUND
1.38E-13	0.489304741	0.403	0.203	4.62E-09	11	N4BP2L2
2.61E-13	0.534494318	0.332	0.158	8.74E-09	11	SFPQ
2.83E-13	0.453861672	0.27	0.115	9.48E-09	11	CHD9
3.08E-13	0.52640355	0.345	0.168	1.03E-08	11	AES
3.99E-13	0.559524723	0.518	0.321	1.34E-08	11	NAP1L1
4.98E-13	0.514969409	0.252	0.104	1.67E-08	11	SPART
5.67E-13	0.342078058	0.257	0.103	1.90E-08	11	TAPBP
8.07E-13	0.563803924	0.288	0.129	2.71E-08	11	SOX4
1.44E-12	0.526232716	0.677	0.487	4.84E-08	11	HNRNPA2B1
1.56E-12	0.40274304	0.265	0.114	5.23E-08	11	MBNL1
2.13E-12	0.329654538	0.296	0.133	7.15E-08	11	VAMP5
2.17E-12	0.457067646	0.252	0.107	7.28E-08	11	EIF4H
2.29E-12	0.522006694	0.381	0.197	7.69E-08	11	XRCC5
2.34E-12	0.670563074	0.473	0.277	7.83E-08	11	TUBA1B
2.87E-12	0.581168345	0.274	0.124	9.64E-08	11	EIF3A
3.14E-12	0.280883045	0.279	0.121	1.05E-07	11	CEBPB
3.30E-12	0.4201894	0.491	0.282	1.11E-07	11	YWHAQ
3.41E-12	0.553428025	0.381	0.199	1.14E-07	11	LAMP1
4.27E-12	0.414053863	0.372	0.185	1.43E-07	11	PDIA3
5.14E-12	0.45445852	0.314	0.152	1.72E-07	11	HNRNPH1
8.00E-12	0.467357562	0.518	0.301	2.68E-07	11	PPIB
1.35E-11	0.49070187	0.327	0.166	4.53E-07	11	HP1BP3
1.45E-11	0.500576592	0.341	0.17	4.86E-07	11	ARL6IP5
1.59E-11	0.406576713	0.323	0.159	5.32E-07	11	MTDH
1.84E-11	0.454585238	0.35	0.177	6.16E-07	11	SRSF11
2.43E-11	0.440394559	0.765	0.568	8.15E-07	11	CST3
2.92E-11	0.374932697	0.296	0.138	9.80E-07	11	GPBP1
4.09E-11	0.469754752	0.265	0.122	1.37E-06	11	EWSR1
4.37E-11	0.25969389	0.261	0.113	1.47E-06	11	STOM
4.42E-11	0.507227753	0.403	0.223	1.48E-06	11	CALR
5.78E-11	0.381215606	0.283	0.133	1.94E-06	11	MPHOSPH8
6.02E-11	0.477191802	0.301	0.147	2.02E-06	11	TTC3
7.44E-11	0.312429919	0.265	0.12	2.50E-06	11	HSPA1B

7.48E-11	0.471447737	0.398	0.22	2.51E-06	11	CANX
8.50E-11	0.459396571	0.748	0.603	2.85E-06	11	HMGB1
9.00E-11	0.321942218	0.296	0.139	3.02E-06	11	IFI27L2
1.01E-10	0.409495424	0.575	0.386	3.39E-06	11	DDX5
1.78E-10	0.304433678	0.942	0.9	5.98E-06	11	PTMA
2.01E-10	0.306644936	0.252	0.113	6.73E-06	11	ANXA2
2.42E-10	0.428712768	0.257	0.12	8.11E-06	11	GOLGA4
2.42E-10	0.341185665	0.341	0.175	8.13E-06	11	ANP32B
2.67E-10	0.39880043	0.296	0.145	8.94E-06	11	LARP7
2.98E-10	0.341063867	0.301	0.147	1.00E-05	11	RRAGA
3.77E-10	0.491184319	0.58	0.385	1.26E-05	11	SEC62
8.92E-10	0.451785521	0.292	0.147	2.99E-05	11	PRMT2
1.55E-09	0.353353341	0.292	0.145	5.20E-05	11	ARGLU1
2.48E-09	0.393152759	0.341	0.184	8.32E-05	11	NME3
3.05E-09	0.49244528	0.27	0.138	0.000102269	11	HDLBP
3.33E-09	0.415476806	0.54	0.354	0.000111634	11	NUCKS1
3.50E-09	0.29770454	0.301	0.154	0.000117389	11	CSNK1A1
4.83E-09	0.371431796	0.319	0.168	0.000161979	11	NFKBIA
8.01E-09	0.447093463	0.327	0.178	0.000268541	11	MSN
8.99E-09	0.393630145	0.735	0.639	0.000301382	11	CALM2
1.13E-08	0.29660655	0.296	0.153	0.000380042	11	GRN
1.42E-08	0.527503129	0.314	0.174	0.000475986	11	NFIB
1.54E-08	0.310201703	0.296	0.154	0.000515409	11	ATP6AP2
1.70E-08	0.318596751	0.261	0.132	0.000570898	11	SF1
1.87E-08	0.370190289	0.456	0.287	0.000625869	11	CNBP
2.49E-08	0.424288794	0.412	0.259	0.000834245	11	SH3BGRL
2.94E-08	0.256822581	0.385	0.222	0.000985811	11	ARPC5
3.65E-08	0.2754885	0.376	0.212	0.001223055	11	CD59
3.71E-08	0.399227918	0.252	0.129	0.001244334	11	TNRC6B
3.75E-08	0.380278693	0.288	0.154	0.001258648	11	BBX
4.91E-08	0.386229157	0.496	0.337	0.001647874	11	HNRNPDL
5.57E-08	0.366390112	0.429	0.266	0.001867195	11	MRFAP1
7.15E-08	0.323912304	0.265	0.138	0.002397488	11	CIR1
8.46E-08	0.282723292	0.327	0.179	0.002835728	11	NUPR1
8.52E-08	0.726112784	0.469	0.324	0.002856474	11	HIST1H4C
1.24E-07	0.533836033	0.27	0.148	0.004144577	11	JUN
1.31E-07	0.315645587	0.876	0.864	0.004390065	11	MYL6
1.47E-07	0.306745487	0.257	0.135	0.004914311	11	KDELR2
1.69E-07	0.395931541	0.372	0.227	0.005679233	11	PPP1CB
1.87E-07	0.406353885	0.292	0.165	0.006263351	11	HNRNPM

2.36E-07	0.34646194	0.394	0.248	0.007906522	11	VAMP2
2.86E-07	0.345454542	0.265	0.143	0.009592531	11	C5orf24
3.17E-07	0.258382286	0.35	0.201	0.010646633	11	NDFIP1
5.75E-07	0.335245006	0.372	0.227	0.01928373	11	TCF25
6.44E-07	0.256015298	0.478	0.313	0.021609173	11	NCL
6.61E-07	0.349117931	0.416	0.275	0.022161431	11	CSDE1
6.86E-07	0.383722316	0.655	0.526	0.023010863	11	HSP90AB1
8.11E-07	0.365594735	0.385	0.242	0.027201067	11	RBM39
9.38E-07	0.316181073	0.27	0.151	0.031448771	11	GNB2
1.50E-06	0.308247241	0.301	0.176	0.050316647	11	PPIG
2.18E-06	0.274919996	0.699	0.568	0.072991533	11	HSP90AA1
2.83E-06	0.539661675	0.27	0.162	0.094988876	11	THY1
3.08E-06	0.373093819	0.487	0.341	0.103297294	11	EIF4G2
3.95E-06	0.295213283	0.376	0.238	0.132525557	11	YBX3
5.99E-06	0.308082706	0.283	0.17	0.20073907	11	TMED10
6.83E-06	0.38918542	0.482	0.338	0.229031803	11	SERBP1
1.11E-05	0.344641498	0.619	0.502	0.373191979	11	CCNI
1.21E-05	0.257043995	0.367	0.234	0.406158889	11	CHURC1
1.79E-05	0.286580813	0.319	0.2	0.600845092	11	BZW1
1.82E-05	0.386605504	0.279	0.175	0.611316306	11	RBMX
2.19E-05	0.346699391	0.434	0.295	0.735054778	11	EIF4B
2.86E-05	0.361160764	0.367	0.25	0.960443218	11	DYNC1I2
5.21E-05	0.330248242	0.27	0.168	1	11	CAPNS1
6.57E-05	0.319467242	0.509	0.396	1	11	EID1
0.000137287	0.259422358	0.332	0.218	1	11	CAP1
0.000145135	0.309766401	0.265	0.169	1	11	KHDRBS1
0.000246047	0.285892731	0.257	0.163	1	11	SH3GLB1
0.00029048	0.34562156	0.381	0.263	1	11	TUBB
0.000310918	0.26908385	0.332	0.231	1	11	YWHAZ
0.001151584	0.299411986	0.288	0.204	1	11	CTSC
0.006552967	0.270074155	0.292	0.219	1	11	CBX3
0.008506226	0.311542613	0.412	0.336	1	11	HMGN2
0	6.037831731	0.896	0.047	0	12	IGHG4
0	5.966216416	0.887	0.024	0	12	IGHG1
0	5.770896193	0.835	0.031	0	12	IGHG3
0	3.911220132	0.73	0.014	0	12	JCHAIN
0	2.650412113	0.965	0.01	0	12	MZB1
0	1.866821838	0.852	0.007	0	12	DERL3
0	1.611611411	0.843	0.036	0	12	FKBP11
0	0.895932369	0.635	0.008	0	12	PDK1

0	0.880012698	0.652	0.019	0	12	TENT5C
0	0.8553304	0.661	0.006	0	12	SPAG4
0	0.849006808	0.504	0.001	0	12	JSRP1
0	0.78368235	0.617	0.005	0	12	TNFRSF17
0	0.658518225	0.287	0	0	12	IGLV3-1
0	0.621882883	0.609	0.012	0	12	FCRL5
0	0.57482251	0.548	0.019	0	12	POU2AF1
0	0.53489758	0.574	0.009	0	12	SLAMF7
0	0.525144122	0.522	0.011	0	12	CD38
0	0.46540705	0.443	0.01	0	12	ZBP1
0	0.401857074	0.409	0.001	0	12	AC012236.1
0	0.346122394	0.348	0.006	0	12	IRF4
0	0.311462002	0.478	0.015	0	12	HSPA13
0	0.281644714	0.443	0.007	0	12	MEI1
1.65E-273	1.066590735	0.748	0.042	5.52E-269	12	CD79A
5.00E-269	1.38692869	0.809	0.053	1.68E-264	12	ITM2C
9.13E-263	0.459916001	0.557	0.024	3.06E-258	12	LY96
2.08E-254	6.957012443	0.983	0.095	6.99E-250	12	IGKC
1.07E-251	0.370238325	0.496	0.02	3.60E-247	12	HSH2D
2.90E-246	0.270752515	0.461	0.018	9.74E-242	12	GAS6
6.80E-244	0.550357845	0.609	0.031	2.28E-239	12	CRELD2
1.79E-243	0.535728187	0.443	0.017	6.00E-239	12	PRDM1
1.17E-221	0.629022485	0.652	0.039	3.93E-217	12	SEL1L
3.86E-220	0.263642855	0.4	0.015	1.29E-215	12	TMEM238
4.03E-215	0.340521893	0.504	0.024	1.35E-210	12	PECAM1
1.16E-214	3.956730564	0.339	0.011	3.90E-210	12	IGHG2
4.84E-210	1.492293486	0.791	0.068	1.62E-205	12	PRDX4
6.20E-208	0.499643986	0.583	0.033	2.08E-203	12	TPST2
4.71E-207	0.695362748	0.678	0.045	1.58E-202	12	TXNDC15
8.83E-196	0.404729016	0.522	0.029	2.96E-191	12	ST6GALNAC4
2.08E-195	0.306395489	0.548	0.031	6.98E-191	12	ARSA
1.24E-187	0.601661263	0.609	0.04	4.17E-183	12	SEL1L3
5.70E-187	2.278182911	0.904	0.11	1.91E-182	12	XBP1
1.62E-186	0.279868873	0.461	0.023	5.42E-182	12	SPATS2
6.61E-183	0.254284255	0.409	0.019	2.22E-178	12	GLCCI1
5.40E-179	0.250627174	0.461	0.024	1.81E-174	12	CREB3L2
1.36E-178	6.178510988	0.557	0.038	4.56E-174	12	IGLC2
3.64E-176	0.33724698	0.443	0.023	1.22E-171	12	FBXW7
2.07E-175	0.343247778	0.513	0.031	6.95E-171	12	ST6GAL1
5.09E-170	1.090510407	0.791	0.076	1.71E-165	12	ISG20

4.21E-167	0.424288609	0.47	0.027	1.41E-162	12	RGCC
8.46E-166	4.645763388	0.391	0.02	2.84E-161	12	IGHA1
5.64E-163	0.305923285	0.4	0.02	1.89E-158	12	CSNK1E
6.44E-161	0.441946402	0.617	0.047	2.16E-156	12	SRPRB
7.21E-160	0.712763439	0.67	0.056	2.42E-155	12	NUCB2
1.00E-159	0.25157061	0.504	0.032	3.36E-155	12	GMPPB
1.02E-156	4.915857128	0.322	0.014	3.43E-152	12	IGLC3
5.35E-156	0.254998026	0.47	0.029	1.80E-151	12	ICAM2
2.00E-155	0.335203232	0.417	0.023	6.70E-151	12	SYVN1
1.08E-146	0.313699359	0.452	0.028	3.62E-142	12	TXNDC11
1.28E-146	0.298485169	0.409	0.024	4.30E-142	12	ADA2
1.49E-146	0.51493155	0.609	0.05	5.00E-142	12	PLPP5
3.78E-144	0.31073204	0.426	0.026	1.27E-139	12	TAPBPL
3.14E-142	0.729007854	0.713	0.069	1.05E-137	12	ERLEC1
5.00E-142	0.316339279	0.557	0.043	1.68E-137	12	SRPRA
6.39E-137	0.280855999	0.487	0.035	2.14E-132	12	SYTL1
7.82E-135	0.331365767	0.252	0.01	2.62E-130	12	NXPE3
1.56E-131	0.82840601	0.661	0.066	5.23E-127	12	BTG2
4.22E-119	0.250172382	0.417	0.03	1.42E-114	12	IFNAR2
4.52E-119	0.358268192	0.583	0.055	1.52E-114	12	DERL1
2.59E-113	0.84396598	0.809	0.109	8.70E-109	12	SPCS3
7.24E-113	0.52360836	0.635	0.067	2.43E-108	12	BST2
2.76E-112	0.434337643	0.513	0.046	9.25E-108	12	SEC61A1
3.47E-111	0.8438286	0.826	0.112	1.16E-106	12	MYDGF
2.50E-105	0.68257295	0.67	0.08	8.38E-101	12	UBE2J1
4.28E-105	1.196154446	0.843	0.14	1.43E-100	12	SEC11C
7.47E-100	0.793188809	0.739	0.102	2.50E-95	12	MANF
3.56E-99	0.876399642	0.739	0.103	1.19E-94	12	SELENOS
9.51E-99	0.523671197	0.687	0.086	3.19E-94	12	CLPTM1L
5.40E-98	0.53494749	0.67	0.083	1.81E-93	12	PDIA4
4.80E-97	3.875497931	0.417	0.037	1.61E-92	12	IGHM
2.25E-95	0.528971122	0.678	0.086	7.54E-91	12	RRBP1
3.64E-95	0.457317249	0.661	0.082	1.22E-90	12	HM13
9.00E-95	0.395580649	0.583	0.066	3.02E-90	12	KLF13
1.54E-94	0.803832781	0.765	0.113	5.16E-90	12	SDF2L1
4.16E-94	0.443394624	0.583	0.067	1.39E-89	12	IFI6
6.52E-93	0.716053173	0.73	0.104	2.19E-88	12	LMAN1
1.66E-92	0.487287313	0.426	0.039	5.58E-88	12	CD27
6.06E-92	0.494745039	0.33	0.024	2.03E-87	12	PIM2
5.85E-91	0.282337671	0.391	0.033	1.96E-86	12	IRF7

1.19E-90	2.508029857	0.974	0.382	3.99E-86	12	SSR4
2.93E-90	0.304344776	0.583	0.068	9.84E-86	12	CCPG1
1.69E-88	0.275557529	0.313	0.023	5.66E-84	12	AC243960.1
5.73E-88	0.647433689	0.774	0.117	1.92E-83	12	RPN2
1.82E-87	0.570811985	0.522	0.059	6.11E-83	12	FOSB
1.57E-85	0.256380544	0.513	0.057	5.27E-81	12	MGAT1
9.73E-85	0.260288138	0.548	0.064	3.26E-80	12	GYPC
1.71E-82	0.25413192	0.339	0.028	5.73E-78	12	CCDC69
1.26E-80	1.706763279	0.887	0.237	4.22E-76	12	HERPUD1
6.00E-78	0.287586608	0.322	0.027	2.01E-73	12	EDEM1
8.59E-77	0.321630778	0.565	0.073	2.88E-72	12	DERL2
1.32E-75	0.345523868	0.591	0.08	4.43E-71	12	ALG5
5.90E-74	0.25273671	0.461	0.053	1.98E-69	12	CYTIP
2.28E-72	0.322309164	0.591	0.083	7.66E-68	12	SDC1
1.31E-70	0.352668172	0.278	0.022	4.38E-66	12	RGS1
2.08E-70	0.395993142	0.678	0.107	6.99E-66	12	ICAM3
2.64E-66	1.04370317	0.774	0.159	8.86E-62	12	HSPA5
3.73E-66	0.405053043	0.67	0.11	1.25E-61	12	TMED9
8.90E-64	0.28729152	0.513	0.071	2.99E-59	12	EVI2B
6.04E-62	0.316116921	0.583	0.091	2.03E-57	12	DNAJC3
5.76E-60	0.285582203	0.539	0.081	1.93E-55	12	SLC38A2
2.57E-57	0.555955569	0.548	0.092	8.61E-53	12	FOS
2.97E-56	1.20817718	0.887	0.282	9.96E-52	12	HSP90B1
3.47E-55	0.377103975	0.67	0.124	1.16E-50	12	ATF4
1.38E-51	0.420928532	0.687	0.137	4.64E-47	12	HDLBP
3.67E-51	0.273037273	0.617	0.113	1.23E-46	12	RPN1
1.54E-50	0.434839434	0.722	0.149	5.15E-46	12	LMAN2
1.11E-49	0.252431805	0.478	0.077	3.71E-45	12	GOLGB1
4.20E-49	0.603614585	0.774	0.18	1.41E-44	12	PDIA6
4.08E-48	0.311777711	0.687	0.14	1.37E-43	12	P4HB
2.41E-47	0.372045038	0.661	0.134	8.10E-43	12	KDELR2
4.26E-47	0.397417291	0.713	0.152	1.43E-42	12	DNAJB9
8.41E-47	0.310265315	0.47	0.079	2.82E-42	12	H1FX
2.88E-44	0.8267965	0.557	0.12	9.65E-40	12	HSPA1B
2.51E-42	0.428157675	0.704	0.167	8.42E-38	12	JUND
4.59E-42	0.840425536	0.939	0.353	1.54E-37	12	HLA-C
1.01E-41	0.725957433	0.791	0.223	3.40E-37	12	SSR3
1.40E-40	0.940308601	0.591	0.147	4.70E-36	12	JUN
4.64E-39	0.385642563	0.704	0.168	1.56E-34	12	TMED10
3.50E-37	0.359224651	0.661	0.159	1.17E-32	12	MTDH

5.99E-36	0.290734095	0.678	0.163	2.01E-31	12	KDELR1
1.54E-35	0.305980966	0.73	0.183	5.16E-31	12	TRAM1
5.37E-35	0.758935957	0.861	0.301	1.80E-30	12	PPIB
2.62E-34	0.786490528	0.791	0.297	8.80E-30	12	SPCS2
9.68E-34	0.475669296	0.774	0.222	3.25E-29	12	CALR
5.24E-31	0.394472586	0.722	0.199	1.76E-26	12	OSTC
6.20E-30	0.267998047	0.357	0.069	2.08E-25	12	HLA-DQA2
1.13E-28	0.455975116	0.878	0.329	3.77E-24	12	HLA-B
1.35E-27	0.297550068	0.765	0.229	4.52E-23	12	ZNF706
9.46E-27	0.859147531	0.504	0.15	3.17E-22	12	HSPA1A
3.28E-25	0.278266949	0.539	0.147	1.10E-20	12	IER2
9.71E-21	0.459987851	0.826	0.328	3.26E-16	12	SERP1
1.62E-18	0.323080212	0.991	0.84	5.43E-14	12	B2M
2.27E-17	0.387555571	0.826	0.351	7.63E-13	12	SELENOK
7.77E-13	0.423983921	0.948	0.582	2.61E-08	12	CYBA
1.69E-08	0.344279939	0.852	0.487	0.000567357	12	SPCS1
0.003365767	0.285347245	0.878	0.61	1	12	SUB1
0	5.511585315	0.93	0.003	0	13	TPSB2
0	4.849616409	0.93	0.001	0	13	TPSAB1
0	4.261336184	0.912	0.001	0	13	CTSG
0	4.076845372	0.93	0.001	0	13	CPA3
0	3.471516656	0.93	0	0	13	MS4A2
0	2.836167478	0.877	0.001	0	13	HPGDS
0	2.786747246	0.912	0.019	0	13	RHEX
0	2.601074683	0.877	0	0	13	HDC
0	2.542339216	0.895	0.006	0	13	GATA2
0	2.39696633	0.912	0.003	0	13	KIT
0	2.371982221	0.895	0	0	13	SLC18A2
0	2.365855198	0.877	0.02	0	13	VWA5A
0	2.3114971	0.684	0	0	13	ADCYAP1
0	2.283470028	0.684	0	0	13	CMA1
0	2.157753724	0.86	0.001	0	13	IL1RL1
0	1.880092926	0.456	0.007	0	13	AREG
0	1.83605571	0.754	0.001	0	13	RGS13
0	1.61459373	0.737	0.015	0	13	ALOX5
0	1.536972336	0.702	0.007	0	13	IL18R1
0	1.50360955	0.684	0	0	13	AL662860.1
0	1.483439156	0.649	0.012	0	13	ADRB2
0	1.353445774	0.649	0	0	13	AL157895.1
0	1.323941887	0.632	0	0	13	TPSD1

0	1.321155446	0.509	0.002	0	13	LIF
0	1.253513177	0.632	0.002	0	13	SLC45A3
0	1.228121367	0.667	0.004	0	13	PTGS1
0	1.210286654	0.684	0.016	0	13	ATP6V0A2
0	1.204111852	0.596	0.003	0	13	CNRIP1
0	1.134087147	0.632	0.014	0	13	BACE2
0	1.128646365	0.509	0.008	0	13	CAVIN2
0	1.105991049	0.526	0.002	0	13	FCER1A
0	1.061380963	0.509	0.004	0	13	AC106739.1
0	1.044961122	0.579	0.003	0	13	P2RX1
0	1.026294894	0.474	0.001	0	13	MLPH
0	1.01996819	0.596	0.002	0	13	RAB27B
0	0.985683907	0.474	0.001	0	13	DHRS9
0	0.959863427	0.474	0.006	0	13	CSF2RB
0	0.959148892	0.579	0.009	0	13	PMP22
0	0.926674369	0.421	0	0	13	CDK15
0	0.887491812	0.298	0.002	0	13	PTGS2
0	0.870735879	0.421	0	0	13	GCSAML
0	0.870135032	0.474	0.001	0	13	AC020571.1
0	0.83536008	0.439	0.004	0	13	GPR34
0	0.783064894	0.333	0	0	13	CALB2
0	0.745355134	0.368	0	0	13	SVOPL
0	0.72616343	0.298	0.001	0	13	PIK3R6
0	0.684649433	0.351	0	0	13	CADPS
0	0.645982098	0.333	0.002	0	13	CATSPER1
0	0.55928849	0.281	0.001	0	13	NTRK1
1.23E-297	0.824968307	0.439	0.007	4.13E-293	13	C3AR1
4.15E-289	1.123632568	0.561	0.012	1.39E-284	13	HS6ST1
6.08E-289	1.18739542	0.526	0.011	2.04E-284	13	DLC1
1.65E-284	1.278003286	0.719	0.021	5.53E-280	13	BTK
3.62E-281	1.154851854	0.614	0.015	1.21E-276	13	RHOBTB3
1.33E-273	2.436069133	0.895	0.035	4.44E-269	13	SAMSN1
1.16E-271	0.77697075	0.368	0.005	3.90E-267	13	CD33
2.88E-271	1.240335619	0.649	0.018	9.66E-267	13	ARHGEF6
2.52E-255	2.021743302	0.702	0.022	8.46E-251	13	RGS1
9.57E-247	0.85119814	0.509	0.011	3.21E-242	13	MEIS2
7.59E-243	0.777185766	0.368	0.006	2.54E-238	13	ADGRE2
2.45E-241	0.559073371	0.298	0.004	8.21E-237	13	TMEM44
1.32E-230	0.957517738	0.491	0.012	4.42E-226	13	LPCAT2
4.27E-230	0.588214013	0.333	0.005	1.43E-225	13	PTAFR

1.06E-229	0.446297859	0.263	0.003	3.55E-225	13	P2RY14
1.02E-223	1.072686412	0.509	0.013	3.41E-219	13	GRAP2
1.64E-214	0.74464121	0.386	0.008	5.49E-210	13	ARMH1
2.23E-212	0.798835872	0.421	0.009	7.48E-208	13	TESPA1
7.67E-208	0.820588824	0.404	0.009	2.57E-203	13	FAM129B
5.45E-205	1.217204837	0.772	0.033	1.83E-200	13	LYL1
6.83E-204	1.395275515	0.596	0.02	2.29E-199	13	TENT5A
5.22E-188	2.297762337	0.93	0.057	1.75E-183	13	LTC4S
2.27E-186	0.83330552	0.474	0.013	7.60E-182	13	LAX1
2.17E-184	1.304646953	0.684	0.029	7.26E-180	13	CD84
3.50E-181	1.787990184	0.842	0.046	1.18E-176	13	ALOX5AP
5.01E-181	1.5865582	0.842	0.043	1.68E-176	13	FCER1G
7.09E-180	0.645158592	0.351	0.008	2.38E-175	13	GALNT6
2.88E-179	1.342884399	0.737	0.035	9.66E-175	13	BMP2K
4.58E-177	0.592819808	0.316	0.006	1.54E-172	13	SLC43A3
2.21E-173	0.867534536	0.456	0.013	7.40E-169	13	CLEC12A
2.91E-173	0.945731513	0.421	0.011	9.77E-169	13	SLC2A6
5.10E-173	0.752939934	0.368	0.009	1.71E-168	13	STXBP5
6.08E-165	2.39537258	0.912	0.062	2.04E-160	13	ANXA1
1.40E-156	1.010618834	0.614	0.027	4.68E-152	13	TMEM154
2.66E-155	0.842935948	0.561	0.022	8.93E-151	13	KLRG1
8.42E-151	0.879126366	0.404	0.012	2.82E-146	13	CD38
1.88E-150	0.951194473	0.596	0.026	6.31E-146	13	MBOAT7
4.35E-150	1.275275939	0.667	0.034	1.46E-145	13	ACSL4
3.22E-146	1.030793985	0.579	0.026	1.08E-141	13	HS3ST1
5.35E-145	0.541496176	0.298	0.007	1.79E-140	13	MARCH3
7.91E-145	0.727785974	0.351	0.009	2.65E-140	13	AKAP5
2.19E-144	1.87959561	0.789	0.051	7.33E-140	13	CD9
5.88E-138	0.571442207	0.281	0.006	1.97E-133	13	DUSP10
4.31E-133	1.067148304	0.526	0.024	1.45E-128	13	RPS6KA5
1.66E-129	1.310481732	0.842	0.061	5.57E-125	13	TIMP1
8.42E-129	0.859811069	0.825	0.055	2.83E-124	13	TYROBP
1.39E-128	0.6149212	0.316	0.009	4.68E-124	13	EMILIN2
6.15E-128	1.47007527	0.684	0.042	2.06E-123	13	SLC2A3
7.62E-127	1.471913746	0.649	0.037	2.55E-122	13	RGS2
6.84E-124	0.555560013	0.281	0.007	2.29E-119	13	INKA1
2.50E-122	1.015566344	0.439	0.018	8.37E-118	13	NR4A1
1.09E-120	1.015959786	0.474	0.021	3.65E-116	13	EGR1
6.54E-119	1.717254797	0.825	0.067	2.19E-114	13	BST2
1.30E-117	0.734696817	0.386	0.014	4.37E-113	13	SLC26A2

1.93E-117	0.648203031	0.368	0.013	6.48E-113	13	PLXNC1
4.80E-117	0.633732592	0.333	0.011	1.61E-112	13	STXBP6
9.28E-117	0.826516563	0.404	0.016	3.11E-112	13	LAIR1
8.64E-115	0.996988088	0.649	0.04	2.90E-110	13	GNPTAB
1.22E-114	0.719549908	0.333	0.011	4.09E-110	13	PLAT
3.87E-114	1.201701104	0.719	0.051	1.30E-109	13	C4orf48
6.38E-114	1.648446373	0.825	0.07	2.14E-109	13	CD44
3.55E-110	1.281804302	0.719	0.053	1.19E-105	13	ARHGAP18
1.61E-109	0.695367033	0.368	0.014	5.39E-105	13	ANTXR2
8.36E-109	0.838683547	0.509	0.027	2.80E-104	13	LAT2
5.63E-108	0.558111749	0.298	0.009	1.89E-103	13	SIGLEC10
3.36E-107	0.749083622	0.351	0.013	1.13E-102	13	FER
2.60E-106	1.037149422	0.596	0.037	8.72E-102	13	BHLHE40
7.08E-106	0.994089412	0.526	0.029	2.37E-101	13	CD82
3.52E-105	2.303842266	0.947	0.106	1.18E-100	13	SRGN
3.16E-104	1.580372733	0.825	0.075	1.06E-99	13	GLUL
2.58E-103	2.332799635	0.877	0.093	8.65E-99	13	LMO4
1.92E-101	0.884886936	0.614	0.04	6.45E-97	13	SLFN5
1.57E-99	1.625041148	0.719	0.06	5.27E-95	13	FOSB
1.05E-98	0.692260861	0.439	0.021	3.51E-94	13	KIAA1522
1.45E-97	0.601567971	0.298	0.01	4.87E-93	13	AC020916.1
2.98E-97	1.917516816	0.877	0.092	9.98E-93	13	FOS
3.17E-97	0.860288271	0.439	0.022	1.06E-92	13	SYTL2
1.19E-94	0.786867347	0.456	0.024	3.99E-90	13	ANKRD28
8.38E-91	0.833298849	0.509	0.031	2.81E-86	13	CCDC88A
4.72E-90	0.484501401	0.263	0.009	1.58E-85	13	EXTL3
1.14E-88	0.641429217	0.439	0.024	3.83E-84	13	CHD7
3.94E-87	1.024903444	0.509	0.033	1.32E-82	13	IL18
3.27E-85	0.425222687	0.263	0.009	1.10E-80	13	FHL3
1.28E-84	1.619433065	0.632	0.052	4.30E-80	13	CD69
6.30E-84	0.779817782	0.368	0.018	2.11E-79	13	AHR
8.57E-84	0.90837059	0.474	0.029	2.88E-79	13	LAT
1.37E-82	0.721062063	0.316	0.014	4.59E-78	13	RAB37
2.29E-82	0.583872554	0.333	0.015	7.67E-78	13	UNC13D
1.54E-81	0.641903737	0.316	0.014	5.17E-77	13	GM2A
1.92E-79	0.471843024	0.316	0.014	6.45E-75	13	ZNRF1
1.17E-77	1.017195521	0.632	0.055	3.91E-73	13	ITM2C
2.19E-77	1.309160116	0.789	0.089	7.35E-73	13	RAC2
4.39E-77	0.699982511	0.368	0.02	1.47E-72	13	SLC11A2
5.24E-77	1.022030272	0.649	0.059	1.76E-72	13	ZEB2

1.11E-76	0.929173572	0.509	0.037	3.73E-72	13	C1orf162
2.31E-76	0.804155369	0.456	0.03	7.75E-72	13	RASSF5
1.22E-75	1.262896559	0.719	0.076	4.09E-71	13	PTPN6
1.55E-75	0.670613283	0.526	0.039	5.21E-71	13	TIMP3
2.28E-75	0.666938784	0.421	0.026	7.65E-71	13	CTTNBP2
8.75E-74	0.991394609	0.526	0.041	2.93E-69	13	RHOH
9.01E-74	0.732970363	0.351	0.019	3.02E-69	13	NBEAL2
1.47E-73	0.856889237	0.368	0.021	4.93E-69	13	TTN
1.95E-73	0.416066919	0.263	0.011	6.56E-69	13	TNFRSF4
2.28E-73	1.026626946	0.509	0.039	7.65E-69	13	LEO1
1.62E-72	2.285833852	0.825	0.117	5.42E-68	13	HPGD
2.36E-72	0.859909023	0.544	0.044	7.93E-68	13	CHST12
2.75E-71	0.984416954	0.474	0.035	9.24E-67	13	TPST2
6.92E-71	1.156698032	0.702	0.074	2.32E-66	13	AHNAK
1.89E-70	1.164647049	0.719	0.08	6.34E-66	13	AKAP13
5.87E-70	0.435763908	0.263	0.011	1.97E-65	13	ALS2
6.79E-70	1.447018394	0.93	0.133	2.28E-65	13	S100A4
2.52E-69	0.520161161	0.298	0.014	8.46E-65	13	MCTP2
1.41E-68	0.709966699	0.368	0.022	4.73E-64	13	PRKX
2.34E-68	0.816646456	0.439	0.031	7.85E-64	13	RASSF1
2.40E-68	0.49475718	0.298	0.014	8.04E-64	13	OTULINL
1.36E-67	0.738775338	0.386	0.024	4.55E-63	13	PAG1
2.00E-67	0.56961872	0.368	0.022	6.70E-63	13	ESYT1
3.10E-67	1.124326571	0.772	0.092	1.04E-62	13	FXYD5
6.91E-67	0.712169949	0.439	0.031	2.32E-62	13	GPR65
3.90E-66	0.606792024	0.263	0.012	1.31E-61	13	DCBLD2
8.06E-65	0.744896898	0.439	0.032	2.70E-60	13	ATXN1
1.59E-64	0.551218131	0.281	0.014	5.34E-60	13	SMIM3
5.50E-63	0.709690577	0.439	0.033	1.84E-58	13	LYN
6.17E-63	1.030428238	0.596	0.06	2.07E-58	13	CELF2
1.11E-62	0.607839109	0.386	0.026	3.71E-58	13	SPN
1.85E-62	0.48450003	0.263	0.012	6.20E-58	13	PRAG1
9.67E-61	0.592300089	0.333	0.02	3.24E-56	13	AL390728.6
1.58E-60	1.18491192	0.789	0.105	5.28E-56	13	LAPTM5
6.25E-60	1.057103468	0.526	0.05	2.10E-55	13	KCNQ1OT1
1.25E-59	0.935543383	0.509	0.047	4.20E-55	13	RGS10
1.64E-59	0.630272501	0.333	0.021	5.49E-55	13	NR4A2
1.93E-59	0.793977784	0.526	0.049	6.48E-55	13	ITM2A
3.02E-59	1.19957138	0.649	0.077	1.01E-54	13	STX3
6.35E-59	0.575408994	0.421	0.032	2.13E-54	13	CXCL16

7.70E-59	0.676771828	0.368	0.025	2.58E-54	13	METRNL
1.13E-58	0.740170707	0.456	0.038	3.80E-54	13	KIDINS220
1.76E-57	0.63541001	0.368	0.025	5.91E-53	13	KRT19
6.20E-57	0.765575603	0.368	0.027	2.08E-52	13	UBA7
7.36E-57	1.091522323	0.614	0.071	2.47E-52	13	FOXP1
3.61E-56	1.009816287	0.667	0.084	1.21E-51	13	ELF1
8.20E-56	1.289456576	0.754	0.11	2.75E-51	13	EMP3
1.07E-55	0.619167185	0.263	0.014	3.57E-51	13	DAPL1
1.08E-55	0.911414044	0.579	0.063	3.63E-51	13	BTN3A2
2.07E-55	0.751091074	0.509	0.049	6.96E-51	13	GRK2
3.78E-55	0.697267655	0.368	0.027	1.27E-50	13	SMYD3
1.60E-53	1.266365809	0.807	0.128	5.36E-49	13	ARHGDIB
1.78E-53	1.604952852	0.825	0.147	5.97E-49	13	IER2
2.12E-53	0.772207854	0.509	0.051	7.10E-49	13	PPP1R15A
2.69E-53	1.903115894	0.965	0.241	9.02E-49	13	VIM
2.87E-53	0.647975302	0.404	0.033	9.64E-49	13	DENND1B
5.28E-53	0.530350506	0.333	0.023	1.77E-48	13	ATP2A3
1.22E-52	0.498914645	0.316	0.021	4.10E-48	13	C21orf91
1.44E-52	0.617993166	0.351	0.025	4.81E-48	13	IFI44
4.29E-52	0.984708643	0.614	0.075	1.44E-47	13	DDIT4
5.34E-52	0.633796404	0.333	0.023	1.79E-47	13	CD22
6.01E-52	1.200952353	0.772	0.121	2.02E-47	13	SERPINB1
3.09E-51	0.43815797	0.281	0.017	1.04E-46	13	MYADM
1.79E-50	1.02141777	0.421	0.038	6.01E-46	13	IER3
6.52E-50	0.659926877	0.404	0.035	2.19E-45	13	MYO9B
1.58E-49	0.429209128	0.298	0.02	5.28E-45	13	GMPR
1.66E-49	0.814933223	0.333	0.025	5.55E-45	13	TMEM173
3.13E-49	1.02370981	0.561	0.068	1.05E-44	13	BTG2
3.20E-49	0.616988068	0.333	0.025	1.07E-44	13	ACER3
9.75E-49	0.685267495	0.333	0.025	3.27E-44	13	ARHGAP25
1.72E-48	0.825930686	0.491	0.052	5.78E-44	13	INTS6
5.21E-48	0.766974315	0.439	0.042	1.75E-43	13	PTPN7
2.46E-47	0.662216841	0.316	0.023	8.27E-43	13	NCF4
6.52E-47	0.636193429	0.368	0.031	2.19E-42	13	EDEM2
6.79E-47	0.553340474	0.386	0.034	2.28E-42	13	CD55
6.99E-47	0.937488074	0.561	0.071	2.34E-42	13	IDS
9.64E-47	1.043789254	0.702	0.108	3.23E-42	13	RSRP1
3.79E-46	0.780852169	0.491	0.054	1.27E-41	13	PYCARD
5.36E-46	1.250017323	0.702	0.115	1.80E-41	13	LGALS3
1.20E-45	1.350439077	0.807	0.147	4.02E-41	13	JUN

1.24E-45	1.233661361	0.754	0.127	4.17E-41	13	DUSP1
1.95E-45	0.637610741	0.439	0.044	6.54E-41	13	CTR9
4.23E-45	0.593009201	0.333	0.027	1.42E-40	13	LPIN1
1.13E-44	0.640435899	0.386	0.035	3.78E-40	13	RASA2
1.26E-44	0.573854472	0.368	0.032	4.22E-40	13	CDKN2AIP
1.44E-44	0.493614842	0.263	0.017	4.82E-40	13	CCDC71L
1.85E-44	0.753069967	0.439	0.046	6.19E-40	13	ELK4
8.02E-44	0.932665665	0.509	0.062	2.69E-39	13	CTNNBL1
1.07E-43	1.218118581	0.737	0.127	3.58E-39	13	ZFP36
3.04E-43	1.344206419	0.947	0.231	1.02E-38	13	HLA-E
3.19E-43	0.708658197	0.439	0.047	1.07E-38	13	SEMA4D
6.32E-43	1.572448085	0.842	0.187	2.12E-38	13	JUNB
1.76E-42	0.477034939	0.281	0.02	5.90E-38	13	BTG3
1.86E-42	0.845981523	0.614	0.089	6.23E-38	13	AC245297.3
1.12E-41	0.802867677	0.333	0.029	3.77E-37	13	SOCS1
3.15E-41	0.521131618	0.263	0.019	1.06E-36	13	TRIM24
5.57E-41	0.534417489	0.316	0.026	1.87E-36	13	FAR1
6.28E-41	0.791490885	0.544	0.072	2.11E-36	13	RNF130
1.20E-40	0.692043035	0.368	0.036	4.03E-36	13	FERMT3
1.53E-40	1.194623054	0.825	0.178	5.12E-36	13	CAPG
3.49E-40	0.511754379	0.298	0.024	1.17E-35	13	HHEX
9.33E-40	0.844566223	0.667	0.109	3.13E-35	13	CRBN
1.39E-39	1.176713183	0.807	0.168	4.68E-35	13	JUND
2.77E-39	0.570547791	0.281	0.022	9.28E-35	13	SLC16A3
4.63E-39	0.498864634	0.333	0.03	1.55E-34	13	IFIT3
5.30E-39	0.563150174	0.368	0.036	1.78E-34	13	AC084033.3
5.70E-39	0.909226701	0.509	0.068	1.91E-34	13	IFI6
2.18E-38	0.441886375	0.281	0.022	7.30E-34	13	ANKRD36C
3.68E-38	0.525204608	0.333	0.031	1.23E-33	13	SKAP1
4.23E-38	0.687657166	0.439	0.051	1.42E-33	13	LYST
6.78E-38	0.454639896	0.263	0.02	2.27E-33	13	GSE1
8.77E-38	0.672473433	0.404	0.045	2.94E-33	13	SLC44A1
9.24E-38	0.398743556	0.263	0.02	3.10E-33	13	ZNF7
9.84E-38	1.285874207	0.754	0.15	3.30E-33	13	HSPA1A
1.36E-37	0.523207194	0.316	0.028	4.55E-33	13	LCP2
2.03E-37	0.720418699	0.561	0.082	6.80E-33	13	HLA-F
3.02E-37	1.063993071	0.947	0.262	1.01E-32	13	S100A6
3.13E-37	0.913986055	0.544	0.078	1.05E-32	13	LCP1
3.80E-37	0.402278823	0.263	0.02	1.27E-32	13	TTLL7
4.84E-37	0.46025913	0.298	0.026	1.62E-32	13	ZNF430

6.10E-37	0.395697978	0.263	0.02	2.04E-32	13	PHTF2
7.26E-37	0.380358208	0.281	0.023	2.44E-32	13	LSG1
7.33E-37	0.613531818	0.404	0.045	2.46E-32	13	IER5
1.23E-36	0.617591235	0.333	0.032	4.13E-32	13	SPNS1
2.23E-36	0.462367036	0.298	0.026	7.47E-32	13	DOCK2
3.18E-36	0.516798854	0.333	0.032	1.07E-31	13	LGALS8
5.27E-36	0.503307813	0.298	0.027	1.77E-31	13	BTN3A1
9.78E-36	0.822648109	0.439	0.055	3.28E-31	13	DUSP6
1.28E-35	1.267778584	0.737	0.16	4.30E-31	13	HSPA5
1.29E-35	0.814986783	0.544	0.082	4.31E-31	13	SLC38A2
2.01E-35	0.953599322	0.632	0.111	6.76E-31	13	CD81
2.48E-35	0.724908946	0.386	0.044	8.31E-31	13	TMEM87A
3.70E-35	0.718211866	0.719	0.126	1.24E-30	13	CD52
5.12E-35	0.73741848	0.912	0.212	1.72E-30	13	NEAT1
8.41E-35	0.719103024	0.351	0.038	2.82E-30	13	XAF1
2.40E-34	0.712286643	0.614	0.098	8.05E-30	13	CD48
2.58E-34	0.740899215	0.351	0.038	8.64E-30	13	PARP4
4.12E-34	0.692190507	0.632	0.104	1.38E-29	13	CD37
5.42E-34	0.455121302	0.333	0.034	1.82E-29	13	ABHD2
1.48E-33	1.042560203	0.561	0.093	4.98E-29	13	RAB32
2.15E-33	0.558937821	0.333	0.035	7.22E-29	13	CCDC14
3.28E-33	0.471219408	0.281	0.025	1.10E-28	13	DOCK11
4.39E-33	0.598280617	0.368	0.041	1.47E-28	13	CST7
4.40E-33	1.373002767	0.947	0.355	1.48E-28	13	HLA-C
8.14E-33	0.887597542	0.667	0.127	2.73E-28	13	CKLF
8.20E-33	1.244784806	0.632	0.12	2.75E-28	13	HSPA1B
9.37E-33	0.520486771	0.298	0.029	3.14E-28	13	PIK3CA
1.02E-32	0.485775438	0.316	0.032	3.42E-28	13	SUSD6
1.75E-32	0.703707711	0.526	0.082	5.88E-28	13	MACF1
6.25E-32	0.663108763	0.561	0.092	2.10E-27	13	NKTR
1.00E-31	1.634232317	0.93	0.512	3.36E-27	13	CD63
1.39E-31	0.691725402	0.368	0.044	4.65E-27	13	BAZ2B
1.60E-31	1.355773868	0.93	0.33	5.36E-27	13	HLA-B
1.89E-31	0.364274589	0.281	0.026	6.34E-27	13	ABCA1
1.93E-31	0.535140762	0.474	0.068	6.46E-27	13	RNASEH2B
1.95E-31	0.504352884	0.474	0.067	6.54E-27	13	IKZF1
2.14E-31	0.371973869	0.298	0.029	7.18E-27	13	SOCS6
5.02E-31	0.590683558	0.474	0.07	1.68E-26	13	MIER1
7.11E-31	0.455621642	0.263	0.024	2.38E-26	13	ZNF92
2.27E-30	0.46881325	0.351	0.041	7.63E-26	13	POGZ

3.83E-30	0.525239825	0.368	0.045	1.29E-25	13	ALAS1
3.86E-30	0.486046601	0.316	0.034	1.29E-25	13	CHD3
4.16E-30	0.712046182	0.456	0.068	1.40E-25	13	EFCAB14
6.51E-30	0.572885107	0.649	0.118	2.18E-25	13	PTPRC
1.16E-29	0.452488354	0.263	0.025	3.90E-25	13	RRP1B
1.45E-29	0.435179956	0.281	0.028	4.85E-25	13	TNIK
2.66E-29	0.591026329	0.351	0.043	8.93E-25	13	SUPT20H
3.99E-29	0.478396082	0.281	0.029	1.34E-24	13	DOCK10
5.15E-29	0.479917992	0.351	0.043	1.73E-24	13	PSMB8-AS1
5.78E-29	0.46047135	0.298	0.032	1.94E-24	13	MITF
6.83E-29	0.617642718	0.526	0.087	2.29E-24	13	IFI16
8.33E-29	0.39001843	0.263	0.025	2.79E-24	13	GNAI1
8.68E-29	1.185826941	0.842	0.263	2.91E-24	13	CLU
8.69E-29	0.623358292	0.386	0.051	2.92E-24	13	PPT1
8.78E-29	0.612381813	0.579	0.104	2.95E-24	13	CD46
9.20E-29	0.789883607	0.684	0.147	3.08E-24	13	LUC7L3
1.19E-28	0.503125349	0.439	0.064	4.00E-24	13	TUT4
1.33E-28	1.173387648	0.947	0.391	4.47E-24	13	HLA-A
2.32E-28	0.613169949	0.456	0.071	7.78E-24	13	VPS13C
2.41E-28	0.576428753	0.596	0.11	8.08E-24	13	RBM25
3.42E-28	0.718262042	0.596	0.115	1.15E-23	13	CHD9
3.53E-28	0.667271878	0.526	0.091	1.18E-23	13	CCDC85B
4.06E-28	0.762650727	0.474	0.078	1.36E-23	13	ISG15
4.91E-28	1.061956048	0.632	0.133	1.65E-23	13	TSC22D3
7.04E-28	0.515650442	0.333	0.041	2.36E-23	13	BCAP29
8.40E-28	0.533488012	0.298	0.034	2.82E-23	13	ANKRD13D
1.06E-27	0.659423689	0.579	0.107	3.56E-23	13	GNAI2
1.98E-27	0.754650796	0.632	0.13	6.63E-23	13	PRRC2C
6.00E-27	0.584109105	0.368	0.051	2.01E-22	13	MIA3
7.75E-27	0.586076672	0.491	0.083	2.60E-22	13	M6PR
1.15E-26	0.33382656	0.298	0.034	3.85E-22	13	TMEM220
1.22E-26	0.675134782	0.526	0.095	4.09E-22	13	STAT1
1.39E-26	0.524975748	0.456	0.072	4.66E-22	13	PBX1
1.74E-26	0.52384149	0.386	0.056	5.83E-22	13	RBM5
3.23E-26	0.595512196	0.474	0.08	1.08E-21	13	PHF14
3.67E-26	0.426630978	0.368	0.05	1.23E-21	13	AP1S2
3.86E-26	0.866642181	0.912	0.278	1.29E-21	13	BTG1
3.94E-26	0.360489548	0.263	0.028	1.32E-21	13	ZNF37A
5.75E-26	0.814809585	0.702	0.17	1.93E-21	13	ARL6IP5
8.11E-26	0.943578692	0.702	0.18	2.72E-21	13	SEPT2

1.02E-25	0.697646952	0.421	0.068	3.42E-21	13	PHIP
1.20E-25	0.682316809	0.474	0.083	4.03E-21	13	BPTF
1.25E-25	0.60185541	0.404	0.062	4.19E-21	13	TRIP11
2.01E-25	0.693745507	0.386	0.058	6.74E-21	13	UBTF
2.72E-25	1.151479791	0.982	0.398	9.12E-21	13	MALAT1
3.40E-25	0.372164174	0.298	0.036	1.14E-20	13	INPP5D
4.80E-25	0.616361771	0.474	0.084	1.61E-20	13	PPP1R10
6.56E-25	1.265584628	0.982	0.84	2.20E-20	13	B2M
7.11E-25	0.491345591	0.281	0.033	2.38E-20	13	NCOA1
7.52E-25	0.966323906	0.702	0.186	2.52E-20	13	PDIA3
7.53E-25	0.367983676	0.509	0.089	2.52E-20	13	HCST
8.88E-25	0.607142399	0.368	0.055	2.98E-20	13	VEGFB
9.44E-25	0.431616484	0.368	0.053	3.17E-20	13	IFNGR1
1.02E-24	0.344273275	0.298	0.036	3.42E-20	13	HPS4
1.59E-24	0.664554161	0.491	0.09	5.33E-20	13	CCNL1
3.45E-24	0.414338176	0.333	0.045	1.16E-19	13	SWAP70
4.02E-24	0.583708852	0.404	0.064	1.35E-19	13	TGFB1
4.50E-24	0.375574217	0.281	0.033	1.51E-19	13	DNAJC9
8.61E-24	0.396450573	0.421	0.068	2.89E-19	13	GSN
8.61E-24	0.488315611	0.263	0.03	2.89E-19	13	BACH1
8.80E-24	0.481957734	0.351	0.05	2.95E-19	13	CARD16
1.24E-23	0.496366173	0.491	0.088	4.15E-19	13	CD53
1.29E-23	0.5690942	0.421	0.069	4.34E-19	13	HSD17B12
1.34E-23	0.730225786	0.544	0.113	4.48E-19	13	XBP1
1.37E-23	0.406477489	0.298	0.038	4.60E-19	13	NCKAP1L
1.43E-23	0.37159304	0.298	0.038	4.80E-19	13	NIN
1.55E-23	0.372542	0.281	0.034	5.19E-19	13	SOS2
1.85E-23	0.532499442	0.456	0.081	6.22E-19	13	PDCD4
2.40E-23	0.525423934	0.386	0.061	8.06E-19	13	PLD3
2.56E-23	0.527485443	0.421	0.071	8.57E-19	13	RHOG
3.05E-23	0.57837205	0.439	0.077	1.02E-18	13	HEXIM1
3.99E-23	0.569412949	0.439	0.077	1.34E-18	13	POLR3GL
4.19E-23	0.386821366	0.263	0.031	1.40E-18	13	TFB1M
4.51E-23	0.429353832	0.351	0.052	1.51E-18	13	LNPEP
5.00E-23	1.161625601	0.825	0.294	1.68E-18	13	CTSD
5.20E-23	0.509200317	0.491	0.092	1.74E-18	13	DNAJC3
7.38E-23	0.366928224	0.263	0.031	2.47E-18	13	CSNK2A2
7.86E-23	0.617963483	0.439	0.078	2.64E-18	13	ROCK1
8.16E-23	0.559763233	0.316	0.043	2.74E-18	13	ARL8A
9.98E-23	0.525718585	0.456	0.083	3.35E-18	13	TAOK3

1.42E-22	0.476049059	0.316	0.044	4.78E-18	13	TIA1
1.79E-22	0.463618046	0.281	0.036	5.99E-18	13	BASP1
1.98E-22	0.90610083	0.982	0.629	6.64E-18	13	H3F3B
2.67E-22	0.832593703	0.649	0.168	8.94E-18	13	NFKBIA
2.69E-22	0.353801987	0.298	0.039	9.04E-18	13	ITGA4
4.63E-22	0.68070502	0.509	0.104	1.55E-17	13	MANF
4.98E-22	0.959051147	0.561	0.129	1.67E-17	13	SOX4
5.62E-22	0.978061404	1	0.825	1.88E-17	13	MT-ND3
6.00E-22	0.439091408	0.421	0.072	2.01E-17	13	EVI2B
6.52E-22	0.472579815	0.333	0.05	2.19E-17	13	NUTM2B-AS1
1.08E-21	0.419887125	0.263	0.033	3.64E-17	13	AGO3
1.32E-21	0.460043371	0.298	0.041	4.42E-17	13	PARVB
1.43E-21	0.875541206	0.702	0.187	4.81E-17	13	ZFP36L2
1.61E-21	0.4639406	0.281	0.037	5.39E-17	13	SPEN
1.62E-21	0.651801984	0.737	0.2	5.43E-17	13	SRRM2
2.08E-21	0.387002156	0.263	0.033	6.98E-17	13	TLE3
2.21E-21	0.874451101	0.877	0.295	7.42E-17	13	TXNIP
2.29E-21	0.478547302	0.386	0.065	7.67E-17	13	KMT2A
2.35E-21	0.47192382	0.351	0.055	7.89E-17	13	MAZ
2.48E-21	0.442662488	0.316	0.046	8.30E-17	13	PAQR5
2.59E-21	0.388021186	0.316	0.045	8.68E-17	13	XRRA1
3.07E-21	0.944380466	1	0.856	1.03E-16	13	MT-ATP6
3.85E-21	0.642115165	0.351	0.057	1.29E-16	13	CCAR1
4.20E-21	0.412858509	0.333	0.051	1.41E-16	13	ANKRD44
4.52E-21	0.366503655	0.351	0.055	1.51E-16	13	PSMD1
4.52E-21	0.397155525	0.614	0.139	1.52E-16	13	IFITM2
5.21E-21	0.480129004	0.368	0.061	1.75E-16	13	TOP2B
5.94E-21	0.740693306	1	0.9	1.99E-16	13	PTMA
6.29E-21	0.390861707	0.439	0.08	2.11E-16	13	THUMPD1
7.59E-21	0.571262466	0.439	0.084	2.55E-16	13	ARID4B
8.12E-21	0.549403018	0.316	0.048	2.72E-16	13	USP48
8.17E-21	0.518747608	0.404	0.072	2.74E-16	13	USP15
8.55E-21	0.485728894	0.421	0.077	2.87E-16	13	CDK5RAP3
9.33E-21	0.596712864	0.316	0.048	3.13E-16	13	GATAD1
1.05E-20	0.415921909	0.368	0.061	3.52E-16	13	TTC14
1.30E-20	0.438494278	0.281	0.038	4.36E-16	13	IRF2BPL
1.51E-20	0.587617559	0.386	0.07	5.07E-16	13	ZNF638
1.55E-20	0.608325589	0.544	0.121	5.21E-16	13	ANKRD12
1.90E-20	0.408451906	0.298	0.043	6.39E-16	13	ARHGAP30
2.04E-20	0.474514879	0.439	0.084	6.84E-16	13	PHF3

2.21E-20	0.379945741	0.333	0.052	7.42E-16	13	ARID4A
2.53E-20	0.609186156	0.649	0.159	8.50E-16	13	MAOB
2.70E-20	0.445536137	0.263	0.035	9.04E-16	13	HLTF
4.21E-20	0.745465243	0.491	0.105	1.41E-15	13	BRD2
4.55E-20	0.606159627	0.474	0.097	1.53E-15	13	IDI1
4.91E-20	0.431876328	0.298	0.044	1.65E-15	13	HERC1
6.02E-20	0.500028042	0.491	0.101	2.02E-15	13	PRNP
7.27E-20	0.309275651	0.263	0.035	2.44E-15	13	MORC3
9.30E-20	0.449373477	0.421	0.079	3.12E-15	13	SEPT9
1.03E-19	0.535294339	0.351	0.06	3.47E-15	13	BAZ1A
1.65E-19	0.414915444	0.368	0.064	5.55E-15	13	EXOC7
1.73E-19	0.392916812	0.298	0.044	5.79E-15	13	MYO1F
1.99E-19	0.51882459	0.263	0.037	6.67E-15	13	SAMD9L
2.13E-19	0.642897251	0.474	0.103	7.15E-15	13	MAPRE1
2.24E-19	0.551626833	0.351	0.061	7.50E-15	13	PHF20
2.35E-19	0.613242039	0.491	0.11	7.89E-15	13	KMT2E
3.64E-19	0.414742327	0.316	0.05	1.22E-14	13	EXOSC8
4.71E-19	0.566865567	0.298	0.046	1.58E-14	13	TSPAN4
5.51E-19	0.692494145	0.368	0.066	1.85E-14	13	CLK1
6.68E-19	0.563559647	0.456	0.096	2.24E-14	13	STK4
7.35E-19	0.450162138	0.421	0.082	2.46E-14	13	RTN3
8.96E-19	0.631706477	0.386	0.074	3.00E-14	13	DDIT3
9.08E-19	0.359460431	0.298	0.045	3.05E-14	13	HOXB2
9.19E-19	0.535074742	0.491	0.109	3.08E-14	13	CMTM6
9.82E-19	0.612118737	0.491	0.111	3.29E-14	13	SPCS3
1.03E-18	0.608046954	0.404	0.079	3.44E-14	13	SYNGR2
1.19E-18	0.528865945	0.404	0.078	3.99E-14	13	CPM
1.21E-18	0.535527459	0.316	0.051	4.06E-14	13	FOSL2
1.25E-18	0.398096274	0.281	0.042	4.19E-14	13	PRDM2
1.30E-18	0.615507233	0.596	0.153	4.37E-14	13	PSMB8
1.55E-18	0.604711084	0.702	0.204	5.21E-14	13	N4BP2L2
1.60E-18	0.442848833	0.316	0.052	5.36E-14	13	BDP1
1.92E-18	0.484213587	0.456	0.097	6.43E-14	13	DHRS7
3.21E-18	0.389819338	0.404	0.077	1.08E-13	13	TUBGCP2
3.26E-18	0.349447664	0.263	0.038	1.09E-13	13	GTF2H1
3.78E-18	0.532739807	0.526	0.126	1.27E-13	13	LRRFIP1
4.13E-18	0.385530326	0.298	0.047	1.39E-13	13	TTC39B
4.89E-18	0.348036844	0.298	0.047	1.64E-13	13	SEC61A1
5.67E-18	0.50776289	0.333	0.059	1.90E-13	13	NIPBL
6.06E-18	0.516609115	0.649	0.172	2.03E-13	13	PNISR

6.57E-18	0.478726772	0.351	0.064	2.20E-13	13	ZNF292
7.17E-18	0.43706387	0.368	0.068	2.41E-13	13	UBE2L6
1.07E-17	0.366977383	0.982	0.459	3.60E-13	13	TMSB4X
1.17E-17	0.628122823	0.684	0.199	3.93E-13	13	DDX17
1.38E-17	0.546431374	0.509	0.121	4.62E-13	13	AKAP9
1.50E-17	0.43563842	0.298	0.049	5.04E-13	13	CHORDC1
1.55E-17	0.410197552	0.368	0.07	5.18E-13	13	EPC1
1.55E-17	0.424844257	0.368	0.069	5.21E-13	13	SMCHD1
1.59E-17	0.39707862	0.298	0.048	5.34E-13	13	MIR4435-2HG
1.67E-17	0.434473888	0.298	0.049	5.60E-13	13	VPS13A
1.81E-17	0.440568059	0.333	0.059	6.06E-13	13	HNRNPL
1.85E-17	0.465649229	0.404	0.082	6.19E-13	13	MESD
2.16E-17	0.610144796	0.526	0.131	7.24E-13	13	DPP7
2.17E-17	0.475368275	0.404	0.083	7.27E-13	13	IQGAP1
2.21E-17	0.374658548	0.368	0.069	7.42E-13	13	POLR2B
2.22E-17	0.571925105	0.614	0.167	7.44E-13	13	FUS
2.76E-17	0.404281686	0.404	0.081	9.27E-13	13	PCM1
3.32E-17	0.517422092	0.526	0.129	1.11E-12	13	THRAP3
3.66E-17	0.405969475	0.333	0.06	1.23E-12	13	SERINC3
4.24E-17	0.523199829	0.614	0.164	1.42E-12	13	TSC22D1
4.51E-17	0.782606932	0.772	0.278	1.51E-12	13	ASAH1
5.00E-17	0.482853722	0.561	0.141	1.68E-12	13	MLEC
5.07E-17	0.471063156	0.439	0.096	1.70E-12	13	STAT3
6.07E-17	0.44865394	0.474	0.108	2.04E-12	13	RAD21
6.51E-17	0.272913741	0.368	0.07	2.18E-12	13	MT-ATP8
7.51E-17	0.310303012	0.263	0.04	2.52E-12	13	MUM1
7.82E-17	0.443293614	0.333	0.061	2.62E-12	13	ZC3H13
7.85E-17	0.385889364	0.281	0.045	2.63E-12	13	ARRB2
8.16E-17	0.316607938	0.263	0.04	2.74E-12	13	ELMSAN1
8.39E-17	0.33151102	0.368	0.07	2.81E-12	13	KPNA4
9.61E-17	0.485659961	0.368	0.073	3.22E-12	13	AGTRAP
9.72E-17	0.383728462	0.474	0.105	3.26E-12	13	NDFIP2
9.79E-17	0.4267388819	0.316	0.056	3.28E-12	13	KMT2C
1.01E-16	0.354386188	0.281	0.045	3.40E-12	13	EPB41L1
1.02E-16	0.541802854	0.386	0.078	3.42E-12	13	NEMF
1.05E-16	0.410239018	0.316	0.056	3.53E-12	13	NABP1
1.23E-16	0.450901014	0.404	0.084	4.12E-12	13	BLOC1S6
1.26E-16	0.37856431	0.333	0.061	4.24E-12	13	SUZ12
1.48E-16	0.586969694	0.544	0.141	4.97E-12	13	P4HB
1.91E-16	0.60194278	0.491	0.121	6.39E-12	13	GOLGA4

2.08E-16	0.374879139	0.263	0.041	6.99E-12	13	APMAP
2.17E-16	0.355270652	0.263	0.041	7.28E-12	13	RYBP
2.40E-16	0.388443044	0.281	0.047	8.05E-12	13	DKC1
2.44E-16	0.413611786	0.316	0.057	8.19E-12	13	ACBD3
2.48E-16	0.400484502	0.316	0.056	8.32E-12	13	TMX4
2.61E-16	0.572703664	0.526	0.136	8.76E-12	13	CD99
2.80E-16	0.490424125	0.316	0.057	9.41E-12	13	FNBP4
3.24E-16	0.637292674	0.772	0.26	1.09E-11	13	ID2
3.34E-16	0.768680366	0.877	0.387	1.12E-11	13	DDX5
3.54E-16	0.342386319	0.298	0.052	1.19E-11	13	PLCG2
3.71E-16	0.51685025	0.351	0.07	1.25E-11	13	ARMCX3
4.42E-16	0.513095625	0.421	0.094	1.48E-11	13	FAM49B
4.57E-16	0.993498958	0.561	0.164	1.53E-11	13	GADD45B
4.57E-16	0.639682618	0.474	0.118	1.53E-11	13	ATRX
4.81E-16	0.774267069	0.579	0.16	1.61E-11	13	MTDH
5.56E-16	0.603359173	0.719	0.228	1.87E-11	13	HNRNPU
5.78E-16	0.520490959	0.421	0.094	1.94E-11	13	MYH9
6.17E-16	0.402221697	0.386	0.081	2.07E-11	13	TPM4
6.26E-16	0.39878382	0.316	0.058	2.10E-11	13	UFL1
7.14E-16	0.5773108	0.702	0.22	2.39E-11	13	CANX
8.40E-16	0.458258687	0.368	0.076	2.82E-11	13	LIMS1
9.41E-16	0.524264908	0.421	0.094	3.16E-11	13	IRF1
9.95E-16	0.698069847	1	0.916	3.34E-11	13	MT-CO2
1.01E-15	0.447081616	0.526	0.133	3.39E-11	13	MPHOSPH8
1.28E-15	0.64526986	0.526	0.146	4.29E-11	13	ARGLU1
1.31E-15	0.46649048	0.368	0.075	4.38E-11	13	NMT2
1.32E-15	0.419950685	0.386	0.082	4.42E-11	13	SERINC1
1.33E-15	0.355510346	0.316	0.058	4.47E-11	13	MGAT1
1.57E-15	0.529484275	0.456	0.109	5.28E-11	13	WSB1
1.63E-15	0.351640751	0.263	0.043	5.47E-11	13	FAM208A
1.66E-15	0.811584277	0.807	0.327	5.55E-11	13	SARAF
1.90E-15	0.356595324	0.333	0.064	6.38E-11	13	STT3B
2.05E-15	0.610465702	0.614	0.187	6.89E-11	13	OSBPL8
2.14E-15	0.516951508	0.439	0.104	7.17E-11	13	PNN
2.40E-15	0.36619361	0.281	0.049	8.06E-11	13	CREB1
2.79E-15	0.697807585	1	0.803	9.37E-11	13	MT-ND1
3.69E-15	0.293216924	0.368	0.075	1.24E-10	13	SCAMP2
3.73E-15	0.805619608	0.877	0.524	1.25E-10	13	LAPTM4A
3.86E-15	0.359524312	0.386	0.083	1.29E-10	13	NASP
3.93E-15	0.506115926	0.263	0.044	1.32E-10	13	EXOC1

3.96E-15	0.455740808	0.368	0.079	1.33E-10	13	CD47
4.10E-15	0.297413508	0.351	0.07	1.37E-10	13	CCPG1
4.98E-15	0.325367898	0.263	0.044	1.67E-10	13	GPATCH2L
5.30E-15	0.325762007	0.263	0.044	1.78E-10	13	PRKCB
6.63E-15	0.288398904	0.281	0.049	2.22E-10	13	DNTTIP2
6.76E-15	0.499713978	0.333	0.068	2.27E-10	13	KLF13
6.89E-15	0.354623814	0.281	0.05	2.31E-10	13	GOPC
7.52E-15	0.367378394	0.474	0.114	2.52E-10	13	MBNL1
7.57E-15	0.608956334	0.579	0.175	2.54E-10	13	RBMX
7.89E-15	0.5653266	0.491	0.126	2.65E-10	13	VMP1
1.33E-14	0.370929257	0.281	0.051	4.46E-10	13	PIP4K2A
1.34E-14	0.34276509	0.263	0.045	4.50E-10	13	TRIM33
1.60E-14	0.356960046	0.263	0.046	5.36E-10	13	RNF113A
1.90E-14	0.328275784	0.333	0.067	6.36E-10	13	PARP14
2.11E-14	0.448830671	0.351	0.075	7.06E-10	13	TSNAX
2.25E-14	0.367073404	0.298	0.056	7.53E-10	13	KDM5A
2.38E-14	0.606256587	0.772	0.278	7.97E-10	13	TUBA1B
2.49E-14	0.535894298	0.632	0.192	8.35E-10	13	BEX4
2.50E-14	0.586851332	0.351	0.077	8.38E-10	13	SMARCA5
2.57E-14	0.290599335	0.298	0.056	8.62E-10	13	CDK13
3.23E-14	0.392656491	0.281	0.052	1.08E-09	13	TAOK1
3.55E-14	0.580686039	0.596	0.182	1.19E-09	13	PDIA6
3.56E-14	0.451547642	0.351	0.076	1.19E-09	13	MIDN
3.93E-14	0.302059637	0.281	0.051	1.32E-09	13	NCK1
4.05E-14	0.51413249	0.649	0.204	1.36E-09	13	SRSF2
4.33E-14	0.502033315	0.439	0.109	1.45E-09	13	HES1
4.39E-14	0.406203175	0.281	0.053	1.47E-09	13	PKN1
5.90E-14	0.414916853	0.263	0.047	1.98E-09	13	SAMD9
6.17E-14	0.424542083	0.298	0.058	2.07E-09	13	NUCB2
6.20E-14	0.392225979	0.404	0.094	2.08E-09	13	MPG
6.69E-14	0.344354994	0.316	0.063	2.25E-09	13	SNX29
6.73E-14	0.619779917	0.544	0.157	2.26E-09	13	LITAF
7.32E-14	0.292207987	0.281	0.052	2.46E-09	13	GABPB1-AS1
7.78E-14	0.422024059	0.386	0.088	2.61E-09	13	AKAP12
9.06E-14	0.432659837	0.421	0.102	3.04E-09	13	BCLAF1
9.46E-14	0.305787636	0.298	0.058	3.17E-09	13	SSFA2
9.71E-14	0.341482704	0.386	0.088	3.26E-09	13	RRBP1
1.02E-13	0.511150137	0.281	0.054	3.42E-09	13	COL4A3BP
1.14E-13	0.657523248	0.614	0.205	3.81E-09	13	CAST
1.17E-13	0.479501947	0.263	0.048	3.93E-09	13	PIM1

1.19E-13	0.355333466	0.281	0.053	4.01E-09	13	RHOT2
1.24E-13	0.305784295	0.281	0.053	4.17E-09	13	PTCD3
1.50E-13	0.496345741	0.368	0.086	5.02E-09	13	USF2
1.68E-13	0.447056234	0.368	0.086	5.62E-09	13	RSF1
1.71E-13	0.333990932	0.439	0.109	5.75E-09	13	SRSF4
1.73E-13	0.426887479	0.456	0.119	5.80E-09	13	TLN1
1.84E-13	0.408163981	0.281	0.054	6.18E-09	13	DGKZ
1.92E-13	0.517765687	0.526	0.153	6.45E-09	13	DDX3X
2.08E-13	0.371668214	0.333	0.072	6.99E-09	13	UNC50
2.65E-13	0.342343958	0.421	0.103	8.88E-09	13	DNAJC1
2.76E-13	0.377034938	0.298	0.061	9.25E-09	13	KANSL1
2.93E-13	0.447280982	0.526	0.152	9.84E-09	13	HNRNPH1
2.94E-13	0.28808036	0.316	0.065	9.87E-09	13	STK17B
3.18E-13	0.437872037	0.456	0.121	1.07E-08	13	GRB2
3.20E-13	0.368286326	0.351	0.079	1.07E-08	13	SH3KBP1
3.47E-13	0.467769122	0.263	0.05	1.16E-08	13	NUDT3
3.58E-13	0.338303321	0.298	0.061	1.20E-08	13	RBM26
3.79E-13	0.383617664	0.298	0.061	1.27E-08	13	SMARCA2
4.02E-13	0.434113683	0.298	0.061	1.35E-08	13	SUPT16H
4.07E-13	0.663849417	0.825	0.384	1.36E-08	13	SSR4
4.28E-13	0.416825525	0.263	0.05	1.44E-08	13	VSIR
4.51E-13	0.378121014	0.351	0.08	1.51E-08	13	PABPC4
5.11E-13	0.443791289	0.386	0.095	1.71E-08	13	EIF4A3
5.37E-13	0.705640922	0.947	0.568	1.80E-08	13	HSP90AA1
5.73E-13	0.399535198	0.351	0.08	1.92E-08	13	UPF2
5.76E-13	0.412396541	0.263	0.05	1.93E-08	13	ZMYM2
6.35E-13	0.670206236	0.877	0.489	2.13E-08	13	CLIC1
7.13E-13	0.523318795	0.333	0.078	2.39E-08	13	IRF2BP2
8.25E-13	0.383535949	0.281	0.056	2.77E-08	13	CYLD
9.06E-13	0.487064197	0.456	0.125	3.04E-08	13	SGK1
9.26E-13	0.32837149	0.351	0.08	3.11E-08	13	TMEM9B
9.67E-13	0.455833731	0.263	0.05	3.24E-08	13	SGK3
9.96E-13	0.274819494	0.298	0.061	3.34E-08	13	CAPN1
1.08E-12	0.631128225	1	0.843	3.61E-08	13	MT-CYB
1.31E-12	0.331383814	0.333	0.074	4.41E-08	13	MDM4
1.35E-12	0.314919773	0.316	0.068	4.54E-08	13	NSD1
1.36E-12	0.617038994	1	0.906	4.57E-08	13	MT-CO1
1.39E-12	0.657260788	0.596	0.207	4.68E-08	13	ACTR3
1.43E-12	0.365886643	0.368	0.088	4.80E-08	13	PSMA3-AS1
1.44E-12	0.427507741	0.404	0.104	4.82E-08	13	TAPBP

1.45E-12	0.435521196	0.421	0.112	4.86E-08	13	NCOR1
1.50E-12	0.373854047	0.439	0.115	5.02E-08	13	RALB
1.54E-12	0.333454995	0.298	0.062	5.17E-08	13	MID1IP1
1.61E-12	0.515211319	0.298	0.064	5.40E-08	13	ZBTB7A
1.63E-12	0.312507981	0.281	0.056	5.46E-08	13	TMX1
1.96E-12	0.76871552	0.596	0.223	6.58E-08	13	CALR
2.01E-12	0.488713144	0.368	0.09	6.75E-08	13	PURA
2.12E-12	0.402194969	0.263	0.052	7.10E-08	13	PLPP5
2.22E-12	0.436118115	0.368	0.09	7.46E-08	13	PLGRKT
2.34E-12	0.409295718	0.263	0.052	7.85E-08	13	PPP1R18
2.37E-12	0.312362246	0.351	0.081	7.94E-08	13	KLF2
2.53E-12	0.338475003	0.316	0.069	8.48E-08	13	PHACTR4
2.62E-12	0.353493805	0.316	0.07	8.79E-08	13	STAG2
2.85E-12	0.356997322	0.333	0.077	9.55E-08	13	PIK3R1
2.89E-12	0.446827819	0.982	0.882	9.70E-08	13	RPL30
2.95E-12	0.45371057	0.351	0.086	9.89E-08	13	UBE3A
3.17E-12	0.299716551	0.298	0.063	1.06E-07	13	CNPY3
3.21E-12	0.540802821	0.684	0.256	1.08E-07	13	GNAS
3.25E-12	0.389945479	0.386	0.098	1.09E-07	13	ZRANB2
3.31E-12	0.33666238	0.263	0.052	1.11E-07	13	MYLIP
3.55E-12	0.40231894	0.421	0.112	1.19E-07	13	PSMD13
4.03E-12	0.593011404	0.614	0.207	1.35E-07	13	EIF3D
4.13E-12	0.352554222	0.298	0.064	1.39E-07	13	QKI
4.20E-12	0.454980415	0.561	0.177	1.41E-07	13	DDAH2
4.21E-12	0.276541135	0.561	0.175	1.41E-07	13	HLA-DPB1
5.42E-12	0.350515479	0.333	0.078	1.82E-07	13	ITSN2
6.46E-12	0.387601159	0.386	0.099	2.17E-07	13	DYNC1H1
6.56E-12	0.251354944	0.333	0.076	2.20E-07	13	ATP6AP1
7.09E-12	0.442770418	0.316	0.073	2.38E-07	13	PPP3CA
7.41E-12	0.368215386	0.404	0.105	2.49E-07	13	SELENOS
7.41E-12	0.329773418	0.386	0.098	2.49E-07	13	PPP1R14B
7.53E-12	0.458776429	0.316	0.072	2.53E-07	13	BOD1L1
7.98E-12	0.381082117	0.368	0.091	2.68E-07	13	RNASET2
8.02E-12	0.338275124	0.316	0.072	2.69E-07	13	RNF146
8.06E-12	0.467472091	0.474	0.14	2.70E-07	13	SLTM
8.48E-12	0.396532253	0.404	0.108	2.84E-07	13	SNAP23
8.58E-12	0.283975343	0.351	0.084	2.88E-07	13	DDOST
9.19E-12	0.353210063	0.298	0.066	3.08E-07	13	RBM6
9.56E-12	0.474073909	0.281	0.061	3.21E-07	13	ARHGEF1
1.03E-11	0.477146641	0.667	0.236	3.45E-07	13	SDCBP

1.14E-11	0.307632373	0.263	0.054	3.82E-07	13	WDR82
1.17E-11	0.500440527	0.509	0.16	3.92E-07	13	SCAF11
1.25E-11	0.424509265	0.263	0.055	4.20E-07	13	TMEM245
1.29E-11	0.499093198	0.684	0.246	4.31E-07	13	ATP6V0B
1.33E-11	0.295030297	0.404	0.105	4.47E-07	13	GMFG
1.36E-11	0.295018086	0.263	0.054	4.56E-07	13	TAP1
1.48E-11	0.392382855	0.386	0.101	4.96E-07	13	ORMDL1
1.49E-11	0.262862272	0.368	0.091	5.00E-07	13	WIPF1
1.55E-11	0.316319742	0.351	0.086	5.19E-07	13	HSD17B4
2.07E-11	0.361151408	0.333	0.08	6.94E-07	13	PPP1R12A
2.12E-11	0.549665739	0.316	0.076	7.10E-07	13	MYCBP2
2.14E-11	0.558481747	0.737	0.299	7.16E-07	13	SRSF5
2.24E-11	0.470076757	0.421	0.12	7.50E-07	13	RPL7L1
2.33E-11	0.341768688	0.281	0.061	7.80E-07	13	UBA2
2.45E-11	0.387251906	0.491	0.147	8.22E-07	13	RRAGA
2.48E-11	0.701664917	0.596	0.226	8.32E-07	13	SEPT7
2.61E-11	0.358601468	0.351	0.089	8.75E-07	13	CELF1
2.79E-11	0.35727702	0.509	0.155	9.37E-07	13	CSNK1A1
2.83E-11	0.468963139	0.316	0.075	9.48E-07	13	SMC3
3.05E-11	0.298200613	0.316	0.073	1.02E-06	13	TRAF3IP3
3.08E-11	0.367785096	0.351	0.088	1.03E-06	13	IQGAP2
3.25E-11	0.606186931	0.719	0.302	1.09E-06	13	PPIB
3.65E-11	0.283357817	0.263	0.055	1.23E-06	13	REL
4.68E-11	0.450035661	0.526	0.17	1.57E-06	13	ACTR2
4.90E-11	0.479577133	0.316	0.078	1.64E-06	13	SKIL
4.93E-11	0.403809009	0.421	0.121	1.65E-06	13	TRA2B
5.41E-11	0.463060178	0.316	0.078	1.82E-06	13	CALCOCO2
5.50E-11	0.482349236	0.404	0.115	1.84E-06	13	SDF2L1
5.86E-11	0.328610913	0.263	0.057	1.97E-06	13	RB1CC1
5.89E-11	0.500723212	0.281	0.064	1.98E-06	13	USP34
6.09E-11	0.382099783	0.298	0.07	2.04E-06	13	UBE2G2
6.37E-11	0.562335027	0.719	0.284	2.14E-06	13	HSP90B1
6.41E-11	0.384233813	0.404	0.113	2.15E-06	13	OSTF1
6.71E-11	0.440722967	0.368	0.101	2.25E-06	13	PARP1
7.14E-11	0.464930556	0.316	0.077	2.39E-06	13	ATP1B3
7.49E-11	0.354343184	0.263	0.057	2.51E-06	13	LONP2
7.82E-11	0.450703323	0.474	0.147	2.62E-06	13	HNRNPD
8.49E-11	0.412003849	0.316	0.077	2.85E-06	13	GPSM3
8.78E-11	0.435138703	0.421	0.122	2.95E-06	13	CEBPB
9.09E-11	0.251919755	0.333	0.081	3.05E-06	13	SLBP

9.72E-11	0.29428764	0.702	0.261	3.26E-06	13	MTRNR2L12
9.91E-11	0.356501899	0.491	0.153	3.32E-06	13	ACTN4
1.07E-10	0.533691217	0.456	0.148	3.59E-06	13	SF3B1
1.21E-10	0.439291825	0.544	0.178	4.06E-06	13	UBE2I
1.21E-10	0.325188791	0.386	0.105	4.07E-06	13	NR3C1
1.32E-10	0.306196403	0.298	0.071	4.43E-06	13	COPB2
1.41E-10	0.290716635	0.263	0.057	4.75E-06	13	UBA3
1.51E-10	0.285625829	0.404	0.112	5.05E-06	13	EMC7
1.56E-10	0.416305931	0.667	0.242	5.24E-06	13	RBM39
1.61E-10	0.331948037	0.316	0.078	5.40E-06	13	BRD4
1.62E-10	0.4361027	0.421	0.124	5.42E-06	13	PCMT1
1.65E-10	0.411548987	0.439	0.13	5.54E-06	13	PAK2
1.68E-10	0.295065819	0.404	0.111	5.64E-06	13	PLEKHF2
1.70E-10	0.535208814	0.316	0.082	5.71E-06	13	CHD4
1.73E-10	0.41545305	0.298	0.074	5.80E-06	13	CPNE1
1.80E-10	0.497232973	0.719	0.298	6.05E-06	13	SSR2
1.81E-10	0.283593262	0.333	0.085	6.08E-06	13	YME1L1
1.89E-10	0.492752287	0.474	0.158	6.33E-06	13	SFPQ
2.07E-10	0.253150092	0.281	0.064	6.95E-06	13	TMX2
2.08E-10	0.470725204	0.842	0.472	6.98E-06	13	SH3BGRL3
2.13E-10	0.356813437	0.263	0.059	7.16E-06	13	REST
2.32E-10	0.405591389	0.421	0.126	7.78E-06	13	ATF4
2.34E-10	0.297730035	0.298	0.071	7.85E-06	13	MPP1
2.37E-10	0.283808512	0.263	0.058	7.95E-06	13	AGPS
2.44E-10	0.326699666	0.368	0.101	8.17E-06	13	UPF3A
2.46E-10	0.318736003	0.351	0.093	8.24E-06	13	MOB4
2.50E-10	0.279730158	0.281	0.065	8.38E-06	13	TACC1
2.97E-10	0.348049157	0.333	0.087	9.96E-06	13	PRPF38B
3.40E-10	0.30100214	0.333	0.086	1.14E-05	13	TIAL1
3.41E-10	0.323242102	0.263	0.06	1.14E-05	13	COMMD2
3.42E-10	0.327914036	0.263	0.06	1.15E-05	13	HCLS1
3.54E-10	0.355902357	0.351	0.095	1.19E-05	13	ANKRD11
4.20E-10	0.448034827	0.316	0.083	1.41E-05	13	TNFAIP8
4.61E-10	0.326020748	0.263	0.06	1.55E-05	13	MXD4
4.84E-10	0.302582539	0.456	0.139	1.62E-05	13	LMNA
5.38E-10	0.404019228	0.281	0.068	1.81E-05	13	TRABD
5.58E-10	0.35691508	0.386	0.111	1.87E-05	13	KIF5B
5.66E-10	0.283906511	0.456	0.139	1.90E-05	13	GPBP1
5.75E-10	0.34065624	0.316	0.081	1.93E-05	13	PPP4R3B
5.86E-10	0.443916948	0.544	0.193	1.97E-05	13	SRSF7

5.92E-10	0.322630964	0.333	0.088	1.98E-05	13	OS9
6.64E-10	0.407491465	0.281	0.068	2.23E-05	13	MARCH7
6.64E-10	0.408253981	0.561	0.2	2.23E-05	13	SPINT2
7.03E-10	0.344243416	0.614	0.219	2.36E-05	13	ARF1
7.07E-10	0.352073322	0.333	0.09	2.37E-05	13	COPB1
7.48E-10	0.490236738	0.614	0.239	2.51E-05	13	HERPUD1
9.13E-10	0.294044207	0.316	0.081	3.06E-05	13	TPP1
9.24E-10	0.37417473	0.281	0.069	3.10E-05	13	SETX
9.65E-10	0.334556188	0.333	0.091	3.24E-05	13	TPR
1.01E-09	0.298626222	0.439	0.133	3.40E-05	13	CCT5
1.06E-09	0.494522903	0.982	0.663	3.56E-05	13	MT-ND5
1.11E-09	0.294719011	0.316	0.082	3.71E-05	13	MBP
1.13E-09	0.279527068	0.281	0.068	3.78E-05	13	DR1
1.50E-09	0.319696323	0.316	0.083	5.03E-05	13	THUMPD3-AS1
1.57E-09	0.415257073	0.632	0.239	5.25E-05	13	TAGLN2
1.95E-09	0.312961065	0.263	0.063	6.53E-05	13	PRKDC
2.16E-09	0.26653127	0.316	0.083	7.26E-05	13	ZNF22
2.40E-09	0.307696488	0.316	0.085	8.04E-05	13	ERGIC2
2.71E-09	0.327568457	0.386	0.115	9.09E-05	13	MYDGF
2.73E-09	0.30547065	0.754	0.317	9.15E-05	13	PSAP
2.76E-09	0.363178931	0.509	0.176	9.24E-05	13	ANP32B
2.95E-09	0.352437822	0.281	0.072	9.89E-05	13	EIF2AK2
3.68E-09	0.273567935	0.298	0.078	0.000123306	13	GOLGB1
4.43E-09	0.326034065	0.263	0.065	0.000148678	13	CNOT2
4.59E-09	0.397469616	0.351	0.103	0.000153896	13	ANXA6
4.96E-09	0.278020094	0.316	0.085	0.000166235	13	DNAJB14
5.32E-09	0.252868127	0.333	0.093	0.000178294	13	WDR33
5.34E-09	0.325150128	0.298	0.08	0.000178989	13	MAPK1
5.67E-09	0.429894782	0.404	0.13	0.000190258	13	TNRC6B
5.71E-09	0.271034962	0.263	0.065	0.00019134	13	TXNDC12
5.80E-09	0.494474007	0.456	0.16	0.00019459	13	KLF6
6.08E-09	0.38763845	0.386	0.12	0.000203895	13	LEPROTL1
6.50E-09	0.500381055	0.404	0.136	0.000218072	13	TAF7
6.70E-09	0.25566124	0.298	0.079	0.00022464	13	GNS
6.72E-09	0.326974971	0.509	0.178	0.000225227	13	PHB2
6.80E-09	0.435029887	0.421	0.137	0.000228171	13	PSMB9
6.82E-09	0.253991838	0.316	0.086	0.000228804	13	ERP44
7.16E-09	0.318849678	0.632	0.24	0.000240076	13	LY6E
7.17E-09	0.387726699	0.579	0.223	0.00024035	13	ARPC5
7.69E-09	0.467967963	0.596	0.242	0.000257853	13	HNRNPA3

8.17E-09	0.448309194	0.912	0.488	0.000274119	13	HNRNPA2B1
9.08E-09	0.302292537	0.351	0.104	0.000304676	13	DHX36
1.02E-08	0.442846948	0.807	0.377	0.000343697	13	DAD1
1.03E-08	0.448351651	0.737	0.34	0.00034493	13	SEC11A
1.04E-08	0.329613676	0.386	0.121	0.000348351	13	EPB41L4A-AS1
1.07E-08	0.282102487	0.263	0.067	0.000359115	13	IRF3
1.08E-08	0.263323998	0.263	0.066	0.000363622	13	SEC63
1.17E-08	0.328427019	0.421	0.137	0.0003937	13	PSMD7
1.18E-08	0.361591362	0.263	0.067	0.000395104	13	CD68
1.27E-08	0.352281819	0.298	0.083	0.000425864	13	APH1A
1.42E-08	0.333055995	0.421	0.139	0.000476692	13	CDC37
1.56E-08	0.4436156541	1	0.907	0.000524079	13	MT-CO3
1.60E-08	0.257642654	0.281	0.074	0.000537958	13	TMEM259
1.62E-08	0.320908617	0.421	0.139	0.000543335	13	TGOLN2
1.62E-08	0.33609154	0.281	0.076	0.000544148	13	CUL3
1.64E-08	0.313246076	0.368	0.115	0.000551035	13	ZNF207
1.70E-08	0.271239676	0.368	0.112	0.000569021	13	PDCD2
1.82E-08	0.447180729	0.404	0.132	0.000612045	13	PRPF4B
2.08E-08	0.294061193	0.281	0.076	0.000696587	13	ZFR
2.17E-08	0.381280184	0.842	0.379	0.000726105	13	IFITM3
2.48E-08	0.357067425	0.491	0.178	0.000830623	13	SRSF11
3.02E-08	0.341173664	0.421	0.141	0.001011304	13	DDX46
3.11E-08	0.347548286	0.561	0.218	0.001043375	13	CAP1
3.18E-08	0.268387535	0.351	0.107	0.00106584	13	U2SURP
3.59E-08	0.252173867	0.333	0.099	0.001203584	13	PRPF6
3.89E-08	0.28717653	0.281	0.078	0.001305486	13	DBNL
4.08E-08	0.496944365	0.509	0.198	0.00137002	13	ANXA11
4.11E-08	0.383556078	0.667	0.286	0.001379076	13	ALDH1A1
4.69E-08	0.361459368	0.298	0.088	0.001571795	13	ASH1L
4.80E-08	0.312176527	0.439	0.151	0.001608534	13	TYMP
4.86E-08	0.313120121	0.702	0.297	0.00163007	13	SON
5.41E-08	0.258921696	0.281	0.077	0.001814752	13	CPPED1
6.58E-08	0.524097345	0.807	0.527	0.00220598	13	HSP90AB1
6.63E-08	0.328334085	0.298	0.088	0.002223755	13	SMAP1
7.23E-08	0.328330775	0.316	0.096	0.002424301	13	PABPN1
7.63E-08	0.253404727	0.316	0.094	0.002559113	13	CCNDBP1
8.18E-08	0.43343533	0.386	0.132	0.002744567	13	CLNS1A
8.53E-08	0.325430087	0.386	0.128	0.002861801	13	HNRNPH3
8.63E-08	0.355860367	0.298	0.09	0.002894207	13	ADD1
9.31E-08	0.378724598	0.316	0.098	0.00312197	13	SEC31A

9.49E-08	0.279382559	0.474	0.17	0.003184328	13	TMEM50A
1.15E-07	0.406268014	0.333	0.108	0.003853888	13	TUBA1A
1.17E-07	0.362725972	0.386	0.136	0.003915576	13	SRSF10
1.27E-07	0.321862807	0.316	0.098	0.004270324	13	PDCD6IP
1.35E-07	0.278087181	0.404	0.141	0.004521911	13	SNRNP70
1.40E-07	0.325724835	0.263	0.074	0.00468221	13	AZIN1
1.53E-07	0.345255279	0.351	0.115	0.005131004	13	RPN1
1.61E-07	0.310434776	0.526	0.206	0.005400814	13	STMN1
1.67E-07	0.358098433	0.386	0.134	0.005599384	13	TERF2IP
1.72E-07	0.327125323	0.456	0.17	0.005779109	13	TMED10
1.86E-07	0.303590498	0.544	0.219	0.006247596	13	RBM3
1.99E-07	0.434122852	0.263	0.075	0.00668166	13	USP22
2.07E-07	0.382369934	0.789	0.392	0.006933878	13	ARPC2
2.22E-07	0.265576889	0.474	0.172	0.007448245	13	SFT2D1
2.29E-07	0.251141192	0.298	0.09	0.007679559	13	LACTB
2.45E-07	0.369097753	0.632	0.286	0.008218156	13	EIF5
2.59E-07	0.355870111	0.561	0.233	0.00868333	13	ZFP36L1
2.73E-07	0.290971316	0.298	0.091	0.009163978	13	RCN2
2.78E-07	0.27894155	0.263	0.076	0.009320285	13	ZC3HAV1
2.92E-07	0.376191176	0.263	0.078	0.00980453	13	ANKRD10
2.97E-07	0.336455146	0.544	0.217	0.009959646	13	SERPINB6
3.26E-07	0.363026699	0.281	0.084	0.010946299	13	ARID1B
3.32E-07	0.296506319	0.544	0.22	0.011147179	13	SRI
3.45E-07	0.38367115	1	0.873	0.011557416	13	MT-ND4
3.90E-07	0.262849433	0.333	0.109	0.013070348	13	LASP1
6.37E-07	0.377327146	0.596	0.259	0.021356468	13	TMBIM4
7.04E-07	0.273546488	0.281	0.087	0.02361041	13	MKRN1
9.35E-07	0.266211125	0.333	0.112	0.031353582	13	GGNBP2
9.44E-07	0.30126347	0.404	0.151	0.031656299	13	TAF1D
9.89E-07	0.34097571	0.702	0.329	0.033176701	13	SERP1
1.06E-06	0.373471279	0.368	0.136	0.03559454	13	MAP2K2
1.06E-06	0.321855611	0.368	0.135	0.035680158	13	MCL1
1.09E-06	0.349058234	0.86	0.416	0.036512897	13	MT-ND4L
1.25E-06	0.256271981	0.263	0.08	0.04204473	13	SDF2
1.50E-06	0.324014228	0.263	0.082	0.050250329	13	CTDSP1
2.15E-06	0.285431457	0.86	0.415	0.072006468	13	PABPC1
2.29E-06	0.317628571	0.333	0.119	0.076942174	13	RPN2
2.34E-06	0.287332238	0.368	0.137	0.078427779	13	MTPN
2.38E-06	0.313456855	0.333	0.119	0.079938294	13	XRN2
2.57E-06	0.335364828	0.281	0.091	0.086255539	13	TLK1

3.42E-06	0.287709434	0.386	0.15	0.114651593	13	SRP19
3.44E-06	0.307254095	0.561	0.26	0.115412976	13	SH3BGRL
3.46E-06	0.272034443	0.526	0.224	0.116142467	13	PLIN2
3.79E-06	0.413859159	0.404	0.154	0.126994401	13	ATP6AP2
4.14E-06	0.353890977	0.263	0.086	0.138815089	13	CUX1
4.89E-06	0.265519783	0.439	0.178	0.163968536	13	CCT6A
7.35E-06	0.262460451	0.456	0.186	0.246531468	13	ARPC1B
7.39E-06	0.341000967	0.596	0.279	0.24799431	13	HNRNPF
7.56E-06	0.37244278	0.86	0.52	0.253687149	13	CIRBP
7.57E-06	0.324206401	0.895	0.627	0.253802081	13	DYNLL1
8.48E-06	0.267001554	0.281	0.096	0.28432854	13	CASP4
8.77E-06	0.307078708	0.439	0.185	0.294065554	13	NME3
1.07E-05	0.295186372	0.649	0.314	0.360258865	13	SRSF3
1.37E-05	0.280953493	0.789	0.395	0.460678084	13	NOP53
1.46E-05	0.27934272	0.281	0.097	0.488383136	13	YIPF3
2.59E-05	0.329077579	0.298	0.113	0.867012189	13	GNB1
2.86E-05	0.298036444	0.351	0.14	0.958879595	13	CAPZA1
4.56E-05	0.33267898	0.93	0.648	1	13	UBC
6.91E-05	0.263641184	0.93	0.565	1	13	MYL12A
6.93E-05	0.272022916	0.947	0.693	1	13	RPL36AL
0.000111906	0.282412908	0.368	0.159	1	13	NOSIP
0.000160047	0.268226265	0.316	0.135	1	13	HNRNPR
0.000170016	0.333129829	0.474	0.229	1	13	ZBTB20
0.000214929	0.264825726	0.421	0.193	1	13	REEP5
0.00027394	0.251769882	0.614	0.336	1	13	HMGN2
0.000663468	0.272250168	0.333	0.156	1	13	MAT2A
0.000882707	0.266507085	0.421	0.221	1	13	ATRAID
0	4.601228638	0.84	0.01	0	14	PODXL
0	4.006384575	0.86	0.007	0	14	PTGDS
0	3.638669569	0.86	0.005	0	14	PLA2R1
0	3.566504472	0.84	0	0	14	NPHS2
0	3.480710658	0.86	0.005	0	14	CLIC5
0	3.365723473	0.84	0.005	0	14	DCN
0	3.276788871	0.84	0.009	0	14	NPNT
0	3.255988324	0.68	0.007	0	14	CTGF
0	3.030711294	0.72	0.004	0	14	ALS2CL
0	2.858849587	0.8	0.001	0	14	FGF1
0	2.853285383	0.8	0.002	0	14	PTPRO
0	2.814389473	0.8	0.011	0	14	IGFBP2
0	2.722929134	0.8	0.003	0	14	TCF21

0	2.717403457	0.7	0.012	0	14	SYNPO
0	2.681574907	0.8	0	0	14	PCOLCE2
0	2.639177761	0.72	0.007	0	14	NES
0	2.600050428	0.84	0.012	0	14	FOXC1
0	2.528494487	0.84	0.001	0	14	NPHS1
0	2.449141672	0.68	0	0	14	DDN
0	2.420034052	0.74	0.016	0	14	THSD7A
0	2.413571055	0.72	0.009	0	14	COL4A3
0	2.342492456	0.76	0.005	0	14	ARMH4
0	2.170124675	0.66	0.004	0	14	SPOCK1
0	2.124205521	0.68	0.01	0	14	DACH1
0	2.108131871	0.74	0.017	0	14	MAFB
0	2.084075535	0.74	0.013	0	14	ITGA3
0	2.05904144	0.66	0.001	0	14	F3
0	2.002103994	0.64	0	0	14	NDNF
0	1.999972378	0.68	0.002	0	14	WT1
0	1.98181784	0.66	0.008	0	14	ITIH5
0	1.966978607	0.64	0.001	0	14	CRB2
0	1.966646725	0.66	0.003	0	14	TYRO3
0	1.961099542	0.64	0.008	0	14	PLCE1
0	1.939432725	0.62	0.001	0	14	NTNG1
0	1.931925177	0.58	0	0	14	AC092813.2
0	1.912006067	0.58	0.001	0	14	CPXM1
0	1.873612225	0.6	0.002	0	14	ST6GALNAC3
0	1.865796848	0.66	0.007	0	14	TMEM150C
0	1.808916849	0.56	0.004	0	14	TPPP3
0	1.749106011	0.5	0.007	0	14	FRY
0	1.740878814	0.62	0.002	0	14	TNNT2
0	1.740785001	0.52	0.006	0	14	NFASC
0	1.738820475	0.54	0	0	14	DPP6
0	1.728750262	0.58	0.009	0	14	CR1
0	1.705216069	0.54	0.003	0	14	KIRREL1
0	1.658354719	0.54	0.001	0	14	SULF1
0	1.59324607	0.54	0.004	0	14	MAGI2
0	1.564959789	0.46	0	0	14	PTPRQ
0	1.525113739	0.46	0.002	0	14	MRGPRF
0	1.502605729	0.5	0.003	0	14	MXRA8
0	1.447930862	0.4	0.001	0	14	LOX
0	1.429575852	0.4	0.003	0	14	LRRC2
0	1.42072022	0.46	0.001	0	14	APOD

0	1.347304004	0.42	0.003	0	14	CLDN5
0	1.294274125	0.4	0	0	14	KLK7
0	1.248339223	0.26	0	0	14	CHI3L1
0	1.222392475	0.44	0	0	14	TNNI1
0	1.215971618	0.36	0.002	0	14	APLN
0	1.204277558	0.34	0.001	0	14	SEMA3G
0	1.19475432	0.26	0.001	0	14	CCBE1
0	1.176699518	0.42	0.004	0	14	TSPAN2
0	1.143226552	0.4	0.002	0	14	MAP6
0	1.136092175	0.4	0.003	0	14	BMP7
0	1.122366067	0.3	0.002	0	14	GJA1
0	1.107869166	0.34	0	0	14	LINC00839
0	1.092806164	0.36	0	0	14	TENM2
0	1.007593219	0.28	0.001	0	14	CFAP45
0	1.006698491	0.34	0.001	0	14	VSIG8
0	0.999101031	0.3	0.002	0	14	GPSM1
0	0.992833858	0.28	0.001	0	14	KLK6
0	0.977713458	0.36	0	0	14	LMX1B
0	0.94878172	0.28	0.002	0	14	FOXC2
0	0.928892905	0.26	0.001	0	14	AC008264.2
0	0.913657086	0.26	0	0	14	TMEM200C
0	0.855535835	0.26	0.001	0	14	MSLN
0	0.804283531	0.26	0	0	14	AC023154.1
0	0.791105999	0.3	0.002	0	14	ARHGEF26
5.52E-306	2.511832153	0.72	0.017	1.85E-301	14	SEMA5A
3.23E-301	1.666349958	0.58	0.011	1.08E-296	14	SRGAP2C
1.08E-291	1.09489201	0.34	0.004	3.62E-287	14	TMEM178A
1.35E-291	1.996030792	0.62	0.013	4.54E-287	14	NEBL
3.19E-279	1.209270129	0.42	0.006	1.07E-274	14	PDGFRB
3.42E-276	1.348334051	0.48	0.008	1.15E-271	14	PARD3B
1.32E-269	3.70848848	0.84	0.028	4.43E-265	14	HTRA1
4.15E-265	1.21083531	0.36	0.005	1.39E-260	14	GAS1
1.92E-261	3.048636245	0.88	0.031	6.43E-257	14	SPOCK2
5.46E-261	2.52191528	0.76	0.023	1.83E-256	14	UACA
1.01E-260	1.197556556	0.42	0.006	3.40E-256	14	MAP1B
6.12E-257	1.344957604	0.38	0.005	2.05E-252	14	ARHGAP28
6.66E-255	2.024532323	0.68	0.019	2.23E-250	14	RIPOR1
5.53E-252	0.883636513	0.34	0.004	1.86E-247	14	PRKAR2B
2.50E-242	1.796024078	0.62	0.016	8.39E-238	14	DAG1
7.38E-240	3.061810896	0.84	0.031	2.48E-235	14	VEGFA

2.80E-239	1.741394134	0.62	0.016	9.38E-235	14	VASN
1.31E-230	1.586126726	0.52	0.012	4.41E-226	14	SRGAP2
5.65E-230	2.192296955	0.8	0.029	1.90E-225	14	DST
5.01E-223	1.248687145	0.48	0.01	1.68E-218	14	MYO1E
6.22E-221	1.670408659	0.62	0.018	2.09E-216	14	F2R
2.74E-214	1.004618318	0.28	0.003	9.20E-210	14	PHYHIP
4.43E-214	2.318945955	0.78	0.03	1.49E-209	14	CDC42EP3
2.82E-211	1.523576998	0.46	0.01	9.46E-207	14	NECAB1
8.45E-209	0.985543088	0.28	0.004	2.83E-204	14	ATP10A
3.95E-207	1.416806861	0.42	0.008	1.33E-202	14	ARRDC4
2.45E-206	0.918892511	0.28	0.004	8.21E-202	14	ARHGAP23
5.73E-202	1.024018294	0.3	0.004	1.92E-197	14	MSRB3
6.29E-200	1.283479191	0.42	0.009	2.11E-195	14	MYO1D
3.61E-193	1.757239229	0.64	0.022	1.21E-188	14	SORT1
4.22E-192	1.002337804	0.34	0.006	1.42E-187	14	GPNMB
3.56E-191	1.050679939	0.34	0.006	1.19E-186	14	NDN
3.20E-183	2.30376922	0.72	0.03	1.07E-178	14	TGFBR3
1.22E-176	2.254763252	0.8	0.038	4.11E-172	14	TJP1
4.36E-176	1.40640271	0.5	0.014	1.46E-171	14	EFNB1
5.56E-175	1.631309848	0.54	0.017	1.87E-170	14	ITGB5
1.22E-174	0.813462063	0.26	0.004	4.08E-170	14	ADORA1
6.45E-166	1.231481687	0.36	0.008	2.16E-161	14	BGN
4.58E-162	0.895437098	0.32	0.006	1.53E-157	14	WIPF3
1.28E-157	0.956216703	0.38	0.009	4.29E-153	14	LIMS2
1.02E-154	1.27790669	0.4	0.01	3.43E-150	14	SRGAP2B
9.95E-152	1.022146187	0.38	0.009	3.34E-147	14	NPR1
4.26E-151	1.252763981	0.44	0.013	1.43E-146	14	TNS2
4.04E-150	1.384712831	0.48	0.016	1.35E-145	14	PMEPA1
4.34E-145	2.185586182	0.78	0.044	1.45E-140	14	GOLIM4
4.86E-145	1.253097356	0.46	0.015	1.63E-140	14	APOL1
1.90E-141	1.172789509	0.4	0.011	6.36E-137	14	CDC14A
4.76E-141	1.807843024	0.7	0.036	1.60E-136	14	FAT1
8.44E-140	1.249814136	0.44	0.014	2.83E-135	14	PTGER4
1.79E-138	1.469956706	0.44	0.014	6.01E-134	14	COL4A4
7.65E-138	1.489271761	0.44	0.014	2.56E-133	14	ST3GAL6
3.71E-134	1.143262211	0.44	0.014	1.24E-129	14	TRIB2
5.18E-133	1.92554823	0.74	0.043	1.74E-128	14	SEMA3B
1.58E-132	1.441147504	0.5	0.019	5.29E-128	14	KLHL42
7.15E-131	1.111757156	0.32	0.008	2.40E-126	14	AMIGO2
2.21E-129	2.661917609	0.82	0.057	7.42E-125	14	CRIM1

5.19E-128	0.835528279	0.26	0.005	1.74E-123	14	SMAD6
9.63E-127	1.462870238	0.46	0.017	3.23E-122	14	MGAT5
1.11E-126	1.565252863	0.62	0.031	3.74E-122	14	MAL2
2.32E-126	2.222233674	0.74	0.046	7.76E-122	14	ITGAV
9.10E-124	1.726055259	0.6	0.03	3.05E-119	14	CTDSPL
4.41E-121	1.291365043	0.38	0.012	1.48E-116	14	TPM2
3.55E-120	2.38806387	0.82	0.06	1.19E-115	14	SPARC
7.63E-120	1.199326869	0.3	0.007	2.56E-115	14	MYO1B
4.26E-118	1.342882602	0.56	0.027	1.43E-113	14	ARHGEF3
6.43E-116	2.111923713	0.72	0.045	2.16E-111	14	AIF1
1.29E-113	1.745566833	0.66	0.039	4.32E-109	14	TIMP3
2.19E-113	1.118733358	0.4	0.014	7.34E-109	14	C1R
1.61E-112	0.814248261	0.26	0.006	5.39E-108	14	PTPN21
2.87E-112	2.146349504	0.74	0.052	9.62E-108	14	MYL9
3.58E-111	1.767812178	0.62	0.036	1.20E-106	14	SLK
1.86E-108	1.538938377	0.58	0.031	6.23E-104	14	MYLK
1.73E-106	1.394067531	0.46	0.02	5.82E-102	14	CMYA5
6.78E-106	1.280539186	0.44	0.018	2.27E-101	14	INF2
1.17E-105	1.118447337	0.4	0.015	3.92E-101	14	ADAMTS9
2.72E-103	1.193927541	0.46	0.021	9.12E-99	14	KANK1
9.34E-103	1.285182049	0.38	0.014	3.13E-98	14	ZNRF3
8.78E-102	1.196621865	0.38	0.014	2.94E-97	14	FSTL1
6.75E-99	1.417411657	0.52	0.028	2.26E-94	14	MIB1
8.27E-99	0.853808774	0.3	0.009	2.77E-94	14	FXYD1
1.42E-97	0.789983044	0.28	0.008	4.76E-93	14	CXADR
4.25E-96	1.862501296	0.62	0.041	1.42E-91	14	MPP5
7.45E-96	2.060905773	0.78	0.068	2.50E-91	14	BST2
2.54E-95	2.974185163	0.82	0.078	8.51E-91	14	CDKN1C
3.02E-91	1.0859252	0.3	0.01	1.01E-86	14	FAM89A
9.03E-91	1.077268751	0.36	0.014	3.03E-86	14	FAM20B
4.08E-90	1.583996805	0.58	0.038	1.37E-85	14	RNF145
2.47E-89	1.314664048	0.44	0.022	8.29E-85	14	SIRPA
9.36E-89	1.141415071	0.52	0.03	3.14E-84	14	SLC9A3R2
1.17E-87	2.216663963	0.86	0.094	3.94E-83	14	MYH9
3.21E-87	0.701249262	0.28	0.009	1.08E-82	14	PRICKLE1
1.43E-86	1.396502445	0.44	0.023	4.80E-82	14	TIMP2
2.16E-86	1.92077935	0.78	0.074	7.26E-82	14	AHNAK
4.44E-86	0.928006313	0.32	0.012	1.49E-81	14	TANC1
7.89E-86	1.356423222	0.5	0.03	2.65E-81	14	TNS3
4.41E-85	1.150609405	0.4	0.019	1.48E-80	14	PLTP

5.61E-85	1.16656757	0.44	0.023	1.88E-80	14	TMCO3
1.83E-82	1.541282655	0.54	0.036	6.15E-78	14	PALLD
1.90E-82	1.504064092	0.58	0.042	6.37E-78	14	PDE4DIP
1.67E-81	0.895115245	0.38	0.018	5.59E-77	14	FRMD3
4.77E-81	1.539655234	0.54	0.037	1.60E-76	14	EPB41L5
5.18E-81	1.871705529	0.62	0.05	1.74E-76	14	KCNQ1OT1
4.77E-79	1.638961834	0.58	0.043	1.60E-74	14	LAMB2
1.04E-77	3.144135906	0.84	0.107	3.49E-73	14	MME
2.71E-76	1.213859841	0.46	0.028	9.08E-72	14	ORMDL3
6.22E-75	1.757209596	0.64	0.057	2.08E-70	14	ZDHHC6
1.31E-74	0.817945372	0.26	0.009	4.38E-70	14	MFSD6
4.51E-74	0.948019085	0.26	0.009	1.51E-69	14	RAMP3
6.97E-74	1.033406858	0.32	0.014	2.34E-69	14	TMCC3
2.50E-73	0.886776424	0.3	0.012	8.39E-69	14	C1S
3.95E-73	1.638310408	0.68	0.064	1.32E-68	14	CAPN2
1.92E-70	2.324260323	0.84	0.111	6.42E-66	14	CD81
3.44E-70	1.736685485	0.58	0.049	1.15E-65	14	GMDS
7.61E-70	1.006865941	0.32	0.015	2.55E-65	14	CLIP4
1.08E-69	1.016581152	0.38	0.021	3.63E-65	14	TOM1L2
1.11E-69	0.94753624	0.32	0.015	3.73E-65	14	SERINC5
9.82E-69	1.230612732	0.4	0.023	3.29E-64	14	USP46
1.88E-68	1.113210055	0.4	0.023	6.30E-64	14	SRGAP1
2.78E-67	1.015102547	0.36	0.019	9.34E-63	14	GAS6
6.11E-67	1.135370514	0.38	0.022	2.05E-62	14	PTPN14
3.69E-66	1.956459292	0.74	0.087	1.24E-61	14	IQGAP2
3.80E-66	1.482739192	0.58	0.051	1.27E-61	14	PLCG2
1.28E-63	0.992051991	0.34	0.018	4.31E-59	14	PTPN13
3.82E-62	2.524722688	0.88	0.149	1.28E-57	14	BCAM
5.04E-62	0.794602713	0.3	0.014	1.69E-57	14	VWA1
5.09E-62	1.654466843	0.7	0.081	1.71E-57	14	ITGB8
1.52E-61	1.281092945	0.5	0.041	5.11E-57	14	SEL1L
2.86E-61	1.170224506	0.46	0.034	9.59E-57	14	MYO1C
6.14E-61	1.074697111	0.4	0.026	2.06E-56	14	AGRN
8.59E-60	0.747948105	0.26	0.011	2.88E-55	14	LRP11
1.68E-59	2.116425004	0.86	0.137	5.63E-55	14	ITGB1
4.00E-59	0.832366929	0.26	0.011	1.34E-54	14	APBB2
4.30E-58	1.131078378	0.36	0.023	1.44E-53	14	NOTCH2
4.45E-57	1.205296124	0.52	0.047	1.49E-52	14	MTSS1
3.51E-55	1.374690326	0.58	0.061	1.18E-50	14	SYNE1
2.39E-53	1.008608314	0.38	0.027	8.03E-49	14	TUSC3

4.61E-53	0.958179027	0.34	0.022	1.55E-48	14	PTPRF
5.36E-53	1.733322819	0.76	0.113	1.80E-48	14	ANXA2
1.89E-52	1.425253926	0.6	0.069	6.33E-48	14	PHACTR4
2.17E-52	1.18766601	0.44	0.036	7.29E-48	14	RERG
2.43E-52	1.09345771	0.34	0.022	8.16E-48	14	FRMD1
2.71E-52	1.103277546	0.4	0.03	9.08E-48	14	ZC3H7B
7.93E-52	2.227358982	0.82	0.153	2.66E-47	14	ACTN4
1.83E-51	1.037501521	0.3	0.018	6.15E-47	14	EFEMP1
4.71E-51	1.61456608	0.64	0.082	1.58E-46	14	SERINC1
6.24E-51	1.322340406	0.56	0.061	2.09E-46	14	NFE2L1
1.58E-50	0.926556682	0.28	0.016	5.31E-46	14	CERS6
8.00E-50	1.086114806	0.34	0.023	2.68E-45	14	LAMA5
1.23E-49	0.819263338	0.26	0.014	4.13E-45	14	GALC
2.32E-49	1.91363179	0.74	0.117	7.77E-45	14	HPGD
2.41E-49	1.249496533	0.52	0.054	8.10E-45	14	TMEM245
3.89E-49	1.017190542	0.4	0.032	1.30E-44	14	LGALS8
2.81E-48	1.215875373	0.6	0.072	9.44E-44	14	RBMS1
1.22E-47	1.179654975	0.42	0.037	4.10E-43	14	NECTIN2
1.00E-46	1.388083804	0.62	0.082	3.36E-42	14	MACF1
3.05E-46	1.111183014	0.42	0.037	1.02E-41	14	PEA15
3.95E-46	1.311066095	0.6	0.077	1.32E-41	14	GOLGB1
1.96E-45	1.036680771	0.4	0.035	6.56E-41	14	CBX7
2.33E-45	1.118066057	0.32	0.023	7.81E-41	14	PIEZO1
8.99E-45	1.549345773	0.68	0.105	3.01E-40	14	NDRG1
2.88E-44	1.16047739	0.54	0.064	9.66E-40	14	SDC2
1.12E-43	0.9612762	0.34	0.026	3.74E-39	14	SPATA13
1.48E-43	0.925666457	0.32	0.023	4.98E-39	14	CBLB
2.57E-43	1.213178426	0.48	0.052	8.63E-39	14	FAM20C
3.65E-43	0.84823411	0.26	0.016	1.22E-38	14	MEGF9
7.44E-43	0.91955276	0.36	0.03	2.50E-38	14	PHF10
6.70E-42	1.220090102	0.4	0.038	2.25E-37	14	PLS3
7.43E-42	0.876031197	0.28	0.019	2.49E-37	14	FHL1
7.89E-42	2.46255125	0.94	0.262	2.65E-37	14	S100A6
1.34E-41	0.763781717	0.26	0.016	4.49E-37	14	KLF7
2.08E-41	1.07778105	0.36	0.031	6.97E-37	14	P3H2
2.42E-41	1.379822558	0.6	0.085	8.13E-37	14	SEPT11
3.78E-41	1.035958072	0.36	0.031	1.27E-36	14	PTPRD
2.63E-40	0.521183154	0.26	0.016	8.81E-36	14	CAVIN1
3.18E-40	1.629918839	0.76	0.152	1.07E-35	14	ENPEP
5.56E-40	1.390292256	0.64	0.102	1.87E-35	14	WASL

2.02E-39	1.193752495	0.5	0.062	6.79E-35	14	SPTAN1
4.04E-39	1.169520489	0.46	0.052	1.35E-34	14	PLXNB2
9.83E-39	0.740752372	0.26	0.017	3.30E-34	14	ARHGAP21
1.09E-38	1.366594374	0.5	0.063	3.66E-34	14	UTRN
1.79E-38	1.435807451	0.6	0.095	6.00E-34	14	ARHGEF12
4.42E-38	1.403156836	0.64	0.105	1.48E-33	14	NORAD
1.75E-37	1.251450538	0.6	0.093	5.88E-33	14	PDLIM5
1.78E-37	1.015948531	0.42	0.045	5.97E-33	14	CSRP1
5.09E-37	1.104826273	0.54	0.074	1.71E-32	14	MAP4
5.26E-37	1.051420274	0.36	0.034	1.77E-32	14	CHD3
1.08E-36	1.166184202	0.44	0.052	3.62E-32	14	MYLIP
1.52E-36	1.302603272	0.9	0.212	5.11E-32	14	NEAT1
1.24E-35	1.050408574	0.44	0.051	4.15E-31	14	CLSTN1
1.61E-35	0.924015448	0.32	0.028	5.40E-31	14	SULF2
2.21E-35	0.791760042	0.28	0.022	7.40E-31	14	NLRP1
4.67E-35	0.92281478	0.38	0.04	1.57E-30	14	NPTN
8.73E-35	1.650785424	0.88	0.241	2.93E-30	14	VIM
1.17E-34	1.098833651	0.5	0.068	3.92E-30	14	GSN
2.56E-34	0.880787632	0.26	0.02	8.57E-30	14	HPS5
3.03E-34	1.167578604	0.48	0.064	1.02E-29	14	LGALS3BP
1.91E-33	1.269364827	0.58	0.095	6.40E-29	14	ARHGAP29
2.68E-33	1.182750684	0.58	0.095	8.99E-29	14	STAT1
3.12E-32	0.759998989	0.26	0.021	1.05E-27	14	VTI1A
5.23E-32	0.689656614	0.28	0.024	1.75E-27	14	PRMT6
1.79E-31	1.375807565	0.4	0.05	6.02E-27	14	RNF220
3.35E-31	1.319657091	0.58	0.104	1.12E-26	14	CD46
4.70E-31	1.12501404	0.56	0.095	1.57E-26	14	PABPN1
6.97E-31	1.438909056	0.86	0.232	2.34E-26	14	HLA-E
7.40E-31	1.052098373	0.34	0.036	2.48E-26	14	TEAD1
6.78E-30	0.834592603	0.4	0.05	2.27E-25	14	RAPGEF3
1.64E-29	0.667196705	0.26	0.022	5.50E-25	14	EVA1B
2.34E-29	0.740754935	0.4	0.05	7.85E-25	14	GULP1
1.80E-28	0.948742206	0.3	0.031	6.04E-24	14	MSL1
1.82E-28	1.010719977	0.62	0.121	6.09E-24	14	ANKRD12
2.56E-28	0.989579217	0.44	0.064	8.60E-24	14	QKI
2.76E-28	0.950470006	0.44	0.063	9.25E-24	14	PAK1
2.79E-28	1.790462862	0.9	0.392	9.37E-24	14	IGFBP7
3.14E-28	0.968386186	0.42	0.059	1.05E-23	14	CCDC91
3.25E-28	1.004286287	0.4	0.053	1.09E-23	14	PAM
3.72E-28	0.901623954	0.34	0.039	1.25E-23	14	TMEM98

4.41E-28	1.262067121	0.66	0.147	1.48E-23	14	PDLIM2
7.09E-28	1.661173757	0.92	0.398	2.38E-23	14	MALAT1
7.59E-28	0.980644818	0.34	0.04	2.54E-23	14	MUM1
8.27E-28	1.203751767	0.66	0.141	2.77E-23	14	SYNE2
1.19E-27	0.88060336	0.3	0.032	4.00E-23	14	PIGS
1.23E-27	0.919487945	0.36	0.045	4.14E-23	14	AKT2
1.25E-27	1.62927185	0.94	0.391	4.20E-23	14	HLA-A
1.53E-27	1.11764992	0.48	0.079	5.12E-23	14	CD47
1.76E-27	0.846548226	0.28	0.028	5.89E-23	14	BCAR3
2.09E-27	0.927926681	0.42	0.06	7.02E-23	14	SERINC3
2.28E-27	0.776446242	0.26	0.024	7.65E-23	14	CHD7
3.03E-27	0.617906652	0.26	0.024	1.02E-22	14	GATD1
4.82E-27	0.64235886	0.26	0.024	1.62E-22	14	TCAF1
6.33E-27	0.817417515	0.28	0.028	2.12E-22	14	RASSF8
7.28E-27	0.967952675	0.4	0.056	2.44E-22	14	TMX4
7.78E-27	1.657991068	0.9	0.33	2.61E-22	14	HLA-B
8.17E-27	0.666707954	0.3	0.032	2.74E-22	14	KBTBD11
8.82E-27	0.88825808	0.42	0.061	2.96E-22	14	PLD3
9.03E-27	0.771488437	0.28	0.028	3.03E-22	14	EFEMP2
1.24E-26	0.767987641	0.26	0.025	4.18E-22	14	CDK6
1.38E-26	0.935516251	0.28	0.029	4.64E-22	14	BMPR2
3.96E-26	0.920539285	0.4	0.057	1.33E-21	14	FTX
4.07E-26	0.834936104	0.36	0.046	1.37E-21	14	TUBB2A
5.52E-26	0.932372511	0.38	0.052	1.85E-21	14	TLE4
5.94E-26	0.741873933	0.28	0.029	1.99E-21	14	HYOU1
1.13E-25	1.170280523	0.78	0.201	3.78E-21	14	RARRES2
1.56E-25	0.78693844	0.3	0.034	5.23E-21	14	HSPA12A
2.53E-25	1.08066324	0.4	0.059	8.49E-21	14	NDRG2
4.17E-25	1.459751932	0.92	0.355	1.40E-20	14	HLA-C
4.20E-25	1.685106624	1	0.795	1.41E-20	14	ITM2B
8.52E-25	0.643300289	0.26	0.026	2.86E-20	14	RSPH3
9.25E-25	0.728712691	0.26	0.026	3.10E-20	14	LGR4
2.21E-24	0.88305948	0.4	0.061	7.42E-20	14	SMARCA2
2.66E-24	1.588737714	0.82	0.317	8.92E-20	14	PSAP
3.04E-24	1.135256377	0.36	0.052	1.02E-19	14	CGNL1
5.23E-24	0.76892698	0.28	0.031	1.75E-19	14	NEK7
8.44E-24	1.065992628	0.46	0.082	2.83E-19	14	NFIC
9.81E-24	0.961255264	0.42	0.068	3.29E-19	14	RERE
1.43E-23	0.846340178	0.44	0.074	4.81E-19	14	FNBP1
1.83E-23	0.73398026	0.34	0.045	6.12E-19	14	PARVA

2.06E-23	0.770115708	0.28	0.032	6.90E-19	14	VCL
4.05E-23	1.413160357	0.58	0.14	1.36E-18	14	CD151
4.18E-23	0.963856117	0.32	0.042	1.40E-18	14	LTBP3
4.86E-23	1.170935849	0.78	0.243	1.63E-18	14	APLP2
6.27E-23	0.853096289	0.46	0.081	2.10E-18	14	PCYOX1
7.21E-23	1.136572761	0.36	0.054	2.42E-18	14	FLRT3
1.07E-22	0.749189242	0.34	0.047	3.60E-18	14	ATXN2
1.56E-22	0.837597316	0.38	0.058	5.24E-18	14	TMEM30A
1.94E-22	0.809277517	0.28	0.033	6.51E-18	14	ALCAM
3.42E-22	1.31388145	0.62	0.16	1.15E-17	14	HSPA5
3.78E-22	1.32179217	0.8	0.284	1.27E-17	14	HSP90B1
4.86E-22	0.806795016	0.28	0.034	1.63E-17	14	AHI1
5.20E-22	0.949800692	0.36	0.054	1.74E-17	14	FERMT2
8.21E-22	0.800266799	0.3	0.039	2.75E-17	14	MGAT4B
8.21E-22	1.318873807	0.58	0.143	2.75E-17	14	RETREG1
9.79E-22	0.980737049	0.54	0.118	3.28E-17	14	ATRX
1.52E-21	0.676188829	0.28	0.034	5.10E-17	14	GOLGA8B
2.26E-21	1.196651794	0.76	0.242	7.57E-17	14	PTH1R
2.29E-21	0.98710061	0.38	0.062	7.67E-17	14	DHRS3
2.30E-21	0.719274948	0.32	0.044	7.70E-17	14	BAZ2B
2.54E-21	1.145729856	0.58	0.135	8.52E-17	14	NPR3
3.10E-21	0.75241951	0.3	0.04	1.04E-16	14	CTNNBIP1
3.31E-21	0.651897776	0.26	0.03	1.11E-16	14	INPPL1
3.54E-21	0.880219053	0.46	0.087	1.19E-16	14	NR2F2
4.58E-21	1.185353198	0.68	0.2	1.54E-16	14	DDX17
9.46E-21	1.078556642	0.78	0.256	3.17E-16	14	GNAS
1.13E-20	0.860598429	0.26	0.031	3.80E-16	14	IGF1R
2.16E-20	0.812675936	0.26	0.032	7.23E-16	14	RALGAPA1
2.83E-20	0.819968442	0.28	0.037	9.49E-16	14	PCNX4
9.10E-20	1.216011584	0.66	0.205	3.05E-15	14	CAST
9.11E-20	1.272901515	0.86	0.479	3.06E-15	14	ACTG1
9.30E-20	0.588771279	0.26	0.032	3.12E-15	14	ACSL3
1.08E-19	0.985764651	0.38	0.067	3.61E-15	14	PON2
1.46E-19	1.190129929	0.66	0.204	4.91E-15	14	LEPROT
1.58E-19	0.719952569	0.26	0.033	5.29E-15	14	USP11
2.34E-19	0.872450367	0.34	0.054	7.85E-15	14	THBS1
5.93E-19	1.241917944	0.64	0.192	1.99E-14	14	AIF1L
6.04E-19	0.78812502	0.3	0.044	2.03E-14	14	PKP4
7.43E-19	0.807118949	0.44	0.088	2.49E-14	14	TMOD3
7.96E-19	1.195804284	0.46	0.103	2.67E-14	14	HIPK2

8.33E-19	0.828758304	0.28	0.039	2.79E-14	14	CRIP2
9.57E-19	1.092101059	0.66	0.2	3.21E-14	14	SRRM2
1.06E-18	1.021558689	0.36	0.063	3.57E-14	14	SUN2
1.52E-18	0.750376964	0.26	0.034	5.10E-14	14	WWC2
5.11E-18	1.250637939	0.6	0.179	1.71E-13	14	KTN1
5.88E-18	0.646889478	0.28	0.04	1.97E-13	14	FOXO3
6.16E-18	1.159874176	0.64	0.2	2.07E-13	14	LAMP1
8.76E-18	1.177522543	0.76	0.294	2.94E-13	14	CTSD
8.80E-18	0.719847451	0.36	0.064	2.95E-13	14	WWP1
1.37E-17	1.187244089	0.64	0.212	4.59E-13	14	CD59
1.39E-17	0.735730415	0.32	0.053	4.65E-13	14	SCARB2
1.45E-17	0.869161003	0.42	0.087	4.87E-13	14	SPTBN1
1.64E-17	0.672398527	0.28	0.041	5.50E-13	14	GNPTAB
1.93E-17	0.819454584	0.34	0.061	6.46E-13	14	ABI2
2.63E-17	0.773292455	0.3	0.048	8.83E-13	14	VGLL4
2.80E-17	0.824503496	0.5	0.121	9.38E-13	14	AKAP9
3.22E-17	0.835333861	0.34	0.061	1.08E-12	14	TTC14
3.30E-17	0.839085993	0.36	0.067	1.11E-12	14	TMEM248
3.69E-17	0.662915317	0.28	0.042	1.24E-12	14	THRA
5.15E-17	0.820876084	0.56	0.147	1.73E-12	14	LUC7L3
7.56E-17	0.782673398	0.3	0.049	2.54E-12	14	ZNF503
8.51E-17	1.013239651	0.78	0.296	2.86E-12	14	TXNIP
8.93E-17	0.874155121	0.36	0.07	2.99E-12	14	ARMCX3
9.30E-17	0.708560123	0.36	0.067	3.12E-12	14	PTBP3
1.01E-16	0.968236993	0.5	0.126	3.39E-12	14	LRRFIP1
1.39E-16	0.635680913	0.26	0.038	4.66E-12	14	ROCK2
2.25E-16	0.942149294	0.42	0.093	7.54E-12	14	COL18A1
2.38E-16	0.890498847	0.32	0.057	7.99E-12	14	HIPK3
2.49E-16	0.85025015	0.36	0.07	8.34E-12	14	UBE2G2
3.14E-16	0.892115902	0.48	0.121	1.05E-11	14	GOLGA4
4.59E-16	0.870090658	0.68	0.226	1.54E-11	14	SEPT7
4.86E-16	0.702349447	0.34	0.063	1.63E-11	14	CAND1
5.48E-16	0.720597163	0.34	0.063	1.84E-11	14	SMAD5
5.97E-16	0.69220973	0.32	0.057	2.00E-11	14	RB1CC1
5.99E-16	0.649541391	0.32	0.057	2.01E-11	14	IFNAR1
1.30E-15	0.69009427	0.3	0.052	4.35E-11	14	BNIP2
1.74E-15	0.669632208	0.32	0.059	5.83E-11	14	NIPBL
1.92E-15	0.783784613	0.38	0.081	6.45E-11	14	AKAP13
1.95E-15	1.09866304	0.58	0.186	6.54E-11	14	PDIA3
2.17E-15	0.89631638	0.5	0.133	7.26E-11	14	TSC22D3

2.40E-15	0.828904017	0.36	0.074	8.07E-11	14	TMEM259
2.75E-15	0.831049763	0.4	0.09	9.21E-11	14	PURA
2.94E-15	0.6393358	0.28	0.046	9.86E-11	14	TSPAN4
2.96E-15	0.545586912	0.26	0.04	9.92E-11	14	IL13RA1
4.25E-15	0.870160039	0.34	0.068	1.43E-10	14	PHIP
4.61E-15	0.835848848	0.4	0.091	1.55E-10	14	TSPAN3
6.38E-15	0.731315949	0.32	0.062	2.14E-10	14	MBD2
6.71E-15	0.896332842	0.38	0.085	2.25E-10	14	DNAJB14
7.27E-15	0.583674513	0.32	0.059	2.44E-10	14	FLNA
8.00E-15	0.838035956	0.3	0.055	2.68E-10	14	XPO1
8.75E-15	0.680394794	0.28	0.048	2.94E-10	14	APPBP2
9.28E-15	0.747040664	0.34	0.066	3.11E-10	14	SERPING1
9.86E-15	0.476994183	0.3	0.052	3.31E-10	14	CD9
1.03E-14	0.889214429	0.3	0.056	3.44E-10	14	ITM2C
1.14E-14	1.066331903	0.76	0.325	3.84E-10	14	HIST1H4C
1.22E-14	0.815502717	0.32	0.062	4.10E-10	14	TRIP11
1.22E-14	0.758373739	0.3	0.055	4.10E-10	14	CCNL2
1.30E-14	0.847218505	0.26	0.043	4.37E-10	14	SLC48A1
1.34E-14	0.706129685	0.4	0.091	4.50E-10	14	TPR
1.68E-14	0.944156082	0.34	0.07	5.63E-10	14	CCPG1
1.82E-14	0.996909739	0.62	0.215	6.10E-10	14	RDX
2.24E-14	0.735100329	0.92	0.459	7.52E-10	14	TMSB4X
2.32E-14	0.778036655	0.44	0.111	7.77E-10	14	WASF2
2.42E-14	0.845167199	0.28	0.05	8.11E-10	14	ZMYM2
2.50E-14	0.629909152	0.3	0.056	8.37E-10	14	PRRC2B
2.51E-14	0.945775213	0.4	0.094	8.41E-10	14	CPEB4
2.76E-14	0.699957534	0.42	0.1	9.25E-10	14	TBC1D1
3.61E-14	0.836061137	0.36	0.079	1.21E-09	14	SPPL2A
3.85E-14	0.679411162	0.32	0.062	1.29E-09	14	TIMP1
4.41E-14	0.762182622	0.36	0.08	1.48E-09	14	ERBIN
4.98E-14	0.697039535	0.3	0.057	1.67E-09	14	DCAF8
5.66E-14	0.751430245	0.34	0.071	1.90E-09	14	VPS13C
5.74E-14	0.873839767	0.38	0.088	1.93E-09	14	TMEM106B
5.81E-14	0.740353388	0.26	0.044	1.95E-09	14	USP9X
6.47E-14	0.702664585	0.48	0.13	2.17E-09	14	PRRC2C
7.51E-14	0.589565296	0.3	0.057	2.52E-09	14	NRIP1
7.70E-14	0.753832097	0.3	0.056	2.58E-09	14	EEA1
8.75E-14	0.860630064	0.34	0.073	2.94E-09	14	LBH
9.35E-14	0.726316534	0.38	0.088	3.14E-09	14	OS9
9.81E-14	0.624149965	0.26	0.044	3.29E-09	14	CSDC2

1.06E-13	0.919334755	0.5	0.145	3.56E-09	14	CITED2
1.23E-13	0.674673292	0.28	0.051	4.11E-09	14	NQO1
1.31E-13	0.577135249	0.28	0.051	4.38E-09	14	MAN1A2
1.33E-13	0.655271049	0.28	0.051	4.48E-09	14	TXNRD1
1.56E-13	0.76838976	0.4	0.098	5.23E-09	14	ZRANB2
1.73E-13	1.024806456	0.38	0.094	5.82E-09	14	TM9SF3
2.39E-13	0.844333622	0.64	0.228	8.02E-09	14	HNRNPU
3.39E-13	0.586306119	0.3	0.059	1.14E-08	14	MLLT6
3.41E-13	0.613300629	0.34	0.073	1.14E-08	14	CTNND1
3.77E-13	0.665568018	0.36	0.08	1.26E-08	14	H1FX
3.82E-13	1.043255787	0.6	0.221	1.28E-08	14	CANX
3.86E-13	0.717574191	0.36	0.083	1.29E-08	14	IQGAP1
4.16E-13	0.735303803	0.36	0.082	1.40E-08	14	HLA-F
4.21E-13	0.669447618	0.3	0.06	1.41E-08	14	MXD4
4.54E-13	0.740555786	0.3	0.06	1.52E-08	14	TRIM56
5.27E-13	0.5214308	0.26	0.046	1.77E-08	14	PHLDB2
5.79E-13	0.638723622	0.34	0.075	1.94E-08	14	WNK1
6.32E-13	0.734309616	0.34	0.075	2.12E-08	14	SMC3
6.36E-13	0.794710421	0.4	0.102	2.13E-08	14	TM9SF2
6.77E-13	0.575479666	0.52	0.151	2.27E-08	14	HSPA1A
7.84E-13	0.762491008	0.34	0.077	2.63E-08	14	GLUL
9.24E-13	0.603371529	0.3	0.061	3.10E-08	14	PTPN11
9.61E-13	0.622154855	0.28	0.054	3.22E-08	14	FNDC3B
1.07E-12	0.592773946	0.26	0.047	3.59E-08	14	NOC2L
1.12E-12	0.791257193	0.4	0.103	3.77E-08	14	PTTG1IP
1.21E-12	0.799217604	0.8	0.386	4.06E-08	14	SEC62
1.67E-12	0.619188058	0.34	0.076	5.59E-08	14	NUB1
1.88E-12	0.675806532	0.42	0.11	6.32E-08	14	KMT2E
2.05E-12	0.85188964	0.62	0.229	6.87E-08	14	ZBTB20
2.30E-12	0.684570311	0.26	0.049	7.70E-08	14	RHOQ
2.69E-12	0.924818952	0.38	0.1	9.03E-08	14	PJA2
5.75E-12	0.789055432	0.42	0.116	1.93E-07	14	CHD9
8.16E-12	0.622693188	0.34	0.079	2.74E-07	14	UBE2H
8.60E-12	0.447281063	0.26	0.049	2.89E-07	14	CBX1
8.74E-12	0.680074506	0.3	0.063	2.93E-07	14	ANXA1
9.02E-12	0.723538774	0.3	0.066	3.02E-07	14	SEC63
9.45E-12	0.469351675	0.26	0.05	3.17E-07	14	ARFGEF1
1.03E-11	0.736074128	0.36	0.09	3.47E-07	14	ADD1
1.06E-11	0.469109878	0.48	0.137	3.57E-07	14	NFE2L2
1.43E-11	0.764326632	0.26	0.052	4.81E-07	14	MAGT1

1.46E-11	1.022074624	0.58	0.223	4.89E-07	14	CALR
1.74E-11	0.770747341	0.4	0.111	5.85E-07	14	KIF5B
1.81E-11	0.715006302	0.34	0.084	6.07E-07	14	BPTF
1.91E-11	0.851384958	0.68	0.297	6.41E-07	14	SON
2.07E-11	0.630339353	0.28	0.059	6.94E-07	14	MCFD2
2.62E-11	0.604659333	0.28	0.059	8.78E-07	14	SIL1
2.83E-11	0.543954847	0.34	0.081	9.50E-07	14	TPM4
2.88E-11	0.766584958	0.5	0.164	9.67E-07	14	TSC22D1
2.91E-11	0.703326728	0.26	0.053	9.74E-07	14	IARS2
3.10E-11	0.787817261	0.5	0.166	1.04E-06	14	HP1BP3
3.90E-11	0.745674142	0.3	0.068	1.31E-06	14	PAWR
4.56E-11	0.602645666	0.26	0.053	1.53E-06	14	PTPRA
5.39E-11	0.827071258	0.38	0.108	1.81E-06	14	RAD21
6.12E-11	0.713581454	0.42	0.125	2.05E-06	14	EIF3A
6.64E-11	0.685099227	0.46	0.146	2.23E-06	14	ARGLU1
8.25E-11	0.672486683	0.38	0.104	2.77E-06	14	TAPBP
8.40E-11	0.693200862	0.32	0.078	2.82E-06	14	OSBPL9
9.70E-11	0.822136824	0.46	0.15	3.25E-06	14	NUCB1
1.18E-10	0.632687042	0.38	0.104	3.95E-06	14	PNN
1.41E-10	0.670123589	0.38	0.102	4.75E-06	14	LGALS1
1.57E-10	0.479655709	0.28	0.061	5.25E-06	14	FNBP1L
2.01E-10	0.442215469	0.98	0.84	6.73E-06	14	B2M
2.50E-10	0.533295981	0.3	0.07	8.37E-06	14	MT-ATP8
2.70E-10	0.748241492	0.32	0.082	9.06E-06	14	PCM1
3.83E-10	0.756880409	0.3	0.074	1.29E-05	14	WASHC1
5.83E-10	0.520020355	0.42	0.126	1.95E-05	14	ATF4
5.89E-10	0.638226368	0.3	0.075	1.97E-05	14	SH3BP5
7.92E-10	0.580781133	0.26	0.059	2.66E-05	14	HECTD1
9.35E-10	0.621419126	0.44	0.141	3.14E-05	14	P4HB
9.73E-10	0.631112522	0.38	0.109	3.26E-05	14	WSB1
1.03E-09	0.696931589	0.5	0.184	3.46E-05	14	GTF2A2
1.23E-09	0.687498039	0.36	0.106	4.13E-05	14	BRD2
1.55E-09	0.694736603	0.4	0.126	5.19E-05	14	PCMTD1
1.57E-09	0.594878372	0.3	0.077	5.26E-05	14	HEXIM1
1.60E-09	0.625628989	0.32	0.086	5.36E-05	14	CUX1
1.83E-09	0.604881864	0.44	0.141	6.15E-05	14	RFC1
2.72E-09	0.664387812	0.28	0.069	9.13E-05	14	SGCB
2.91E-09	0.59787064	0.36	0.105	9.77E-05	14	PPP2CB
3.10E-09	0.64672807	0.36	0.106	0.000104117	14	FBXO21
3.18E-09	0.579267449	0.32	0.088	0.000106789	14	CD2AP

4.15E-09	0.516142829	0.3	0.078	0.00013914	14	TMEM50B
4.19E-09	0.696881202	0.44	0.155	0.0001405	14	BBX
4.58E-09	0.680110198	0.58	0.249	0.000153512	14	VAMP2
4.78E-09	0.634173918	0.28	0.071	0.000160269	14	WWTR1
5.01E-09	0.643341546	0.32	0.089	0.000168039	14	APPL1
6.75E-09	0.809090222	0.44	0.164	0.00022631	14	GTF2I
7.03E-09	0.624944766	0.3	0.081	0.000235779	14	GLG1
7.55E-09	0.563858642	0.26	0.063	0.000253345	14	C16orf72
8.18E-09	0.546226241	0.26	0.063	0.000274455	14	SENP6
8.94E-09	0.750683584	0.26	0.065	0.000299839	14	C1orf115
1.04E-08	0.781164782	0.42	0.148	0.000349457	14	PRMT2
1.16E-08	0.577971205	0.26	0.064	0.00038898	14	RASSF7
1.37E-08	0.757892326	0.58	0.253	0.00046004	14	EZR
1.38E-08	0.625210362	0.28	0.075	0.000462984	14	DDB1
1.49E-08	0.509070659	0.26	0.065	0.000501288	14	TUT4
1.68E-08	0.656879337	0.4	0.135	0.000563774	14	HNRNPR
2.21E-08	0.756018041	0.26	0.068	0.000740243	14	ITFG1
2.50E-08	0.605024589	0.3	0.084	0.000840028	14	ARID1B
2.74E-08	0.774778951	0.28	0.078	0.000917573	14	BRD4
3.48E-08	0.622771652	0.28	0.078	0.001166332	14	ROCK1
3.73E-08	0.507569846	0.4	0.135	0.001252123	14	MCL1
3.87E-08	0.682370288	0.36	0.119	0.001297917	14	TLN1
4.07E-08	0.419811402	0.26	0.066	0.001364103	14	SMAD2
4.36E-08	0.536544361	0.48	0.176	0.001462264	14	APP
4.38E-08	0.579474084	0.26	0.068	0.001468779	14	EFCAB14
4.40E-08	0.54518184	0.26	0.067	0.001476607	14	PDXDC1
4.59E-08	0.646559431	0.36	0.115	0.001538482	14	RPN1
4.86E-08	0.451418046	0.28	0.076	0.001630775	14	MECP2
5.19E-08	0.645581194	0.3	0.088	0.001740933	14	ASH1L
5.71E-08	0.504089942	0.26	0.068	0.001914745	14	PPP2R5E
6.60E-08	0.557271604	0.32	0.098	0.002212908	14	TM2D1
8.67E-08	0.666795051	0.32	0.099	0.002907807	14	DYNC1H1
8.72E-08	0.442599503	0.28	0.078	0.002925137	14	ZNF148
9.77E-08	0.654953647	0.32	0.099	0.00327514	14	UGCG
1.06E-07	0.722101347	0.42	0.157	0.003565614	14	CTNNA1
1.08E-07	0.541342266	0.86	0.655	0.00360759	14	DSTN
1.16E-07	0.565352794	0.28	0.08	0.0038901	14	TMEM9B
1.23E-07	0.561154748	0.26	0.072	0.004114947	14	ERLEC1
1.26E-07	0.805532141	0.46	0.192	0.004221028	14	ADD3
1.26E-07	0.551449188	0.3	0.089	0.004242514	14	TROVE2

1.42E-07	0.633046162	0.3	0.09	0.004759136	14	ZBTB38
1.53E-07	0.564628211	0.34	0.112	0.005145131	14	TMED9
1.60E-07	0.652146907	0.42	0.159	0.005380906	14	SFPQ
1.65E-07	0.576150516	0.74	0.416	0.005523386	14	HNRNPK
1.77E-07	0.441527426	0.26	0.071	0.005943657	14	IDS
1.95E-07	0.530091172	0.64	0.309	0.006535748	14	SQSTM1
1.96E-07	0.604719309	0.28	0.082	0.006557741	14	CHD4
2.07E-07	0.695782559	0.28	0.085	0.00695571	14	SDF4
2.21E-07	0.535050507	0.32	0.102	0.007409332	14	PRNP
2.60E-07	0.681354148	0.28	0.084	0.008727334	14	PHF3
2.83E-07	0.631280369	0.48	0.201	0.009482922	14	SPINT2
2.91E-07	0.541948727	0.56	0.25	0.009747705	14	DYNC1I2
3.51E-07	0.691597912	0.52	0.252	0.011777425	14	EIF3M
3.51E-07	0.576283052	0.28	0.086	0.011782509	14	UBE3A
3.61E-07	0.583131963	0.26	0.076	0.012095528	14	ZFR
3.74E-07	0.651773582	0.44	0.18	0.012534441	14	SEPT2
3.80E-07	0.735742546	0.34	0.119	0.01275766	14	AP2B1
3.84E-07	0.514545772	0.28	0.084	0.012870676	14	RTL8C
4.02E-07	0.699751336	0.28	0.086	0.013473256	14	RSF1
4.16E-07	0.446586432	0.28	0.083	0.013951118	14	ARHGAP5
5.50E-07	0.550934295	0.7	0.396	0.01843944	14	EID1
5.57E-07	0.593836953	0.34	0.119	0.018668748	14	RPN2
5.68E-07	0.574874836	0.32	0.108	0.019047683	14	SSR1
5.94E-07	0.422452752	0.32	0.104	0.019914889	14	DHX36
6.85E-07	0.582074986	0.26	0.077	0.022987037	14	NPEPPS
7.10E-07	0.622172224	0.42	0.166	0.023801749	14	HNRNPM
7.49E-07	0.495732493	0.32	0.105	0.025131335	14	NR3C1
7.88E-07	0.610575294	0.26	0.077	0.026416481	14	C8orf33
8.17E-07	0.557104524	0.26	0.078	0.027391953	14	LRP10
8.86E-07	0.492100933	0.26	0.078	0.029730385	14	ZNF106
1.02E-06	0.727265077	0.5	0.237	0.034163585	14	H2AFJ
1.03E-06	0.436049499	0.26	0.077	0.0345703	14	SMARCA5
1.04E-06	0.711073934	0.26	0.081	0.034763347	14	DYNC1LI2
1.16E-06	0.55554452	0.34	0.121	0.038755294	14	CDV3
1.20E-06	0.503638507	0.4	0.155	0.040236081	14	CSNK1A1
1.35E-06	0.65983156	0.32	0.113	0.045141724	14	GNB1
1.36E-06	0.42071737	0.3	0.098	0.045763857	14	SRSF1
1.38E-06	0.572822436	0.28	0.09	0.046312562	14	RNF19A
1.52E-06	0.541221648	0.26	0.08	0.050850125	14	LARP1
1.68E-06	0.454381133	0.34	0.121	0.056184661	14	NAP1L4

1.70E-06	0.416921643	0.26	0.078	0.057145076	14	NBR1
1.71E-06	0.572473506	0.56	0.275	0.057413858	14	DDX24
1.87E-06	0.635072538	0.26	0.081	0.062586936	14	EML4
1.90E-06	0.502277932	0.32	0.113	0.063827518	14	NCOR1
1.91E-06	0.433990812	0.3	0.099	0.064164754	14	PRPF6
2.34E-06	0.55509411	0.36	0.134	0.078580449	14	VAMP5
2.42E-06	0.591371096	0.5	0.242	0.081040137	14	HNRNPA3
2.83E-06	0.537985159	0.36	0.135	0.095021097	14	FXR1
2.84E-06	0.688158575	0.36	0.141	0.095278009	14	SNRNP70
2.97E-06	0.497638009	0.38	0.148	0.099533031	14	SF3B1
3.07E-06	0.500626157	0.4	0.163	0.102887492	14	SH3GLB1
3.35E-06	0.617528767	0.6	0.338	0.112320422	14	HNRNPDL
3.52E-06	0.632923177	0.3	0.106	0.117905017	14	SELENOS
3.55E-06	0.650037523	0.26	0.084	0.119213164	14	MSI2
3.67E-06	0.528534402	0.68	0.387	0.123187038	14	DDX5
3.74E-06	0.488013852	0.28	0.091	0.125495903	14	STARD7
3.90E-06	0.54838981	0.4	0.163	0.130800014	14	CLTC
4.03E-06	0.856780792	0.36	0.147	0.135263303	14	SDC4
4.18E-06	0.487243002	0.3	0.104	0.140233437	14	WAC
4.49E-06	0.559445881	0.26	0.083	0.150424787	14	IL6ST
4.92E-06	0.403158869	0.28	0.092	0.164948699	14	CYB5B
5.08E-06	0.46456147	0.28	0.094	0.170377354	14	CBX5
5.74E-06	0.488928141	0.46	0.204	0.192508865	14	N4BP2L2
5.82E-06	0.554224123	0.78	0.488	0.195349338	14	HNRNPA2B1
5.83E-06	0.552164833	0.66	0.379	0.19536084	14	IFITM3
6.89E-06	0.467807749	0.36	0.139	0.231124885	14	LMNA
7.11E-06	0.571245176	0.3	0.109	0.238311485	14	RSRP1
7.90E-06	0.604820806	0.58	0.328	0.265042495	14	SARAF
8.04E-06	0.437192122	0.3	0.107	0.269680327	14	LMAN1
8.07E-06	0.408885281	0.26	0.085	0.27054814	14	YME1L1
9.08E-06	0.380045003	0.36	0.135	0.304577668	14	CTSL
1.00E-05	0.545226868	0.4	0.168	0.336541842	14	FUS
1.09E-05	0.729757424	0.26	0.09	0.365748566	14	ILF3
1.09E-05	0.722055419	0.36	0.153	0.366744067	14	HNRNPH1
1.23E-05	0.402306789	0.3	0.108	0.411138596	14	EIF4H
1.31E-05	0.51098285	0.36	0.15	0.4390453	14	YY1
1.35E-05	0.502445576	0.28	0.101	0.451869863	14	KPNB1
1.50E-05	0.486457234	0.26	0.089	0.504619713	14	RRBP1
1.52E-05	0.823363757	0.36	0.156	0.510350783	14	ZFAND5
1.65E-05	0.430541017	0.42	0.18	0.554533385	14	NUPR1

1.68E-05	0.458679434	0.32	0.122	0.563180372	14	CYB5R3
1.81E-05	0.523953657	0.44	0.205	0.608390233	14	CXXC5
1.83E-05	0.604038527	0.28	0.102	0.614418454	14	PSIP1
2.30E-05	0.609207772	0.26	0.092	0.77255175	14	DNAJC3
2.56E-05	0.401593961	0.3	0.11	0.857173666	14	SLC7A8
2.59E-05	0.521903805	0.34	0.143	0.868194486	14	SEC11C
2.60E-05	0.473711564	0.34	0.139	0.872803357	14	HDLBP
2.65E-05	0.478745822	0.28	0.101	0.887288973	14	PARD6B
3.02E-05	0.429463171	0.32	0.124	1	14	CKB
3.12E-05	0.410199123	0.44	0.206	1	14	UBXN4
3.44E-05	0.662484627	0.34	0.147	1	14	HNRNPD
3.96E-05	0.419252906	0.32	0.127	1	14	CFAP97
4.23E-05	0.38440304	0.26	0.093	1	14	ATXN10
4.88E-05	0.496957725	0.28	0.108	1	14	GNAI2
5.06E-05	0.630015768	0.32	0.139	1	14	TGOLN2
5.13E-05	0.498398925	0.28	0.105	1	14	COMT
5.21E-05	0.556899187	0.36	0.161	1	14	SCAF11
6.45E-05	0.630250386	0.38	0.183	1	14	PDIA6
7.01E-05	0.360891737	0.26	0.095	1	14	DDX6
7.34E-05	0.499719787	0.26	0.098	1	14	SEC31A
7.73E-05	0.387179554	0.52	0.269	1	14	CALD1
8.66E-05	0.606666946	0.44	0.223	1	14	SSB
9.45E-05	0.515146942	0.34	0.151	1	14	VCP
0.00010233	0.361390945	0.54	0.287	1	14	CNBP
0.000103602	0.396545642	0.28	0.111	1	14	RBM25
0.000107006	0.47115604	0.32	0.139	1	14	GPBP1
0.000107644	0.436084673	0.44	0.215	1	14	OCIAD1
0.000113038	0.271705111	0.86	0.569	1	14	HSP90AA1
0.000124851	0.36834088	0.4	0.184	1	14	IGFBP4
0.000143273	0.484901186	0.38	0.183	1	14	DEK
0.000163585	0.591556173	0.26	0.1	1	14	CERS2
0.000175436	0.367055873	0.84	0.603	1	14	HMGB1
0.000183823	0.427253958	0.28	0.116	1	14	G3BP1
0.000193668	0.344709677	0.26	0.101	1	14	UPF3A
0.000198211	0.361324582	0.46	0.231	1	14	YWHAZ
0.000199428	0.426770024	0.4	0.195	1	14	FYN
0.000210129	0.405847326	0.32	0.14	1	14	IFITM2
0.000226691	0.53109626	0.36	0.17	1	14	TMED10
0.00025186	0.426157421	0.3	0.127	1	14	RCAN1
0.000253156	0.373092685	0.56	0.314	1	14	NCL

0.000270092	0.396664861	0.3	0.13	1	14	TNRC6B
0.000274169	0.589689011	0.36	0.172	1	14	PNISR
0.000313111	0.449959663	0.3	0.134	1	14	MPHOSPH8
0.000315856	0.549618534	0.28	0.126	1	14	LSM14A
0.000345008	0.452972193	0.28	0.119	1	14	PLEKHA5
0.000349444	0.397674246	0.3	0.133	1	14	PRPF4B
0.000349613	0.350712101	0.34	0.155	1	14	PNRC2
0.000366468	0.351555374	0.3	0.131	1	14	AAK1
0.000405965	0.400381813	0.26	0.109	1	14	SRSF4
0.000441038	0.383448079	0.28	0.121	1	14	RAD23B
0.000443737	0.483376706	0.3	0.137	1	14	TAF7
0.000447154	0.379702357	0.28	0.12	1	14	WASHC3
0.000486535	0.413664136	0.26	0.108	1	14	RBBP4
0.000525336	0.33200446	0.32	0.145	1	14	LARP7
0.000532381	0.502109746	0.46	0.267	1	14	MRFAP1
0.000541582	0.411656643	0.32	0.151	1	14	LMAN2
0.000581169	0.442013892	0.34	0.17	1	14	CFLAR
0.00061108	0.393289761	0.26	0.11	1	14	CMTM6
0.00061595	0.301297599	0.68	0.417	1	14	MT-ND4L
0.000618025	0.354277987	0.68	0.52	1	14	CIRBP
0.00066235	0.380967516	0.42	0.23	1	14	PRKAR1A
0.000668453	0.424778539	0.3	0.138	1	14	PSMB9
0.000684642	0.468898791	0.36	0.183	1	14	TMCO1
0.000762987	0.349230763	0.3	0.139	1	14	CIR1
0.000786128	0.63366348	0.48	0.291	1	14	GADD45A
0.000813444	0.364916473	0.26	0.112	1	14	CEBPZ
0.00081618	0.420429926	0.26	0.113	1	14	NSRP1
0.000818415	0.400353096	0.54	0.314	1	14	SRSF3
0.000819165	0.444349221	0.34	0.168	1	14	CAPNS1
0.000857128	0.447009302	0.7	0.484	1	14	YWHAE
0.000907169	0.318487528	0.32	0.151	1	14	CPNE3
0.000970811	0.47305477	0.4	0.22	1	14	ARF6
0.001020316	0.344622498	0.26	0.114	1	14	PICALM
0.001038487	0.413808885	0.38	0.203	1	14	TSPYL1
0.001062423	0.31356228	0.42	0.228	1	14	DNAJA1
0.001154002	0.47337154	0.58	0.355	1	14	NUCKS1
0.001185593	0.520884272	0.28	0.132	1	14	SF1
0.001222344	0.472690106	0.36	0.193	1	14	BEX4
0.001319294	0.352638046	0.3	0.14	1	14	RNF11
0.001331813	0.420300886	0.32	0.16	1	14	MTDH

0.001399651	0.260902781	0.32	0.154	1	14	PSMB8
0.001456523	0.275944076	0.54	0.322	1	14	NAP1L1
0.001520811	0.456935578	0.28	0.136	1	14	SRSF10
0.001561264	0.492354671	0.36	0.195	1	14	VKORC1
0.001604053	0.303971171	0.28	0.13	1	14	MED4
0.001615025	0.416974039	0.5	0.299	1	14	SRSF5
0.001796985	0.250327449	0.26	0.116	1	14	OAZ2
0.001877721	0.372113892	0.26	0.12	1	14	NMT1
0.001984565	0.33890265	0.26	0.121	1	14	NBEAL1
0.002145762	0.28720153	0.26	0.118	1	14	SP100
0.002202556	0.322816943	0.26	0.12	1	14	RPL7L1
0.002363549	0.4255067	0.28	0.134	1	14	TERF2IP
0.002426044	0.411754665	0.36	0.201	1	14	NDFIP1
0.002447872	0.31584858	0.3	0.146	1	14	RALBP1
0.002586606	0.482445959	0.32	0.171	1	14	ACTR2
0.00261109	0.478325067	0.74	0.64	1	14	CALM2
0.00277674	0.432213085	0.52	0.354	1	14	PTGES3
0.002977229	0.364690995	0.28	0.141	1	14	SLTM
0.003147509	0.425669508	0.34	0.189	1	14	G0S2
0.003374558	0.400864128	0.26	0.129	1	14	HNRNPH3
0.003417413	0.385191118	0.34	0.185	1	14	NME3
0.003484381	0.432383483	0.3	0.155	1	14	ATP6AP2
0.003774962	0.37834166	0.26	0.128	1	14	USP16
0.004102608	0.269616533	0.28	0.136	1	14	FAM133B
0.004209262	0.368621161	0.26	0.13	1	14	THRAP3
0.004298609	0.370250256	0.28	0.142	1	14	MLEC
0.004957231	0.38871751	0.32	0.179	1	14	UBE2I
0.005243857	0.310052133	0.3	0.157	1	14	LITAF
0.005314409	0.395845284	0.52	0.357	1	14	C11orf58
0.005640512	0.311536791	0.32	0.172	1	14	OPTN
0.005904528	0.274835305	0.38	0.216	1	14	RBPMS
0.006023556	0.38482838	0.44	0.276	1	14	CSDE1
0.007108162	0.462968987	0.38	0.228	1	14	TCF25
0.007440745	0.330756254	0.68	0.503	1	14	CCNI
0.007902297	0.280074327	0.4	0.243	1	14	RBM39
0.008288414	0.392137114	0.26	0.137	1	14	CTSZ
0.008484965	0.267071525	0.4	0.24	1	14	EIF3J
0.008567919	0.283279797	0.3	0.16	1	14	PFKL
0.008778011	0.337184622	0.34	0.198	1	14	ARHGDIA
0.00958965	0.348870645	0.26	0.135	1	14	EI24

Table S5. Subpopulation characteristics for the four cell types.

Cluster	Annotated name	Cells (Total)	CKTR	Normal	Marker genes	Violin plot or tSNE plot (ssGSEA)
NKT Cluster0	CD4+ T cells	638	281	357	LTB, IL7R	REACTOME_SELENOAMINO_ACID_METABOLISM
NKT Cluster1	CD8+ T cells	499	471	28	CD8A, CD8B	GO_MHC_CLASS_II_PROTEIN_COMPLEX, GO_MHC_CLASS_II_RECEPTOR_ACTIVITY
NKT Cluster2	cytotoxic T lymphocyte (CTL)	498	131	367	GNLY, GZMB	GO_CCR1_CHEMOKINE_RECEPTOR_BINDING, GO_GRANZYME_MEDIATED_APOPTOTIC_SIGNALING_PATHWAY
NKT Cluster3	Tregs	192	182	10	FOXP3, CTLA4	GO_NEGATIVE_REGULATION_OF_INTERLEUKIN_4_PRODUCTION, GO_NEGATIVE_REGULATION_OF_INTERLEUKIN_5_PRODUCTION
NKT Cluster4	NKs	146	9	137	MT1X, MT2A	GO_POSITIVE_REGULATION_OF_NATURAL_KILLER_CELL_CHEMOTAXIS
B-cell Cluster0	Memory B-cells	562	556	6	Single R package	GO_B_CELL_PROLIFERATION_INVOLVED_IN_IMMUNE_RESPONSE,GO_REGULATION_OF_B_CELL_RECEPTOR_SIGNALING_PATHWAY,GO_B_CELL_LINEAGE_COMMITMENT, REACTOME_ANTIGEN_ACTIVATES_B_CELL_RECEPTOR_BCR_LEADING_TO_GENERATION_OF_SECOND_MESSENGERS
B-cell Cluster1	naive B-cells	442	354	88	Single R package	NA
B-cell Cluster2	Class-switched memory B-cells	89	84	5	Single R package	NA
B-cell Cluster3	Plasma cells	87	76	11	Single R package	NA
B-cell Cluster4	Memory B-cells	87	51	36	Single R package	NA
Monocytes Cluster0	CD14+Monocytes	403	179	224	CD163, CD14	GO_MHC_CLASS_II_PROTEIN_COMPLEX,KEGG_ALLOGRAFT_REJECTION,KEGG_ANTIGEN_PROCESSING_AND_PRESENTATION
Monocytes Cluster1	CD14+Monocytes	324	53	271	S100A12, CD14	GO_SHORT_CHAIN_FATTY_ACID_CATABOLIC_PROCESS,GO_FATTY

						_ACID_HOMEOSTASIS
Monocytes Cluster2	CD16+Monocytes	190	16	174	LILRA1, FCGR3A(CD16)	NA
Monocytes Cluster3	CD14+Monocytes	48	4	33	SELL, CD14	GO_INTERLEUKIN_10_BIOSYNTHETIC_PROCESS,GO_INTERLEUKIN_1_BIOSYNTHETIC_PROCESS,GO_POSITIVE_REGULATION_OF_INTERLEUKIN_8_SECRETION
Monocytes Cluster4	Myeloid dendritic cells	46	38	8	XCR1, CLEC9A	NA
Fibroblasts Cluster0	Cluster0	151	148	3	Calculate score across top 10 differentially expressed genes (DEGs) among samples using IMvigor210CoreBiologies R package	GO_COLLAGEN_BINDING_INVOLVED_IN_CELL_MATRIX_ADHESION_REACTOME_SIGNALING_BY_PDGF
Fibroblasts Cluster1	Cluster1	46	11	35	Calculate score across top 10 differentially expressed genes (DEGs) among samples using IMvigor210CoreBiologies R package	REACTOME_SIGNALING_BY_FGFR2_IIIA_TM_REACTOME_TGF_BETA_RECEPTOR_SIGNALING_IN_EMT_EPITHELIAL_TO_MESENCHYMAL_TRANSITION
Fibroblasts Cluster2	Cluster2	29	29	0	Calculate score across top 10 differentially expressed genes (DEGs) among samples	GO_POSITIVE_REGULATION_OF_CELLULAR_BIOSYNTHETIC_PROCESS

		using<br>IMvigor210C<br>oreBiologies<br>R package

NA, not available
